# An Introductory Point-of-Care Ultrasound Curriculum for an Anesthesiology Residency Program

**DOI:** 10.15766/mep_2374-8265.11291

**Published:** 2022-12-23

**Authors:** Susan C. Lee, Edward C. Yang, Jovany Cruz Navarro, Charles G. Minard, Xiaofan Huang, Yi Deng

**Affiliations:** 1 Associate Professor, Department of Anesthesiology, Division of Trauma Anesthesiology, Baylor College of Medicine; 2 Assistant Professor, Department of Anesthesiology, Division of Cardiovascular Anesthesiology and Critical Care, Baylor College of Medicine; 3 Assistant Professor, Department of Anesthesiology, Division of Trauma Anesthesiology and Division of Neuroanesthesiology and Critical Care, Baylor College of Medicine; 4 Associate Professor, Dan L. Duncan Institute for Clinical and Translational Research, Baylor College of Medicine; 5 Biostatistician, Dan L. Duncan Institute for Clinical and Translational Research, Baylor College of Medicine; 6 Assistant Professor, Department of Anesthesiology, Division of Trauma Anesthesiology and Division of Cardiovascular Anesthesiology and Critical Care, Baylor College of Medicine

**Keywords:** Point-of-Care Ultrasound, Ultrasonography, Anesthesiology, Clinical/Procedural Skills Training, Ultrasound Skills

## Abstract

**Introduction:**

The use of point-of-care ultrasound (POCUS) is a growing trend in the field of anesthesiology. However, formal POCUS curriculums are still not widely implemented in residency programs. As the Accreditation Council for Graduate Medical Education and the American Board of Anesthesiology have both incorporated POCUS into their educational aims and expectations for graduates, we recognized the need for a formal POCUS curriculum for our residency program. We developed and implemented a comprehensive 3-week POCUS curriculum for our first-year anesthesiology residents (CA1s) in the latter half of their academic year.

**Methods:**

Twenty CA1s participated in this educational activity. The POCUS curriculum spanned seven topics and was given in weekly 2-hour sessions over the course of 3 weeks. Each session was designed with the first hour consisting of a traditional lecture-based presentation followed by live hands-on practice. A pretest on POCUS knowledge was given to every resident before the curriculum, and a posttest and survey were administered afterwards.

**Results:**

Every CA1 showed an improvement in their posttest scores. The median scores of the pretest and posttest were 49% and 75%, respectively. Survey results were positive, with all of the CA1s agreeing that the POCUS educational materials were appropriate to their level of training and that their POCUS knowledge and technical skills improved after the curriculum.

**Discussion:**

We have shown that our formal POCUS curriculum improved anesthesiology residents’ knowledge as well as resulting in positive views on the implementation of this intervention.

## Educational Objectives

By the end of this activity, learners will be able to:

1. Describe the basic principles and functions of the ultrasound.
2. Identify normal anatomic structures in lung, cardiac, vascular access, airway, abdominal, and neuraxial examinations of adult patients using a portable ultrasound machine.
3. Modify scanning maneuvers to improve the acquired image.
4. Deduce potential pathologies.

## Introduction

Point-of-care ultrasound (POCUS) can be used to better serve patients clinically by providing real-time images that potentially result in faster diagnoses and guidance of clinical decisions.^1^ However, a major barrier to the use of POCUS is the lack of training and curricula.^2^ *MedEdPORTAL* currently features POCUS content for adult and pediatric critical care that focuses mainly on cardiac and lung exams.^3–5^ While these are important, a more comprehensive POCUS curriculum for anesthesiologists is needed to address areas specific to this specialty. These areas include, but are not limited to, gastric ultrasound to assess aspiration risk, airway ultrasound to delineate the cricothyroid membrane in a difficult airway patient, and neuraxial ultrasound to assist in epidural placement.

POCUS has seen a huge rise in popularity in the last few years across many specialties. Anesthesiologists have traditionally been leaders in the use of ultrasound technology for nerve blocks, central venous access, and intraoperative transesophageal echocardiography. However, mastery of a comprehensive POCUS examination of the whole body appears to be rare based on the paucity of evidence in the literature. A formal POCUS curriculum for anesthesiology residents is not widely available and/or is limited to systems such as cardiac or pulmonary.^6,7^

In recent years, two governing bodies, the Accreditation Council for Graduate Medical Education (ACGME) and the American Board of Anesthesiology (ABA), have both included POCUS in their educational aims and expectations for graduates.^8,9^ The ACGME program requirements for graduate medical education in anesthesiology include understanding the physics and principles of ultrasound, being competent in obtaining cardiac views and lung pathology, and using information obtained with ultrasound to guide invasive procedures.^8^ Recently, the ABA began incorporating POCUS exam questions into the objective structured clinical examination (OSCE) portion of the APPLIED Examination, which must be passed for board certification.^9^

Developing a formal POCUS curriculum was a priority for our residency program. We wanted to implement the most comprehensive curriculum specific to anesthesiology so residents could achieve a proficiency in utilizing POCUS in clinical practice, enhance their competency in patient care and safety, and successfully meet the emerging board and regulatory requirements expected of our profession. We designed and implemented a complete curriculum covering all major organ systems with relevant applications for perioperative care for a practicing anesthesiologist. We conducted this pilot program once, collected data, and used peer review feedback for improvements before running it in earnest for the academic year 2022.

We developed a new, comprehensive 3-week POCUS curriculum for all first-year anesthesiology residents (CA1s) at the Baylor College of Medicine. Our goal was to investigate whether a standardized, formal curriculum would improve resident knowledge of POCUS.

## Methods

This educational intervention was approved by the Institutional Review Board at Baylor College of Medicine (no. H-47035, March 10, 2020) and scheduled during designated lecture time as a weekly 2-hour session over 3 consecutive weeks in March 2022. The target audience was 20 CA1s in their second year of postgraduate training. The CA1s were selected so they could build and practice POCUS skills throughout their training. One of the new ACGME milestones for anesthesiology was POCUS starting at the beginning of training.^10^

The majority of CA1s did not have any previous POCUS knowledge/training, and no prerequisite knowledge was required prior to this course. The POCUS curriculum was developed by three physicians who were all board certified in both anesthesiology and critical care and had extensive knowledge of and experience with POCUS. No senior residents helped with this course. The comprehensive curriculum was divided into 3 weeks and included seven topics:

• Week 1: (1) basic ultrasound operation and physics (Appendix A), (2) focused lung (Appendix B);
• Week 2: (3) focused cardiac (Appendix C); and
• Week 3: (4) vascular access (Appendix D), (5) airway (Appendix E), (6) abdominal (Appendix F), and (7) neuraxial (Appendix G).

The only equipment used for this course was a computer, a projector, and four ultrasound machines. No other materials were provided to the residents. Each weekly session started with a 1-hour traditional lecture-based presentation led by a faculty anesthesiologist. The second hour was dedicated to hands-on practice to reinforce topics addressed in the lecture. We split the 20 CA1s into four stations of five participants each, with each group allotted one ultrasound for scanning on a live model. The average scan time per resident per station was 10 minutes. Each of the stations had a checklist of required views that the residents had to obtain prior to advancing (Appendix H). A faculty instructor was present at each station to ensure successful completion of the checklist per resident. For further engagement, the instructors also elicited potential pathologies covered during lecture from residents during live scanning (as models were healthy volunteers).

At the beginning of the course, a pretest of 20 multiple-choice questions in PowerPoint format was given to the participants for knowledge assessment (Appendix I). The pretest included questions pertaining to the content of the POCUS material, and images and/or videos were used when appropriate. Residents had 1 minute to answer each question, and the use of outside resources was not permitted. The tests were graded, and the scores were tabulated in masked fashion. We did not provide residents with the answer key to review the test because we wanted them to learn the correct answers with explanations during the course and to retain the information subsequently.

At the end of the 3 weeks, all participants took a posttest of 20 multiple-choice questions (Appendix J) to assess their knowledge, as well as a survey of seven questions to evaluate the course material and perceived usefulness/comfort regarding POCUS (Appendix K). The posttest questions had knowledge content and difficulty similar to the pretest. We applied the same time limit, and no outside resources were permitted. Grading and tabulating scores were done in a masked fashion as well. The survey data were collected anonymously.

We estimated the proportion of participants who improved with a 95% exact, binomial confidence interval. Scores were summarized by medians with 25th and 75th percentiles. We tested the null hypothesis that there would be no change in score using the Wilcoxon signed rank test and assessed statistical significance the .05 level. Survey results were summarized by frequencies with percentages.

## Results

**Figure 1.**
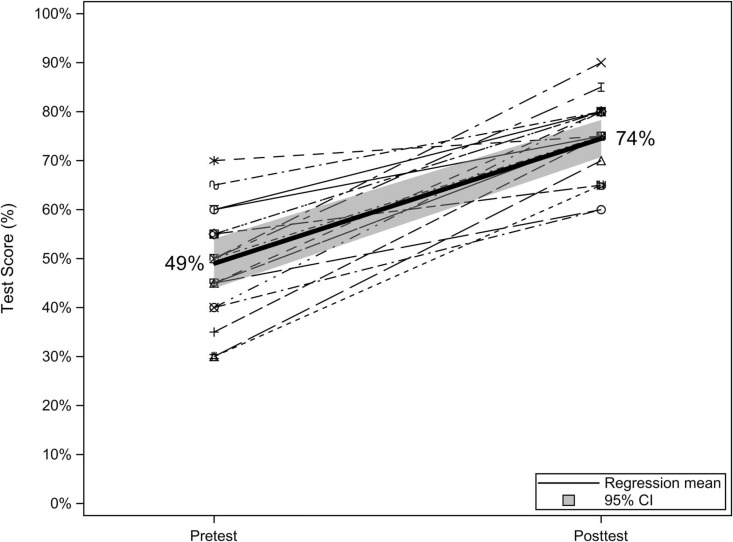
Pretest versus posttest knowledge scores for residents who completed the point-of-care ultrasound curriculum (*N* = 20).

All 20 CA1s in the program showed an improvement in their posttest scores, with mean test scores improving from 49% (95% CI, 44%-54%) before the intervention to 74% (95% CI, 71%-78%) after the intervention (Figure 1). On average, test scores improved by 25.5. points (95% CI, 20.2-30.8).

**Figure 2.**
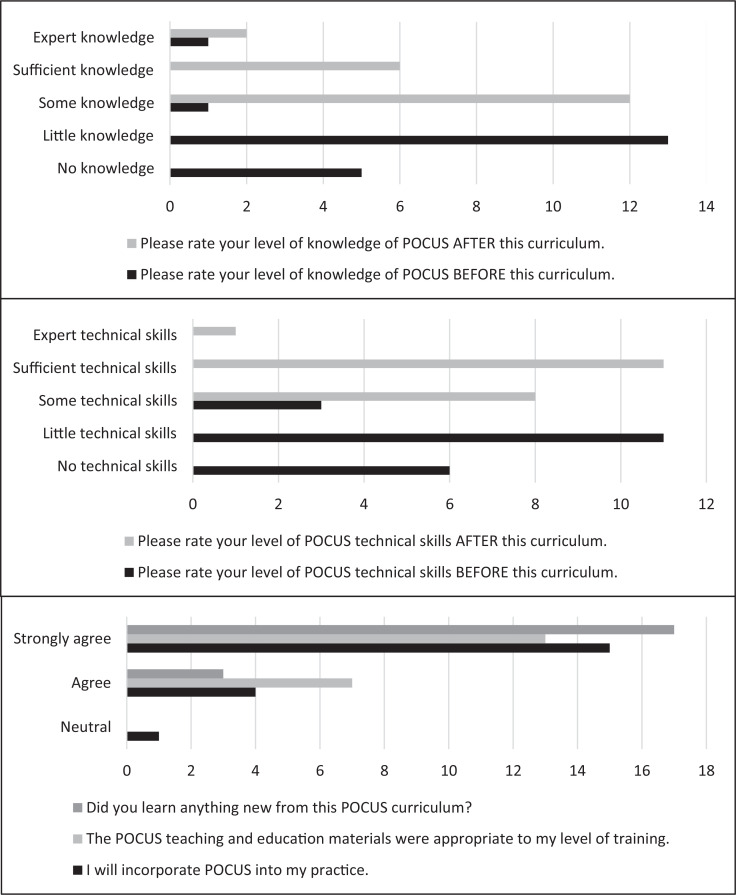
Survey results (*N* = 20). Abbreviation: POCUS, point-of-care ultrasound.

The survey results showed that before the curriculum, 90% of the CA1s perceived themselves as having little to no knowledge of POCUS. However, after the curriculum, 100% of the CA1s rated themselves with some, sufficient, or expert knowledge of POCUS (Figure 2).

In regard to POCUS technical skills, 85% of the CA1s rated themselves as having little to no technical skills before the curriculum. The percentage of residents who felt they gained some, sufficient, or expert technical skills increased to 100% after the completion of the curriculum.

Overall, all the CA1s felt that they learned something new from this POCUS curriculum and that the POCUS teaching and educational materials were appropriate to their level of training. Ninety-five percent agreed that they would incorporate POCUS into their practice.

## Discussion

With the growing trend towards the use of POCUS for patient care as well as accreditation bodies requiring the acquisition of these skills, the need for anesthesiology residency programs to incorporate a formal POCUS curriculum is emerging. The majority of our CA1 residents had limited POCUS knowledge and experience prior to participating in the course. By implementing this new curriculum in our anesthesiology residency program, we have shown that it significantly increased residents’ POCUS knowledge.

The POCUS curriculum was also in alignment with the new milestones for residency and OSCE skills assessment required for board certification. The advantage of introducing the course in the CA1 year was that residents could build upon the skills and knowledge gained over the next 2 years of residency. This, perhaps, could prepare them for the ABA OSCE and increase their confidence in passing it.

Although more of our faculty are using POCUS in clinical practice, it is difficult to assess and/or standardize their knowledge and skills. Some may be more proficient in one area versus another. Many faculty attend POCUS courses offered within our department as well as nationally. Ultrasound machines are available on all rotations in every institution. As residents work with many different faculty during their residency, they have the opportunity to learn every aspect of POCUS.

Our educational activity has several limitations. First, the sample size was relatively small, which limited statistical power. We were constrained by the number of current CA1s in our anesthesiology residency program. In subsequent years, we have the potential to add more participants to increase our sample size.

Second, only knowledge of POCUS was tested, as opposed to a practicum exam, which could show whether residents were able to properly scan and obtain images in an exam environment.

Additionally, the hands-on training was performed in a controlled classroom setting with healthy models and not real patients. Hence, only normal views were obtained during the instructional sessions. Although pathologies were covered during the didactics portion, the CA1s did not have the opportunity to scan for these views on live patients. Furthermore, the live models were scanned in optimal conditions without challenging factors such as large body habitus or poor positioning.

Since the exam was given shortly after teaching, retention of the material was not factored. Repeat exams in 3 or 6 months would be a stronger assessment of retention of the material. Hubert et al. studied the effect of simulation training on technical ability and skills retention for cricothyrotomy, randomizing anesthesiology residents into 3, 6, or 12 months for posttraining assessments.^11^ Results showed that the training session helped participants retain this skill for at least a year afterward.^11^ At some point, a practicum exam assessing the ultrasound technical skills might also be considered in addition to the knowledge test. However, according to Tomasi, Stark, and Scheiermann, a POCUS curriculum may increase trainees’ knowledge, but it does not necessarily translate to mastery of the practical application.^12^ They suggested that the only way to improve psychomotor skills necessary for POCUS is through clinical practice.^12^ Keeping a case log and assessing skills more frequently could be another way to track progress and improve retention in our program.

Finally, it is not known if improvements in POCUS knowledge translate to superior clinical outcomes. In the future, as POCUS training and technology become more accessible in our institution, additional outcome measures such as unexpected intensive care unit admissions or delayed diagnosis and/or treatment may be evaluated.

## Appendices


Ultrasound Basics.pptxLung Ultrasound.pptxCardiac Ultrasound.pptxVascular Access Ultrasound.pptxAirway Ultrasound.pptxAbdominal Ultrasound.pptxNeuraxial Ultrasound.pptxChecklist for POCUS Scanning.docxPOCUS CA1 Curriculum Pretest.pptxPOCUS CA1 Curriculum Posttest.pptxPOCUS Survey.docx
*All appendices are peer reviewed as integral parts of the Original Publication.*
