# Supplementary material for: An Introductory Point-of-Care Ultrasound Curriculum for an Anesthesiology Residency Program
Source: MedEdPORTAL. 2022 Dec 23;18:11291. doi: 10.15766/mep_2374-8265.11291 (PMC9780414; doi:10.15766/mep_2374-8265.11291)
Supplement: Supplementary file 1 — Ultrasound Basics.pptxLung Ultrasound.pptxCardiac Ultrasound.pptxVascular Access Ultrasound.pptxAirway Ultrasound.pptxAbdominal Ultrasound.pptxNeuraxial Ultrasound.pptxChecklist for POCUS Scanning.docxPOCUS CA1 Curriculum Pretest.pptxPOCUS CA1 Curriculum Posttest.pptxPOCUS Survey.docx [file mep_2374-8265.11291-s001.zip › A. Ultrasound Basics.pptx]

## Slide 1
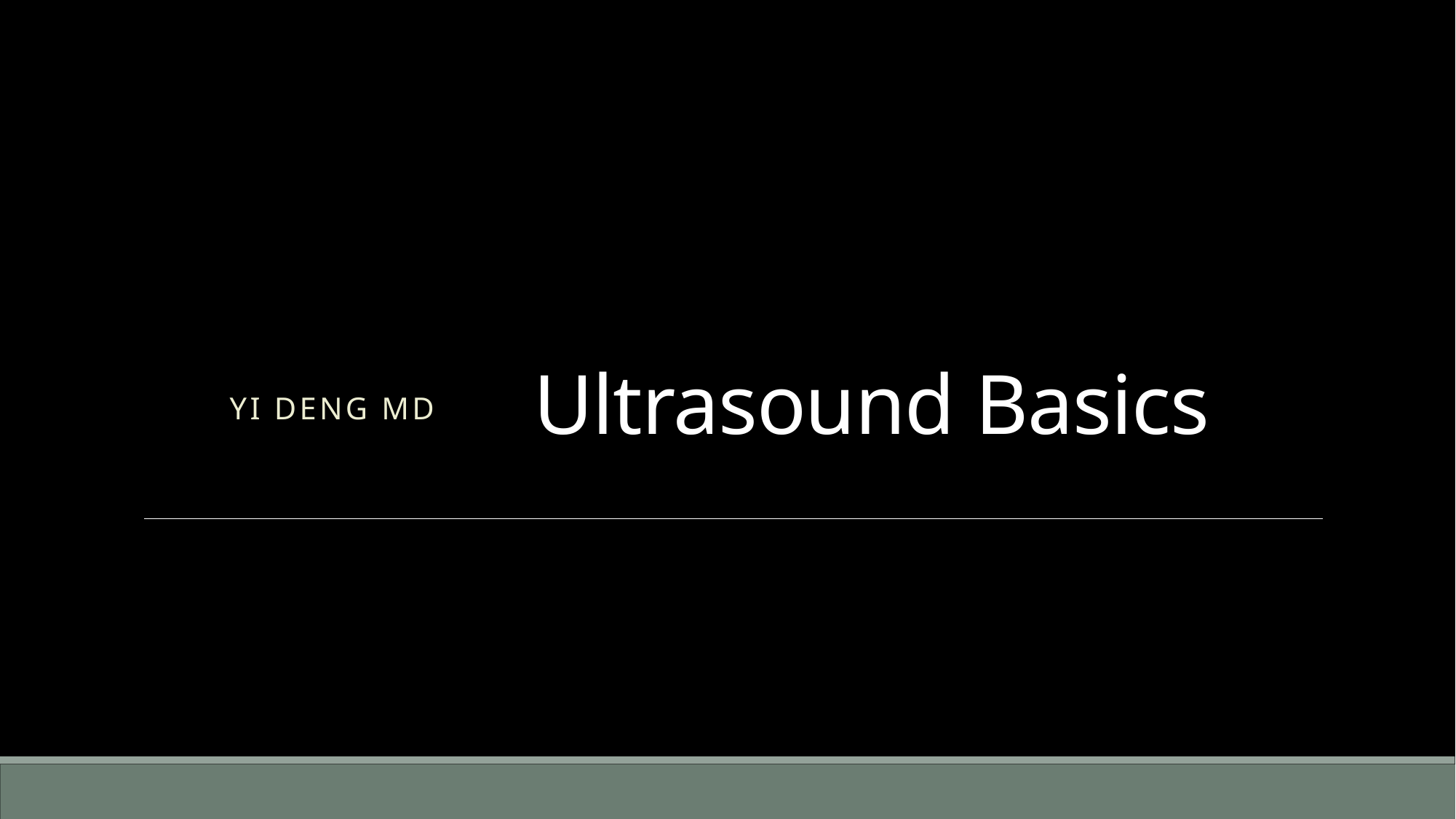

Yi Deng MD
# Ultrasound Basics

## Slide 2
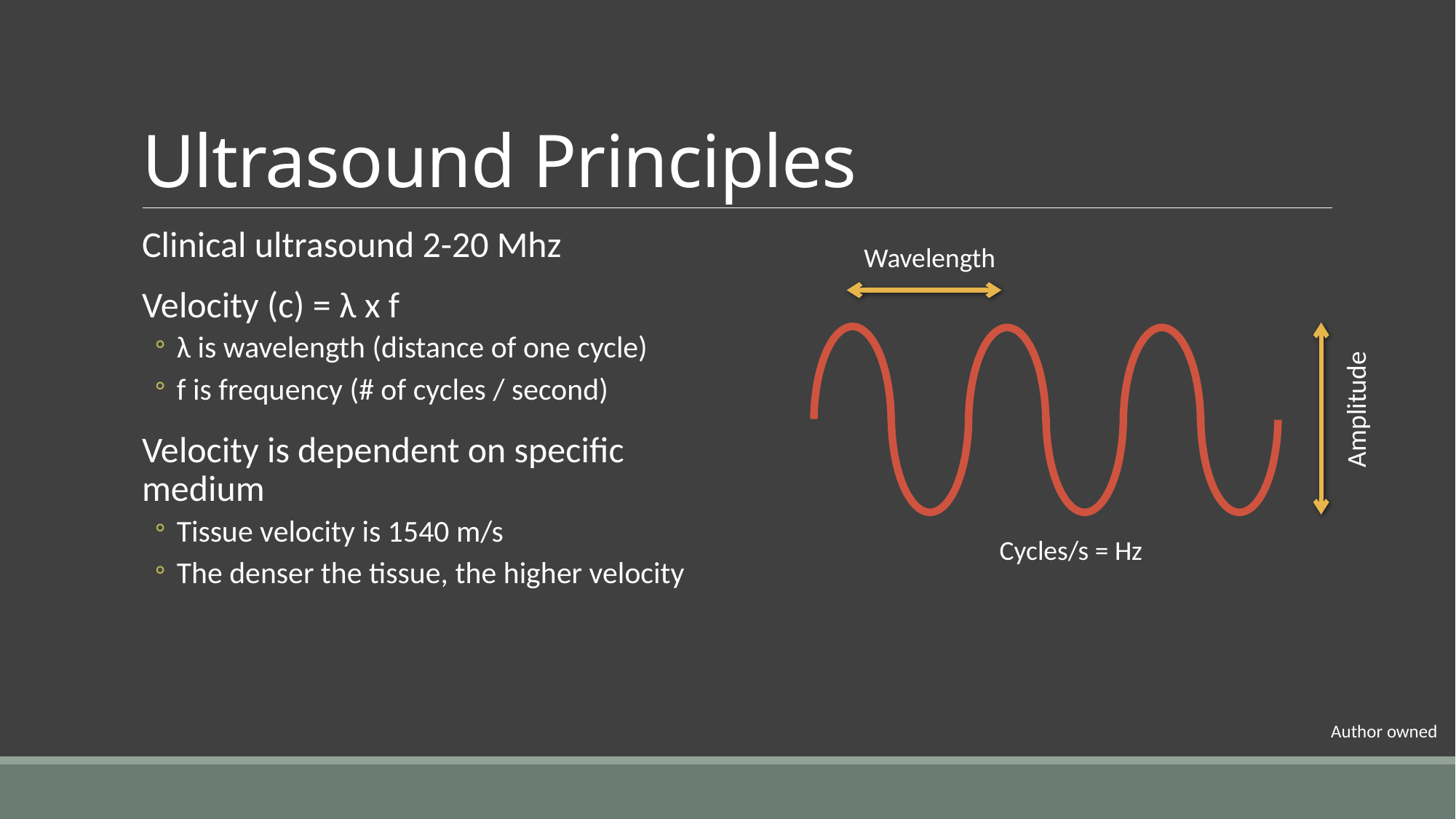

# Ultrasound Principles
Clinical ultrasound 2-20 Mhz
Velocity (c) = λ x f
λ is wavelength (distance of one cycle)
f is frequency (# of cycles / second)
Velocity is dependent on specific medium
Tissue velocity is 1540 m/s
The denser the tissue, the higher velocity
Wavelength
Amplitude
Cycles/s = Hz
Author owned

## Slide 3
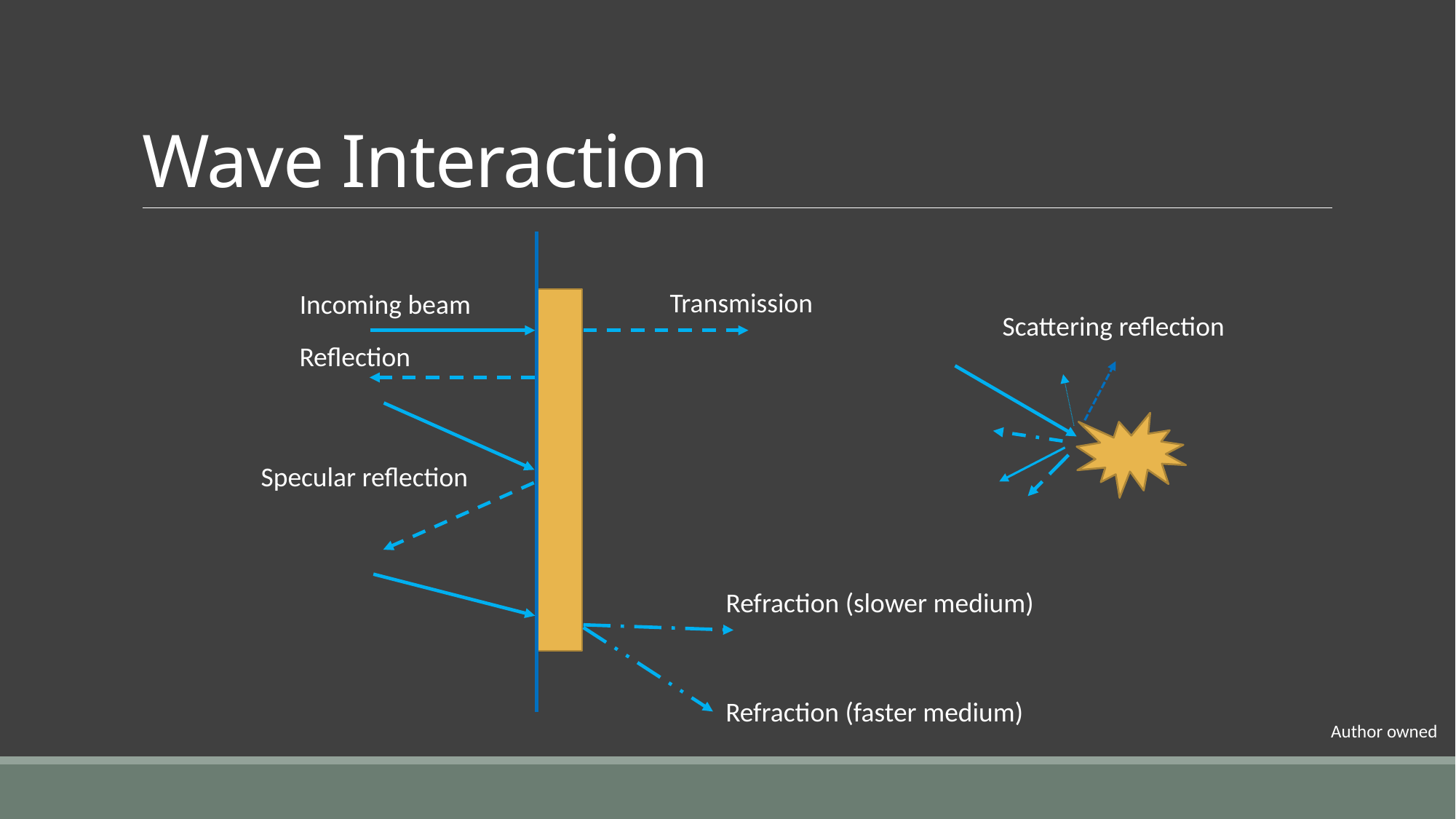

# Wave Interaction
Transmission
Incoming beam
Scattering reflection
Reflection
Specular reflection
Refraction (slower medium)
Refraction (faster medium)
Author owned

## Slide 4
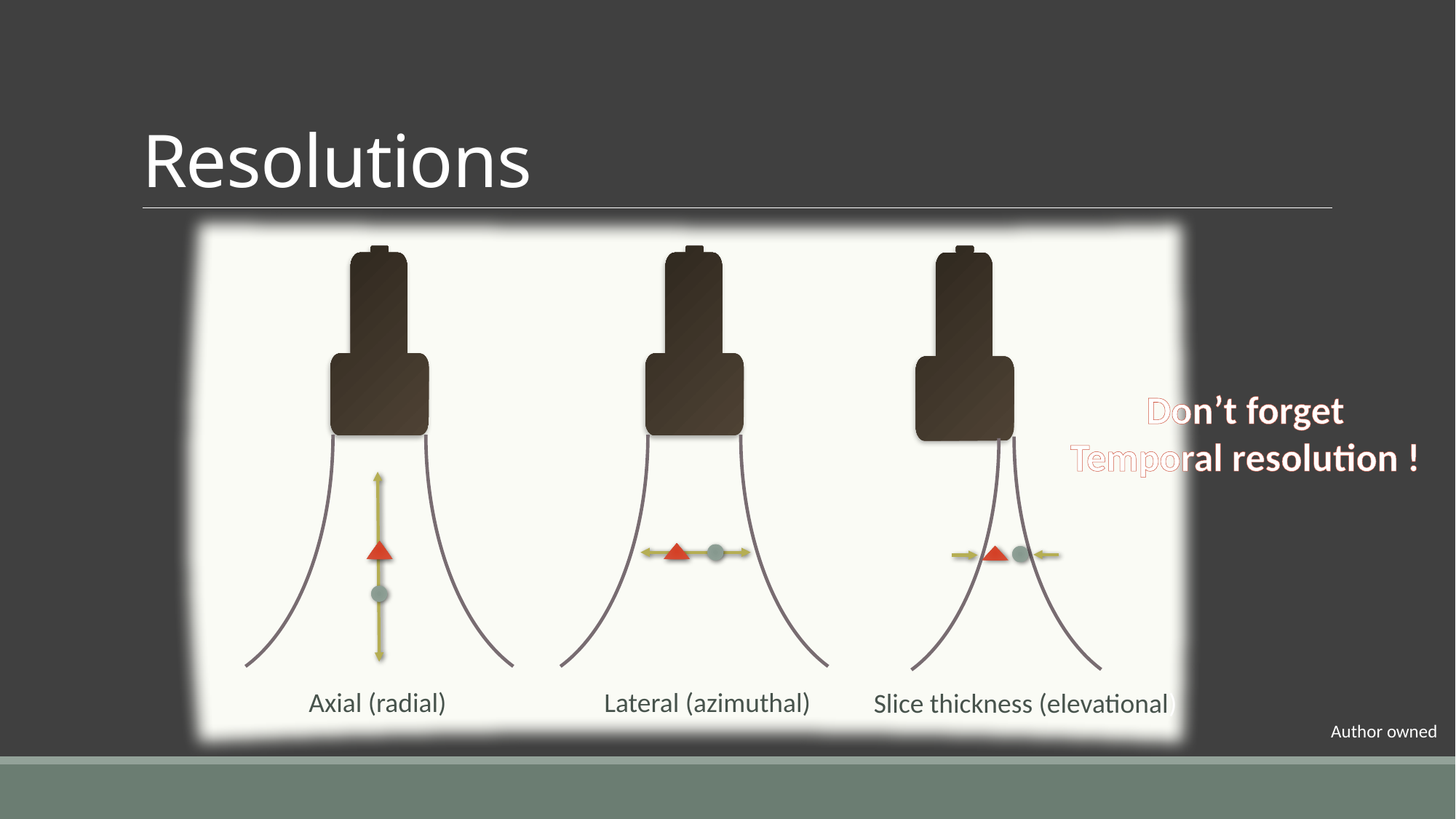

# Resolutions
Axial (radial)
Lateral (azimuthal)
Slice thickness (elevational)
Don’t forget Temporal resolution !
Author owned

## Slide 5
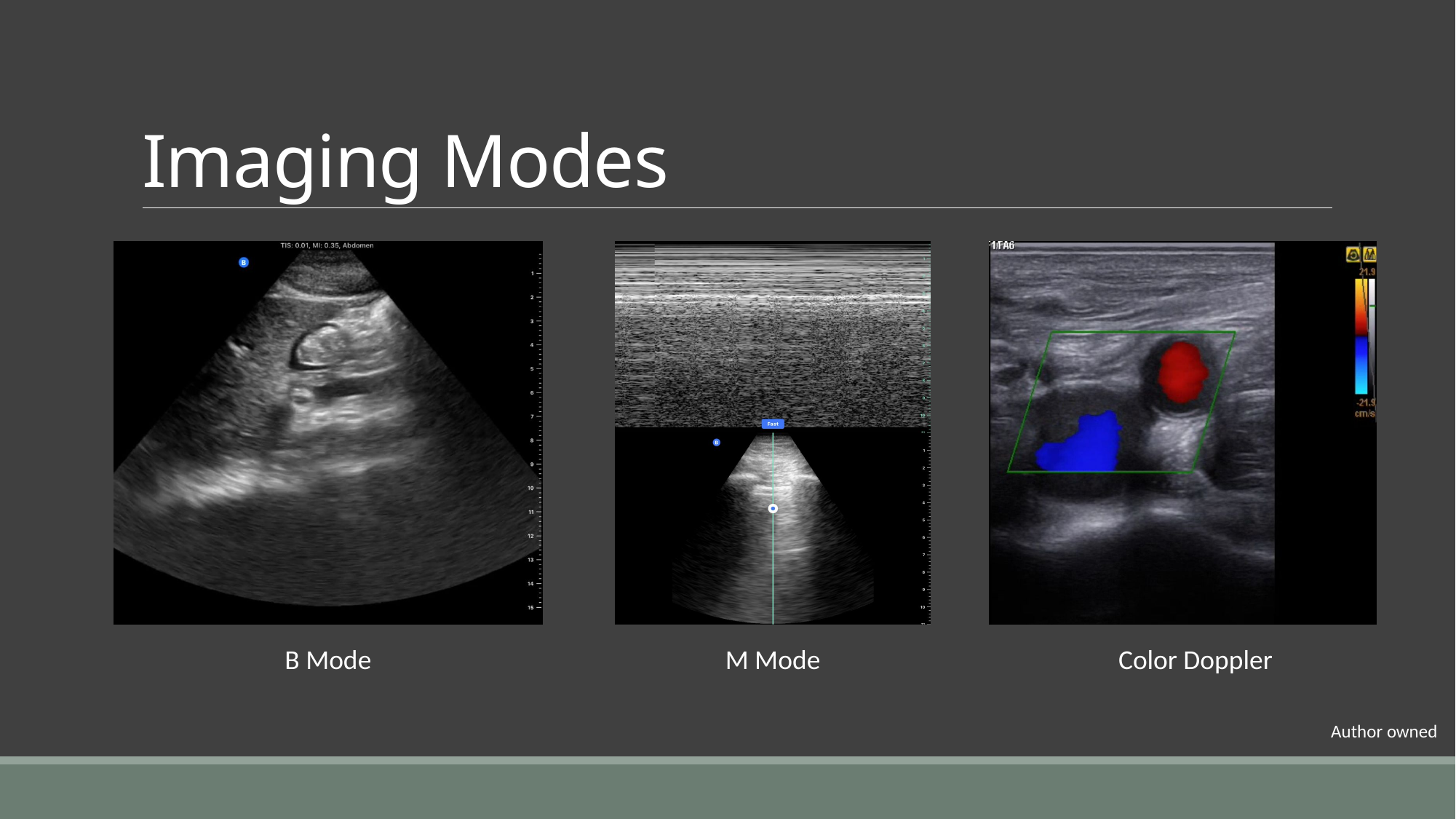

# Imaging Modes
B Mode
M Mode
Color Doppler
Author owned

## Slide 6
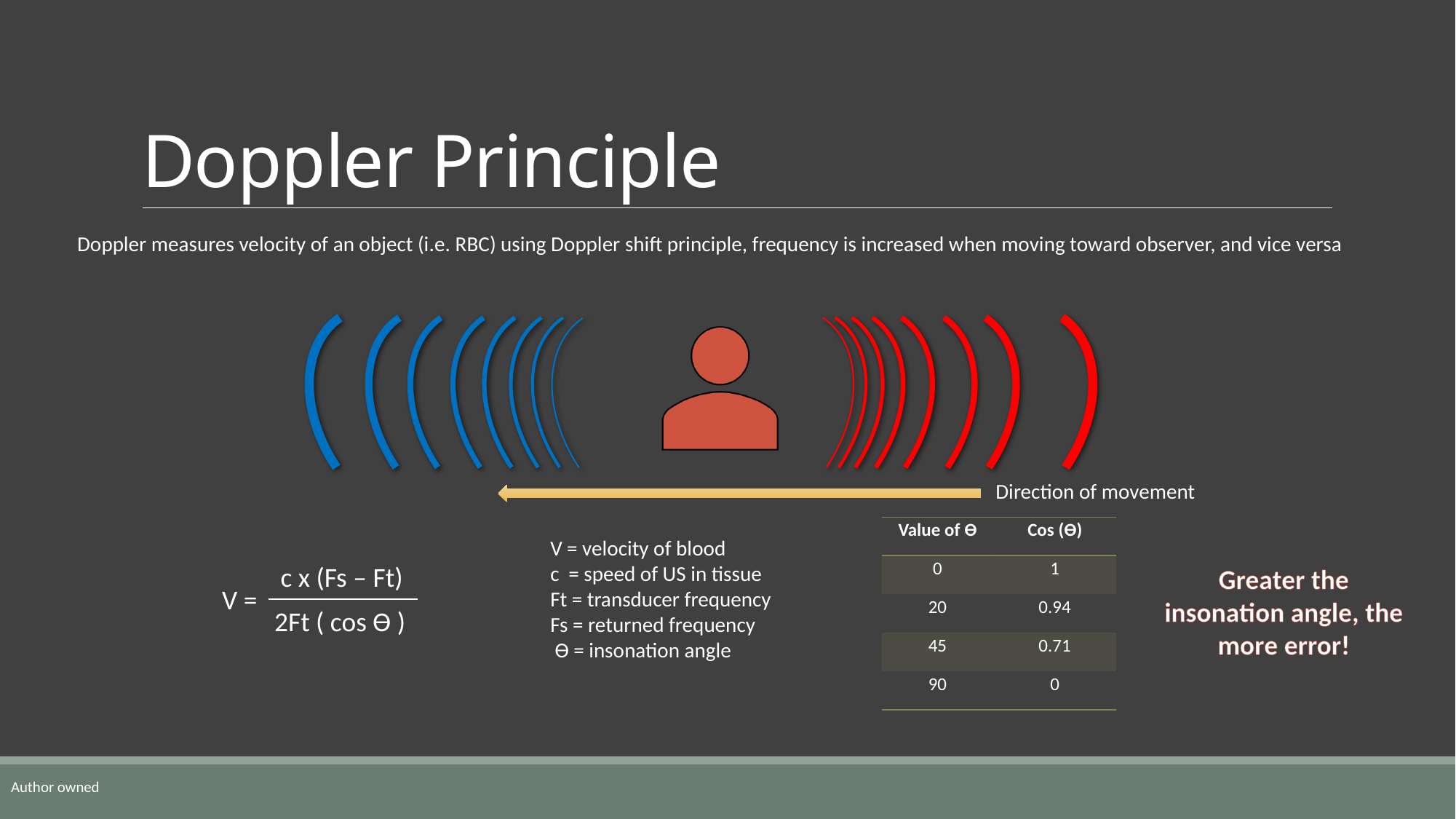

# Doppler Principle
Doppler measures velocity of an object (i.e. RBC) using Doppler shift principle, frequency is increased when moving toward observer, and vice versa
Direction of movement
| Value of ϴ | Cos (ϴ) |
| --- | --- |
| 0 | 1 |
| 20 | 0.94 |
| 45 | 0.71 |
| 90 | 0 |
V = velocity of blood
c = speed of US in tissue
Ft = transducer frequency
Fs = returned frequency
 ϴ = insonation angle
c x (Fs – Ft)
V =
2Ft ( cos ϴ )
Greater the insonation angle, the more error!
Author owned

## Slide 7
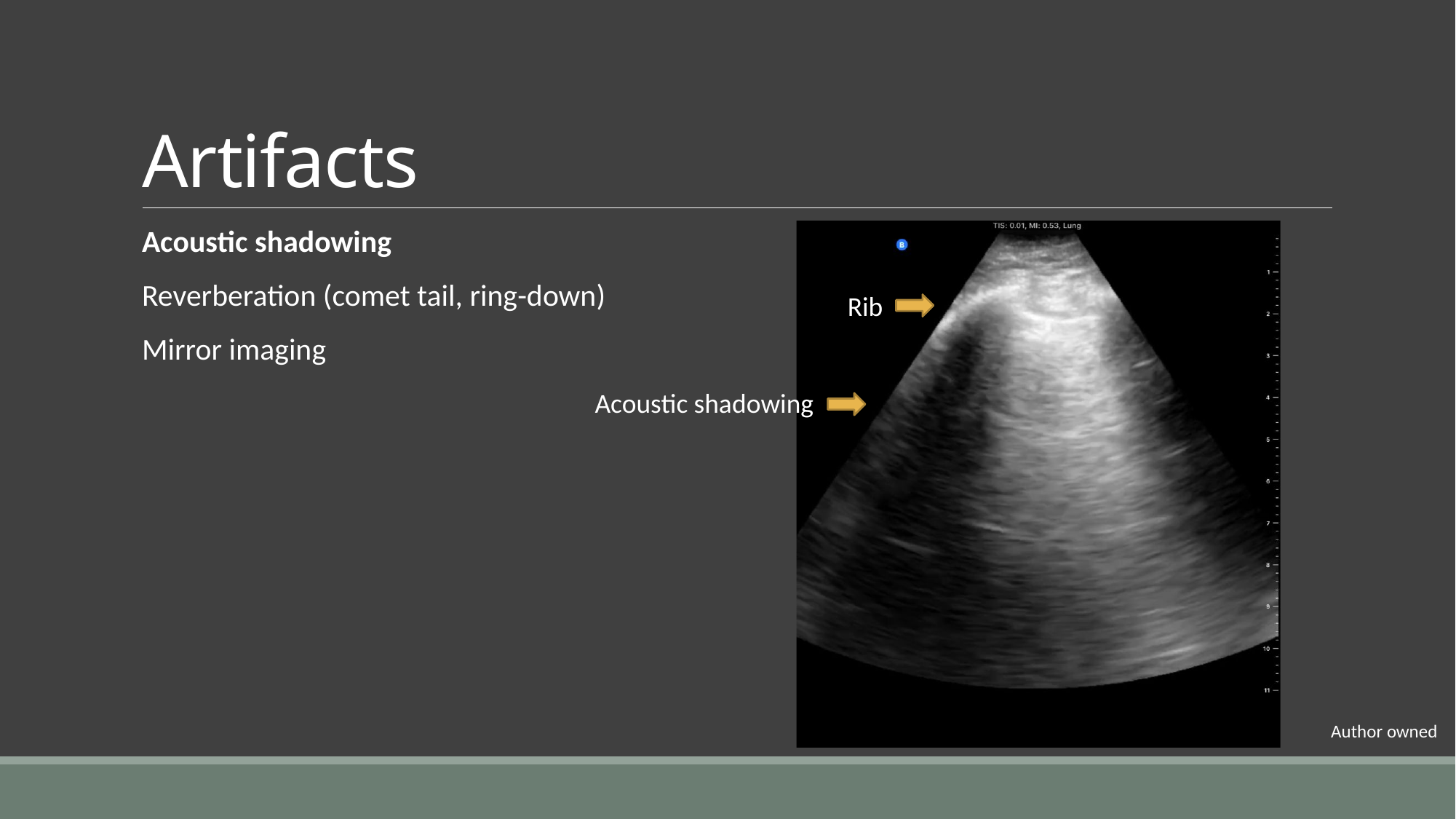

# Artifacts
Acoustic shadowing
Reverberation (comet tail, ring-down)
Mirror imaging
Rib
Acoustic shadowing
Author owned

## Slide 8
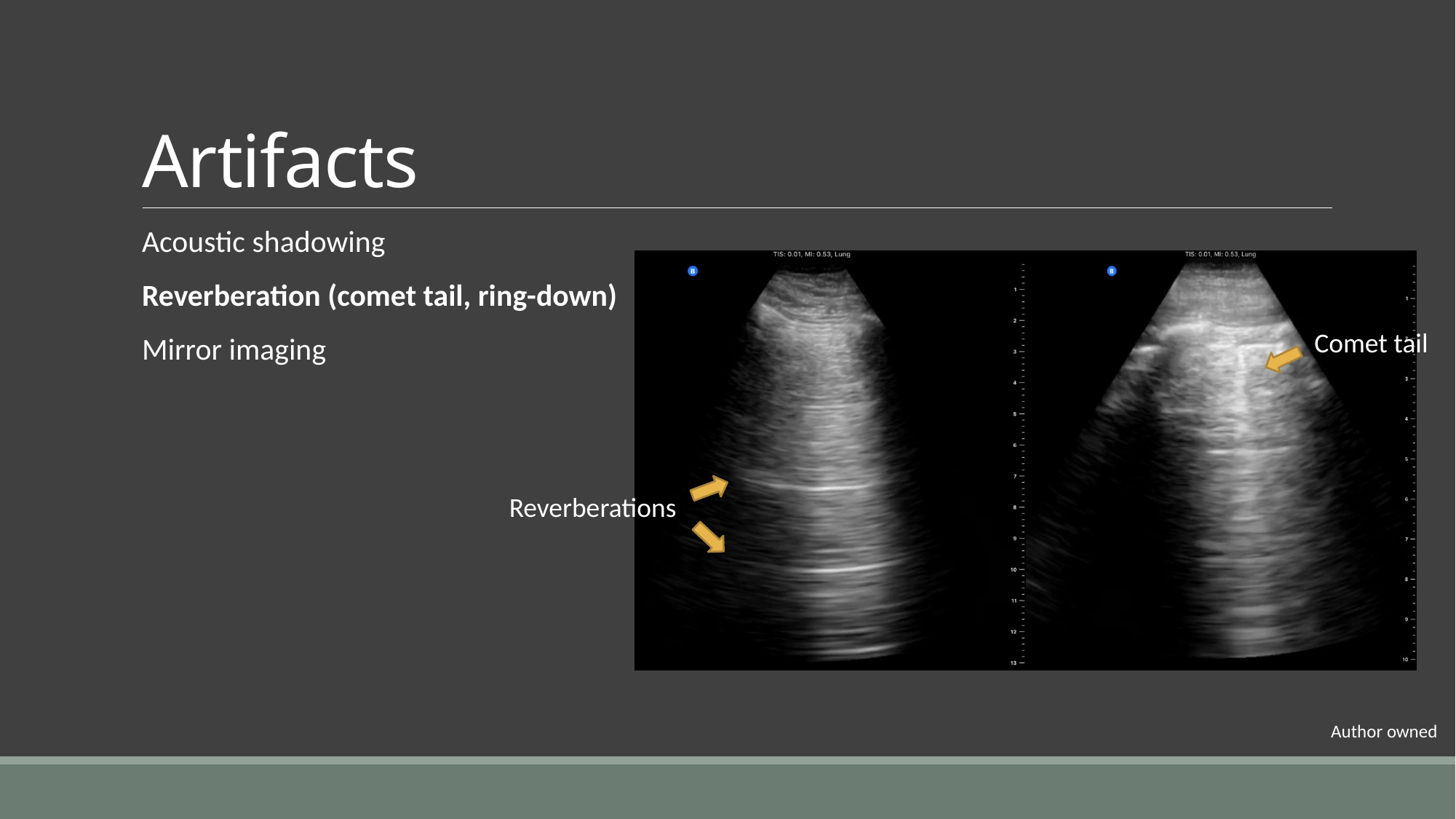

# Artifacts
Acoustic shadowing
Reverberation (comet tail, ring-down)
Mirror imaging
Comet tail
Reverberations
Author owned

## Slide 9
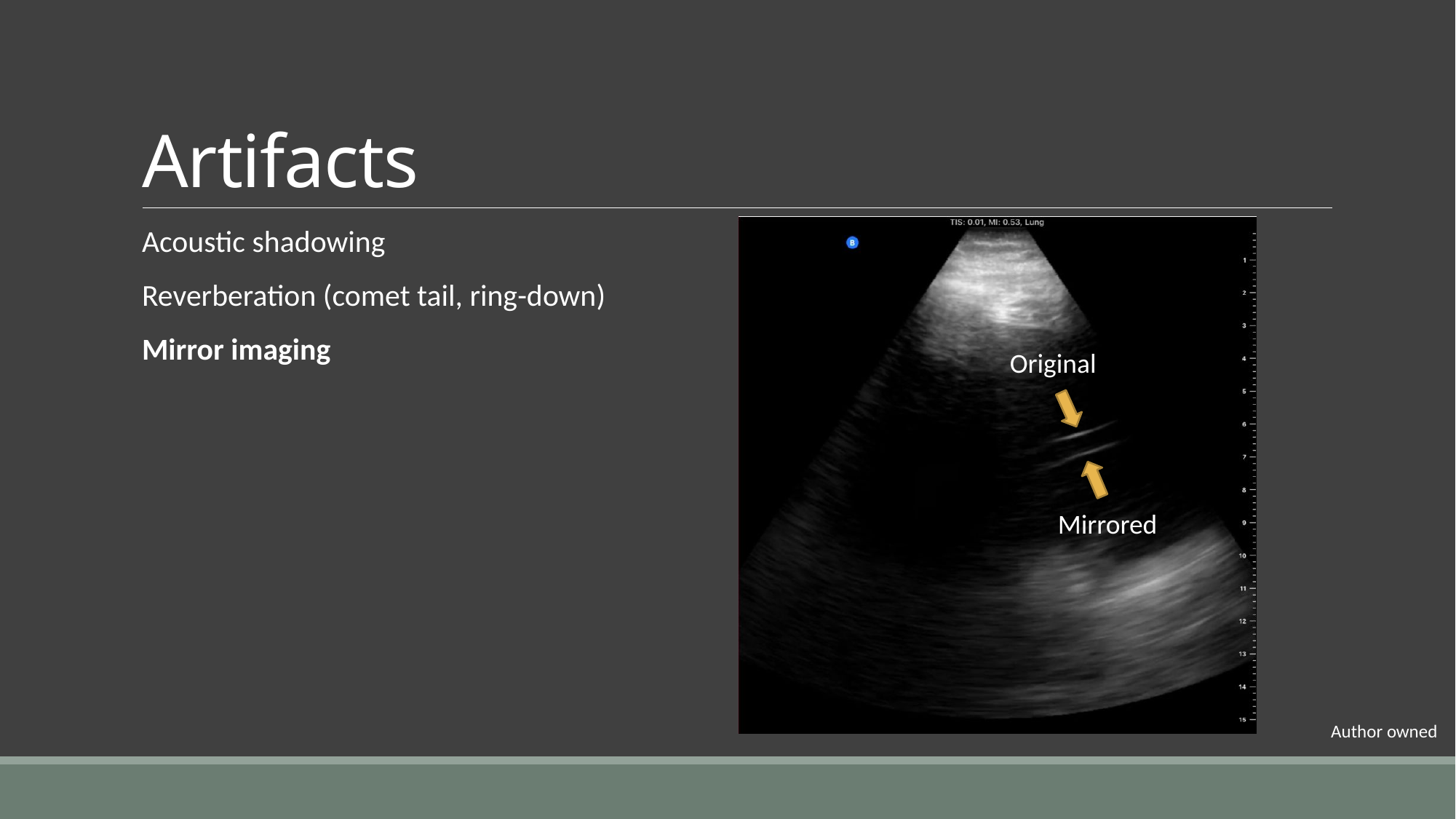

# Artifacts
Original
Mirrored
Acoustic shadowing
Reverberation (comet tail, ring-down)
Mirror imaging
Author owned

## Slide 10
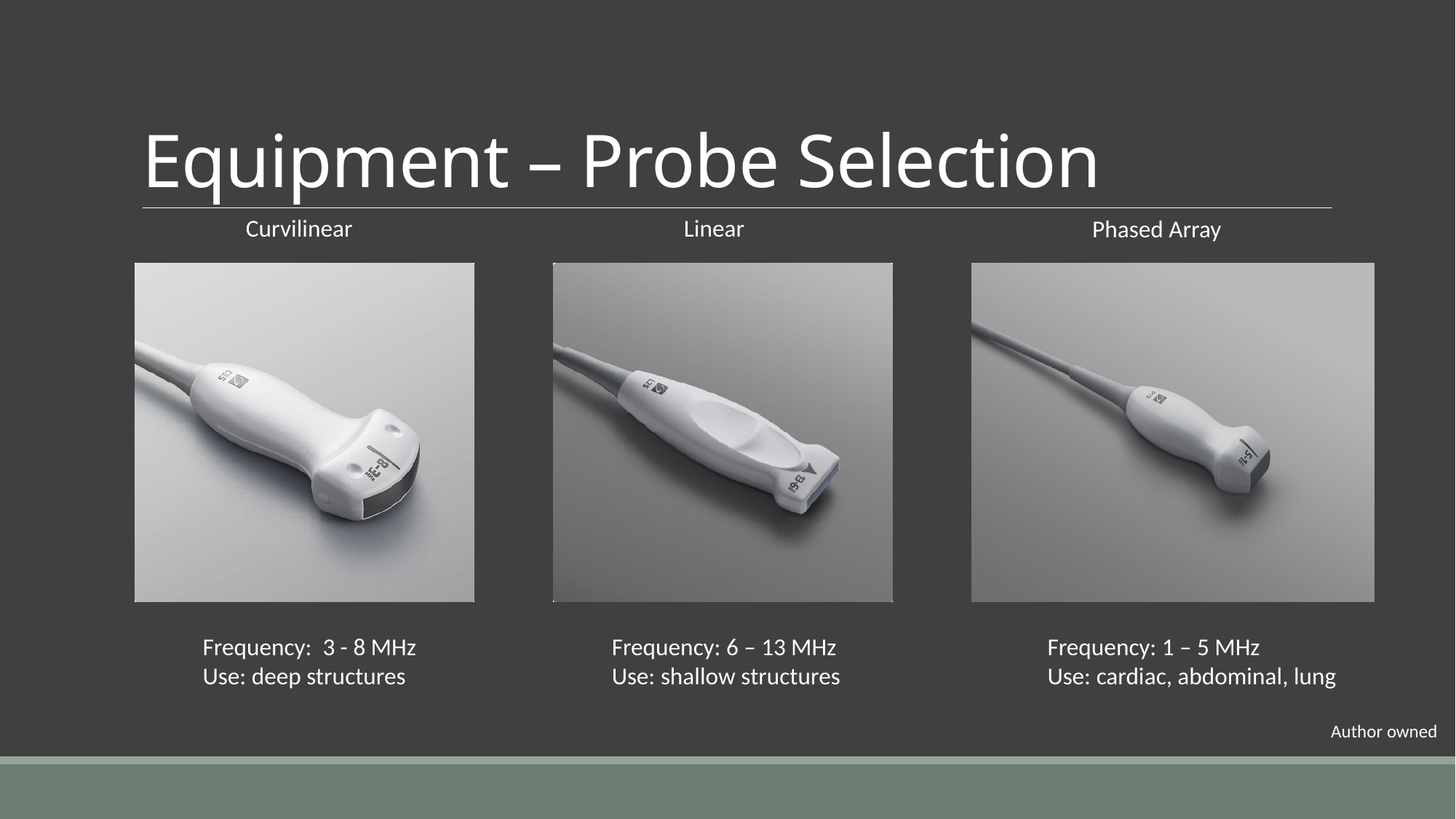

# Equipment – Probe Selection
Curvilinear
Linear
Phased Array
Frequency: 6 – 13 MHz
Use: shallow structures
Frequency: 1 – 5 MHz
Use: cardiac, abdominal, lung
Frequency: 3 - 8 MHz
Use: deep structures
Author owned

## Slide 11
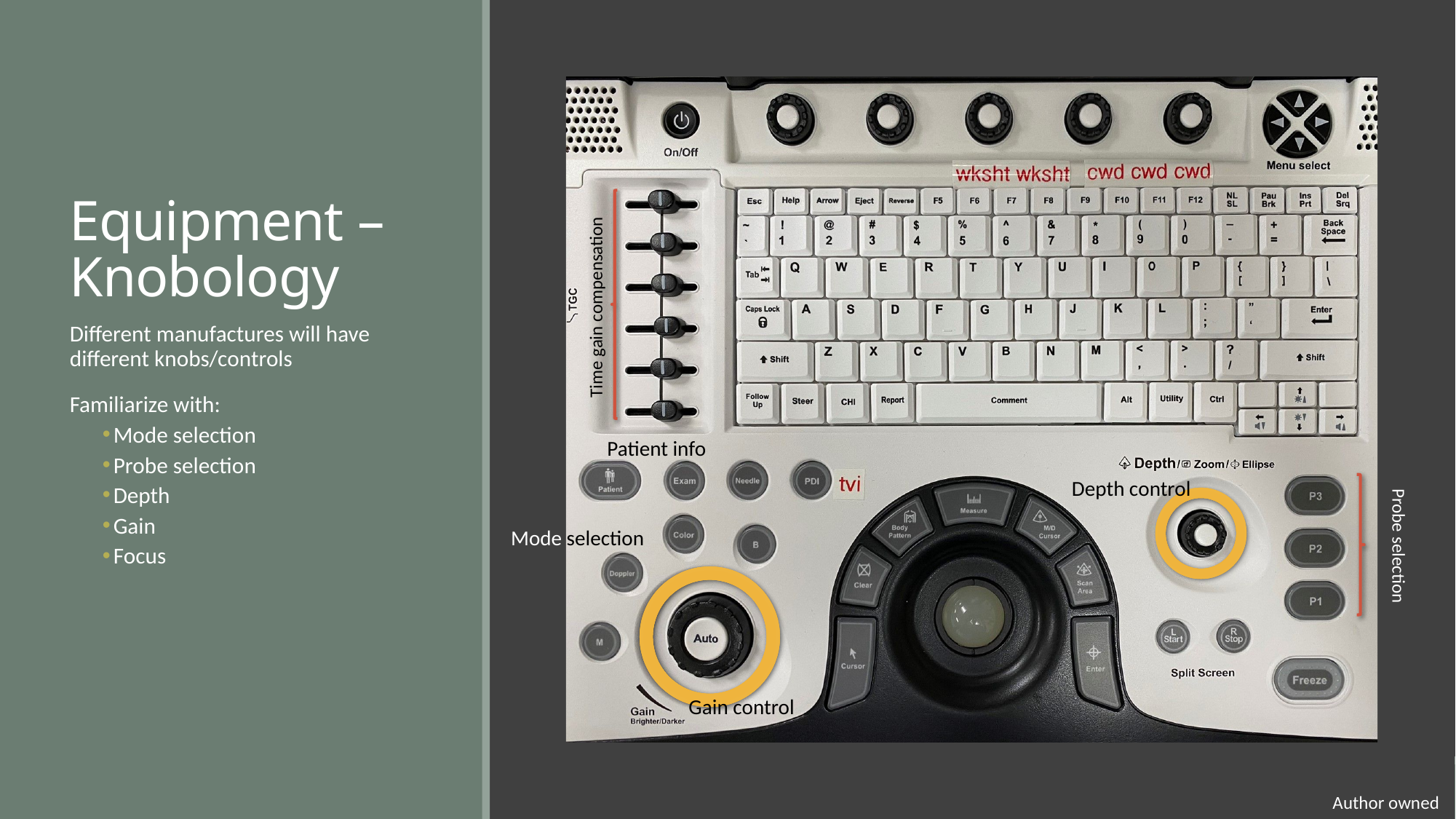

# Equipment – Knobology
Time gain compensation
Different manufactures will have different knobs/controls
Familiarize with:
Mode selection
Probe selection
Depth
Gain
Focus
Patient info
Depth control
Mode selection
Probe selection
Gain control
Author owned

## Slide 12
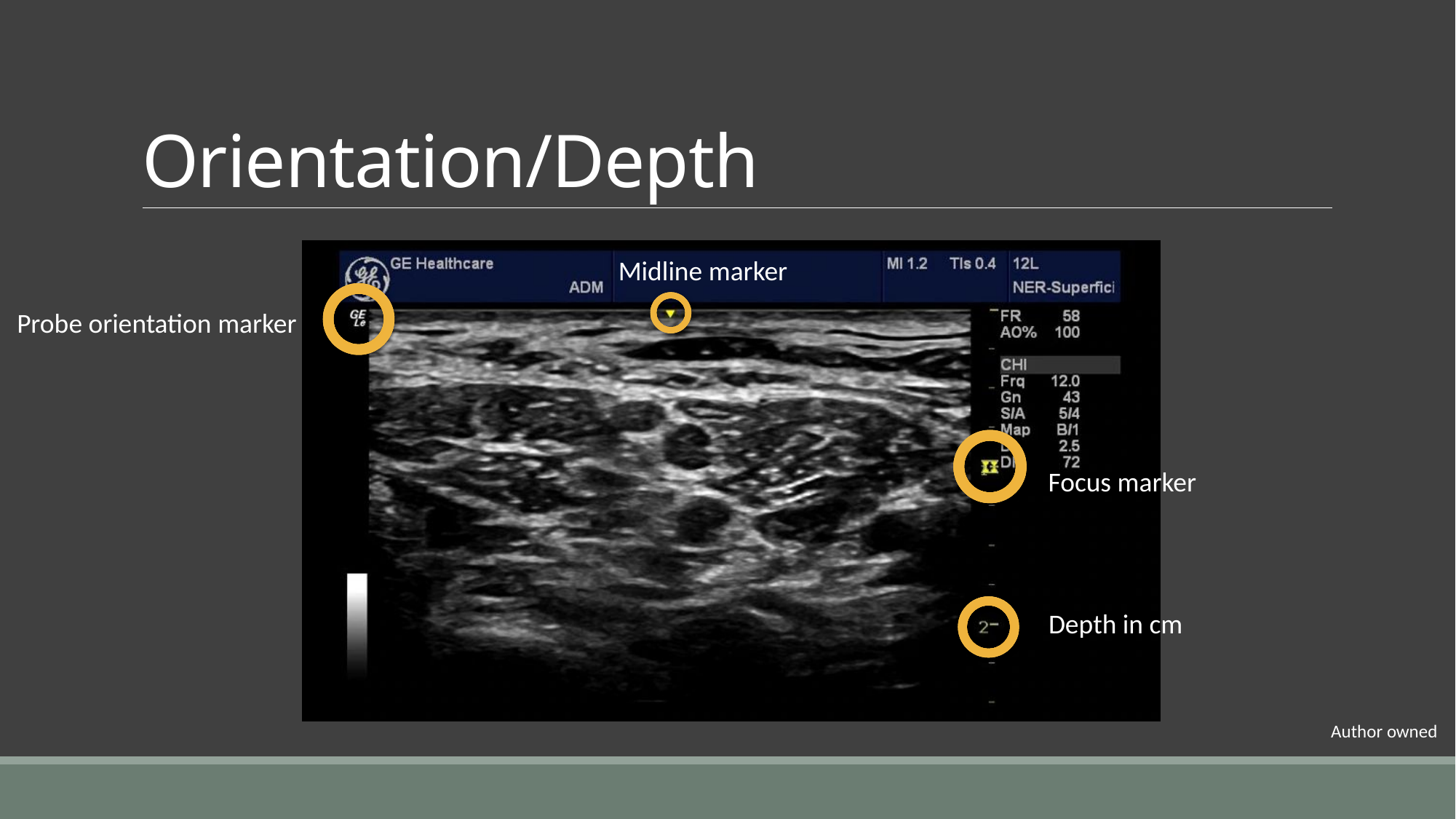

# Orientation/Depth
Midline marker
Probe orientation marker
Focus marker
Depth in cm
Author owned

## Slide 13
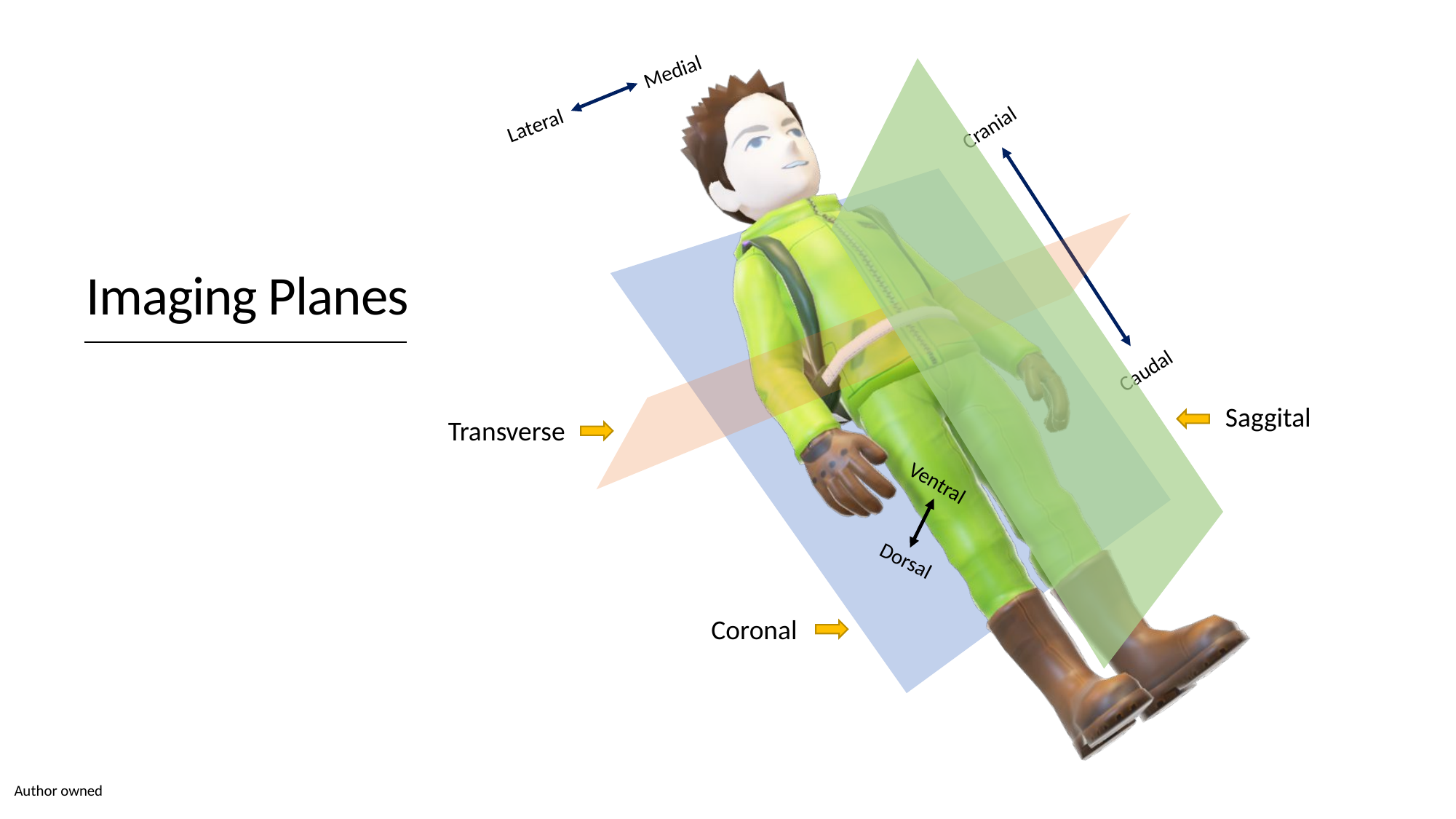

Medial
Cranial
Lateral
Caudal
Saggital
Transverse
Ventral
Dorsal
Coronal
Imaging Planes
Author owned

## Slide 14
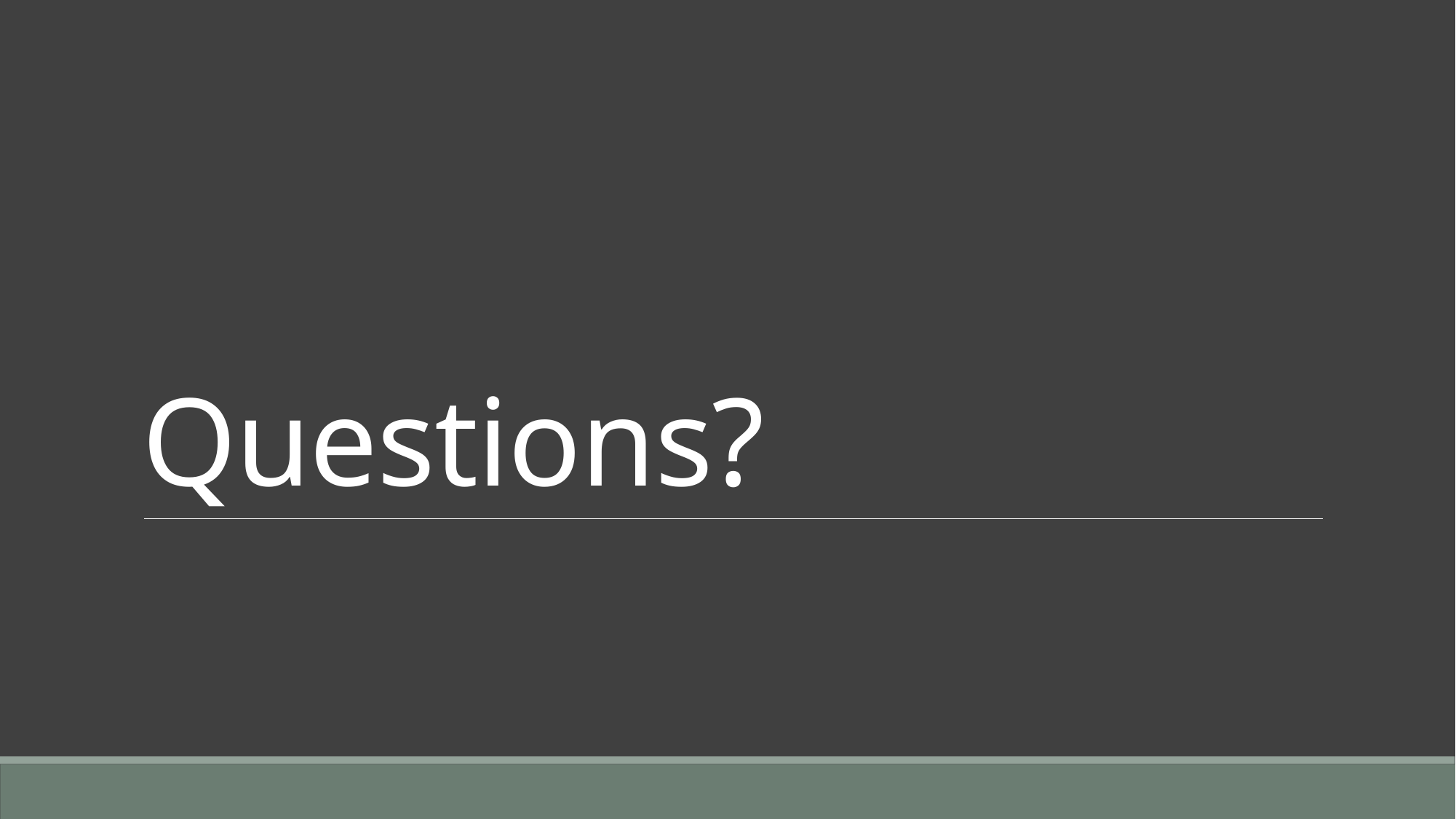

# Questions?
