# Supplementary material for: An Introductory Point-of-Care Ultrasound Curriculum for an Anesthesiology Residency Program
Source: MedEdPORTAL. 2022 Dec 23;18:11291. doi: 10.15766/mep_2374-8265.11291 (PMC9780414; doi:10.15766/mep_2374-8265.11291)
Supplement: Supplementary file 1 — Ultrasound Basics.pptxLung Ultrasound.pptxCardiac Ultrasound.pptxVascular Access Ultrasound.pptxAirway Ultrasound.pptxAbdominal Ultrasound.pptxNeuraxial Ultrasound.pptxChecklist for POCUS Scanning.docxPOCUS CA1 Curriculum Pretest.pptxPOCUS CA1 Curriculum Posttest.pptxPOCUS Survey.docx [file mep_2374-8265.11291-s001.zip › B. Lung Ultrasound.pptx]

## Slide 1
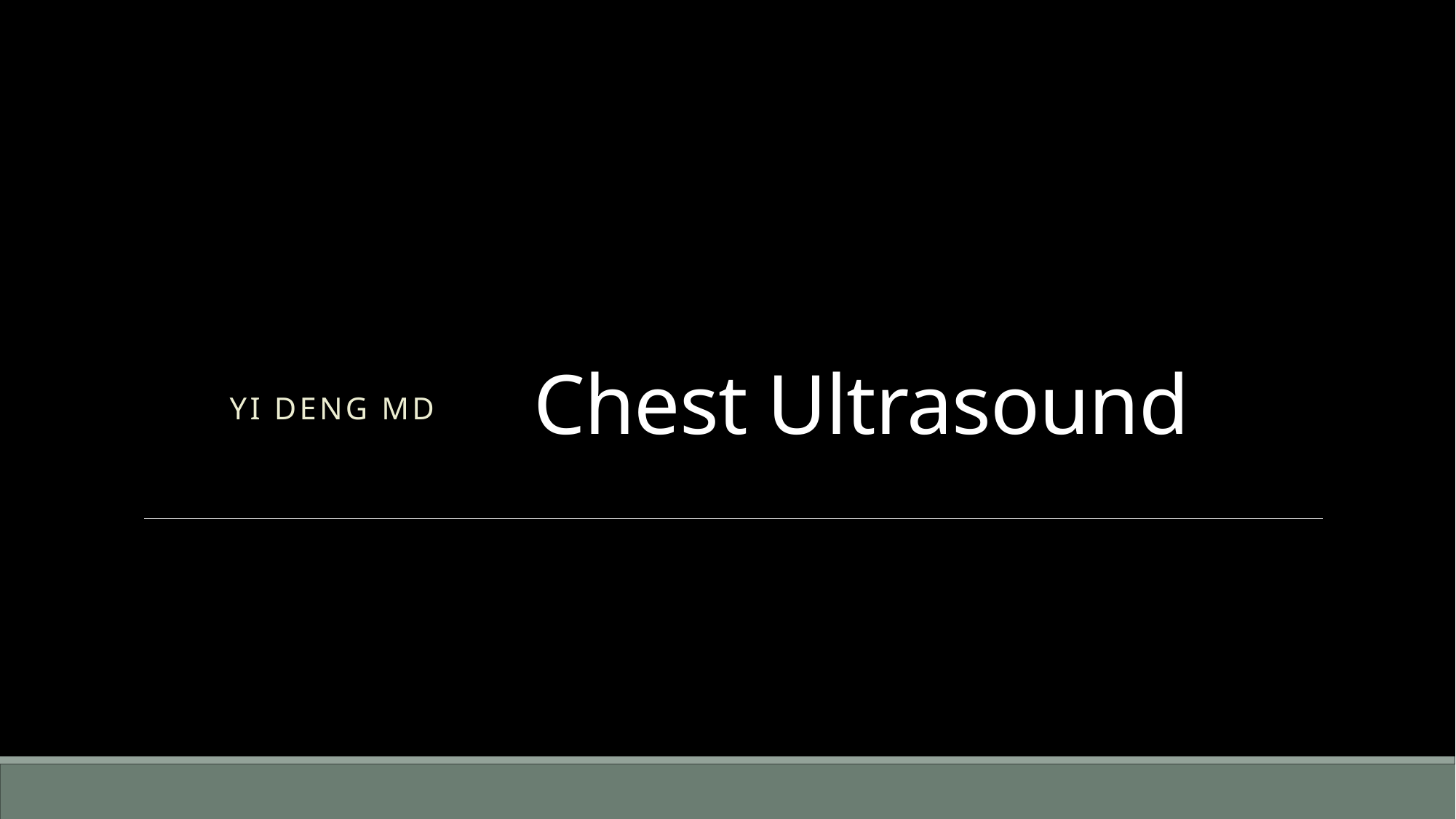

Yi Deng MD
# Chest Ultrasound

## Slide 2
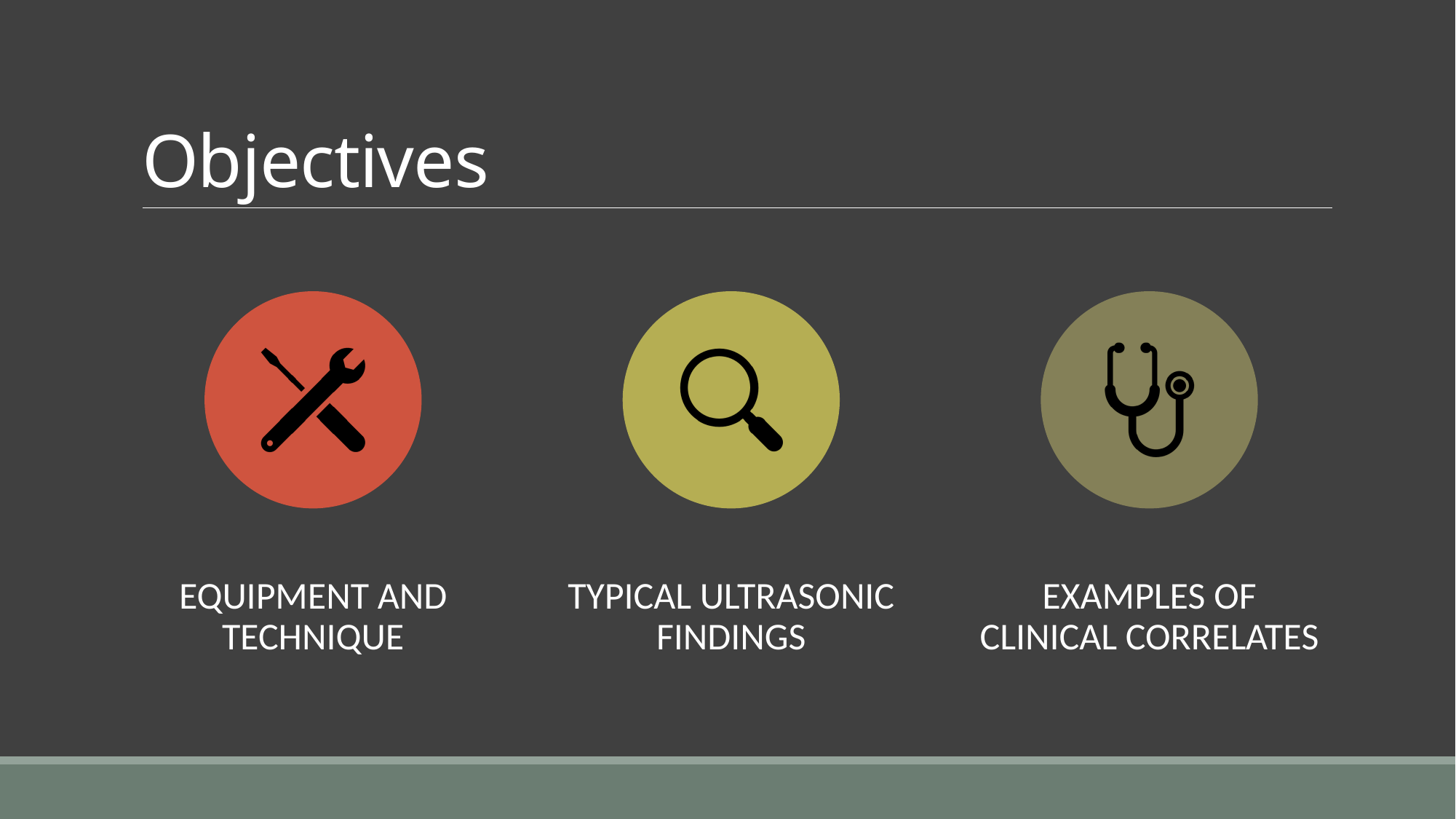

# Objectives

## Slide 3
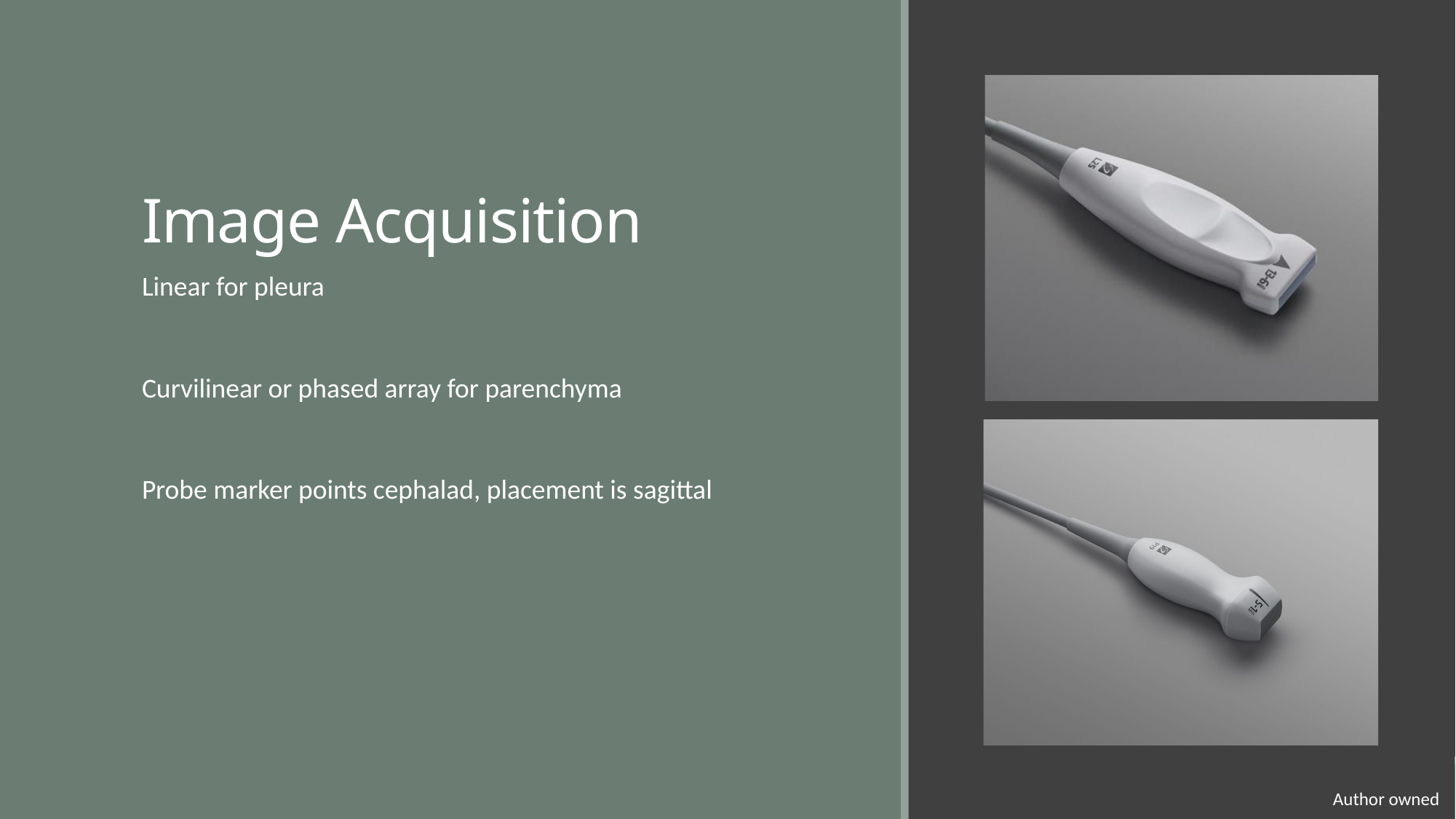

# Image Acquisition
Linear for pleura
Curvilinear or phased array for parenchyma
Probe marker points cephalad, placement is sagittal
Author owned

## Slide 4
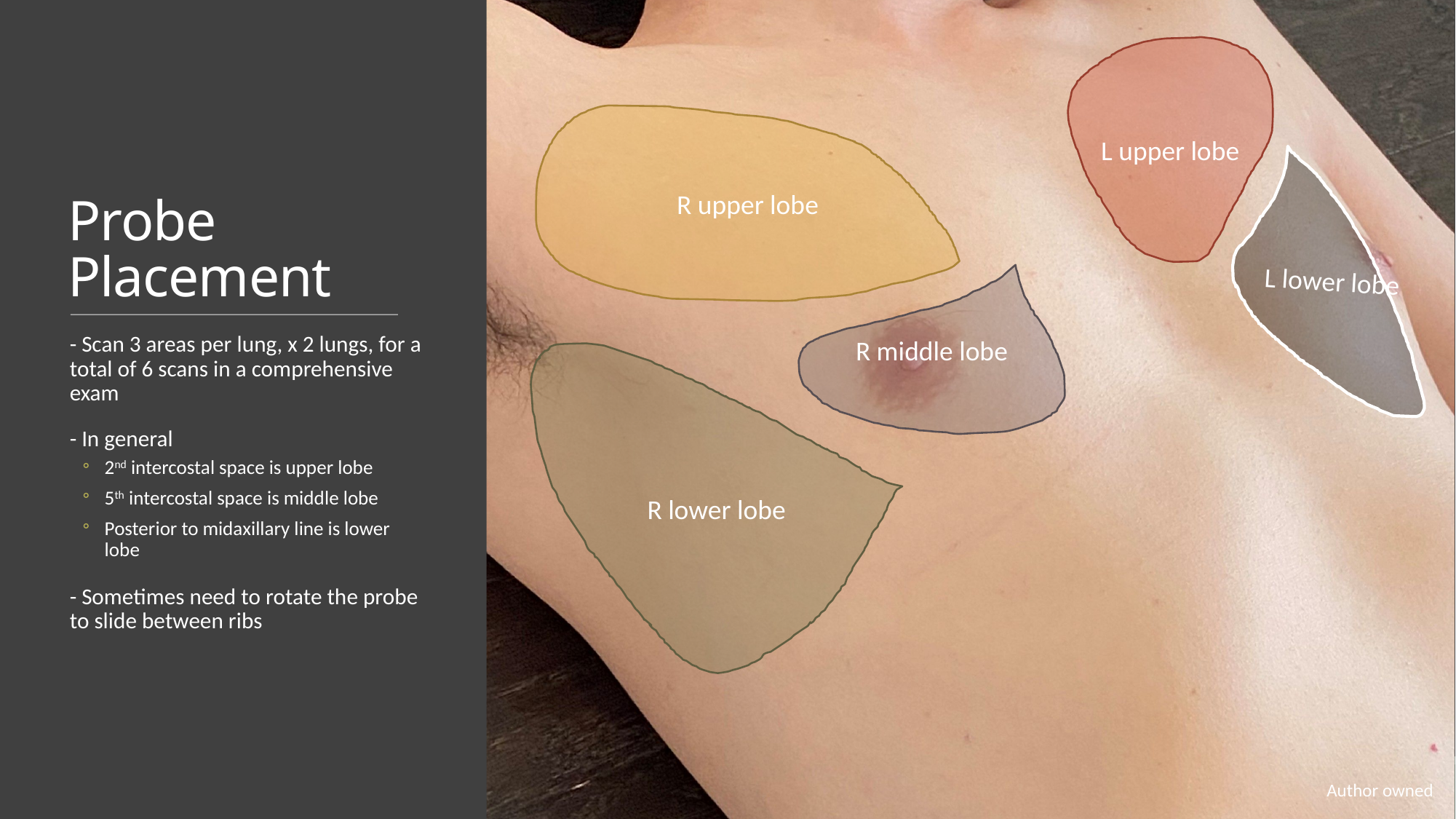

L upper lobe
# Probe Placement
R upper lobe
L lower lobe
R middle lobe
- Scan 3 areas per lung, x 2 lungs, for a total of 6 scans in a comprehensive exam
- In general
2nd intercostal space is upper lobe
5th intercostal space is middle lobe
Posterior to midaxillary line is lower lobe
- Sometimes need to rotate the probe to slide between ribs
R lower lobe
Author owned

## Slide 5
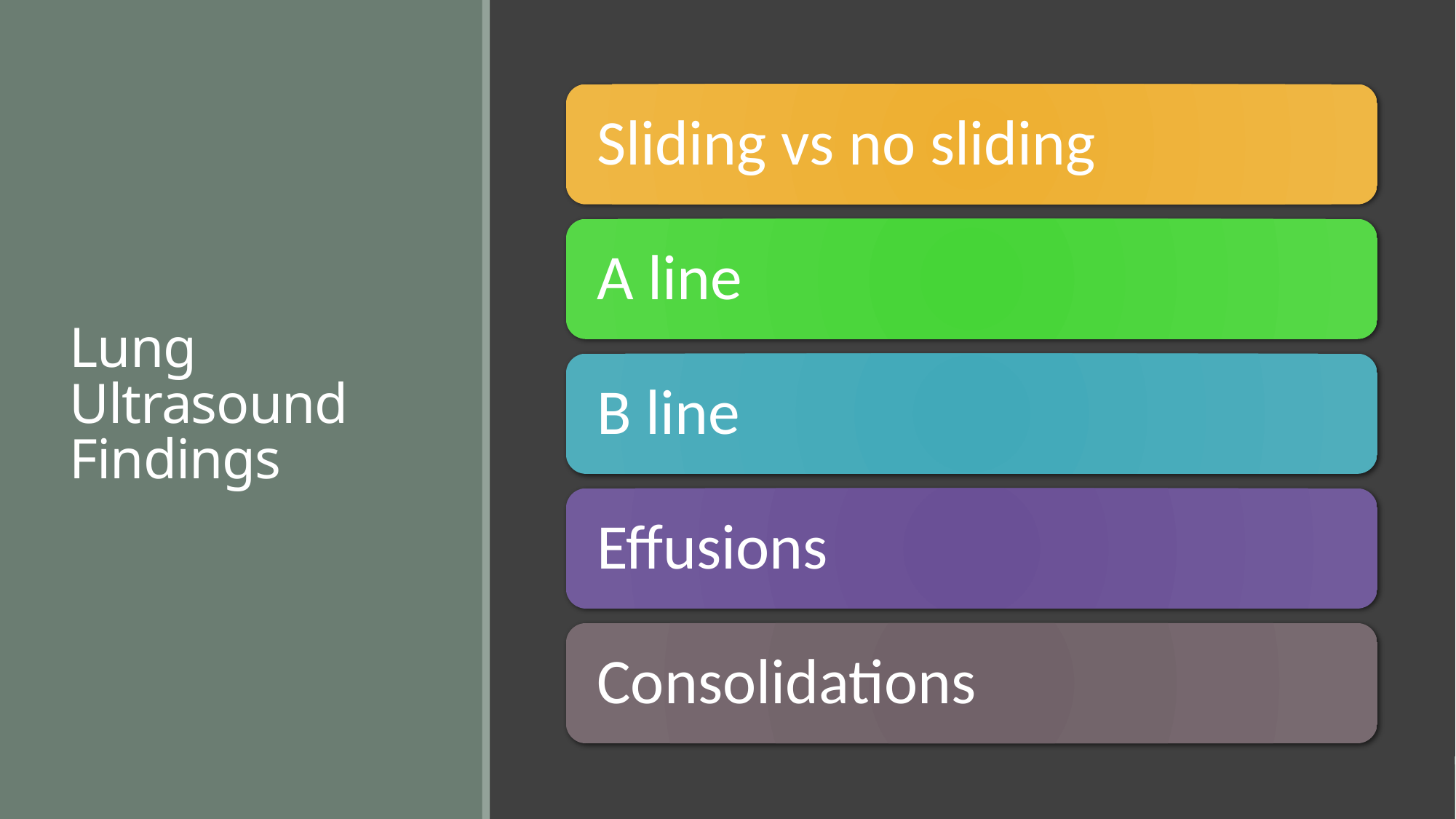

# Lung Ultrasound Findings

## Slide 6
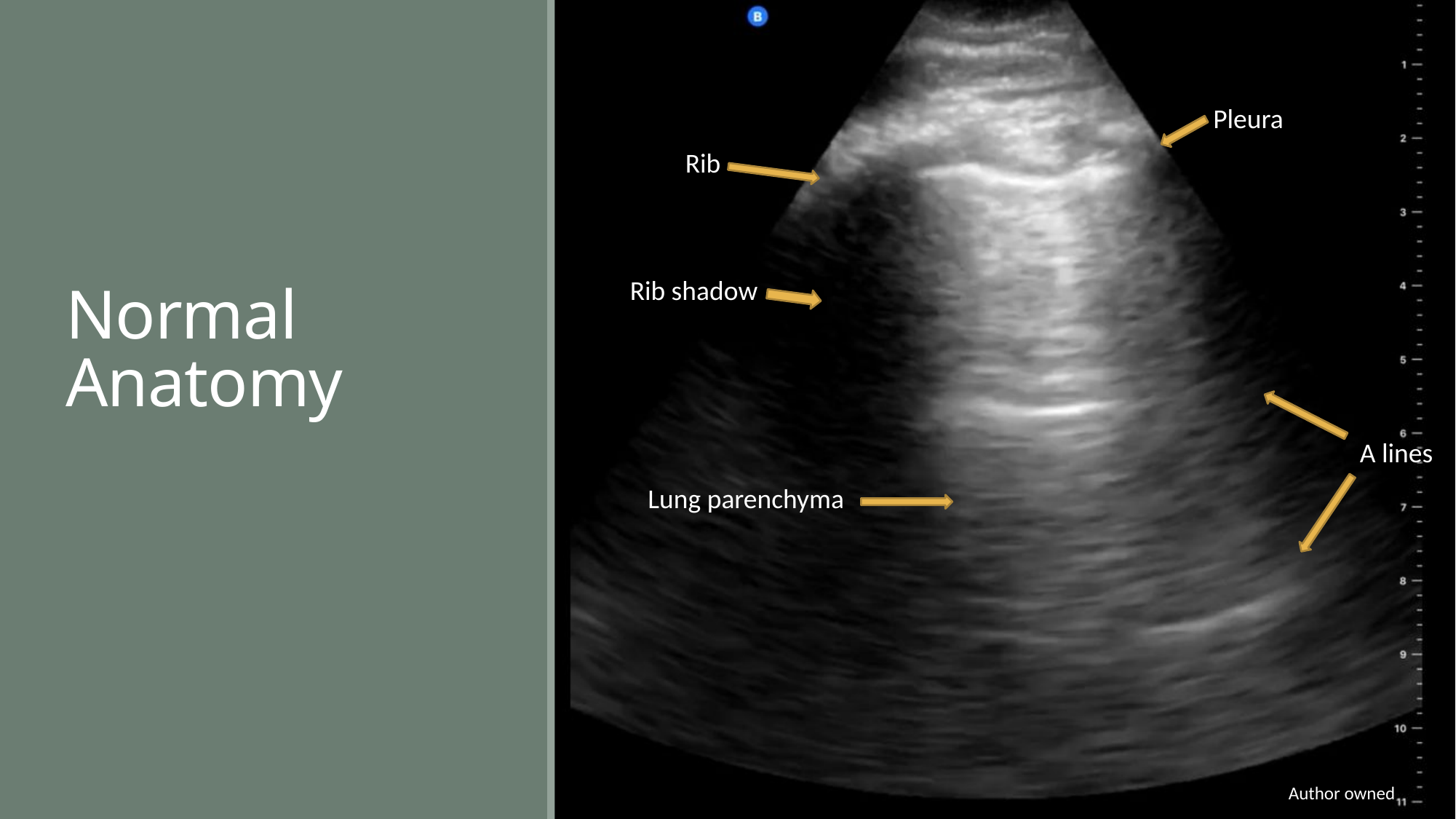

# Normal Anatomy
Pleura
Rib
Rib shadow
A lines
Lung parenchyma
Author owned

## Slide 7
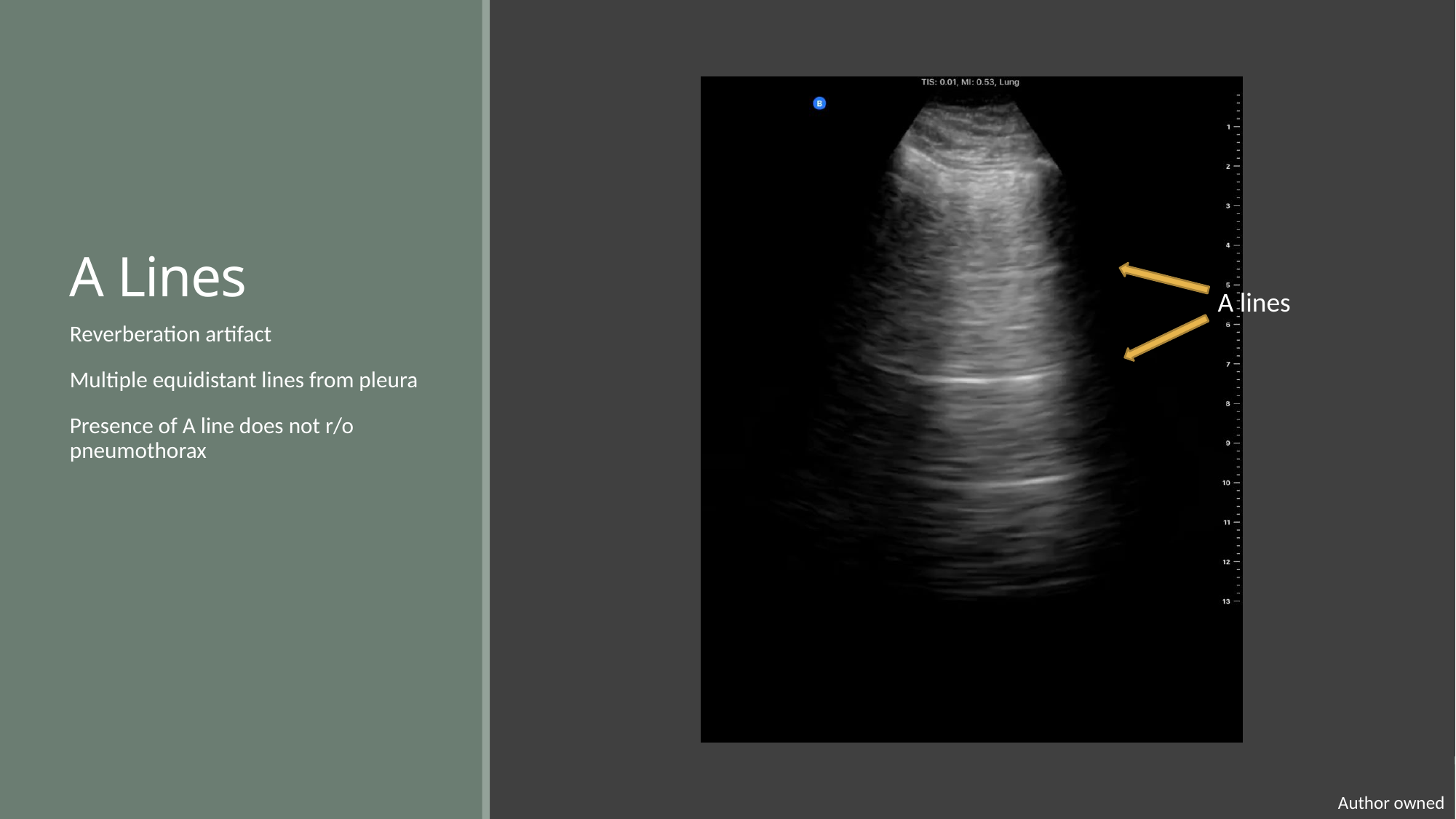

# A Lines
A lines
Reverberation artifact
Multiple equidistant lines from pleura
Presence of A line does not r/o pneumothorax
Author owned

## Slide 8
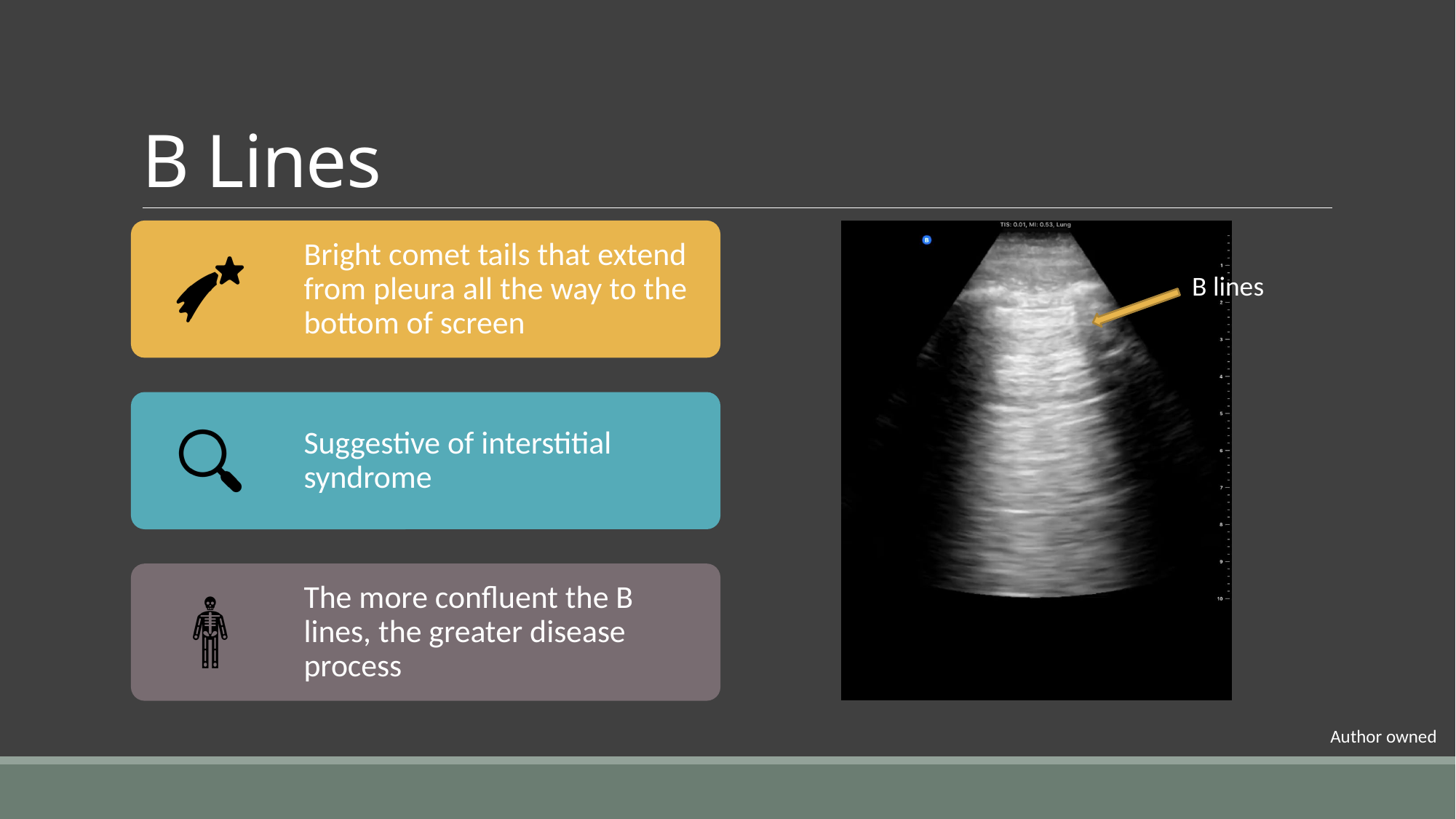

# B Lines
B lines
Author owned

## Slide 9
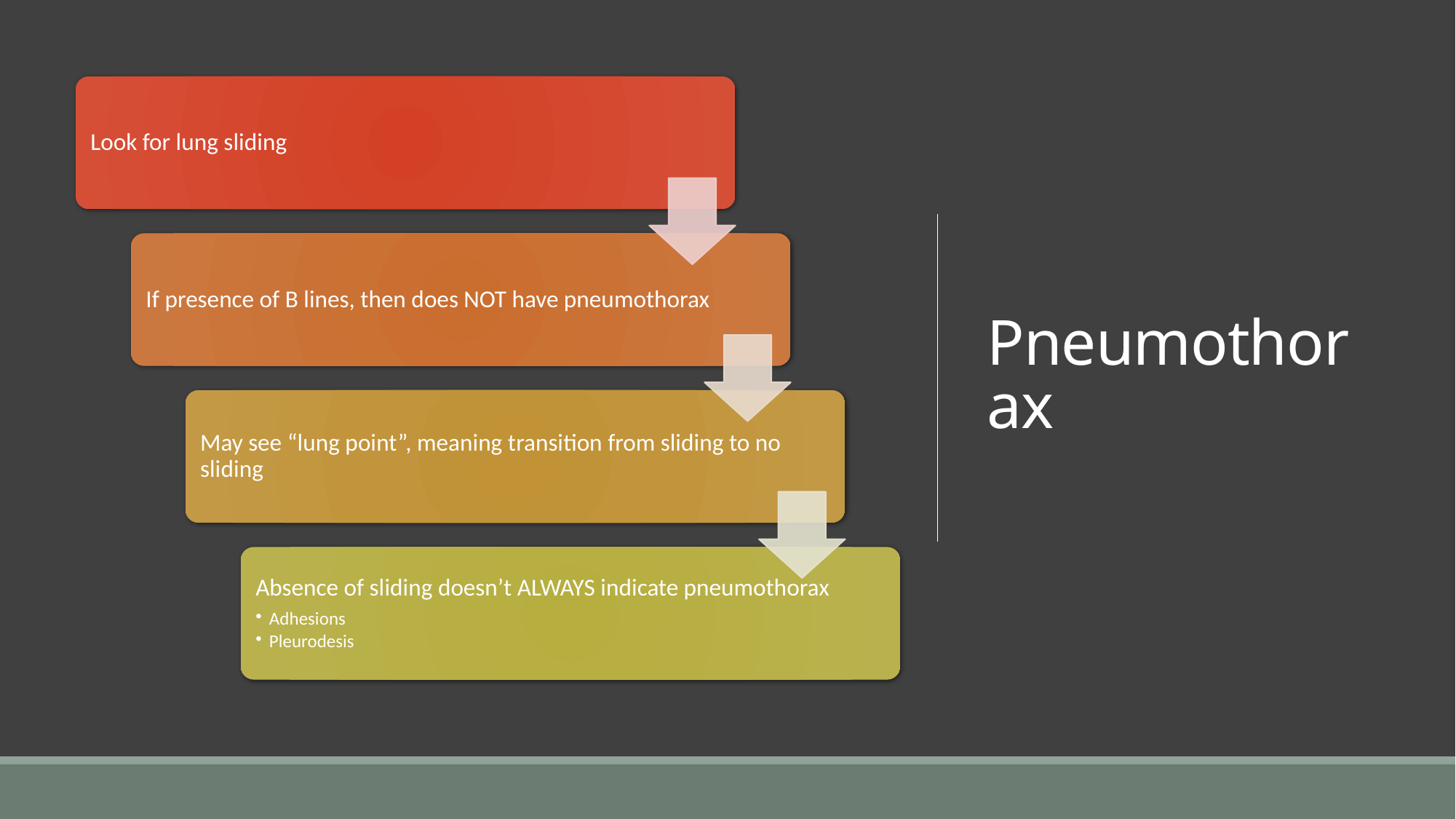

# Pneumothorax

## Slide 10
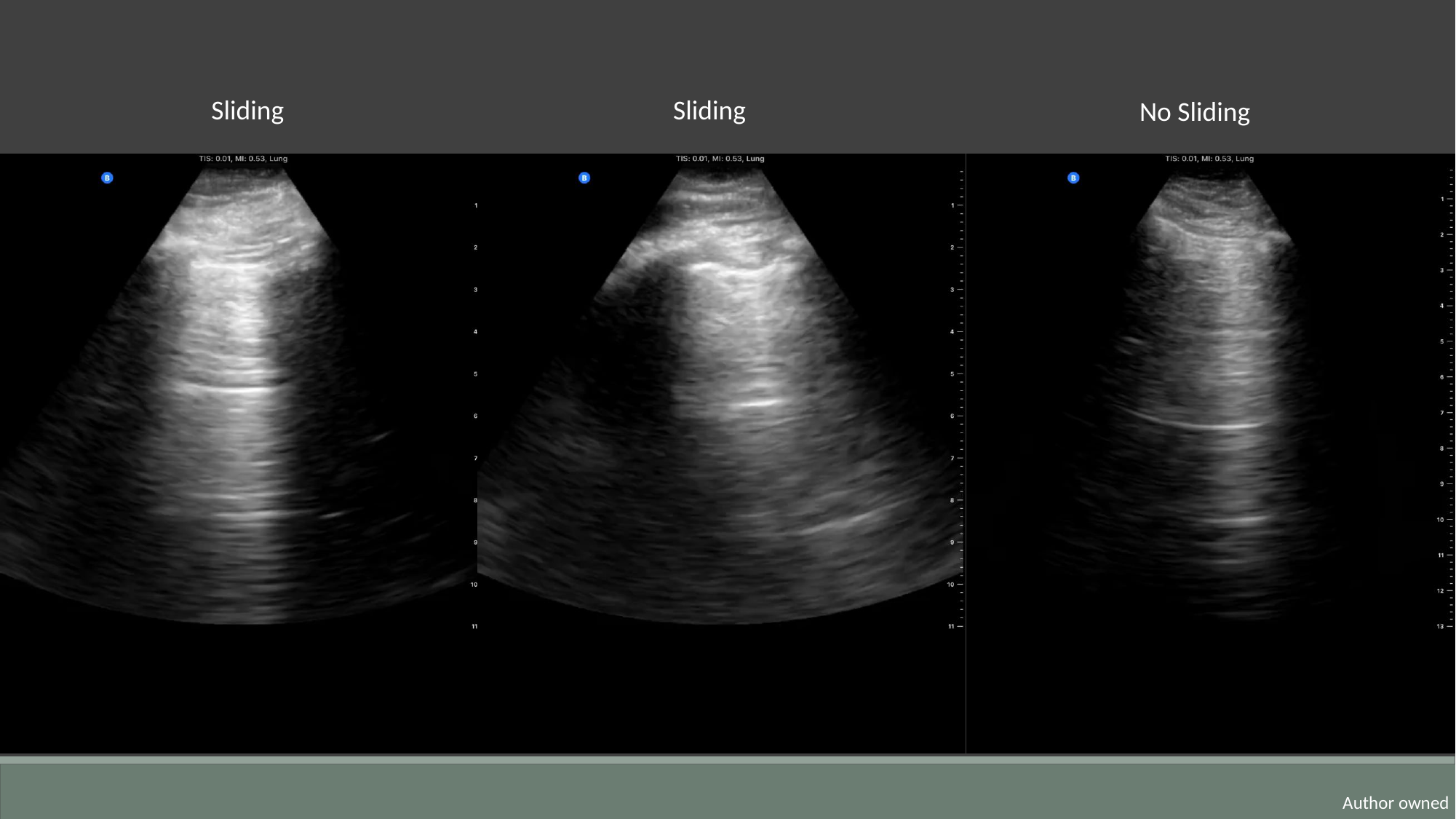

Sliding
Sliding
No Sliding
Author owned

## Slide 11
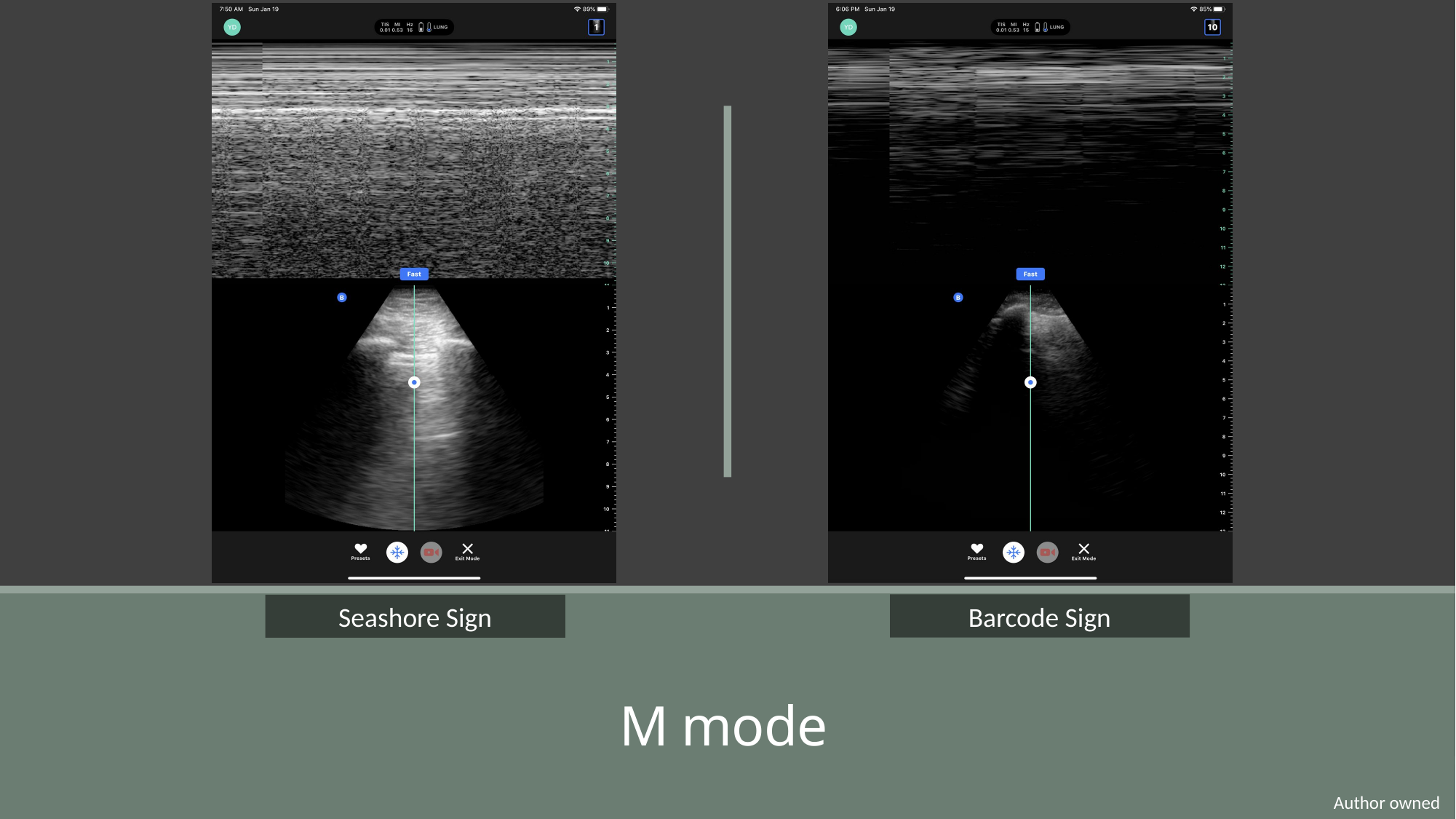

Barcode Sign
Seashore Sign
# M mode
Author owned

## Slide 12
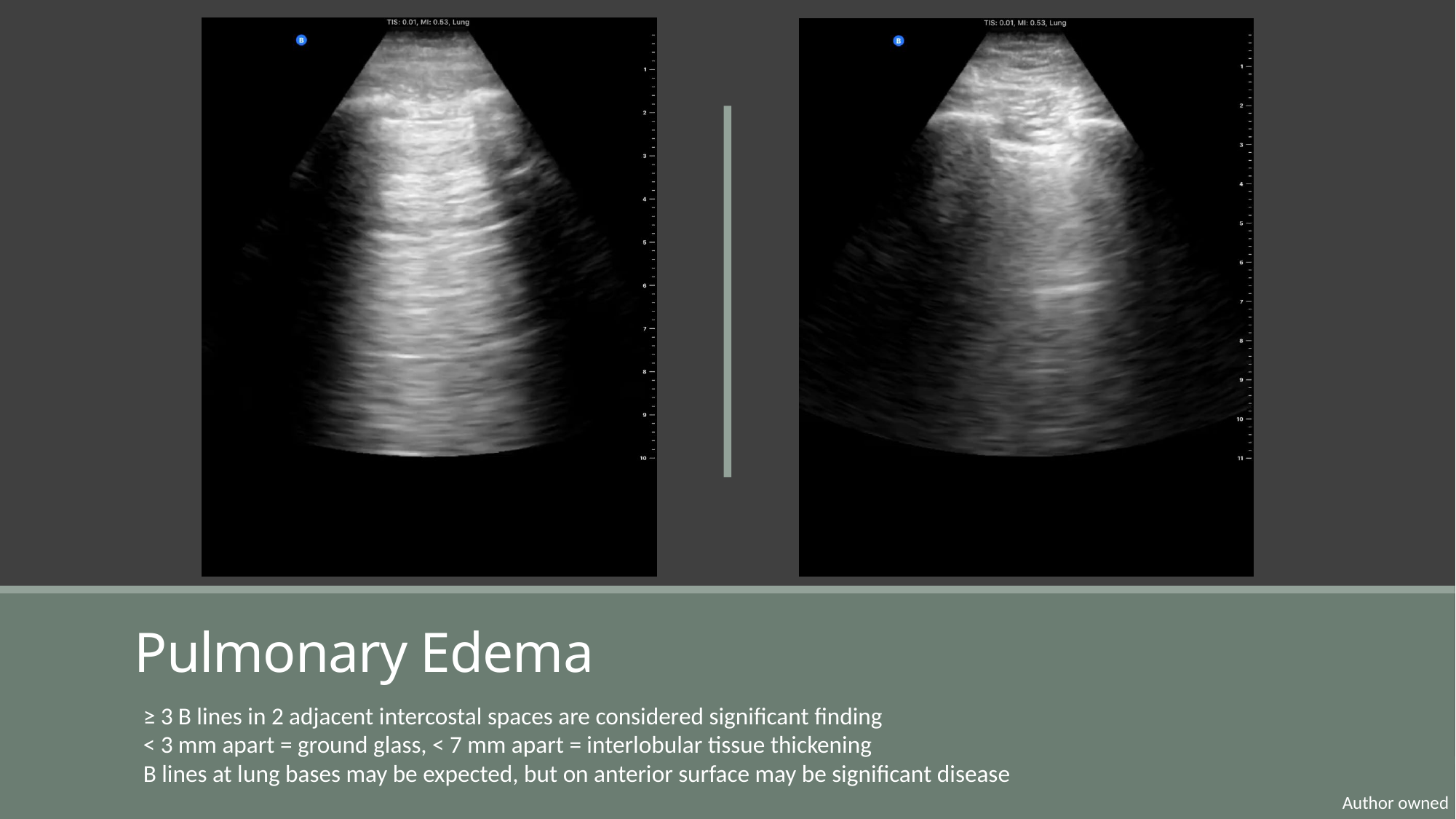

# Pulmonary Edema
≥ 3 B lines in 2 adjacent intercostal spaces are considered significant finding
< 3 mm apart = ground glass, < 7 mm apart = interlobular tissue thickening
B lines at lung bases may be expected, but on anterior surface may be significant disease
Author owned

## Slide 13
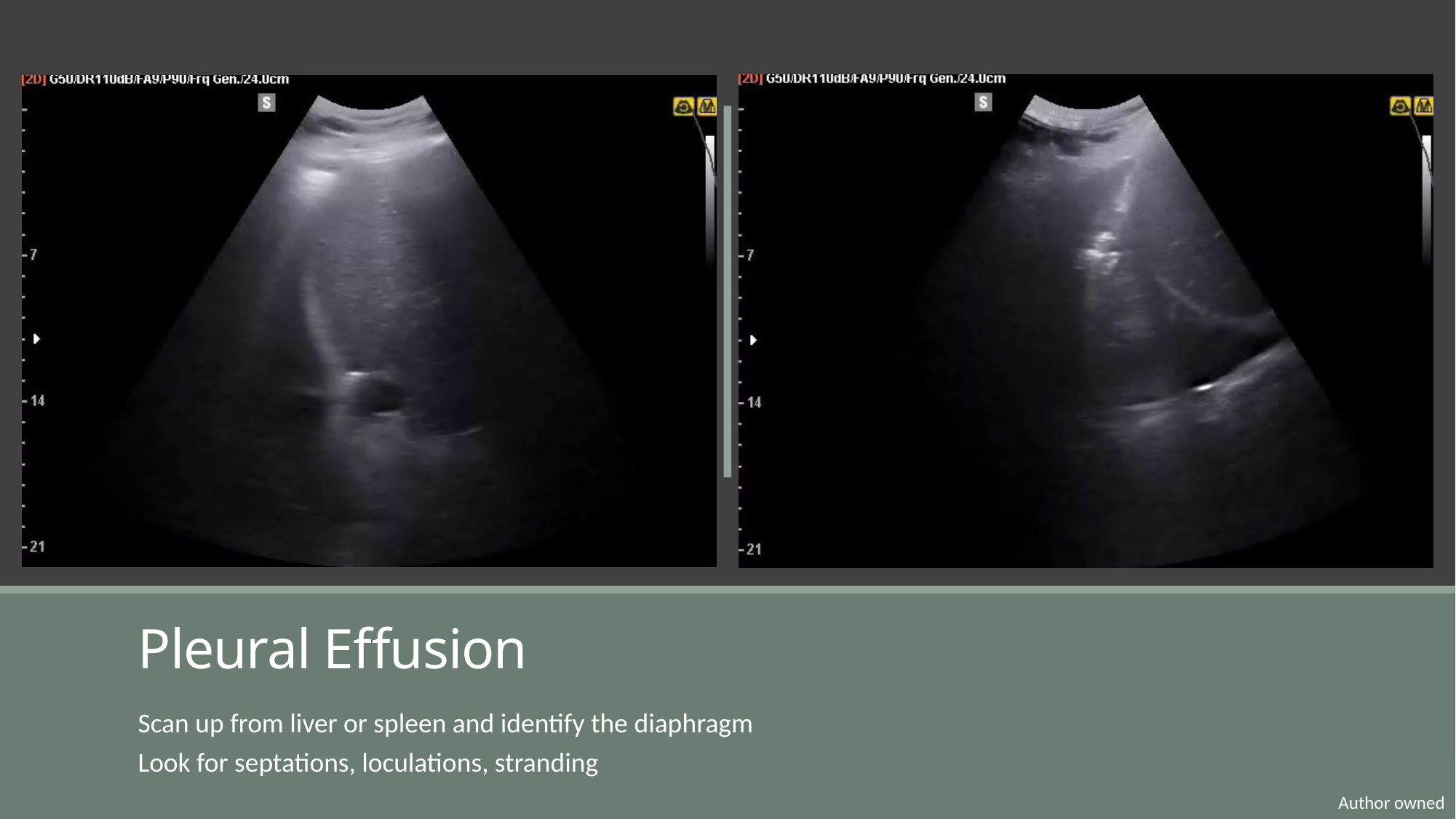

# Pleural Effusion
Scan up from liver or spleen and identify the diaphragm
Look for septations, loculations, stranding
Author owned

## Slide 14
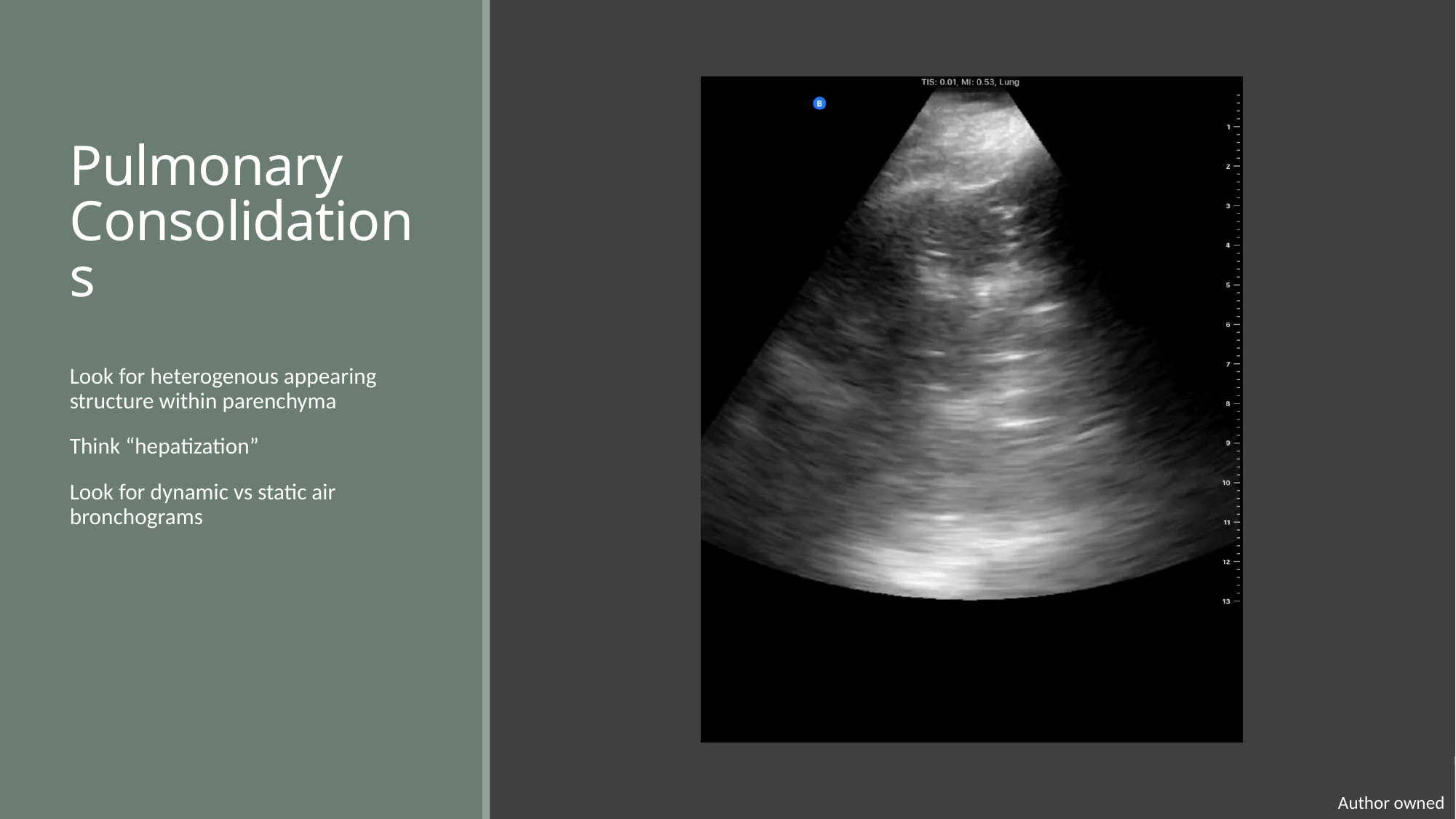

# Pulmonary Consolidations
Look for heterogenous appearing structure within parenchyma
Think “hepatization”
Look for dynamic vs static air bronchograms
Author owned

## Slide 15
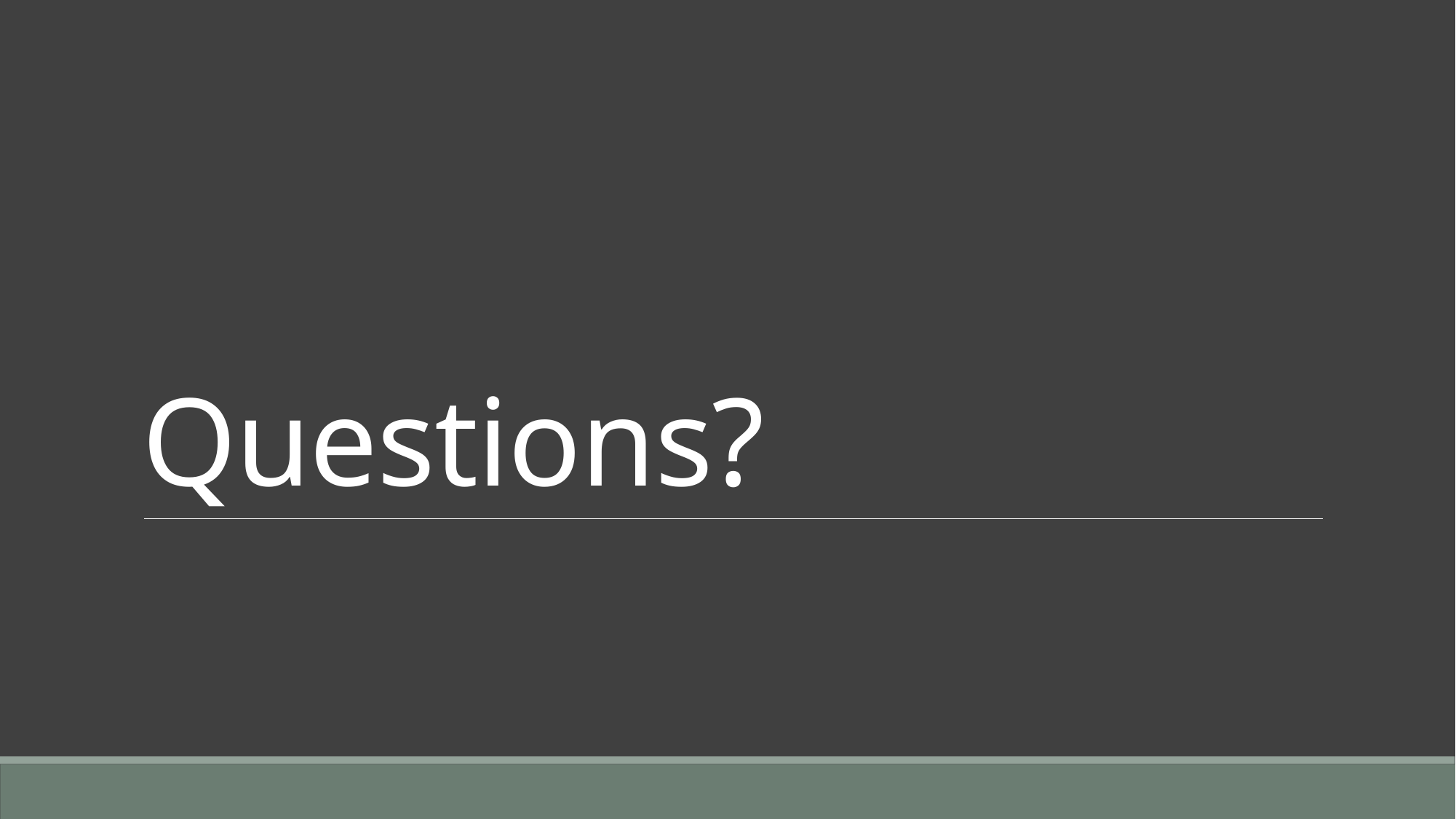

# Questions?
