# Supplementary material for: An Introductory Point-of-Care Ultrasound Curriculum for an Anesthesiology Residency Program
Source: MedEdPORTAL. 2022 Dec 23;18:11291. doi: 10.15766/mep_2374-8265.11291 (PMC9780414; doi:10.15766/mep_2374-8265.11291)
Supplement: Supplementary file 1 — Ultrasound Basics.pptxLung Ultrasound.pptxCardiac Ultrasound.pptxVascular Access Ultrasound.pptxAirway Ultrasound.pptxAbdominal Ultrasound.pptxNeuraxial Ultrasound.pptxChecklist for POCUS Scanning.docxPOCUS CA1 Curriculum Pretest.pptxPOCUS CA1 Curriculum Posttest.pptxPOCUS Survey.docx [file mep_2374-8265.11291-s001.zip › C. Cardiac Ultrasound.pptx]

## Slide 1
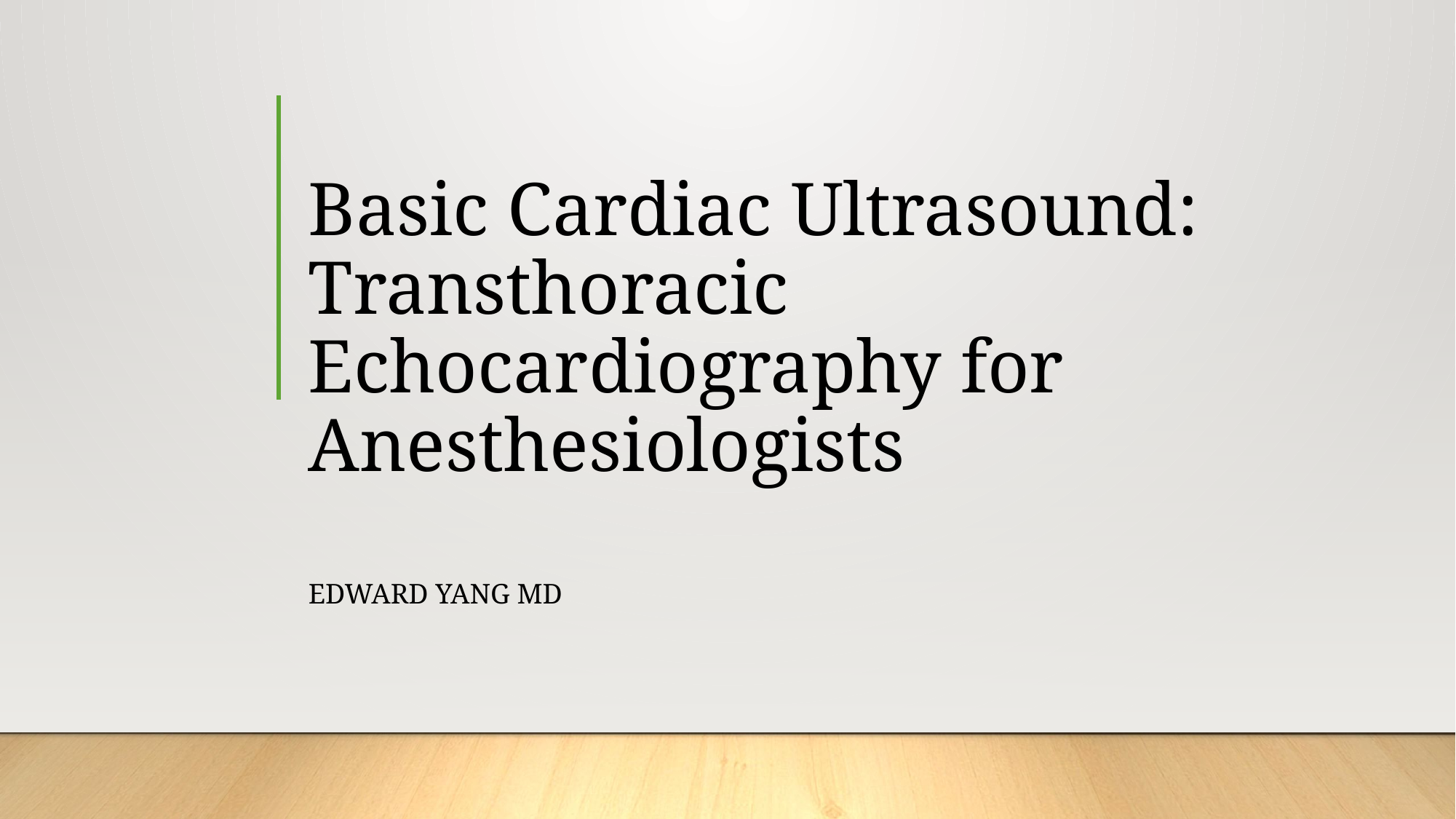

# Basic Cardiac Ultrasound: Transthoracic Echocardiography for Anesthesiologists
Edward Yang MD

## Slide 2
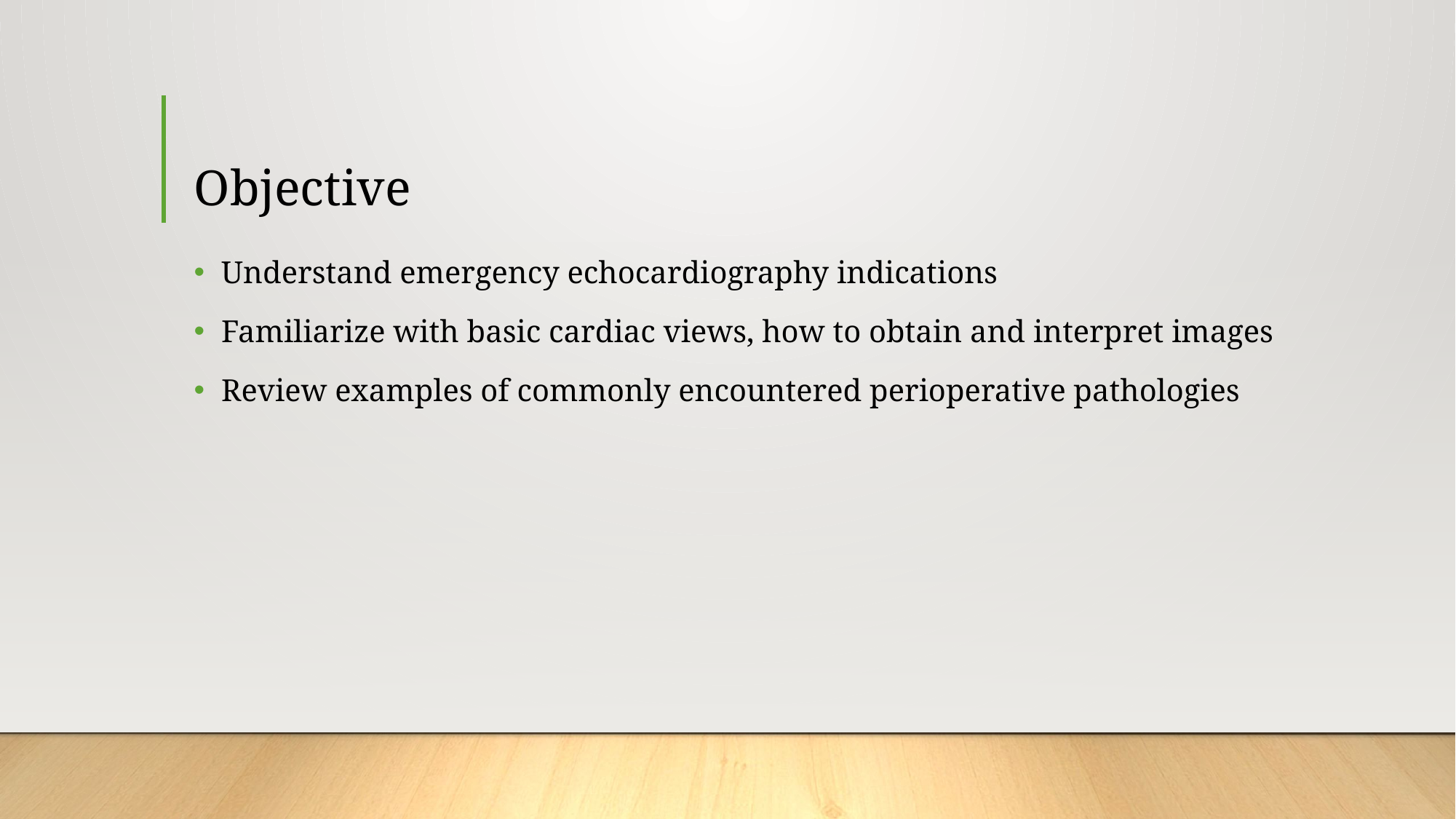

# Objective
Understand emergency echocardiography indications
Familiarize with basic cardiac views, how to obtain and interpret images
Review examples of commonly encountered perioperative pathologies

## Slide 3
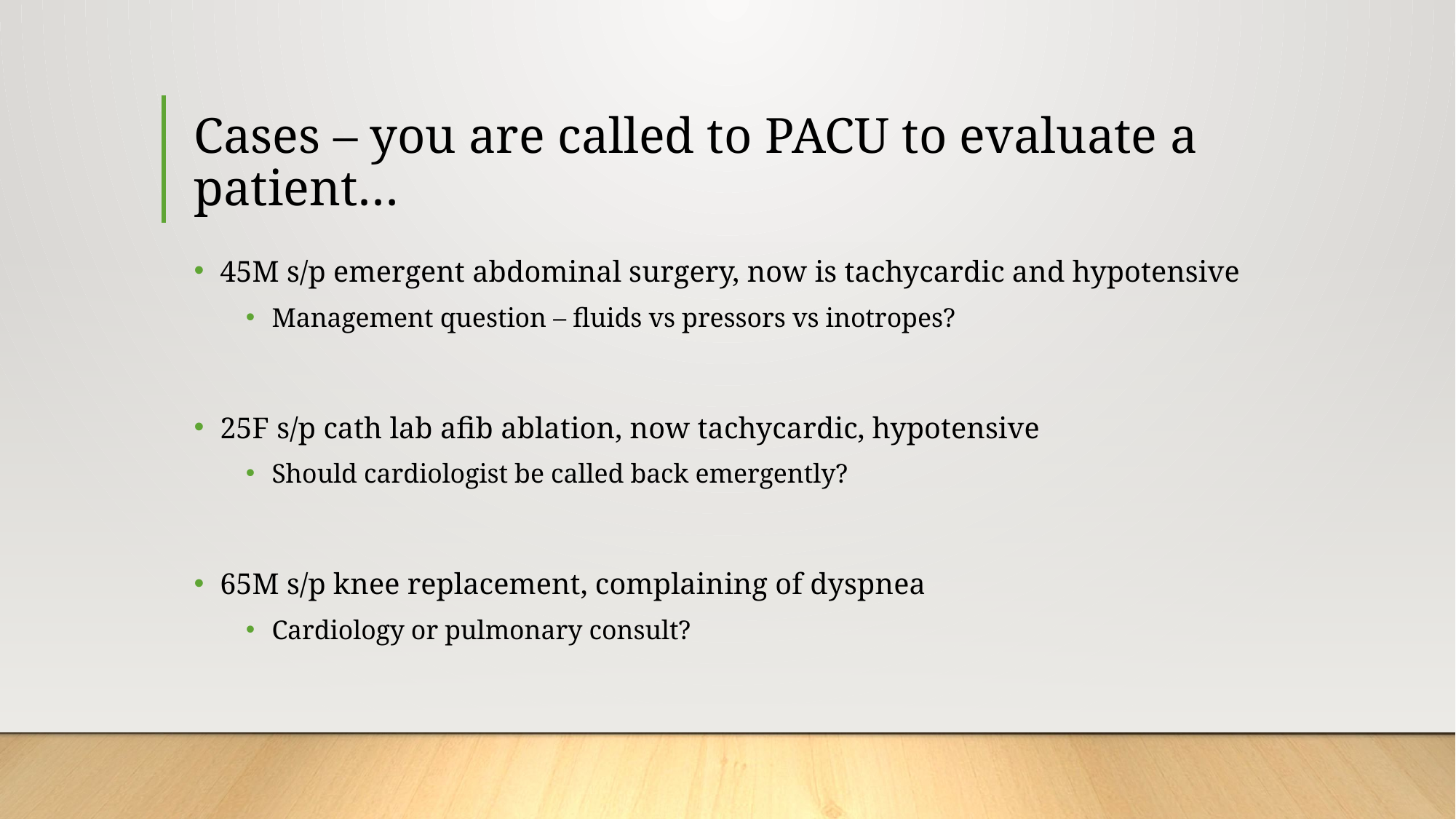

# Cases – you are called to PACU to evaluate a patient…
45M s/p emergent abdominal surgery, now is tachycardic and hypotensive
Management question – fluids vs pressors vs inotropes?
25F s/p cath lab afib ablation, now tachycardic, hypotensive
Should cardiologist be called back emergently?
65M s/p knee replacement, complaining of dyspnea
Cardiology or pulmonary consult?

## Slide 4
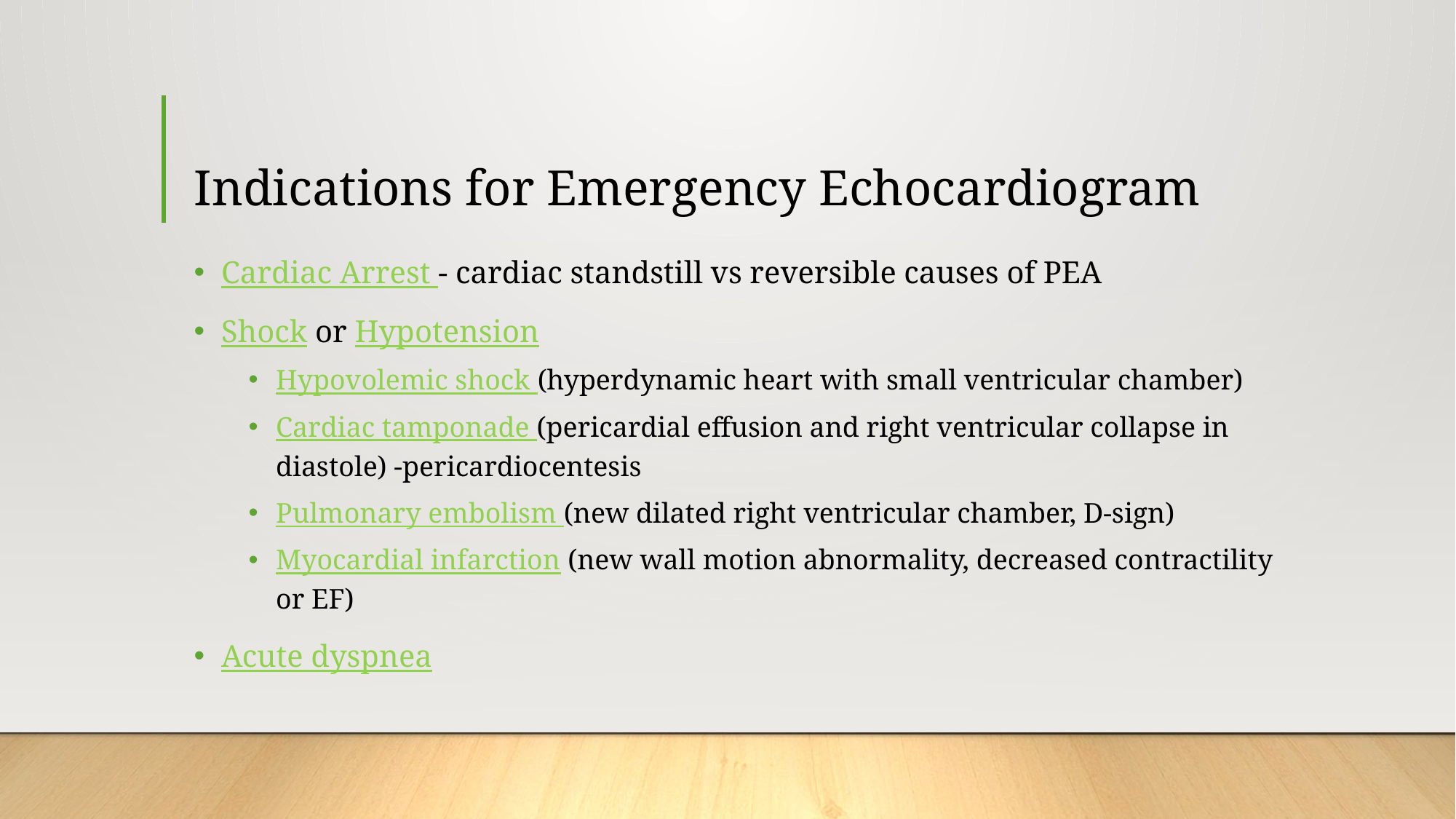

# Indications for Emergency Echocardiogram
Cardiac Arrest - cardiac standstill vs reversible causes of PEA
Shock or Hypotension
Hypovolemic shock (hyperdynamic heart with small ventricular chamber)
Cardiac tamponade (pericardial effusion and right ventricular collapse in diastole) -pericardiocentesis
Pulmonary embolism (new dilated right ventricular chamber, D-sign)
Myocardial infarction (new wall motion abnormality, decreased contractility or EF)
Acute dyspnea

## Slide 5
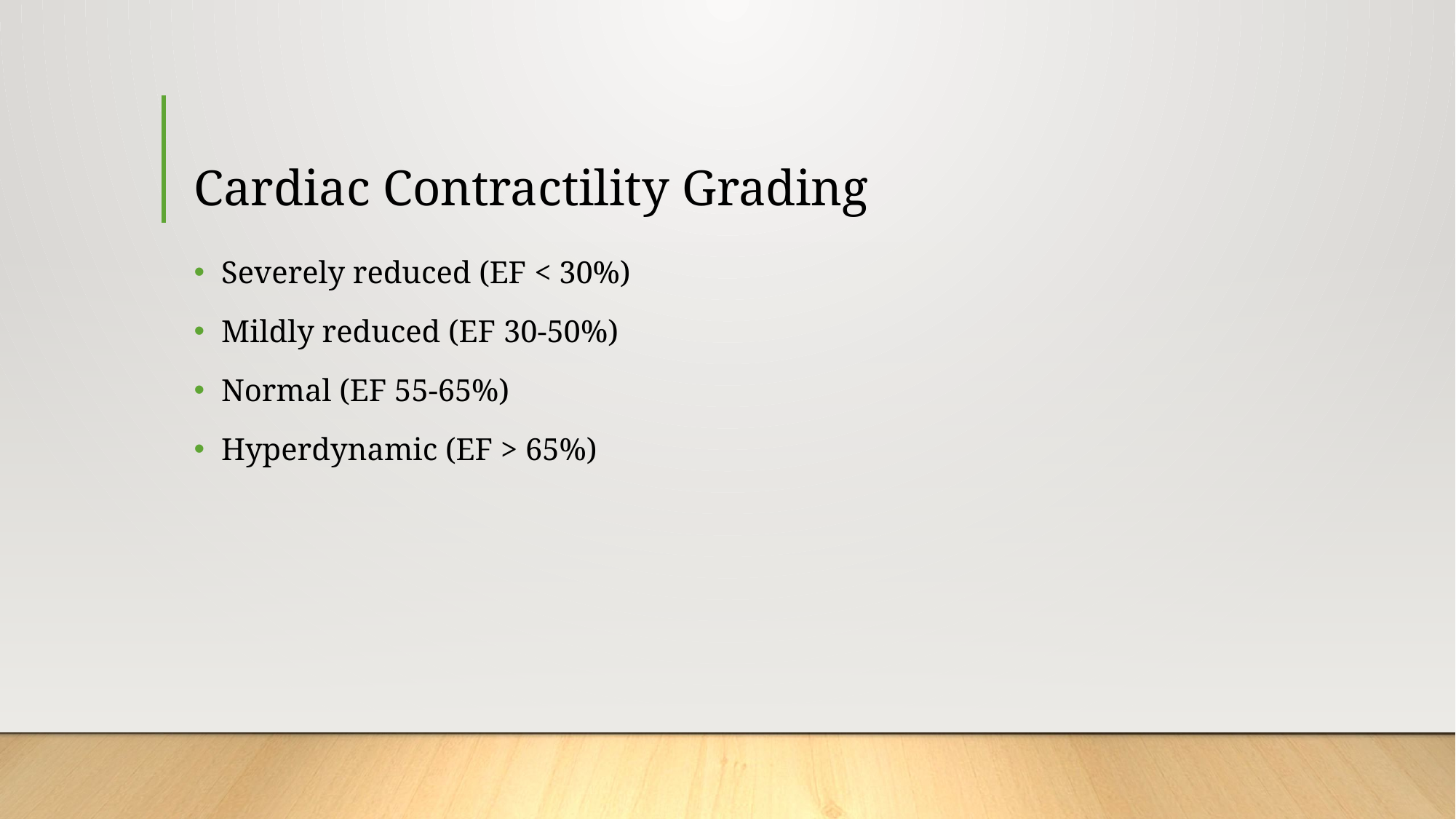

# Cardiac Contractility Grading
Severely reduced (EF < 30%)
Mildly reduced (EF 30-50%)
Normal (EF 55-65%)
Hyperdynamic (EF > 65%)

## Slide 6
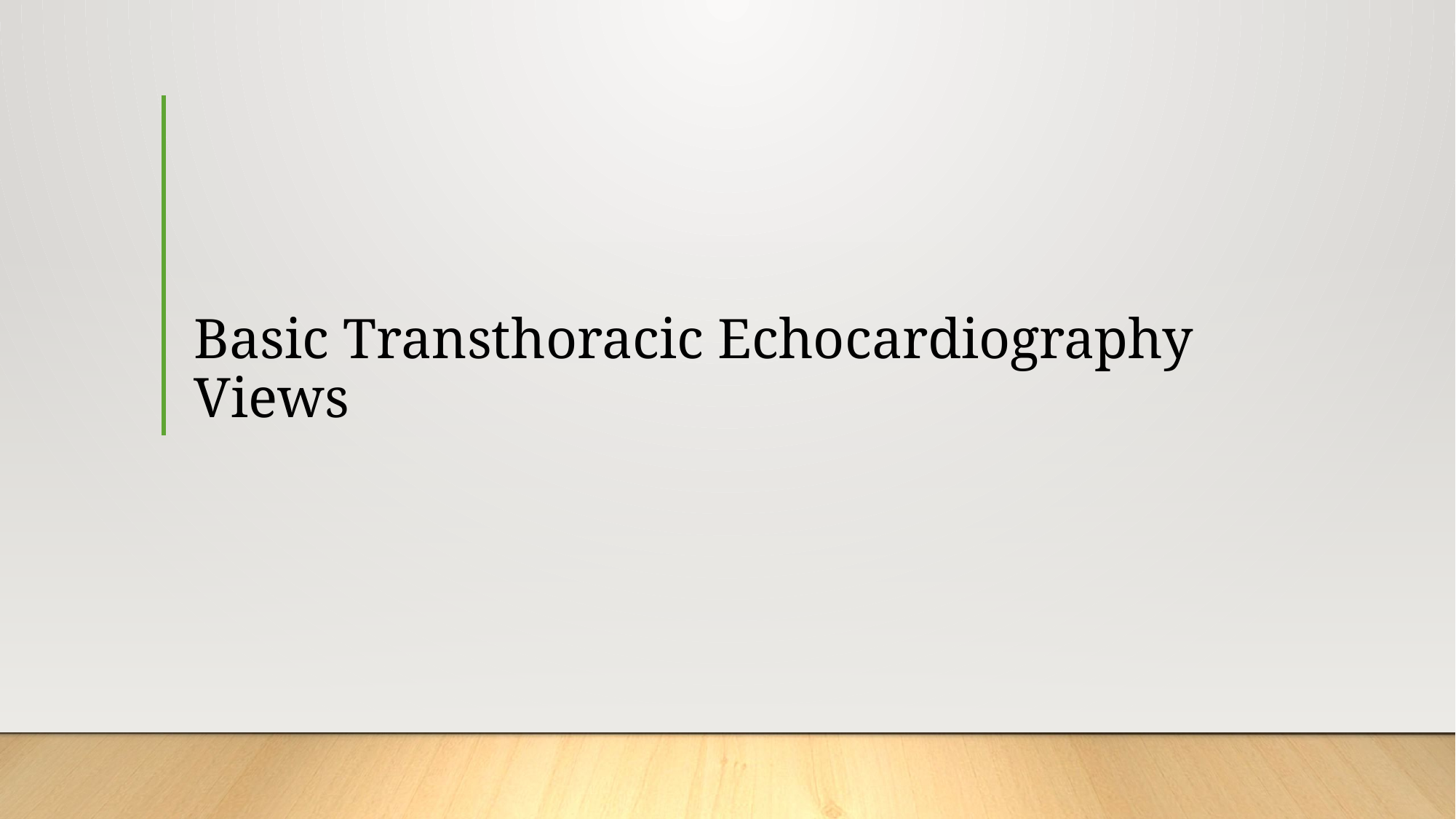

# Basic Transthoracic Echocardiography Views

## Slide 7
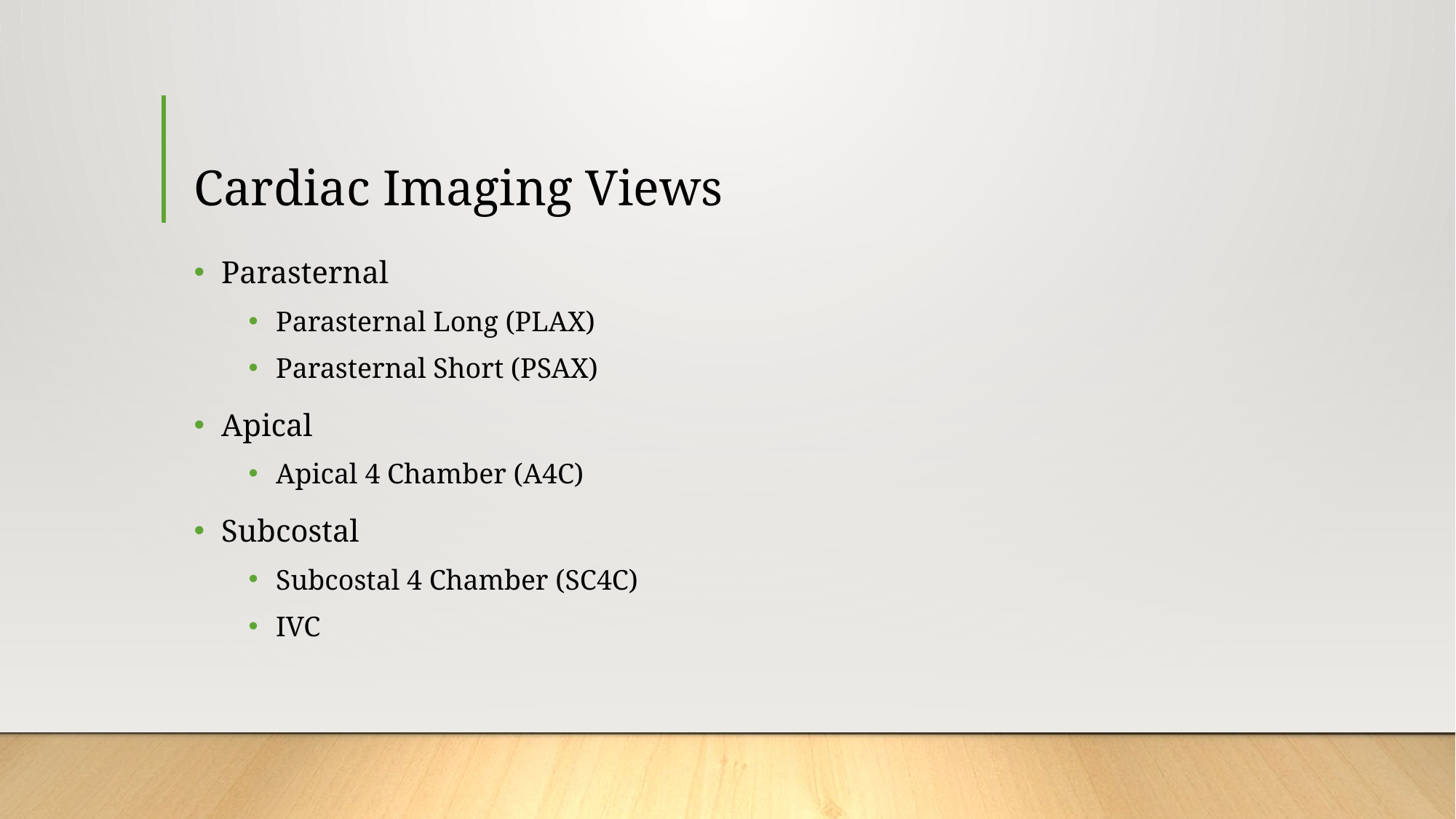

# Cardiac Imaging Views
Parasternal
Parasternal Long (PLAX)
Parasternal Short (PSAX)
Apical
Apical 4 Chamber (A4C)
Subcostal
Subcostal 4 Chamber (SC4C)
IVC

## Slide 8
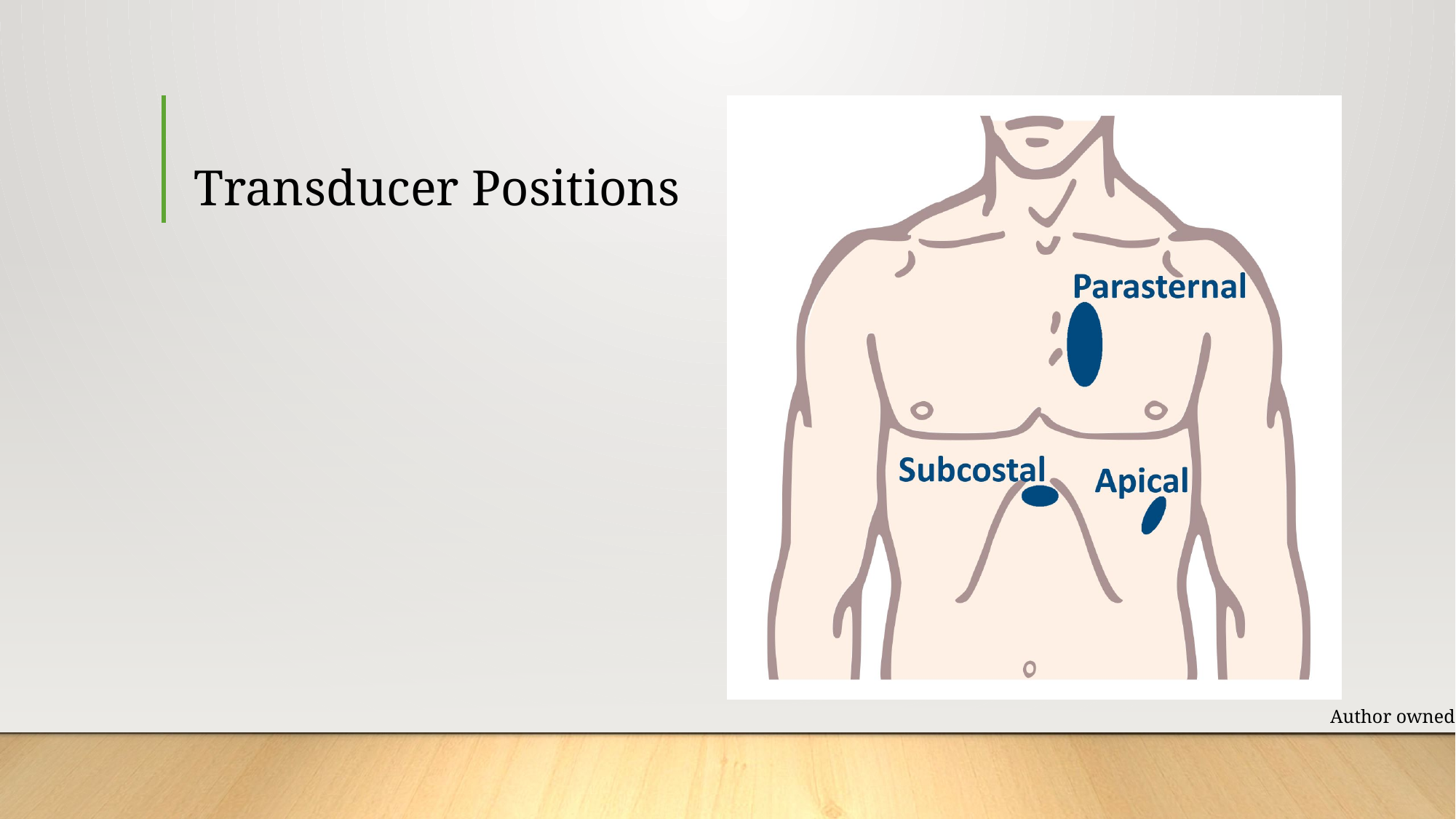

# Transducer Positions
Author owned

## Slide 9
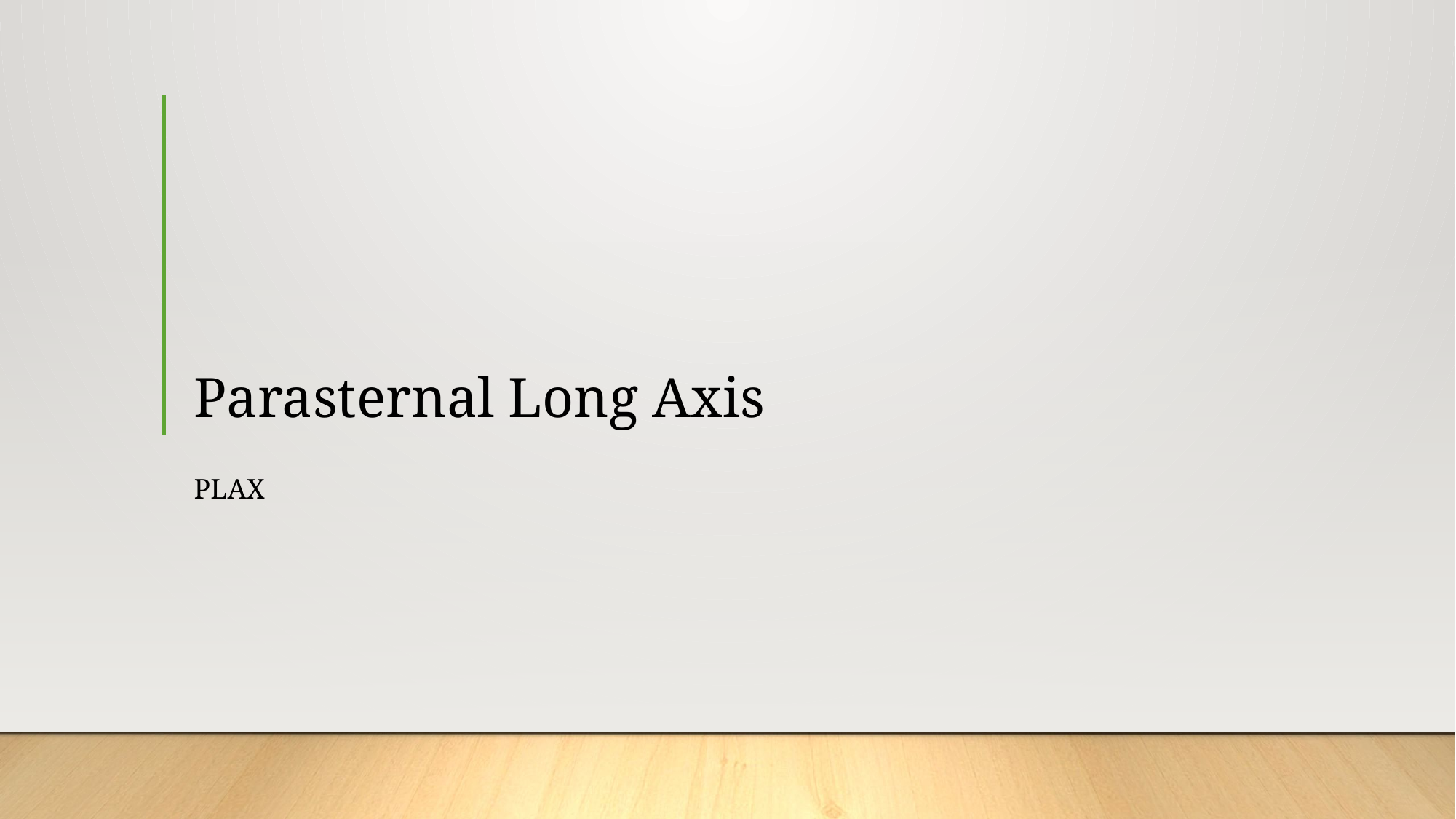

# Parasternal Long Axis
PLAX

## Slide 10
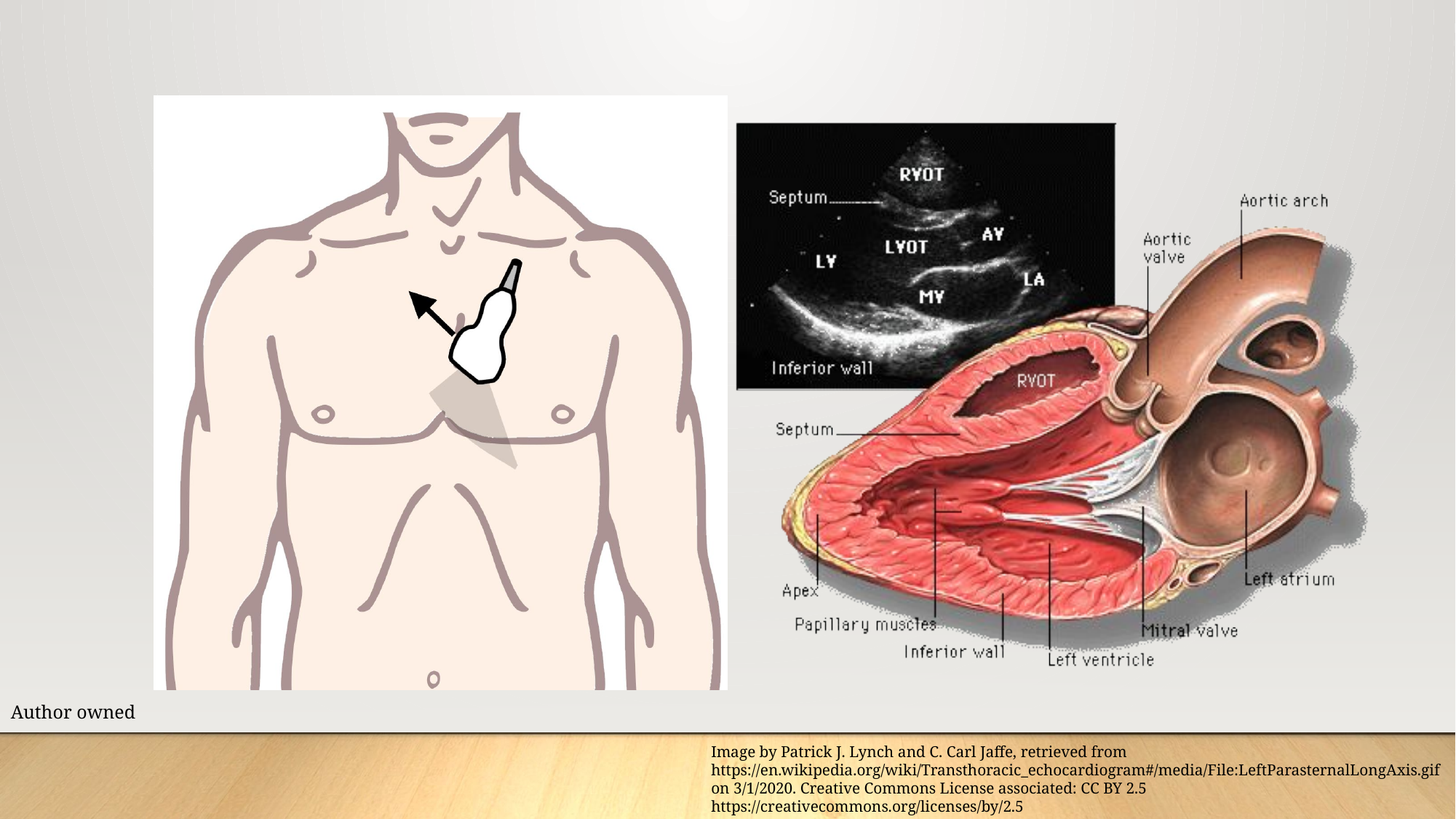

#
Author owned
Image by Patrick J. Lynch and C. Carl Jaffe, retrieved from https://en.wikipedia.org/wiki/Transthoracic_echocardiogram#/media/File:LeftParasternalLongAxis.gif on 3/1/2020. Creative Commons License associated: CC BY 2.5 https://creativecommons.org/licenses/by/2.5

## Slide 11
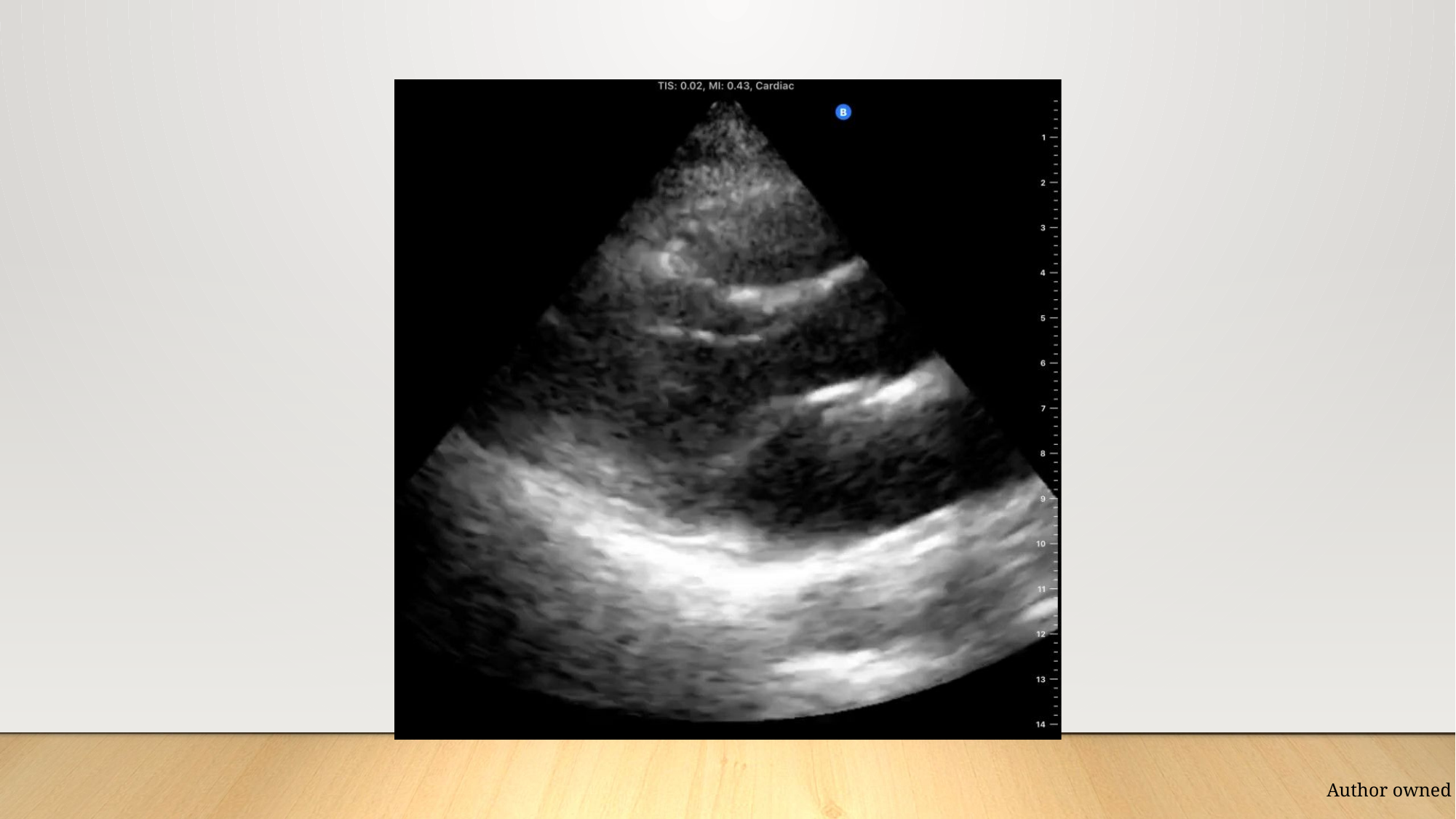

Author owned

## Slide 12
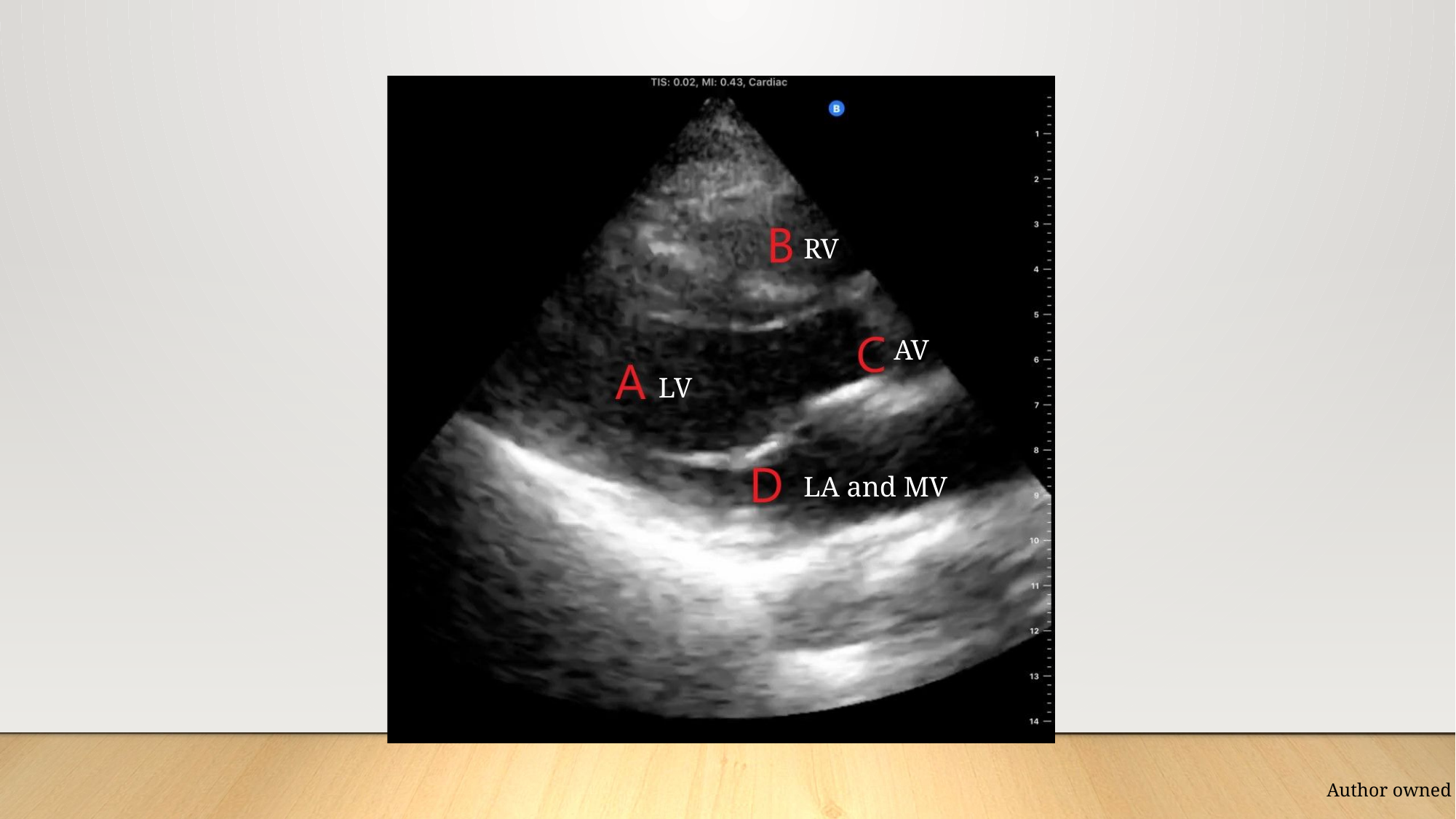

RV
AV
LV
LA and MV
Author owned

## Slide 13
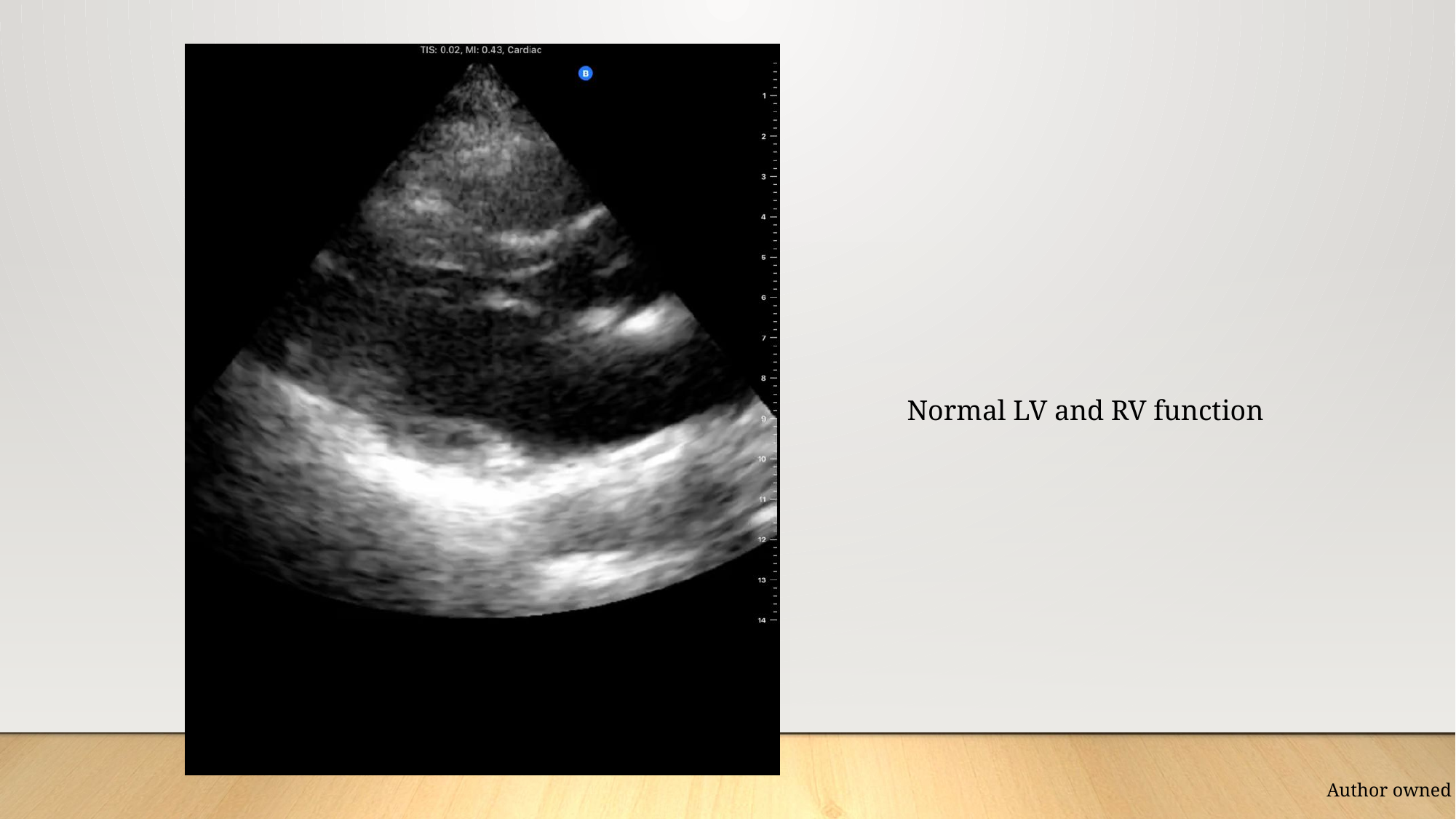

Normal LV and RV function
Author owned

## Slide 14
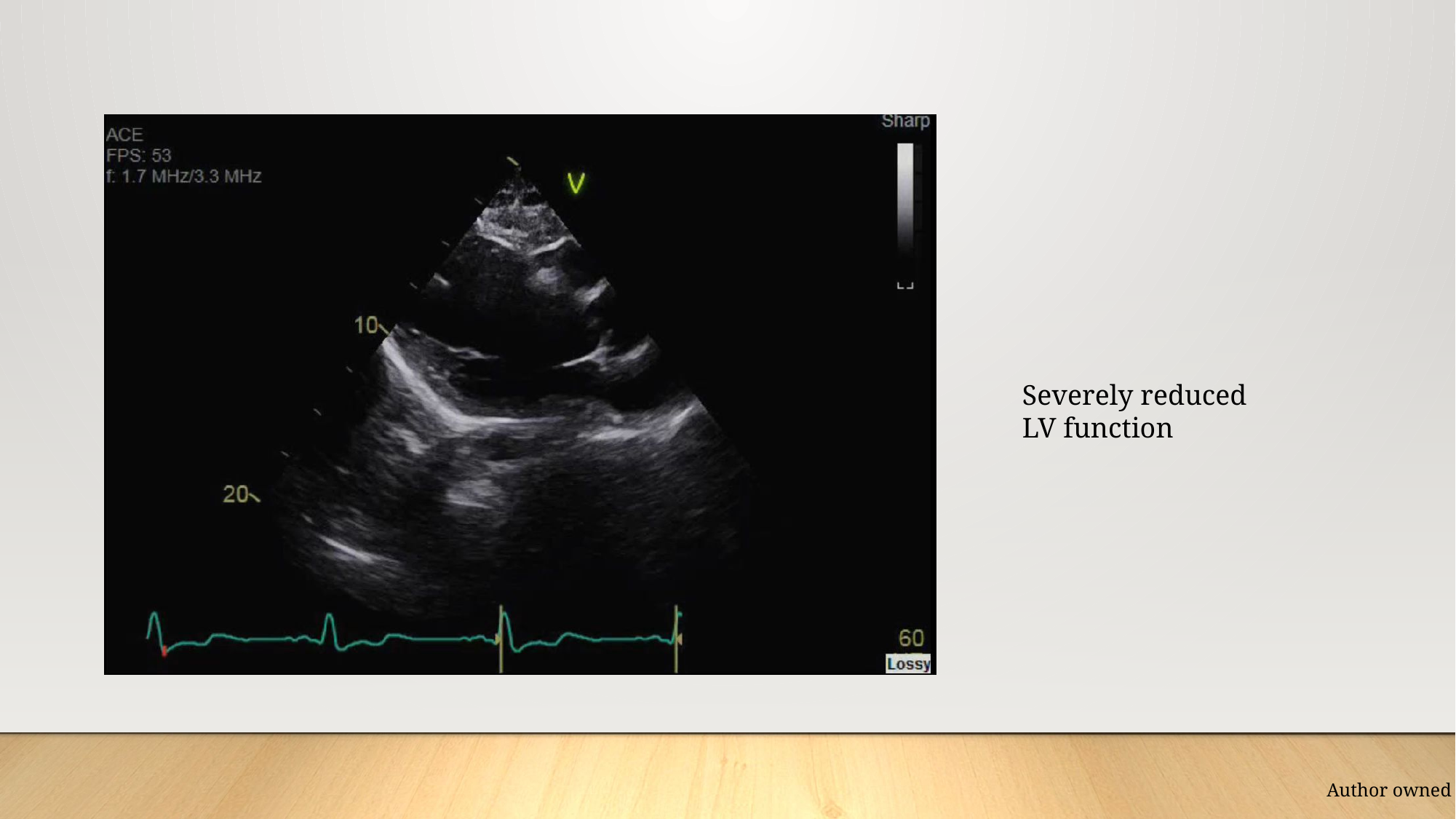

Severely reduced LV function
Author owned

## Slide 15
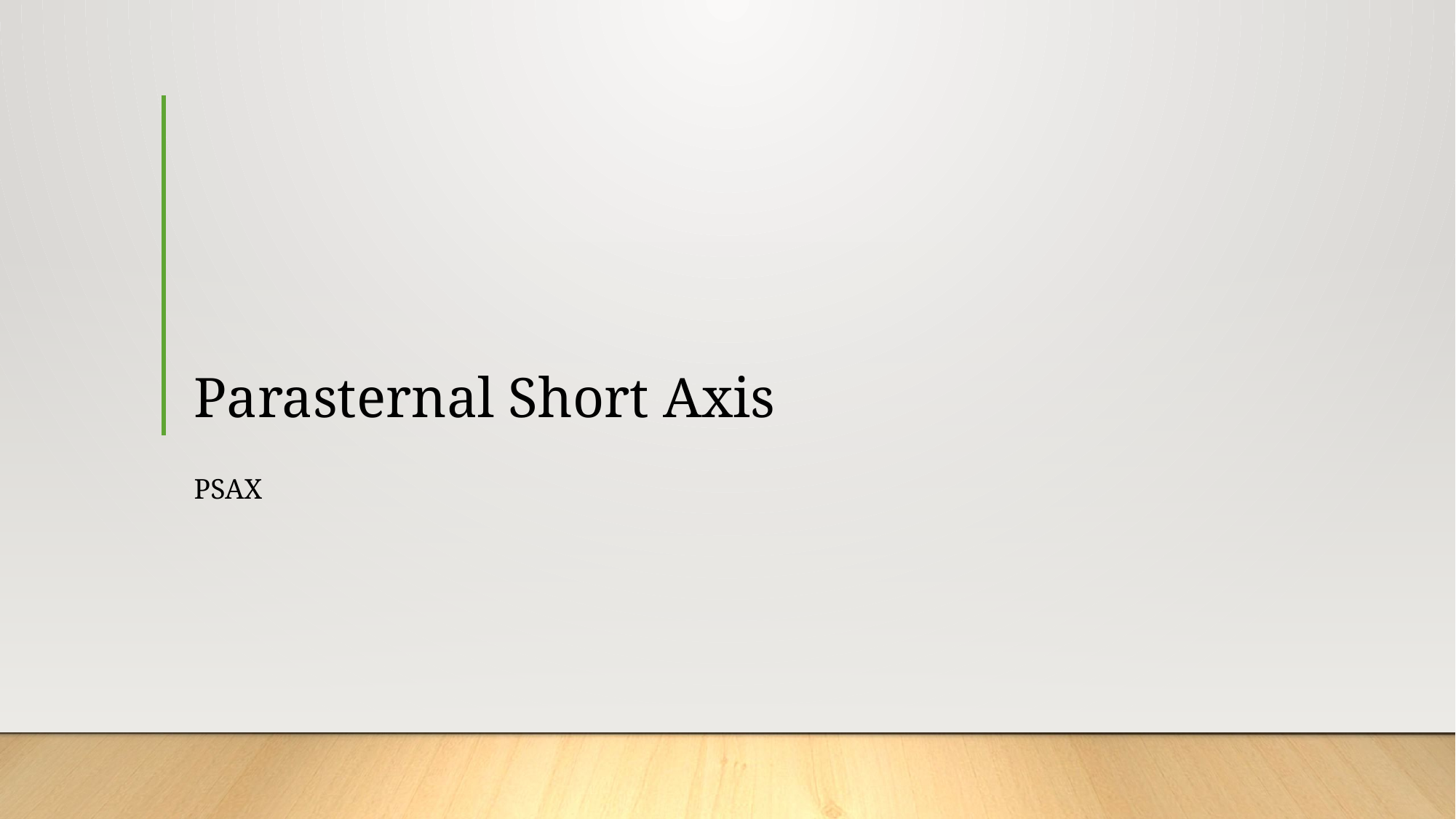

# Parasternal Short Axis
PSAX

## Slide 16
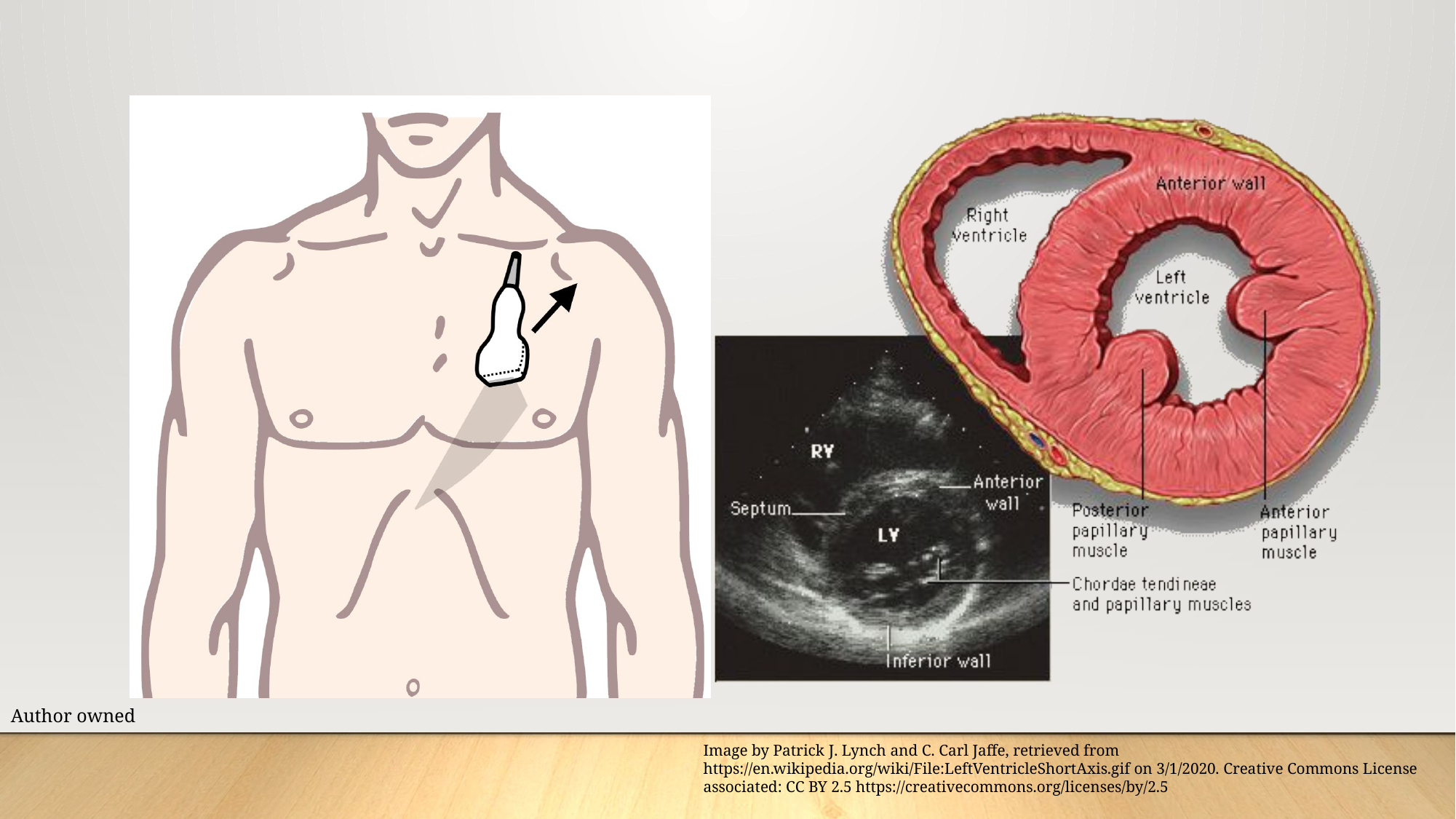

#
Author owned
Image by Patrick J. Lynch and C. Carl Jaffe, retrieved from https://en.wikipedia.org/wiki/File:LeftVentricleShortAxis.gif on 3/1/2020. Creative Commons License associated: CC BY 2.5 https://creativecommons.org/licenses/by/2.5

## Slide 17
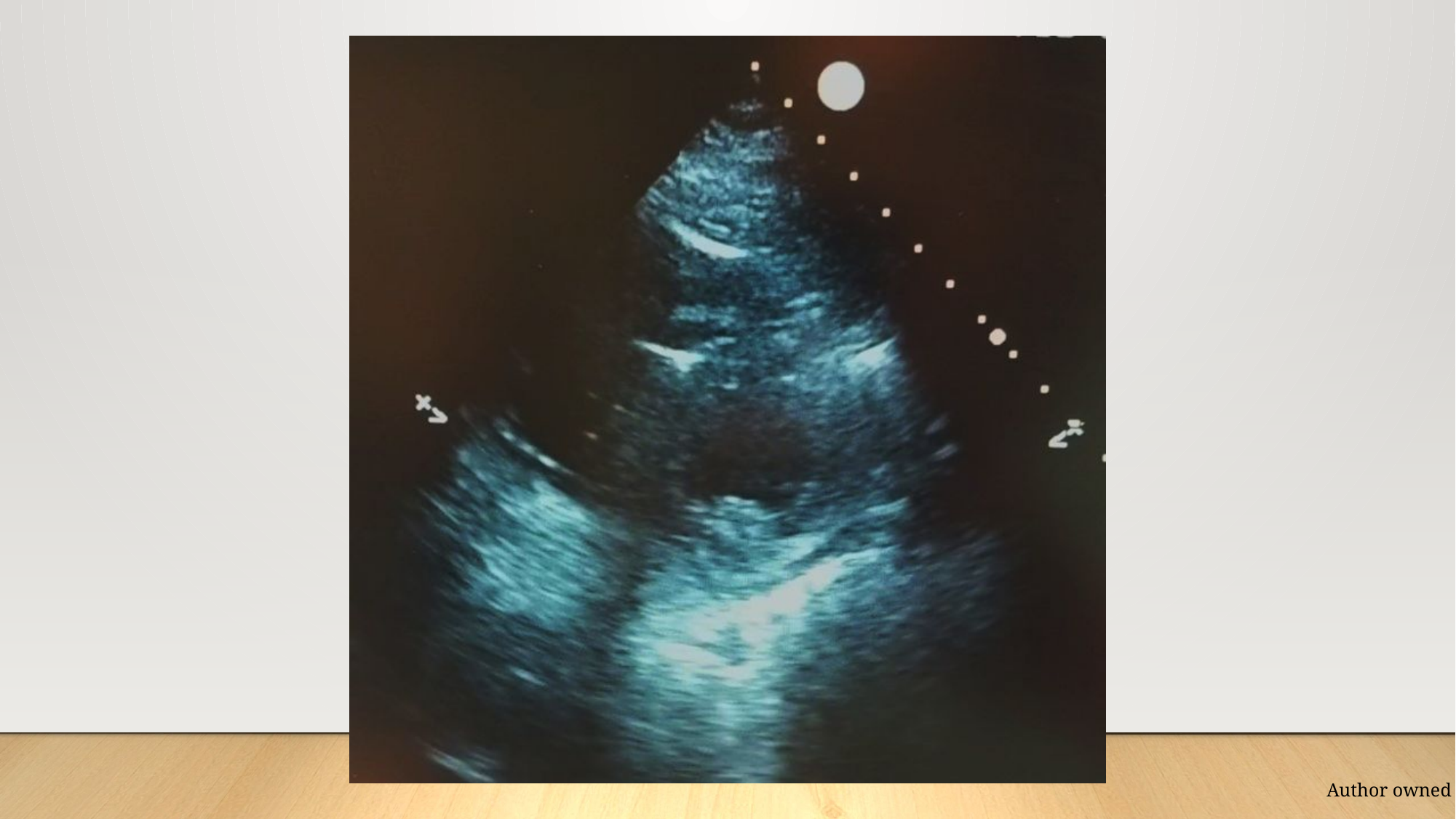

Author owned

## Slide 18
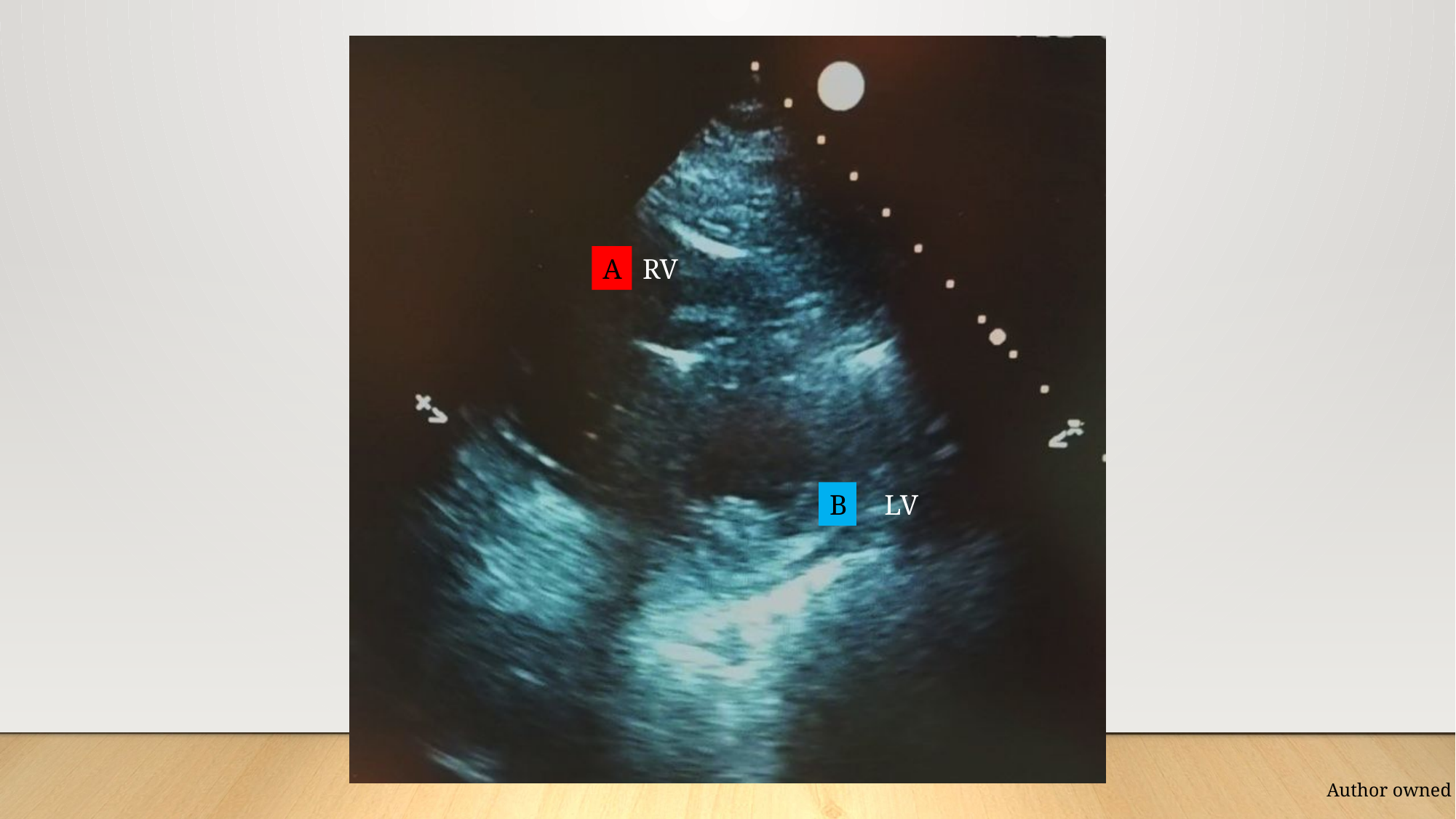

A
RV
B
LV
Author owned

## Slide 19
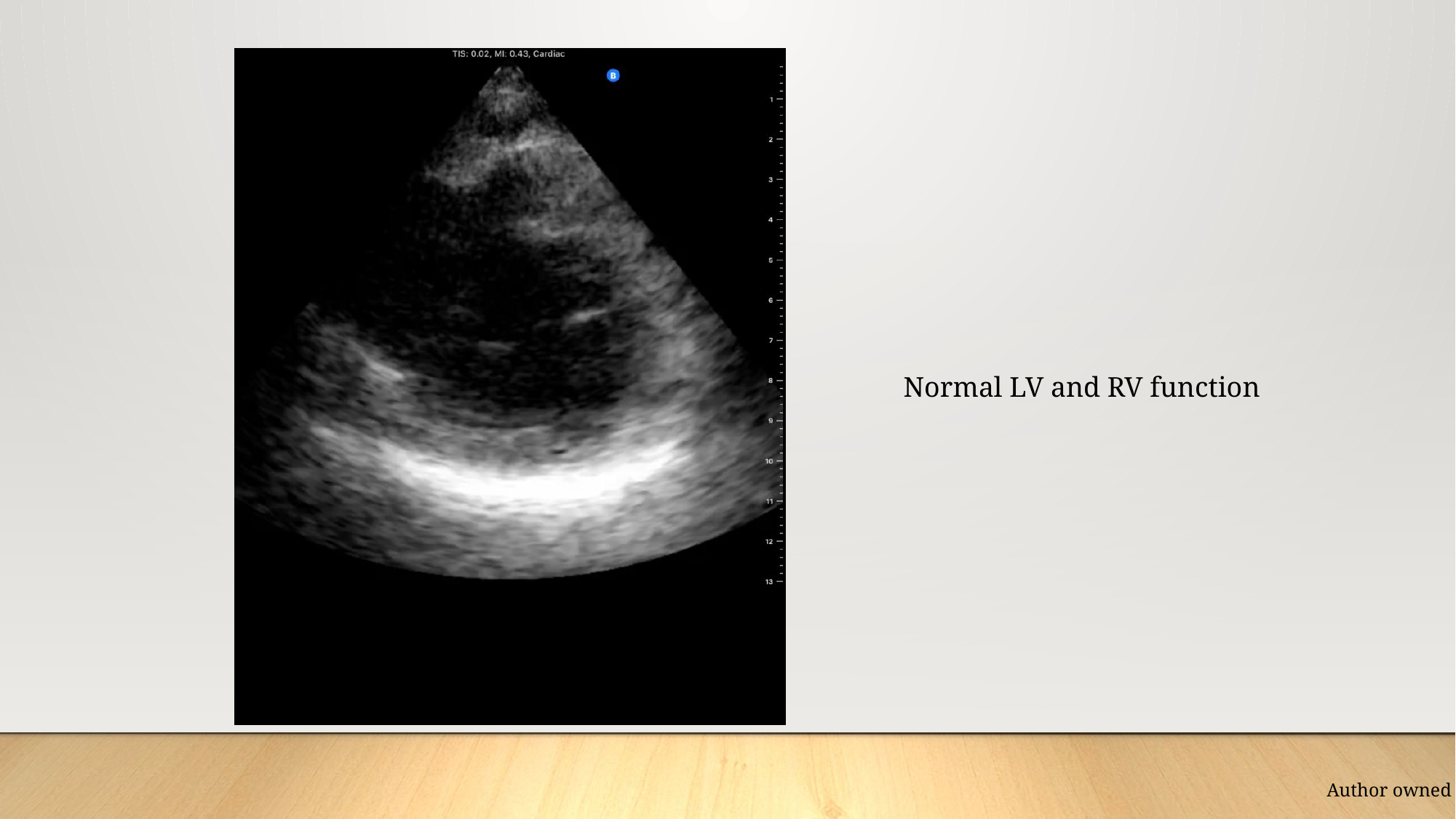

Normal LV and RV function
Author owned

## Slide 20
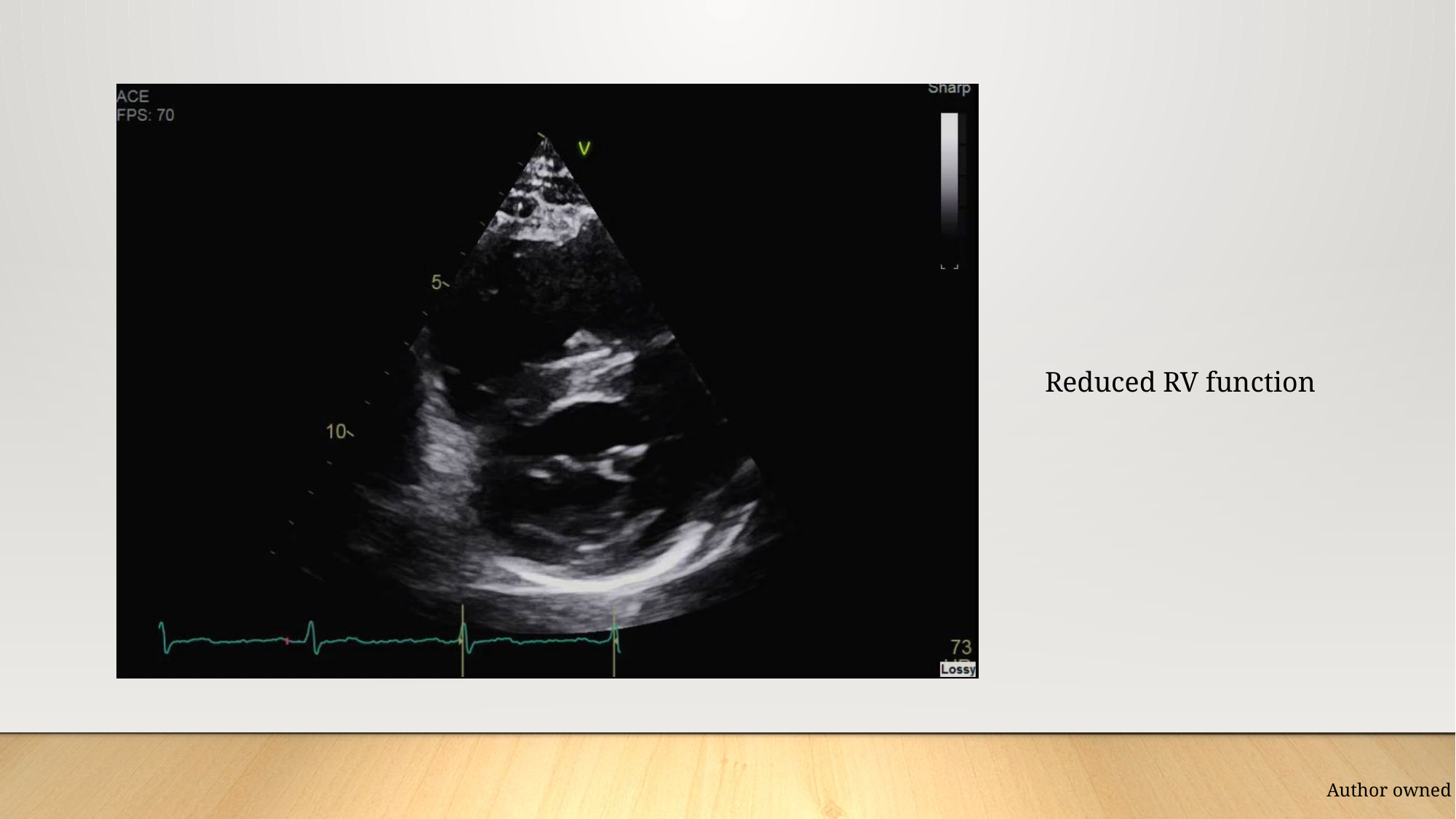

Reduced RV function
Author owned

## Slide 21
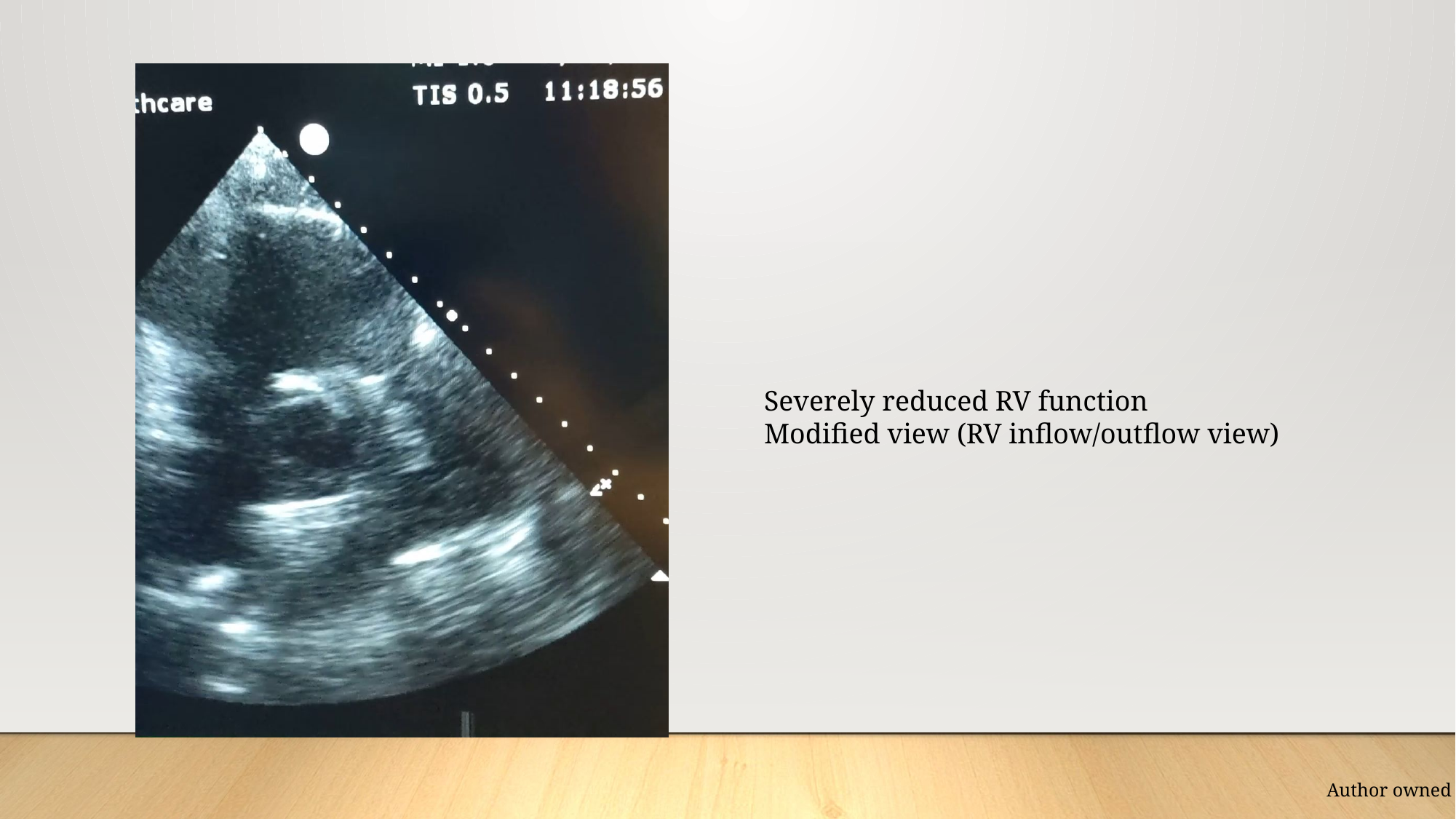

Severely reduced RV function
Modified view (RV inflow/outflow view)
Author owned

## Slide 22
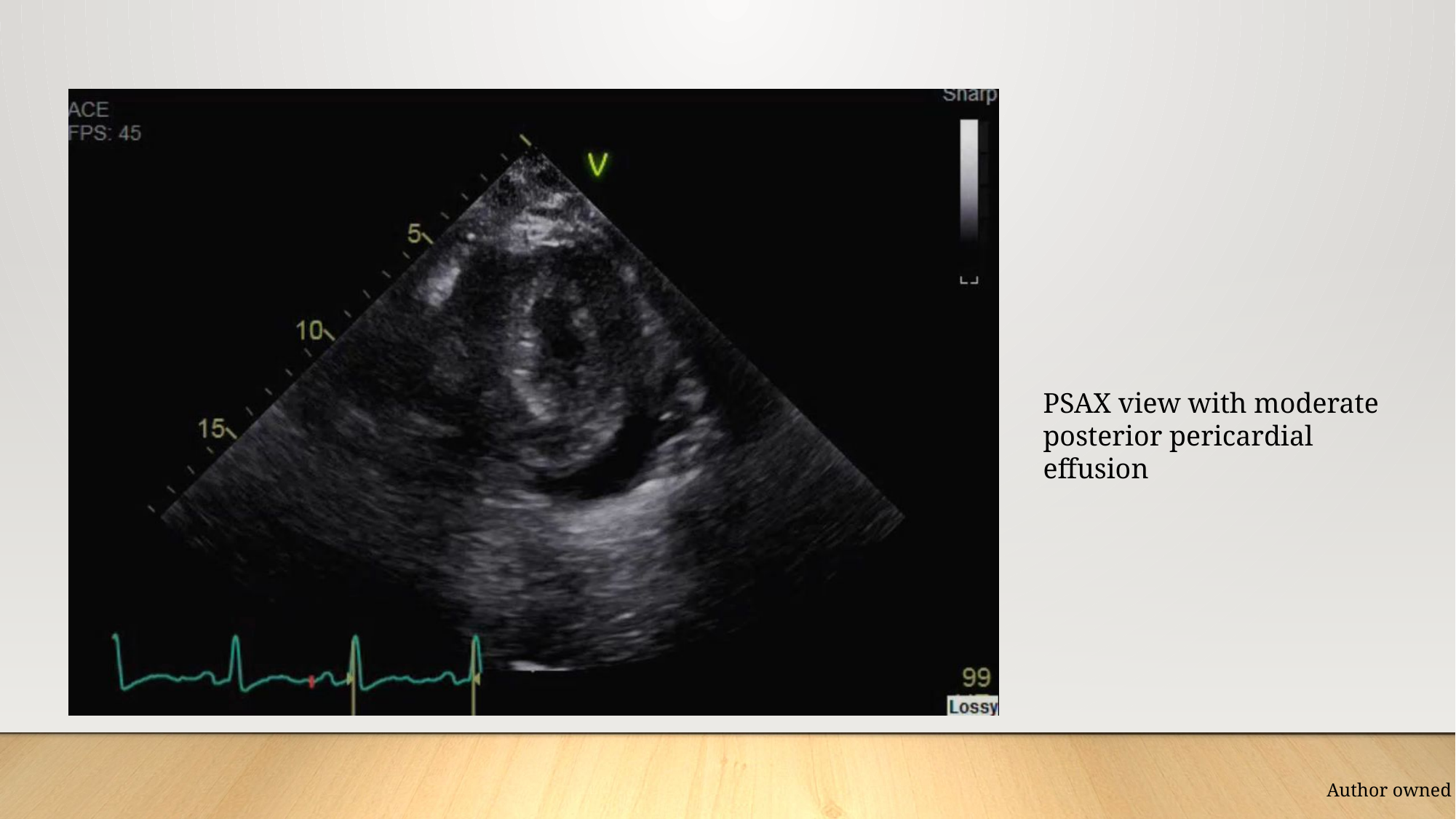

PSAX view with moderate posterior pericardial effusion
Author owned

## Slide 23
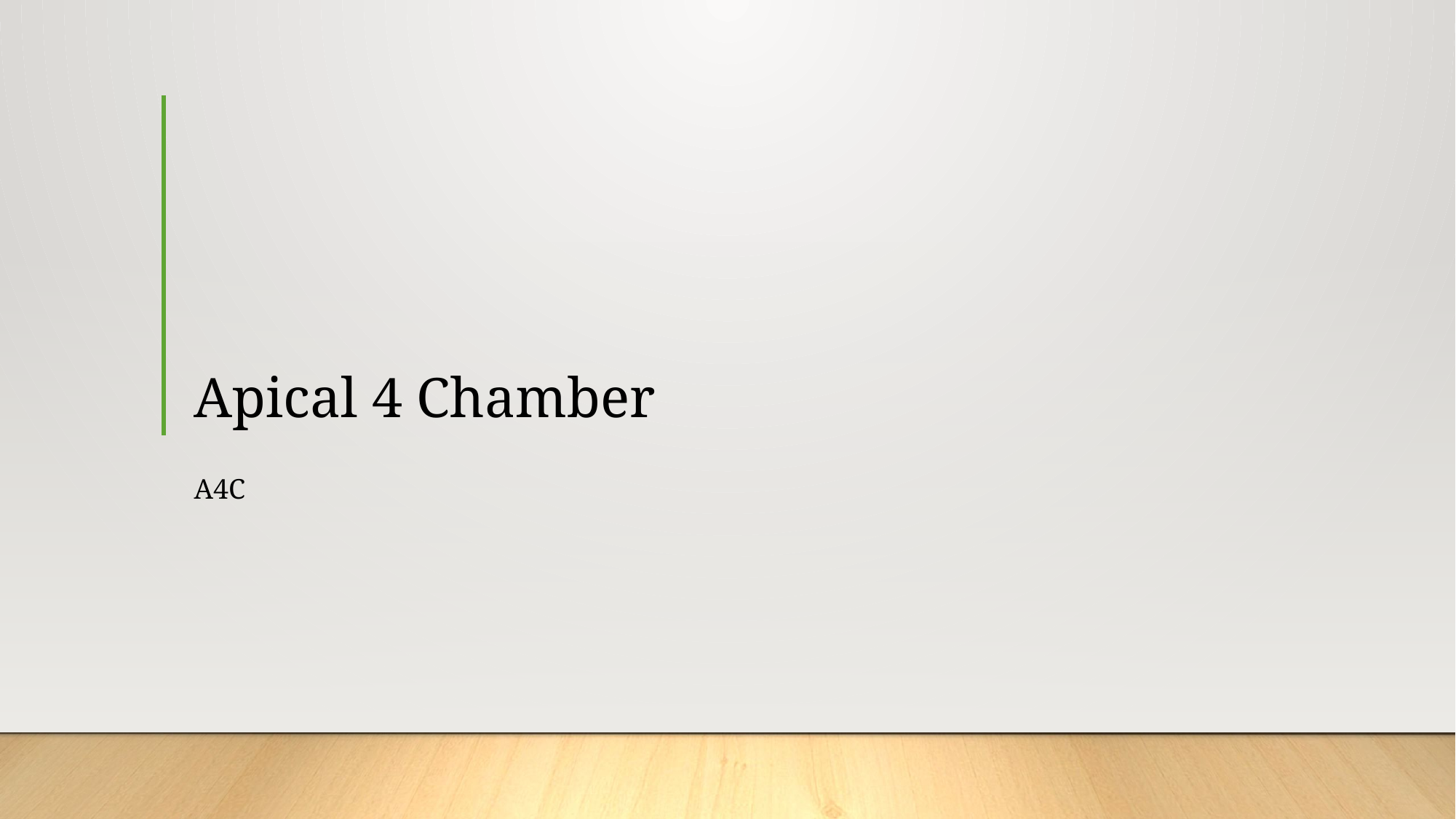

# Apical 4 Chamber
A4C

## Slide 24
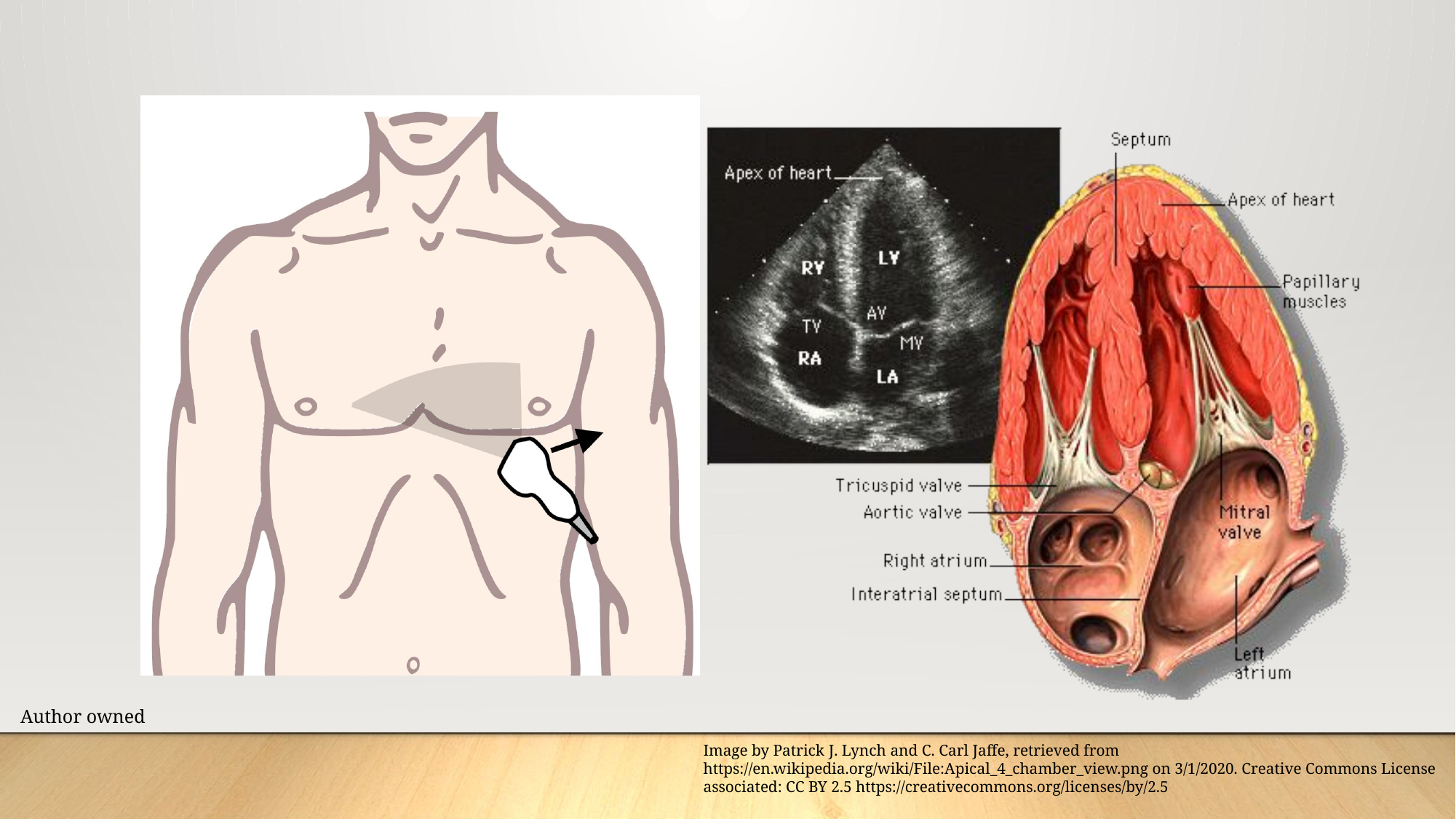

#
Author owned
Image by Patrick J. Lynch and C. Carl Jaffe, retrieved from https://en.wikipedia.org/wiki/File:Apical_4_chamber_view.png on 3/1/2020. Creative Commons License associated: CC BY 2.5 https://creativecommons.org/licenses/by/2.5

## Slide 25
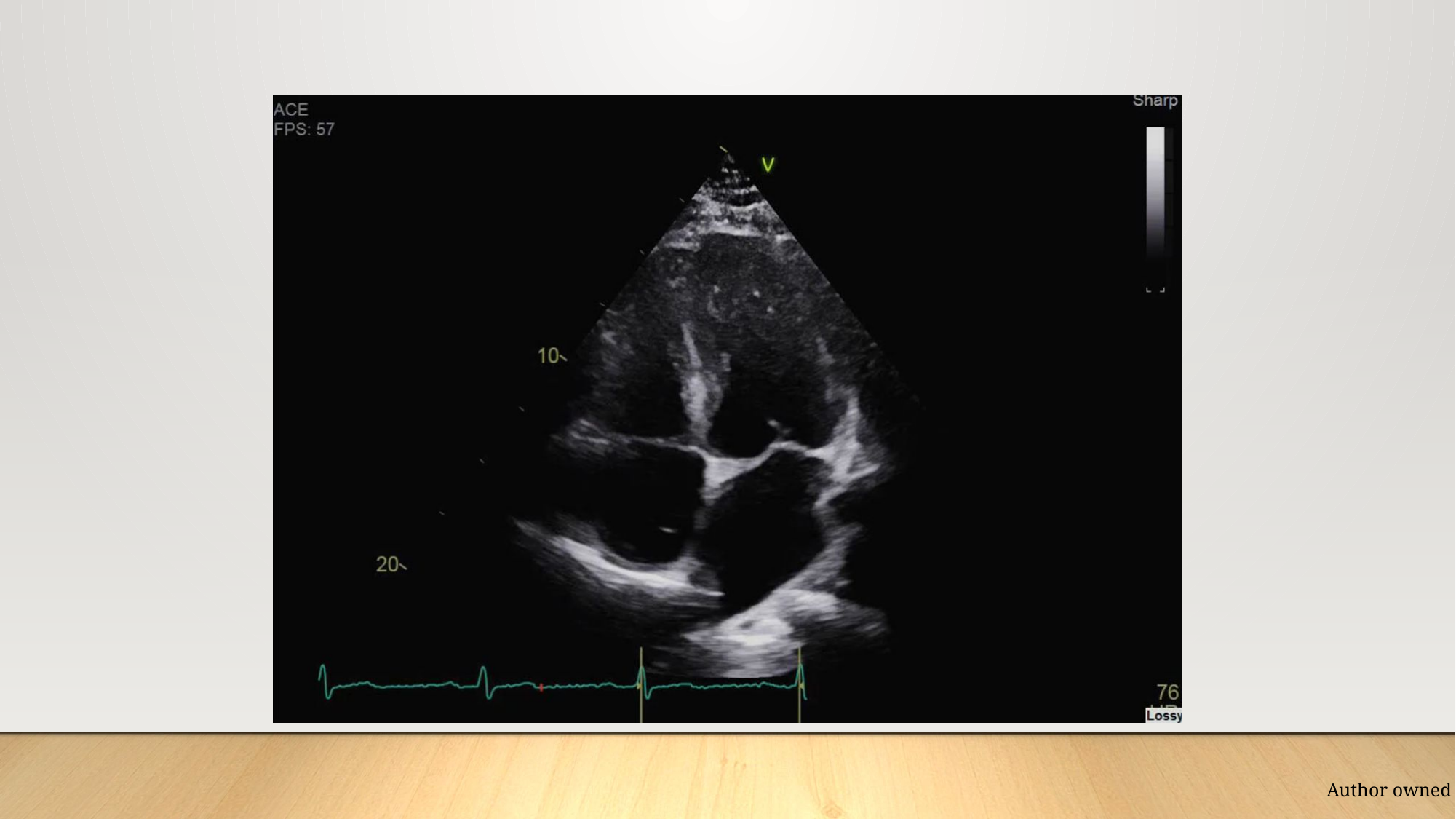

Author owned

## Slide 26
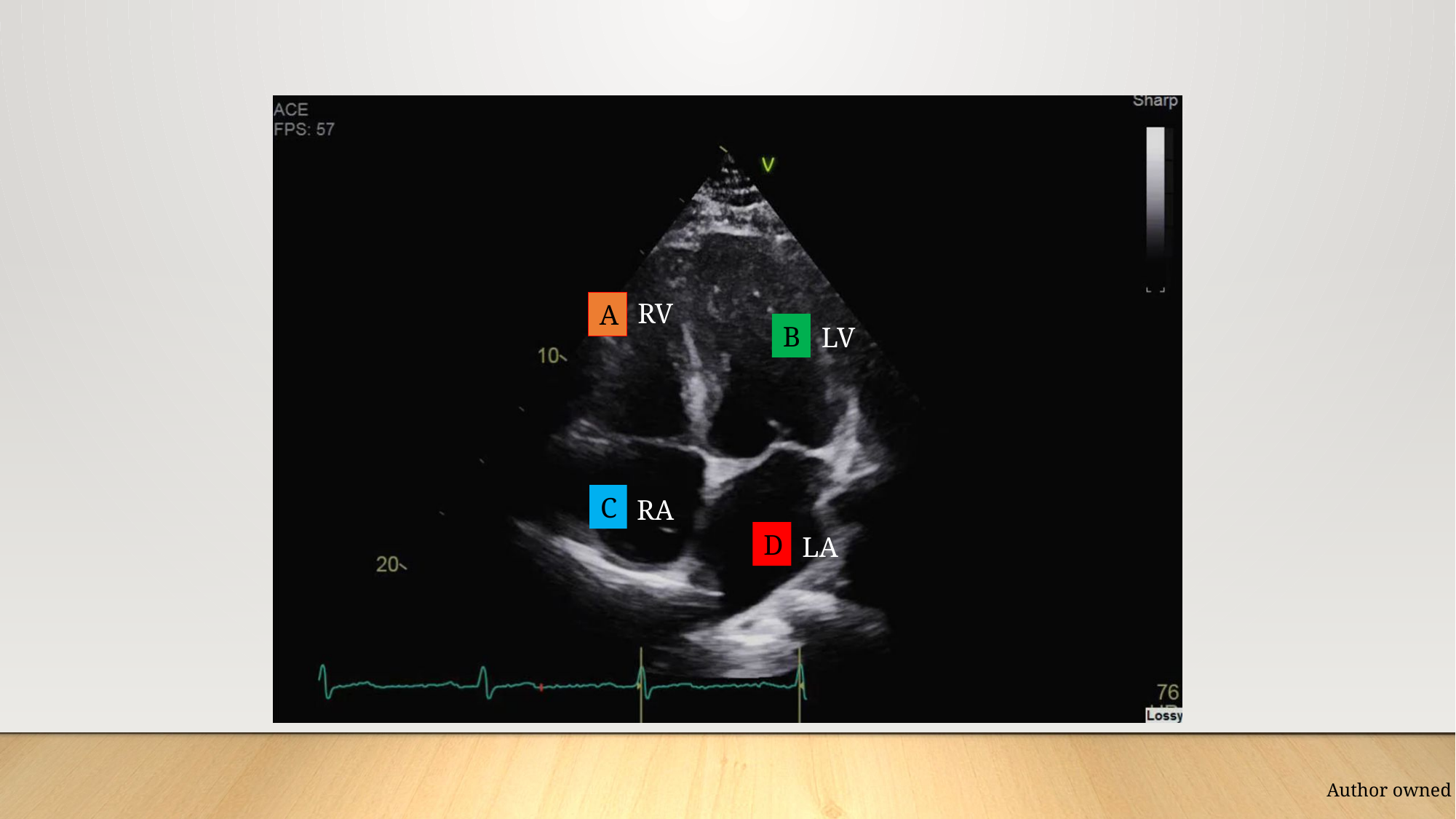

RV
A
B
LV
C
RA
D
LA
Author owned

## Slide 27
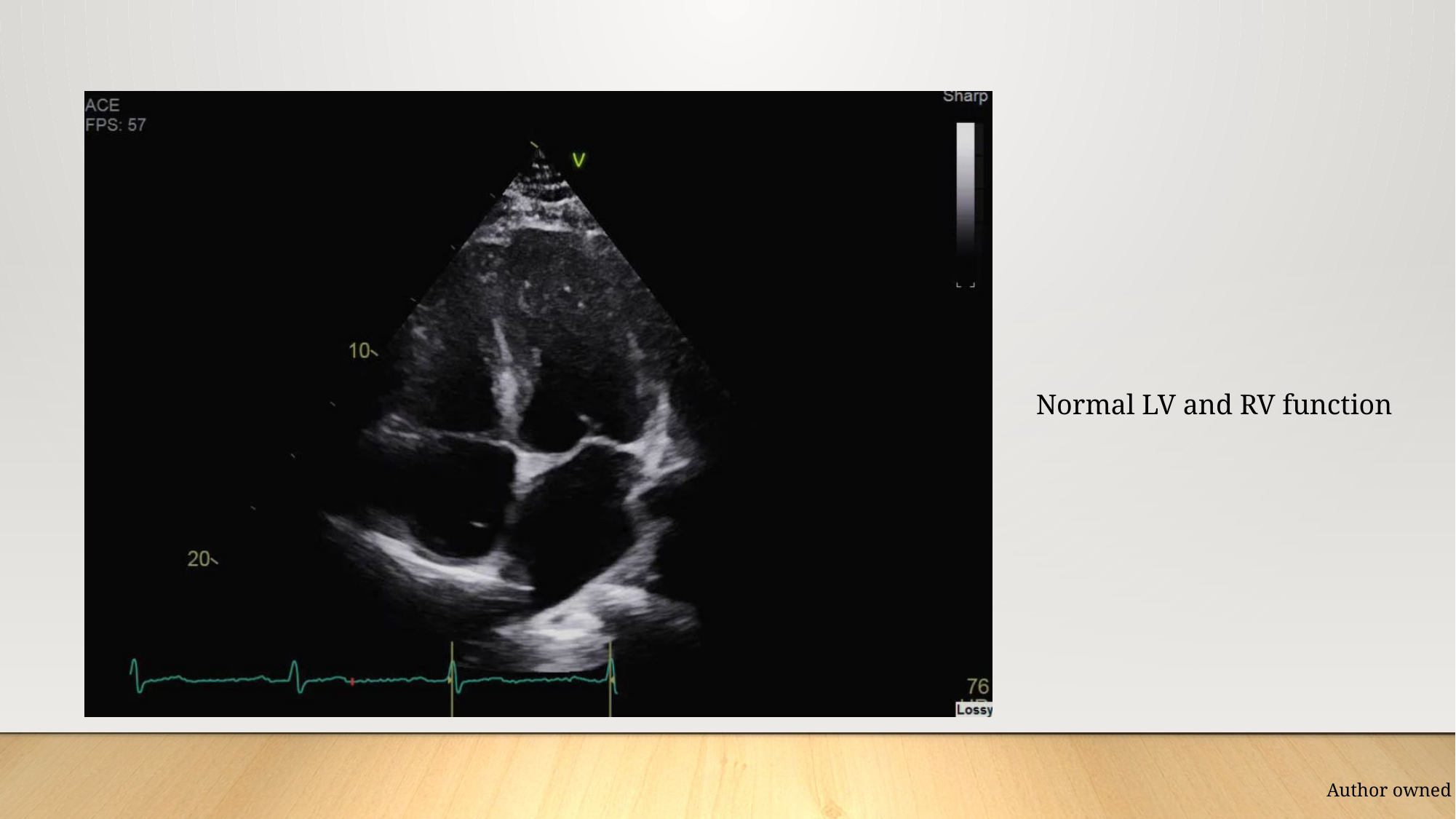

Normal LV and RV function
Author owned

## Slide 28
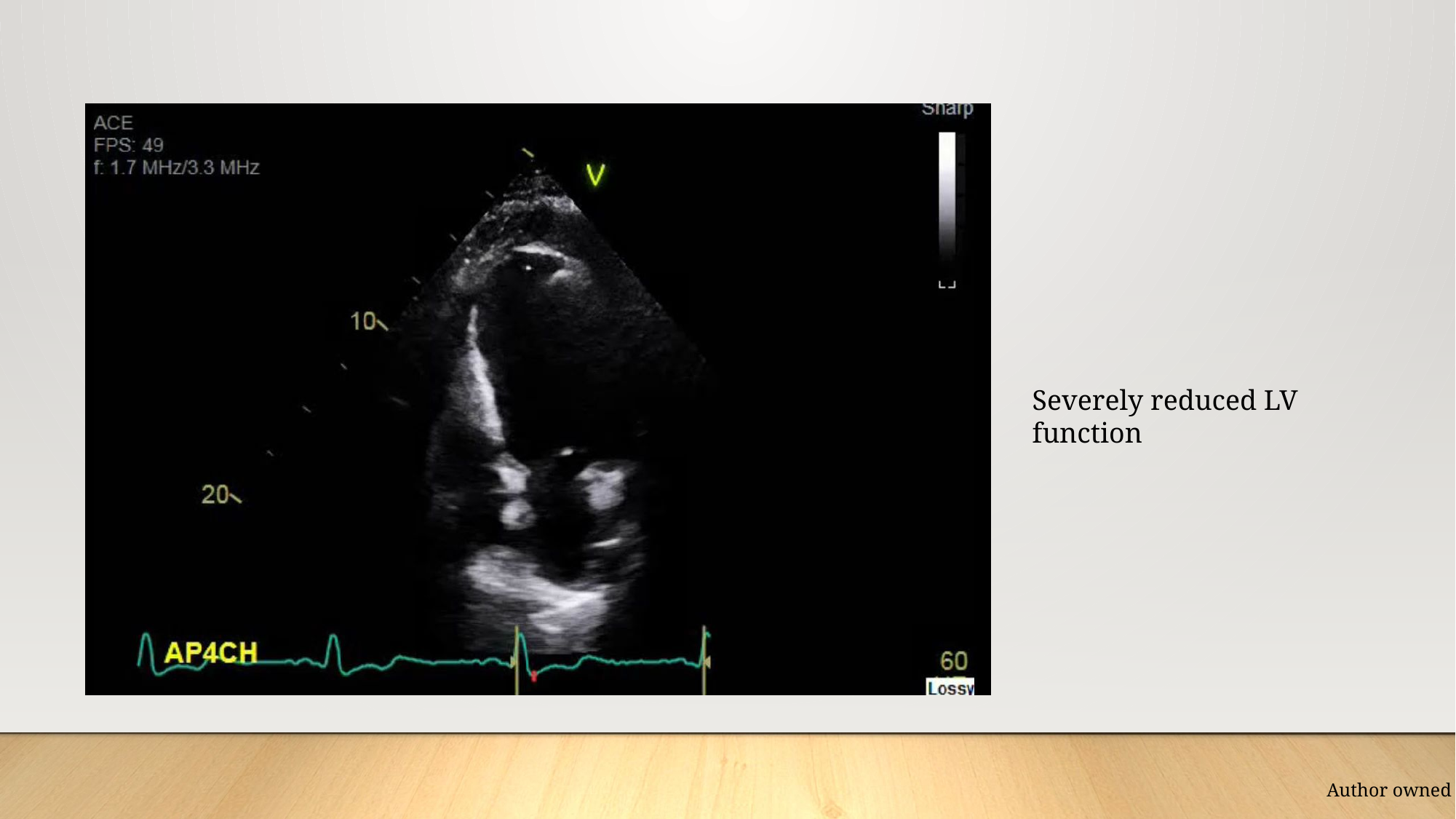

Severely reduced LV function
Author owned

## Slide 29
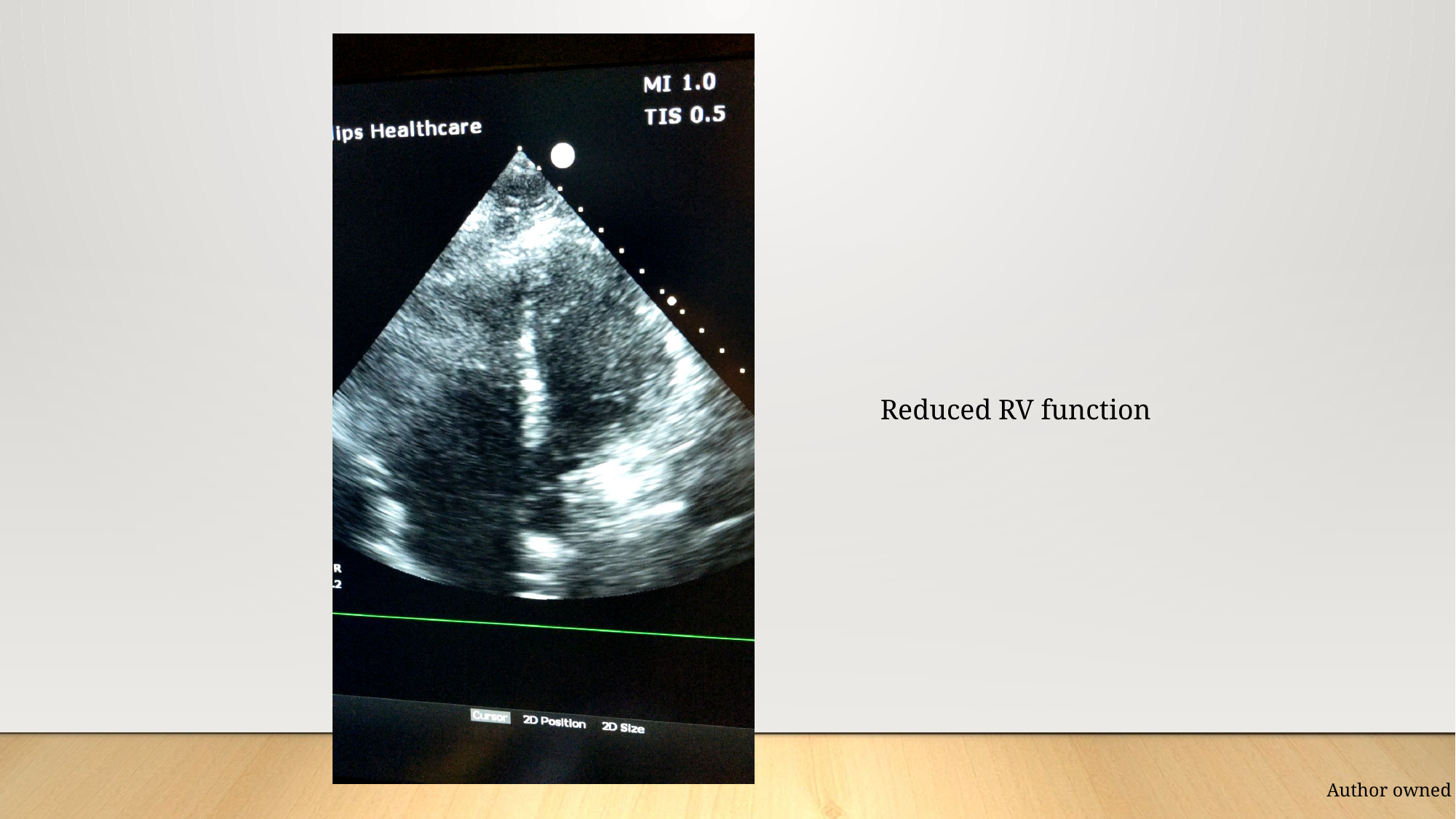

Reduced RV function
Author owned

## Slide 30
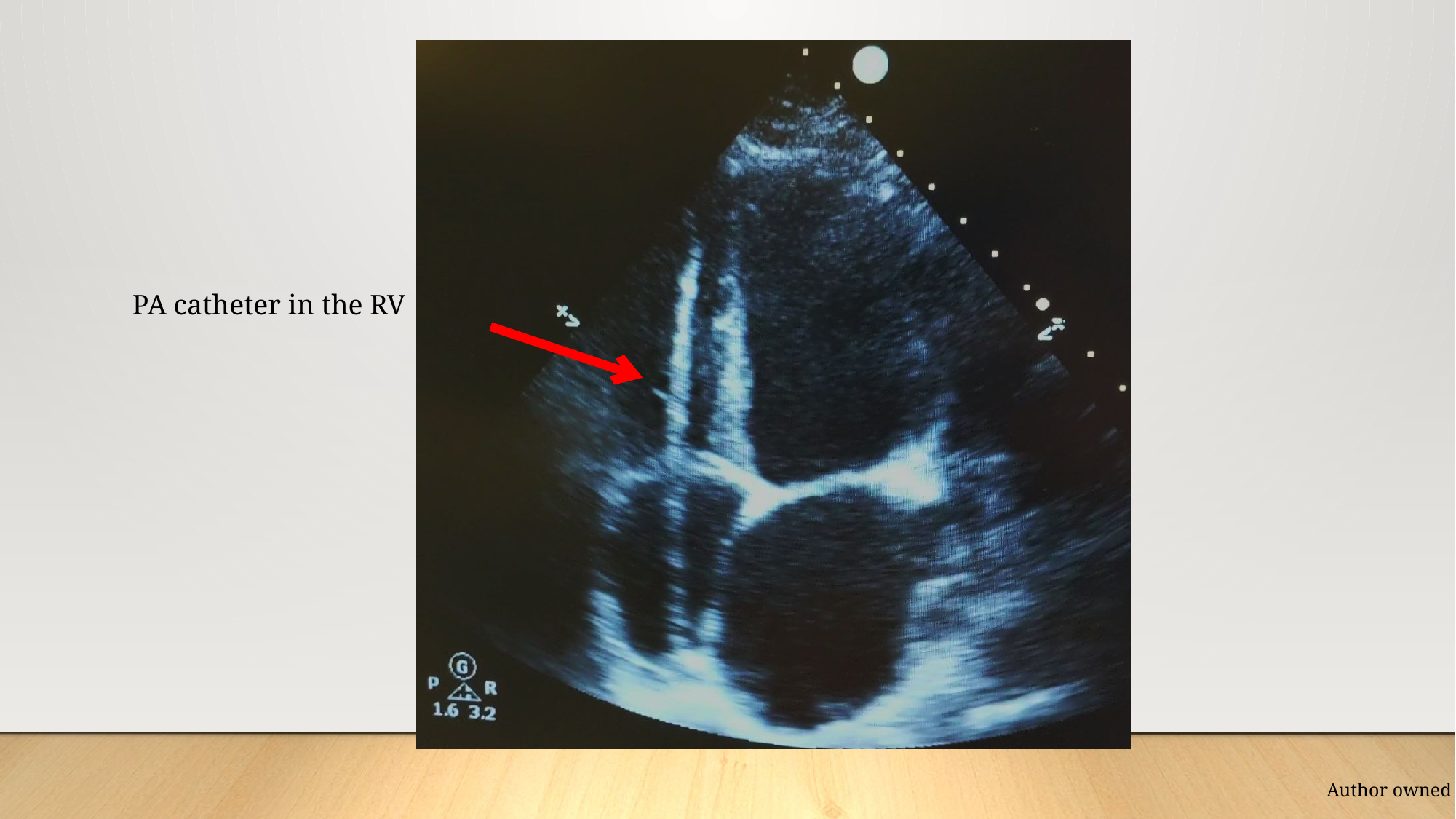

PA catheter in the RV
Author owned

## Slide 31
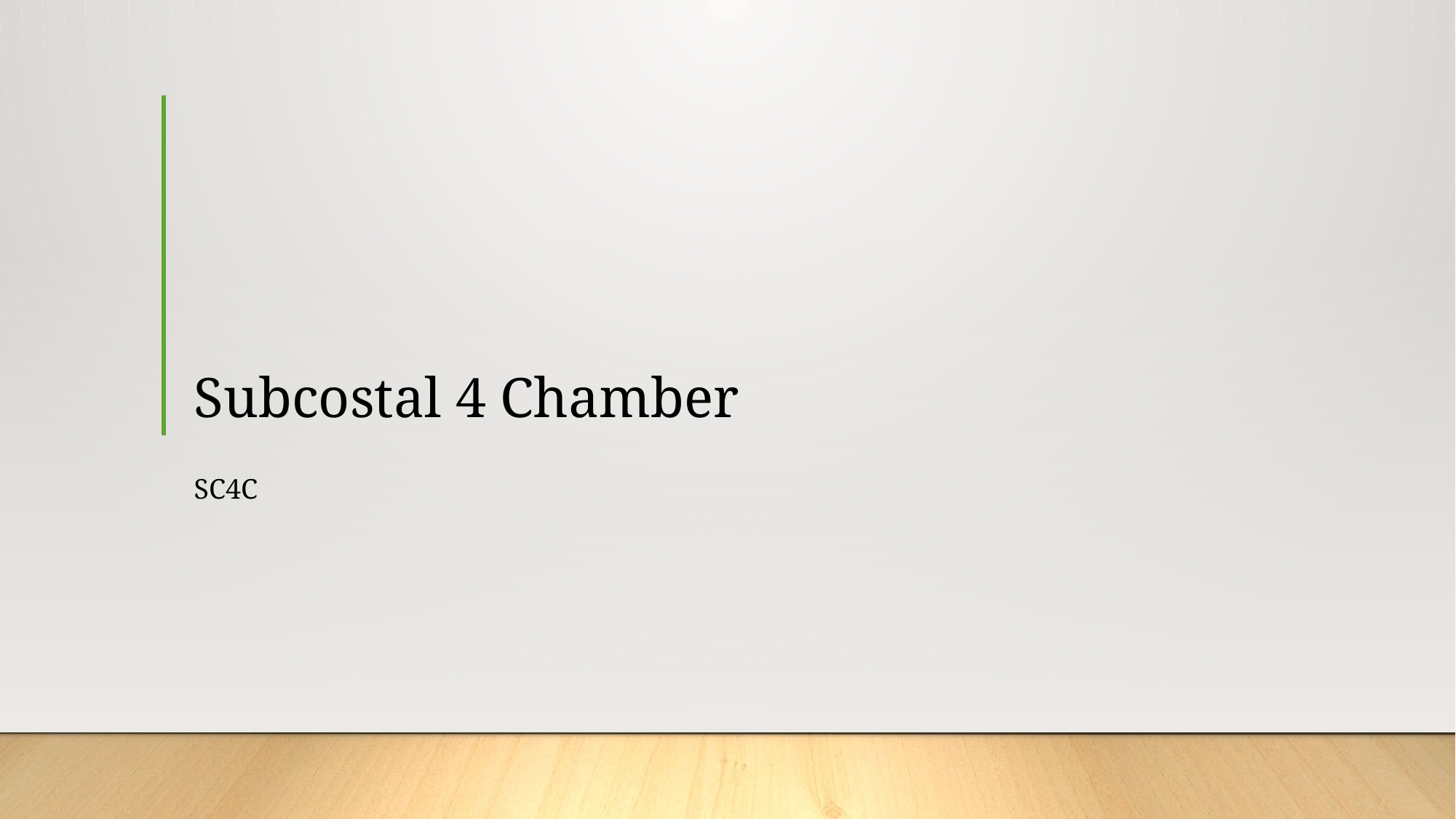

# Subcostal 4 Chamber
SC4C

## Slide 32
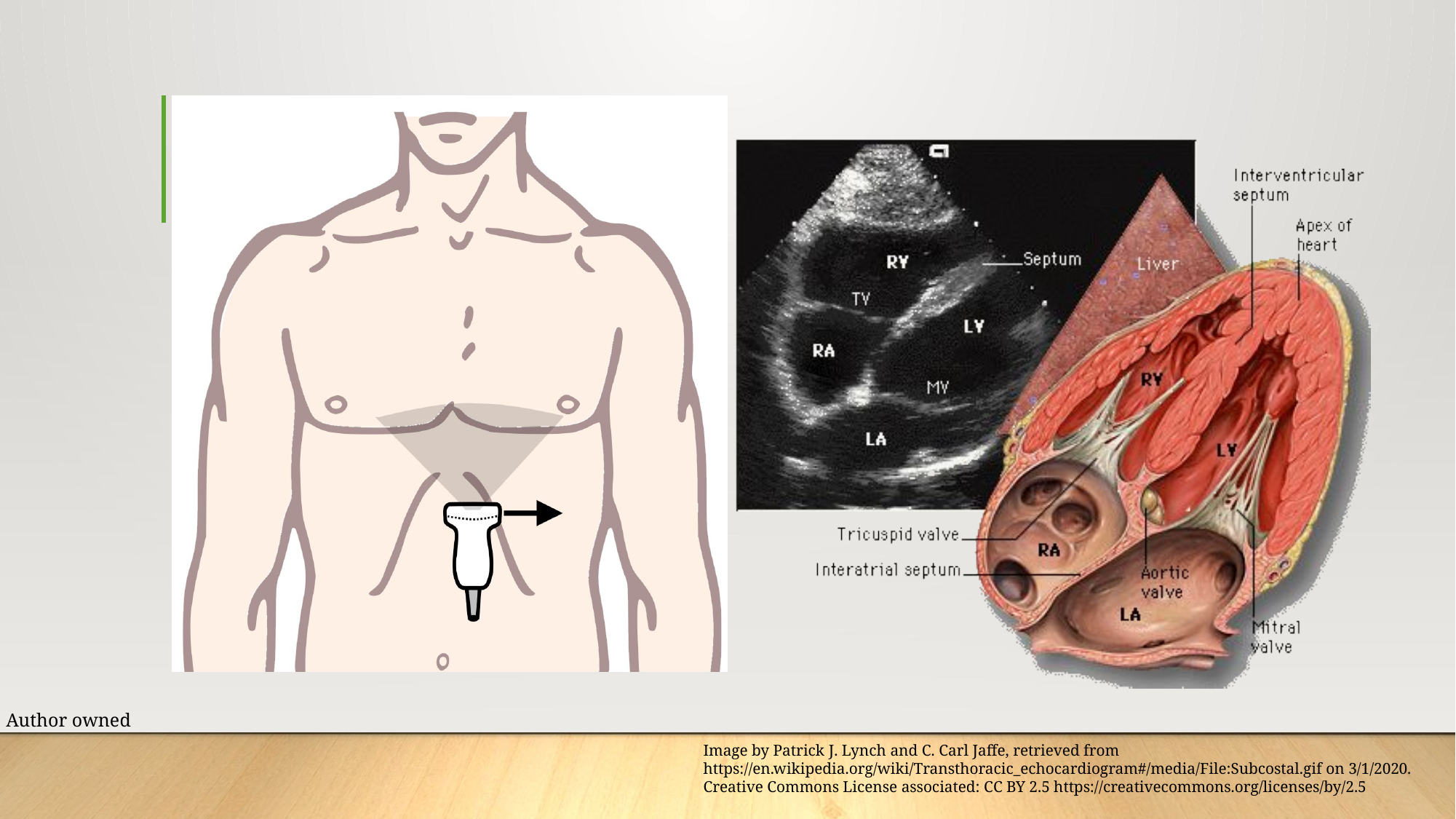

#
Author owned
Image by Patrick J. Lynch and C. Carl Jaffe, retrieved from https://en.wikipedia.org/wiki/Transthoracic_echocardiogram#/media/File:Subcostal.gif on 3/1/2020. Creative Commons License associated: CC BY 2.5 https://creativecommons.org/licenses/by/2.5

## Slide 33
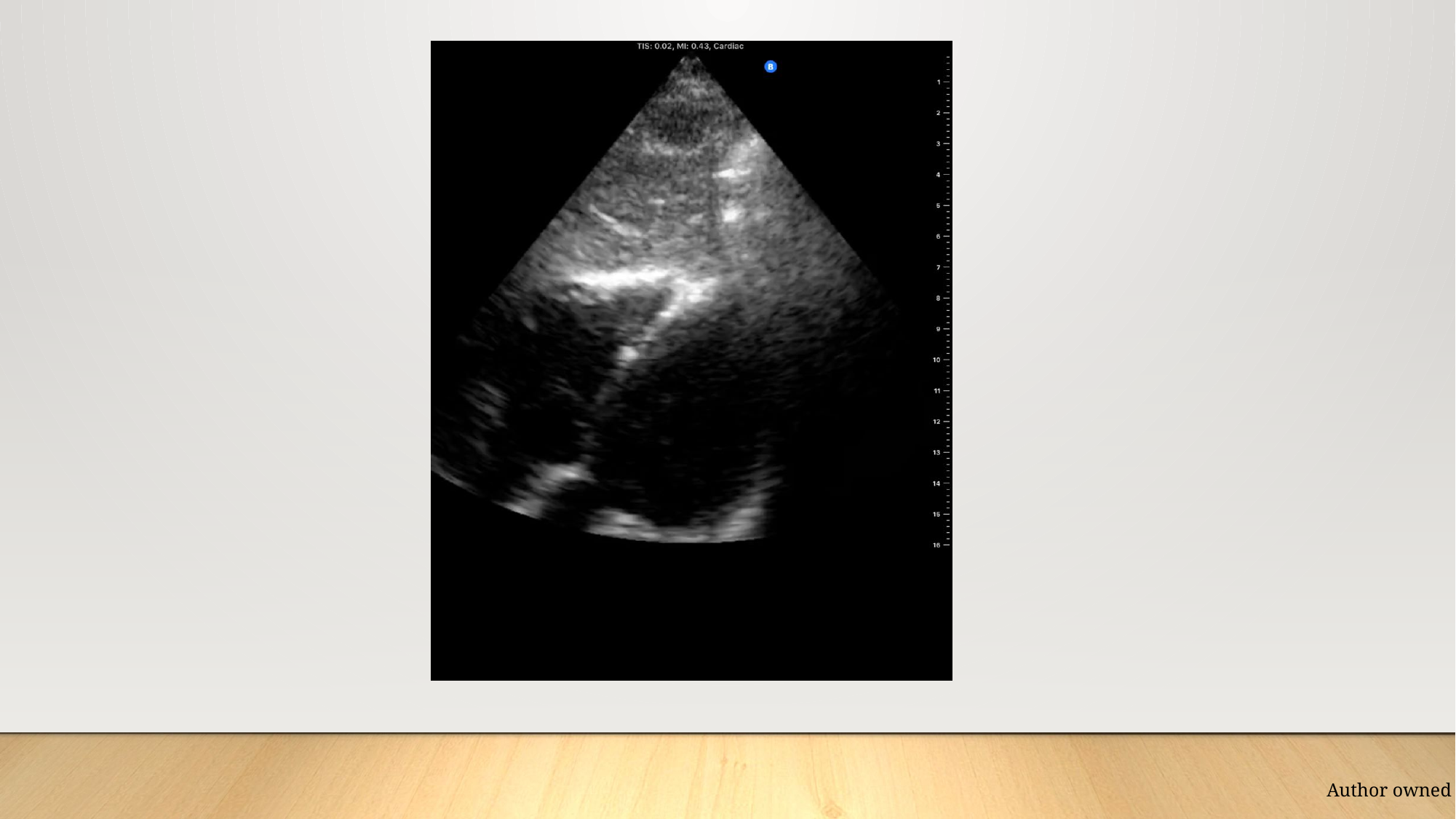

Author owned

## Slide 34
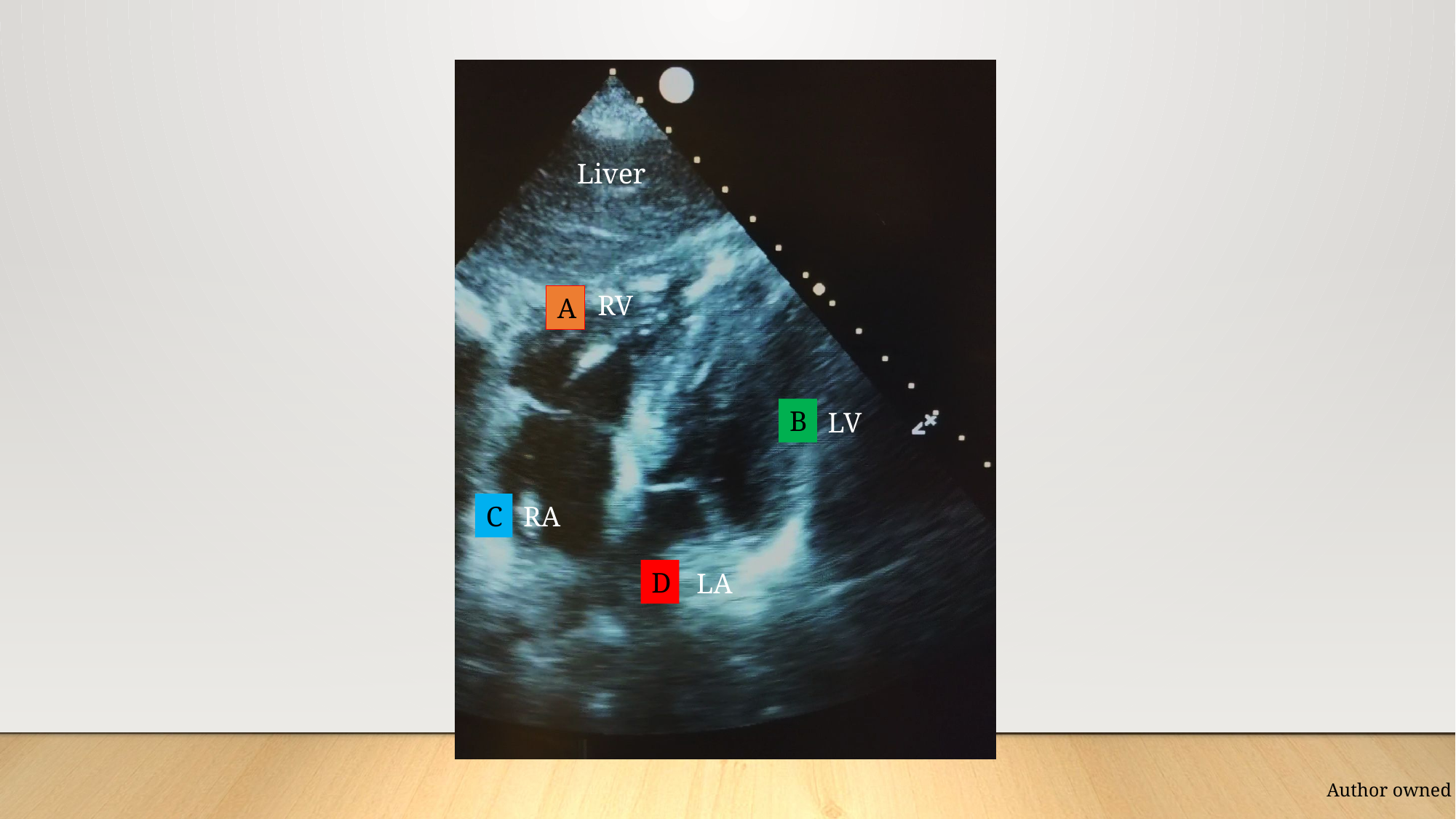

Liver
RV
A
B
LV
C
RA
D
LA
Author owned

## Slide 35
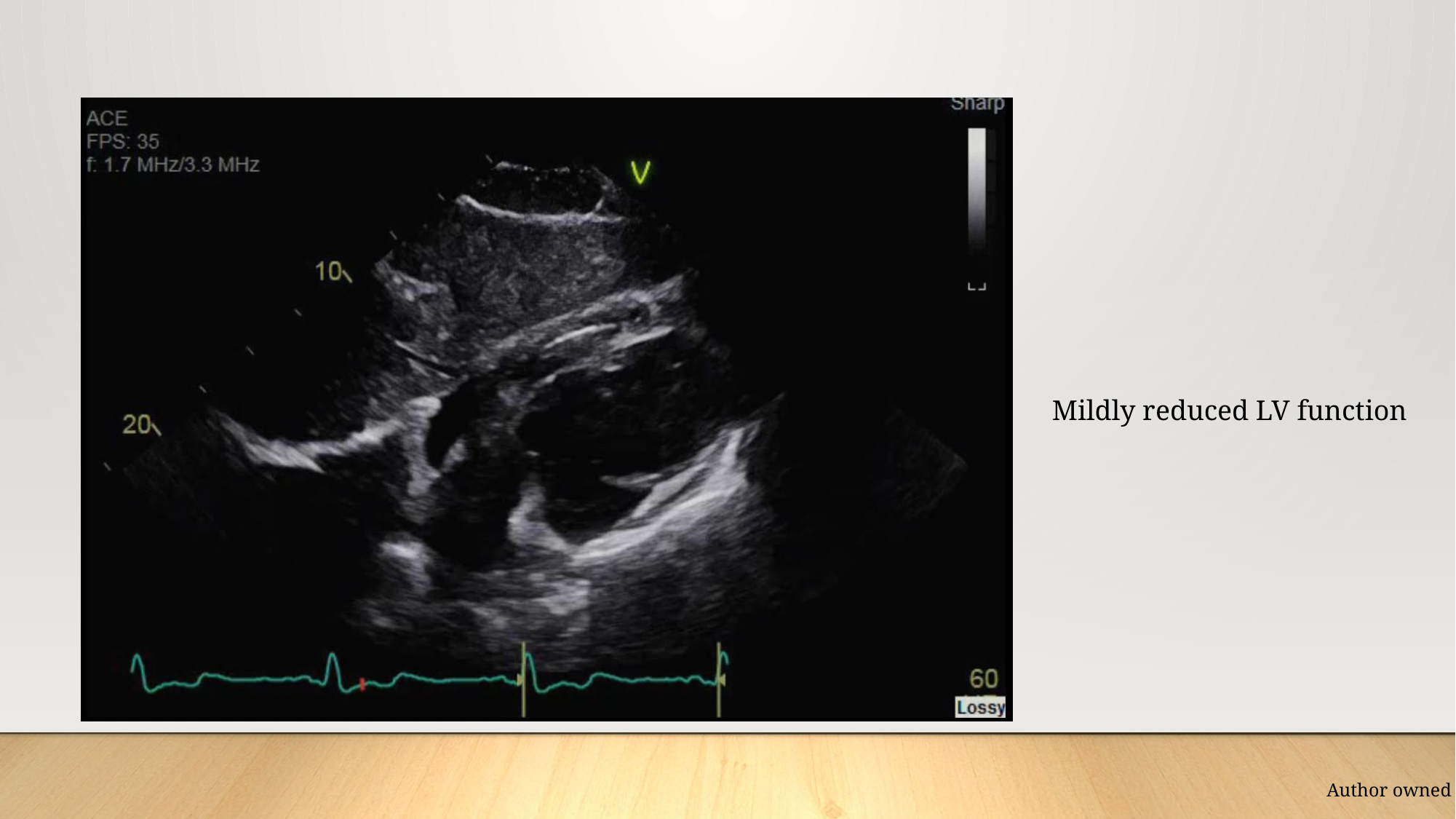

Mildly reduced LV function
Author owned

## Slide 36
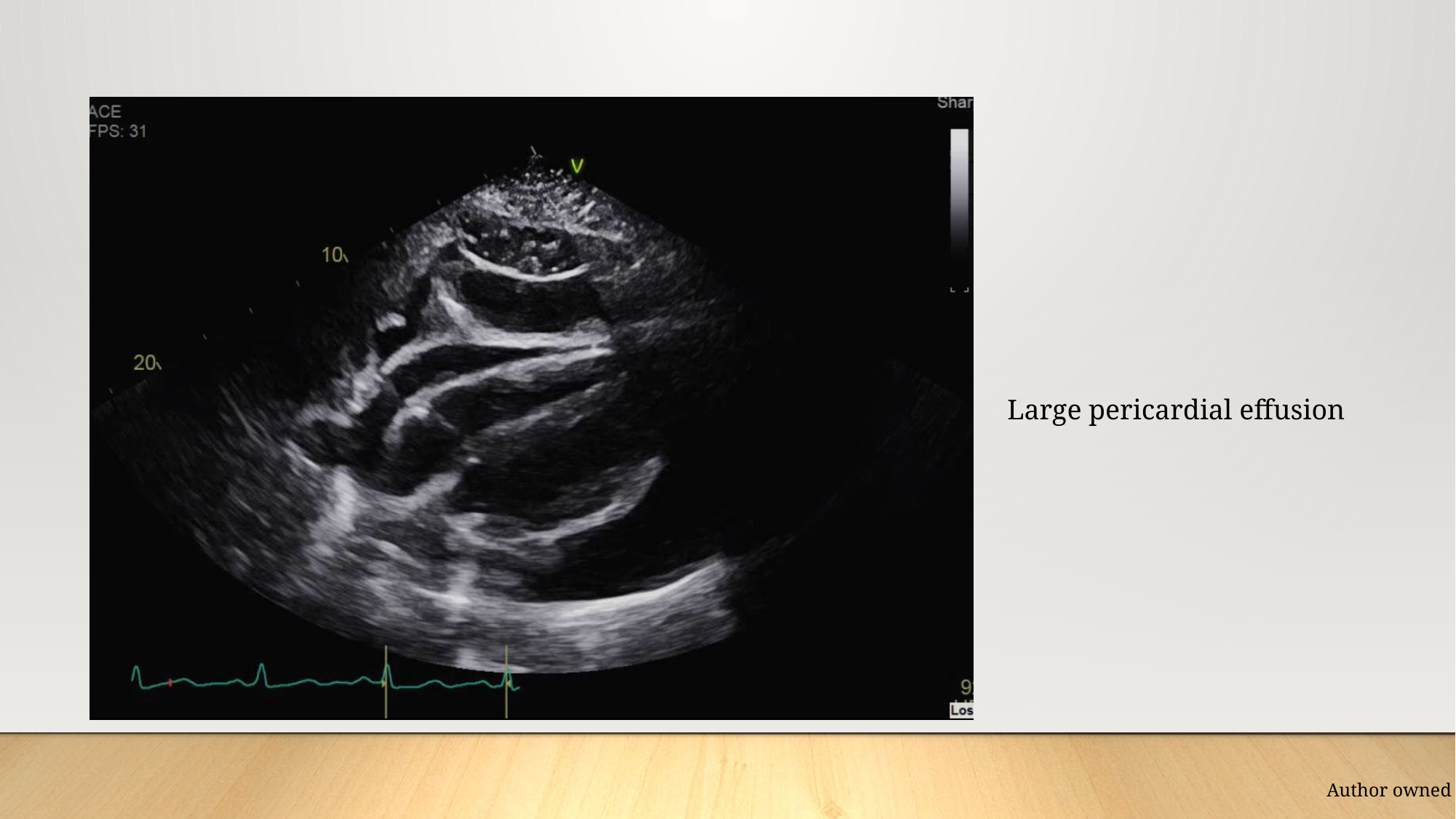

Large pericardial effusion
Author owned

## Slide 37
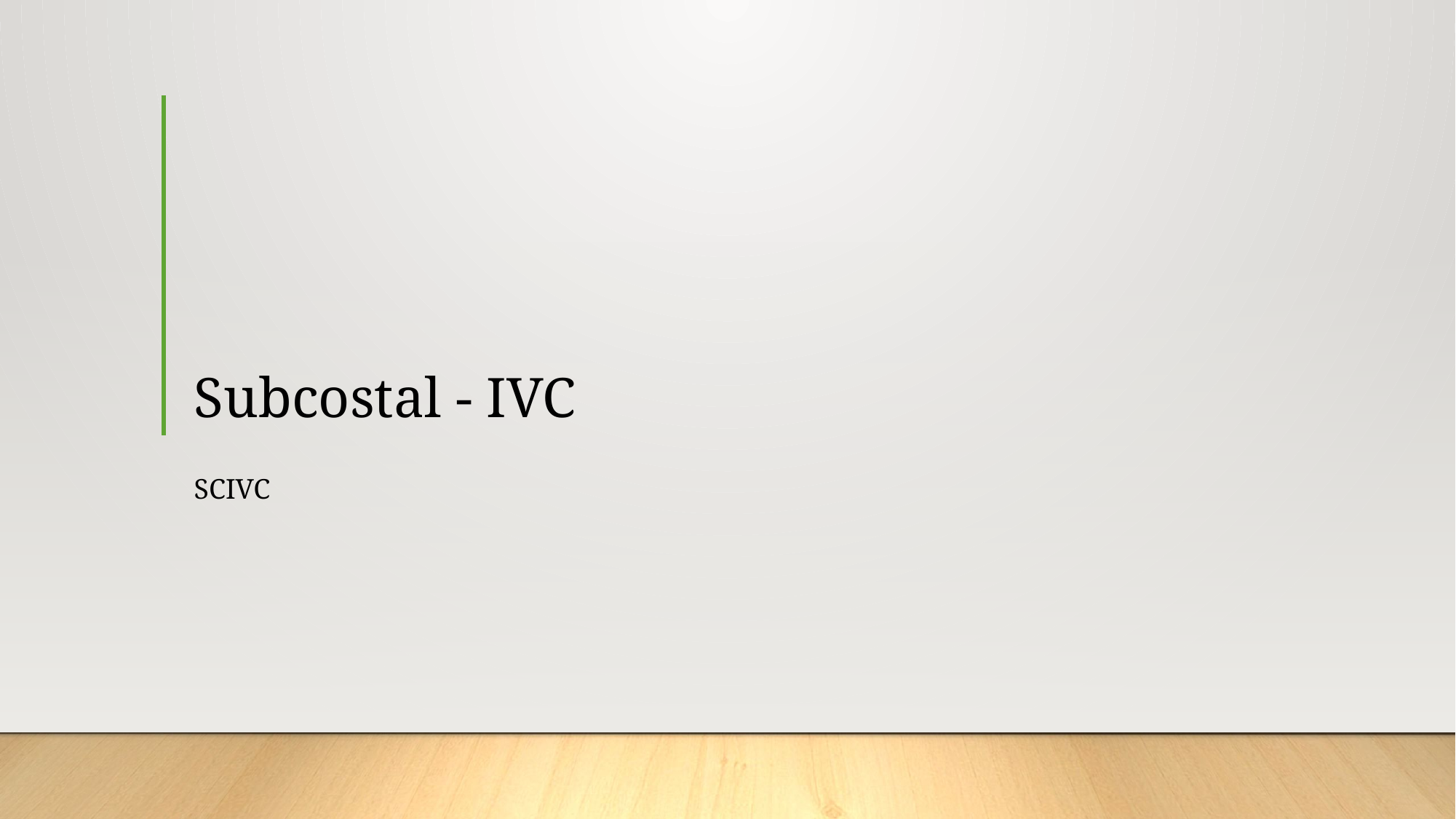

# Subcostal - IVC
SCIVC

## Slide 38
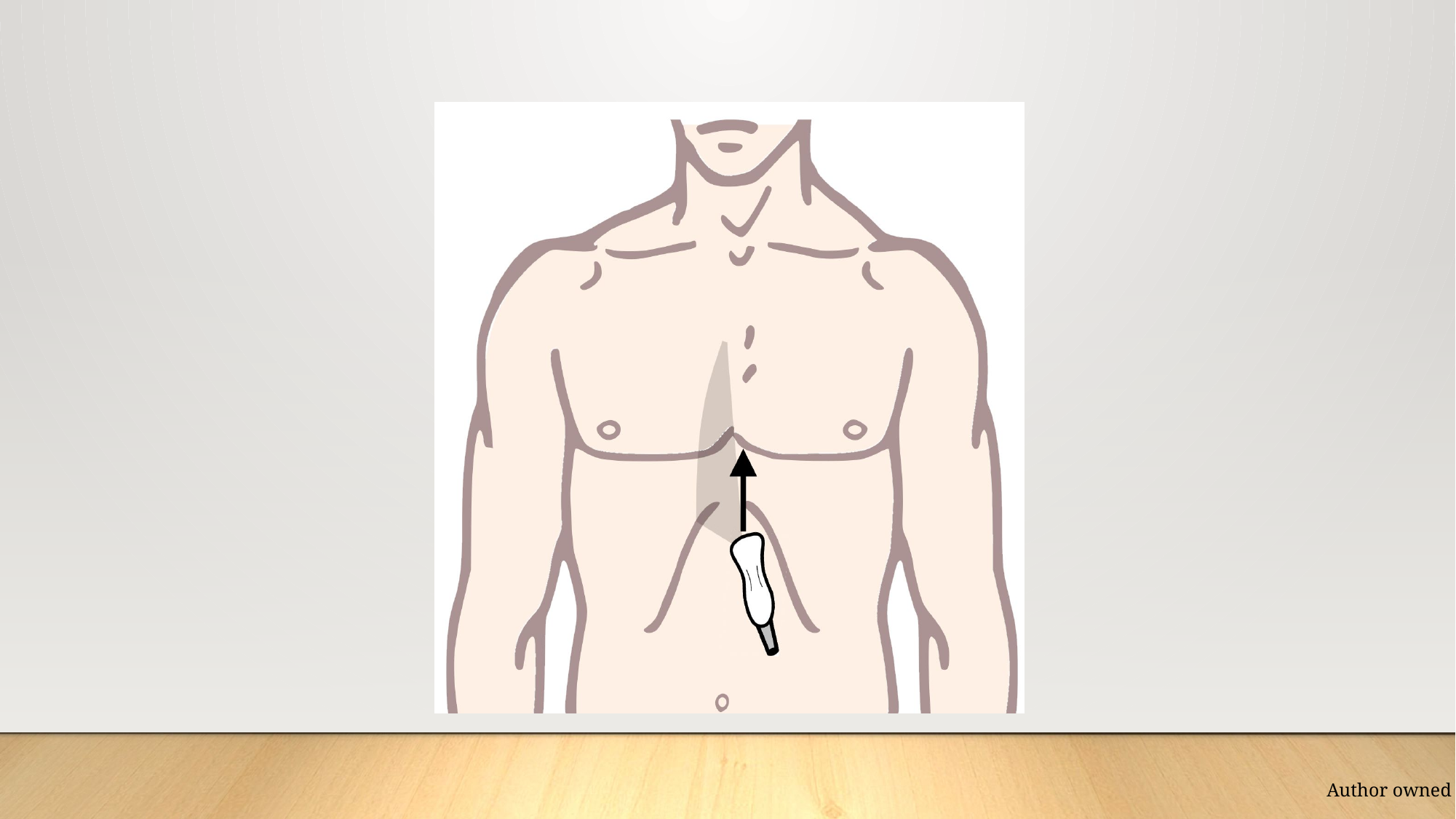

Author owned

## Slide 39
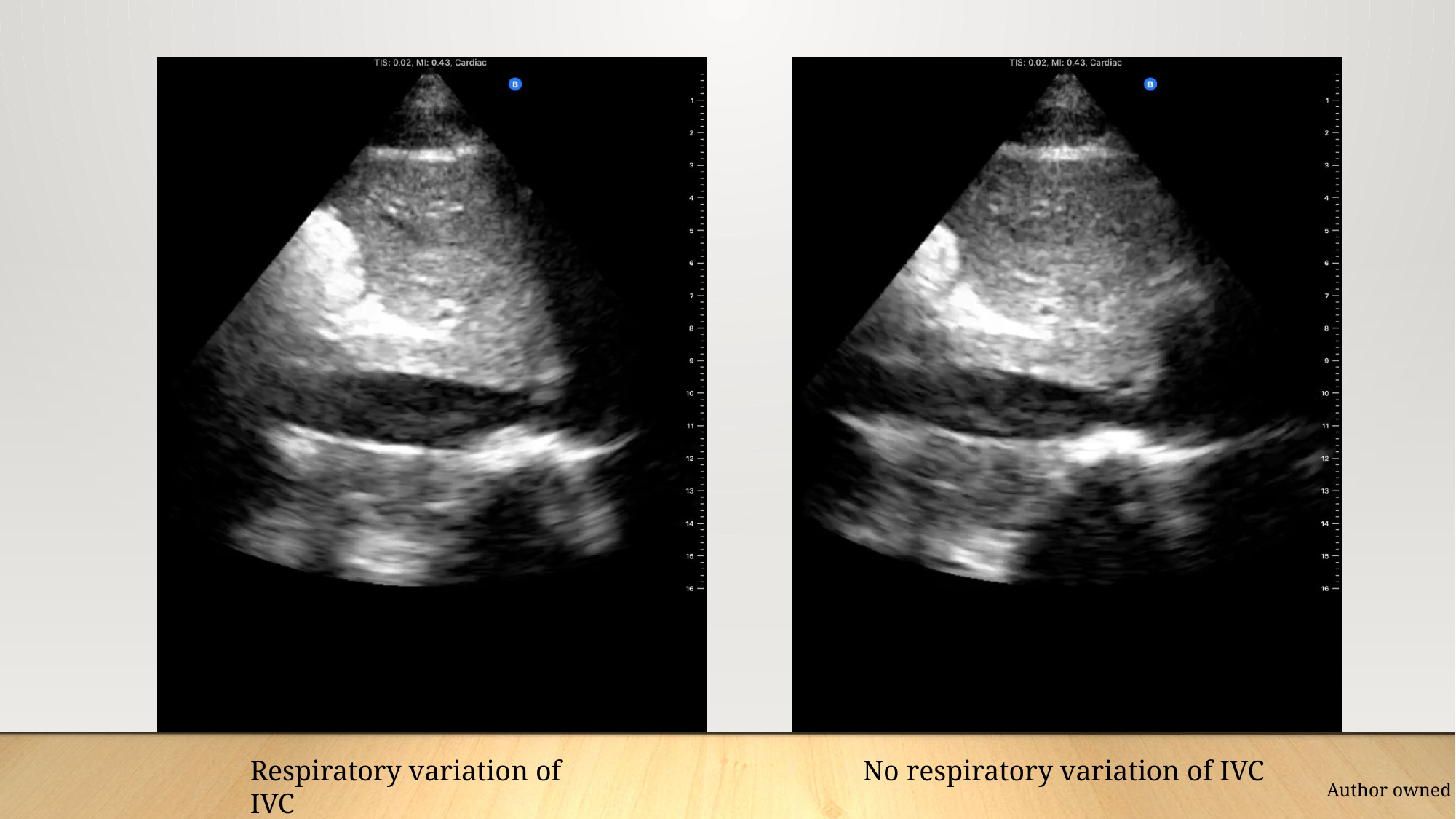

Respiratory variation of IVC
No respiratory variation of IVC
Author owned

## Slide 40
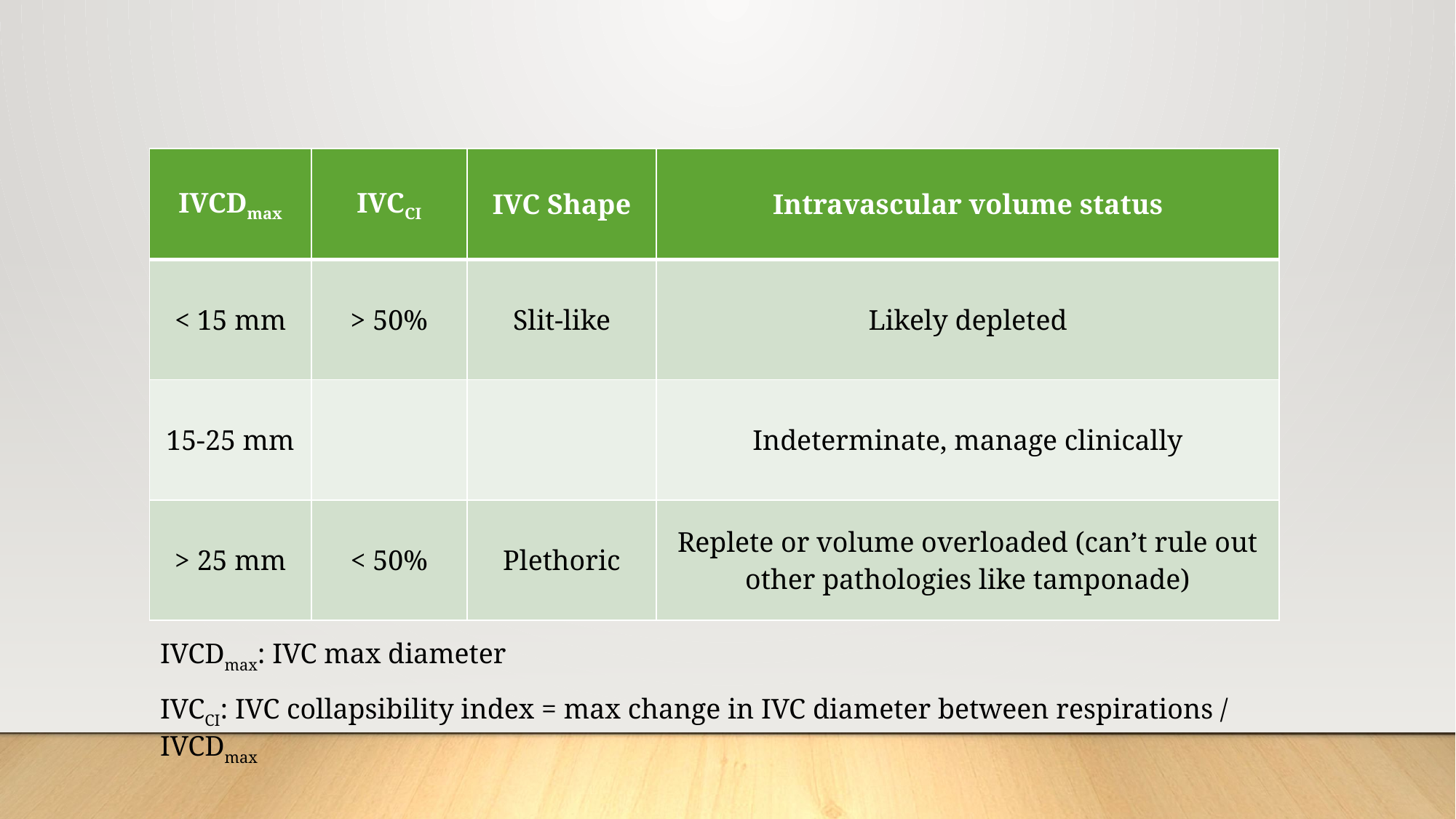

| IVCDmax | IVCCI | IVC Shape | Intravascular volume status |
| --- | --- | --- | --- |
| < 15 mm | > 50% | Slit-like | Likely depleted |
| 15-25 mm | | | Indeterminate, manage clinically |
| > 25 mm | < 50% | Plethoric | Replete or volume overloaded (can’t rule out other pathologies like tamponade) |
IVCDmax: IVC max diameter
IVCCI: IVC collapsibility index = max change in IVC diameter between respirations / IVCDmax

## Slide 41
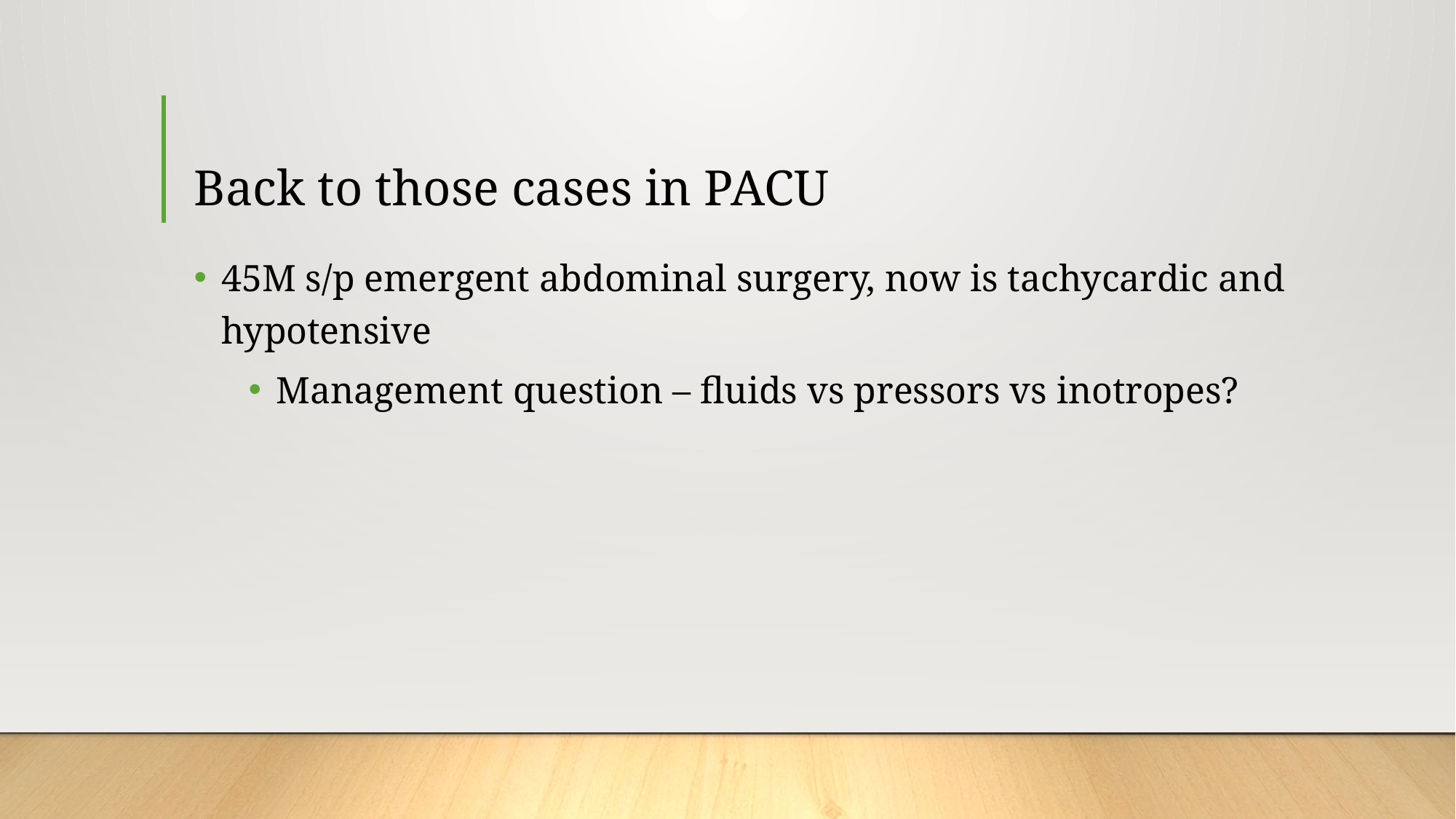

# Back to those cases in PACU
45M s/p emergent abdominal surgery, now is tachycardic and hypotensive
Management question – fluids vs pressors vs inotropes?

## Slide 42
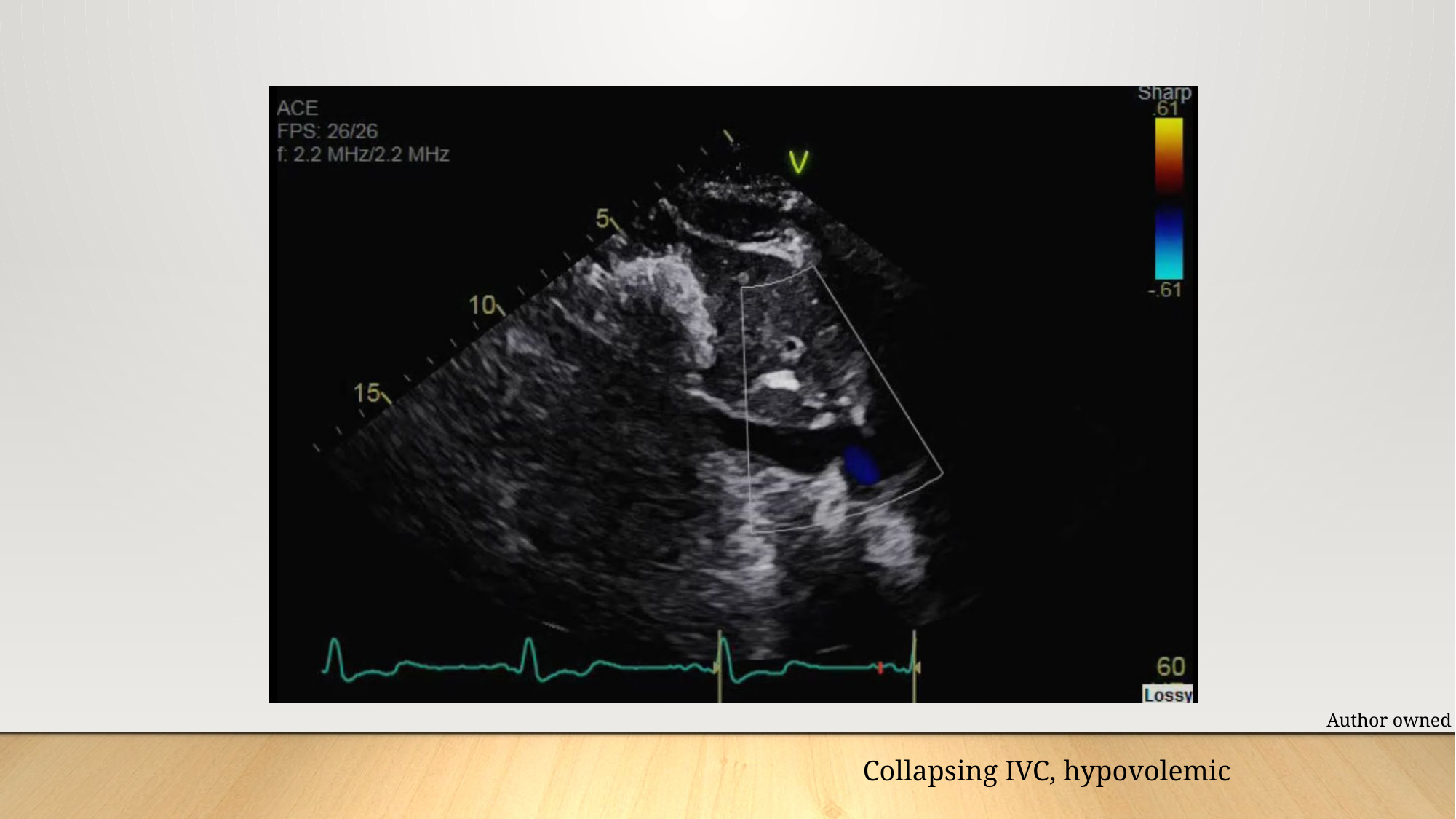

Author owned
Collapsing IVC, hypovolemic

## Slide 43
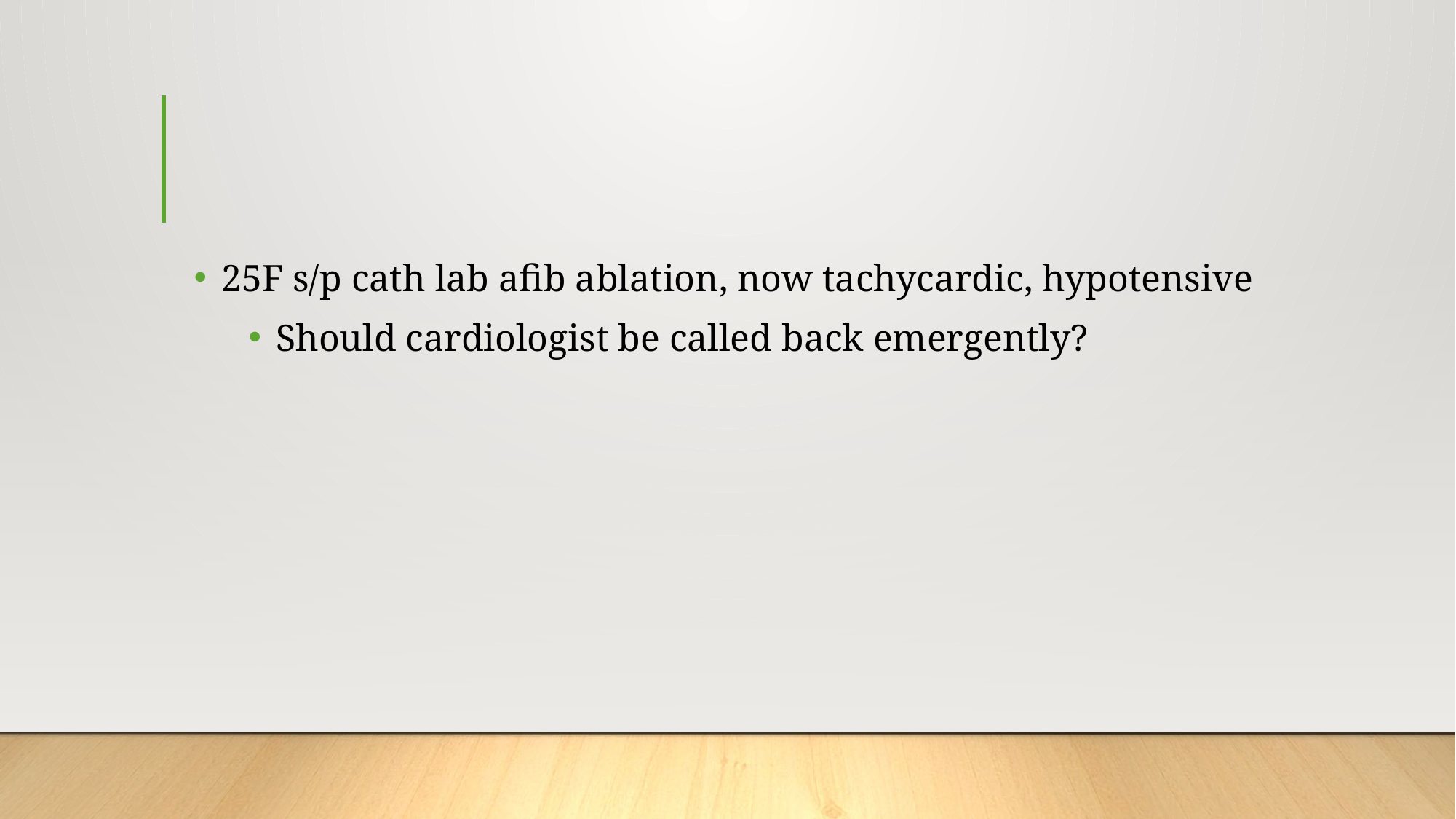

#
25F s/p cath lab afib ablation, now tachycardic, hypotensive
Should cardiologist be called back emergently?

## Slide 44
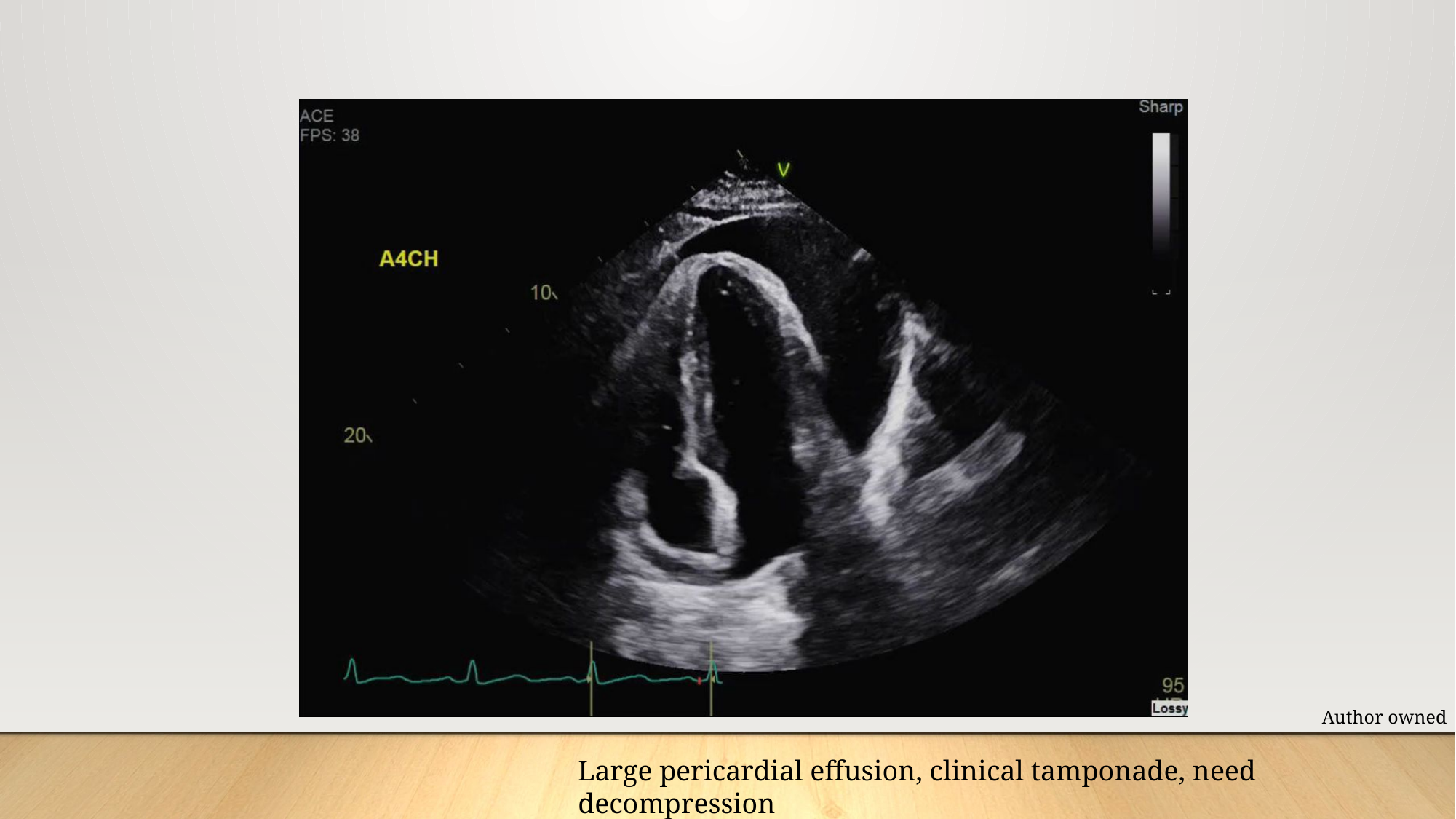

Author owned
Large pericardial effusion, clinical tamponade, need decompression

## Slide 45
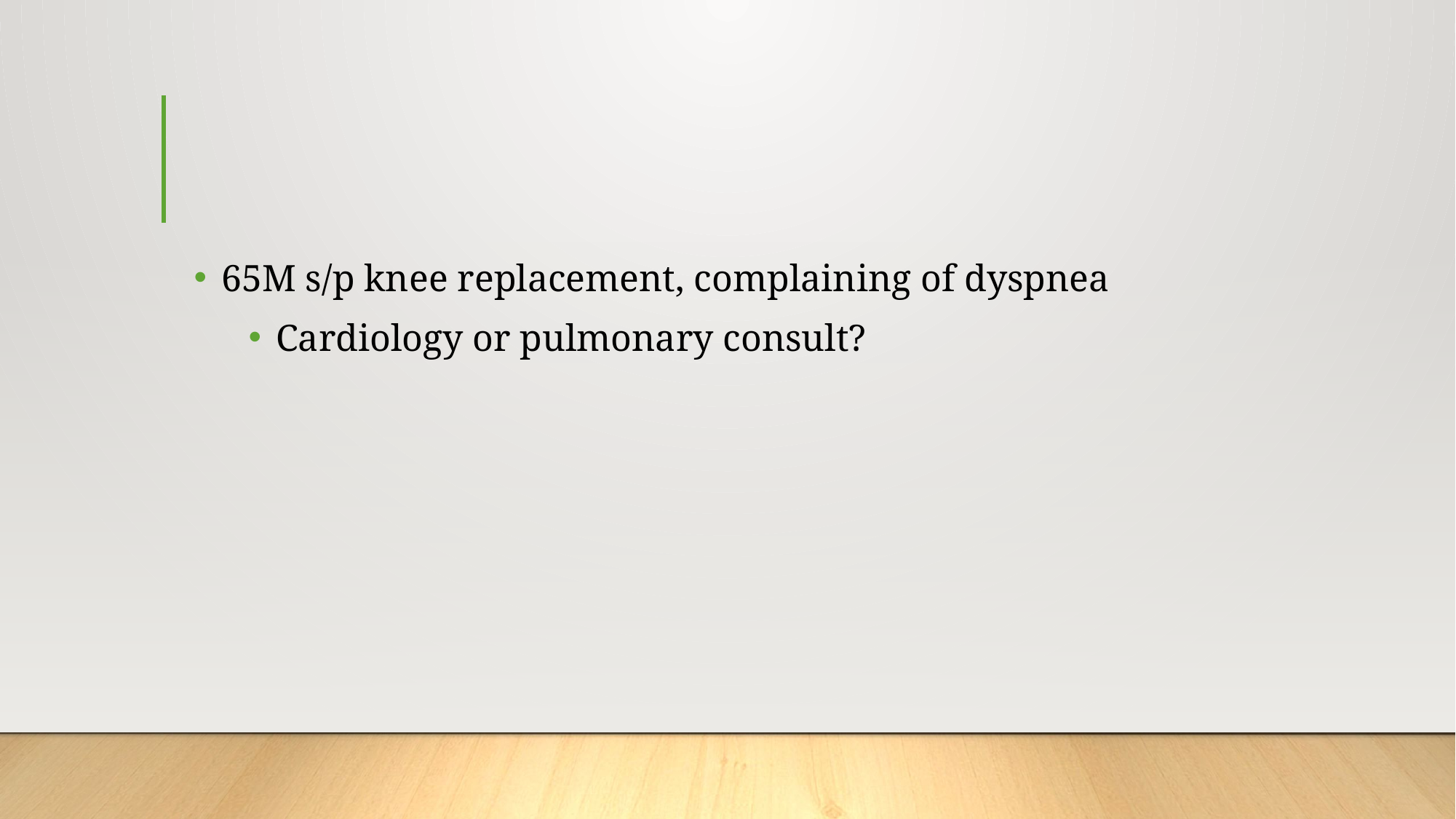

#
65M s/p knee replacement, complaining of dyspnea
Cardiology or pulmonary consult?

## Slide 46
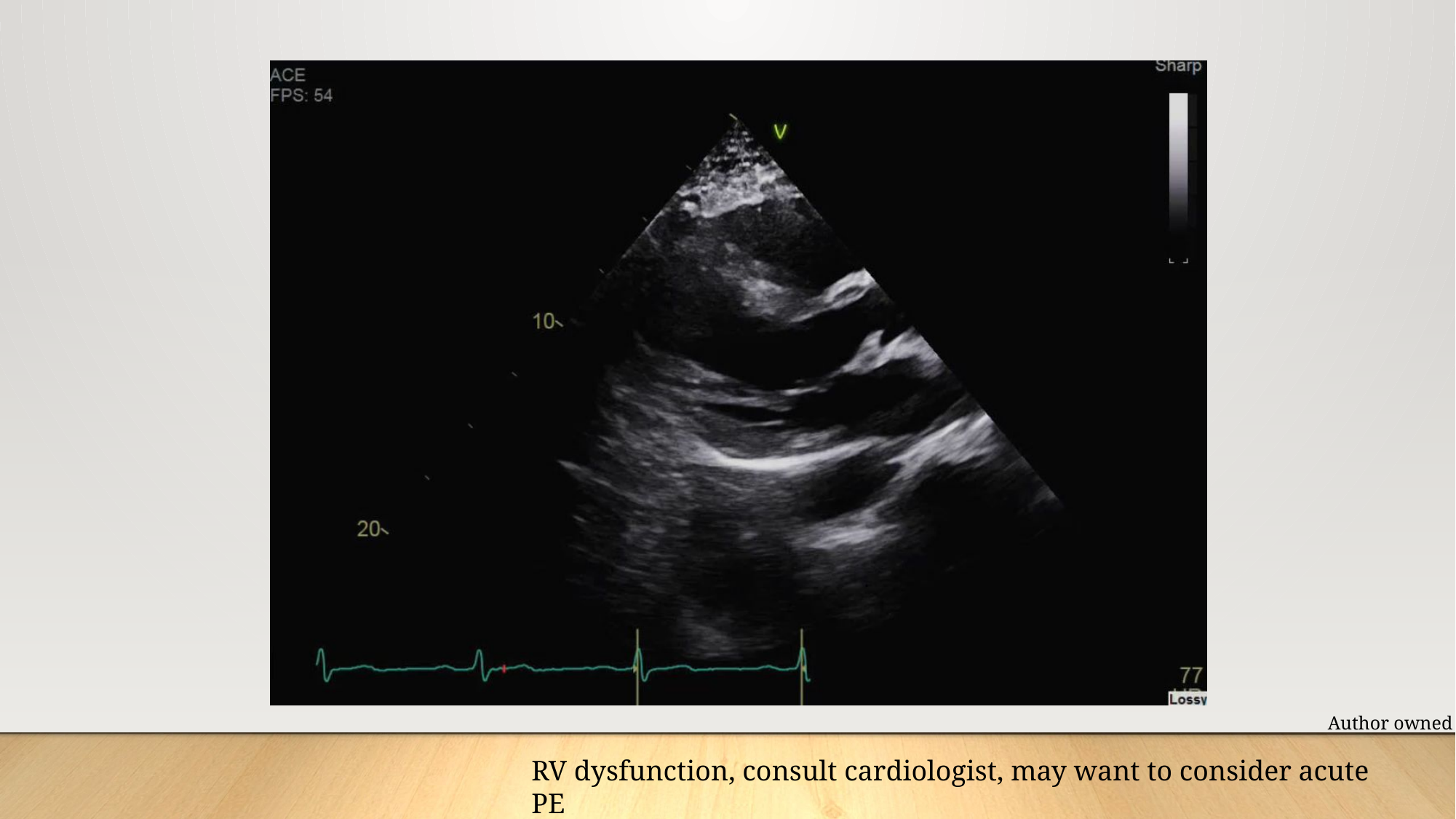

Author owned
RV dysfunction, consult cardiologist, may want to consider acute PE

## Slide 47
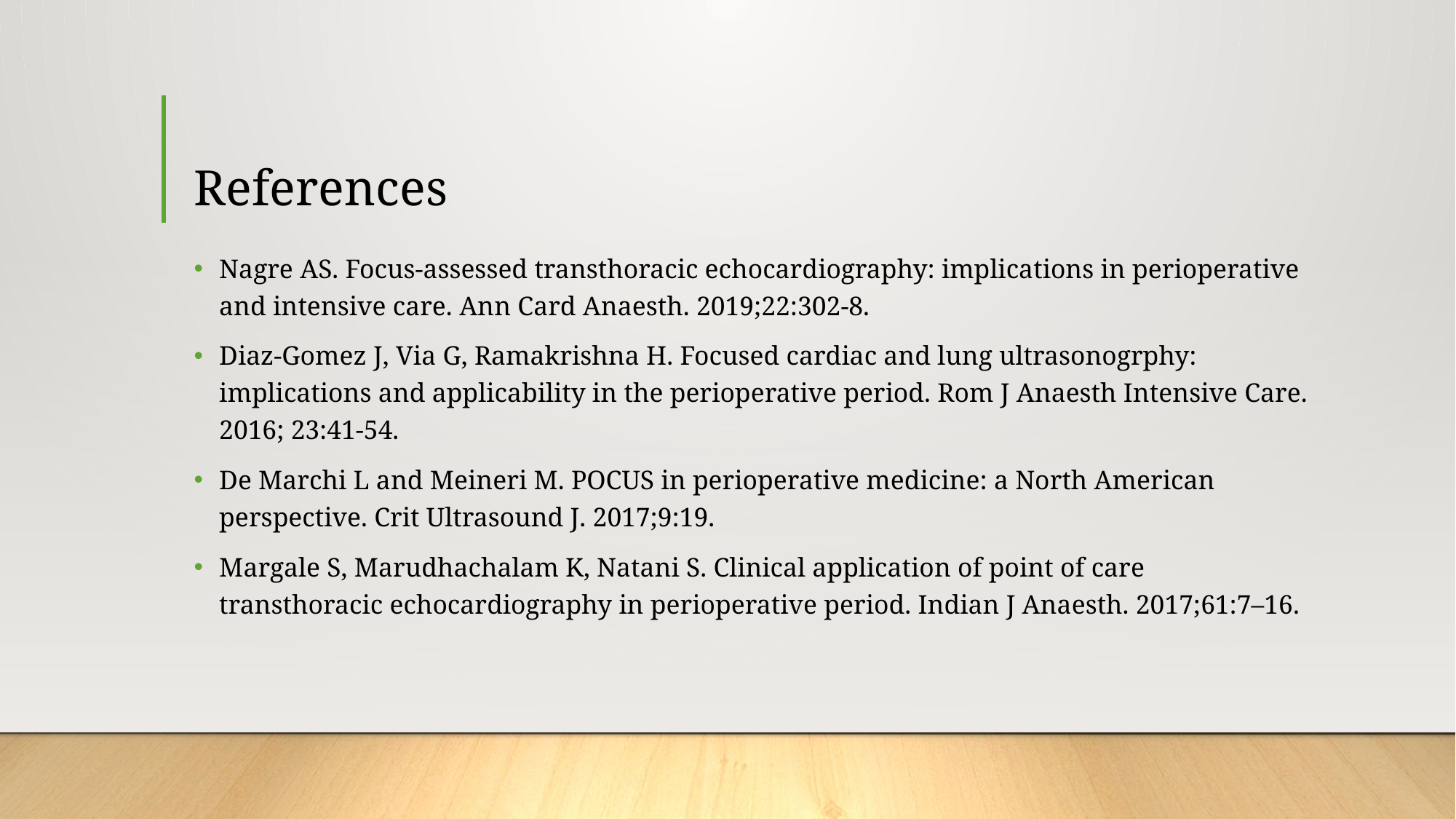

# References
Nagre AS. Focus-assessed transthoracic echocardiography: implications in perioperative and intensive care. Ann Card Anaesth. 2019;22:302-8.
Diaz-Gomez J, Via G, Ramakrishna H. Focused cardiac and lung ultrasonogrphy: implications and applicability in the perioperative period. Rom J Anaesth Intensive Care. 2016; 23:41-54.
De Marchi L and Meineri M. POCUS in perioperative medicine: a North American perspective. Crit Ultrasound J. 2017;9:19.
Margale S, Marudhachalam K, Natani S. Clinical application of point of care transthoracic echocardiography in perioperative period. Indian J Anaesth. 2017;61:7–16.

## Slide 48
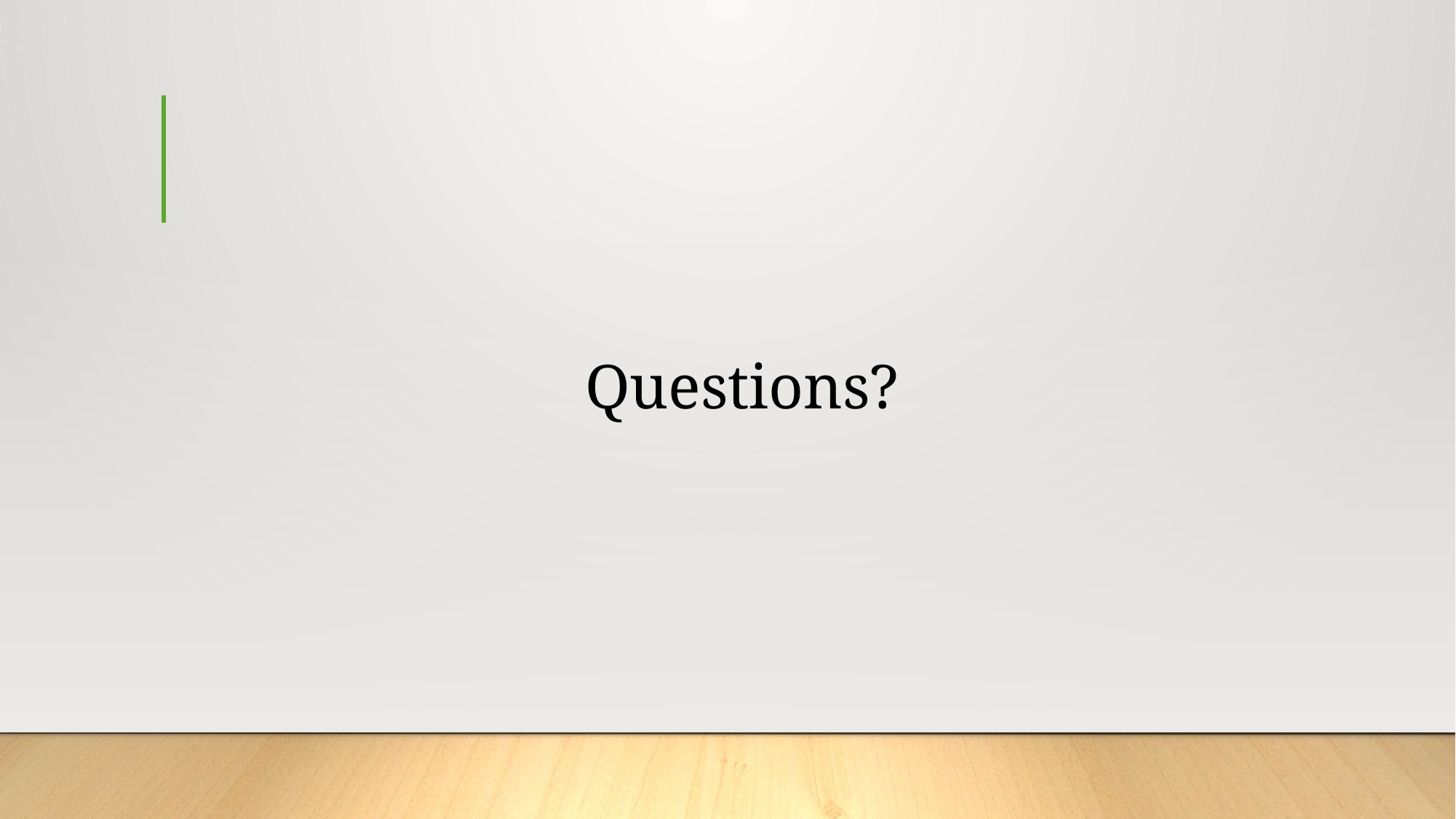

Questions?
