# Supplementary material for: An Introductory Point-of-Care Ultrasound Curriculum for an Anesthesiology Residency Program
Source: MedEdPORTAL. 2022 Dec 23;18:11291. doi: 10.15766/mep_2374-8265.11291 (PMC9780414; doi:10.15766/mep_2374-8265.11291)
Supplement: Supplementary file 1 — Ultrasound Basics.pptxLung Ultrasound.pptxCardiac Ultrasound.pptxVascular Access Ultrasound.pptxAirway Ultrasound.pptxAbdominal Ultrasound.pptxNeuraxial Ultrasound.pptxChecklist for POCUS Scanning.docxPOCUS CA1 Curriculum Pretest.pptxPOCUS CA1 Curriculum Posttest.pptxPOCUS Survey.docx [file mep_2374-8265.11291-s001.zip › G. Neuraxial Ultrasound.pptx]

## Slide 1
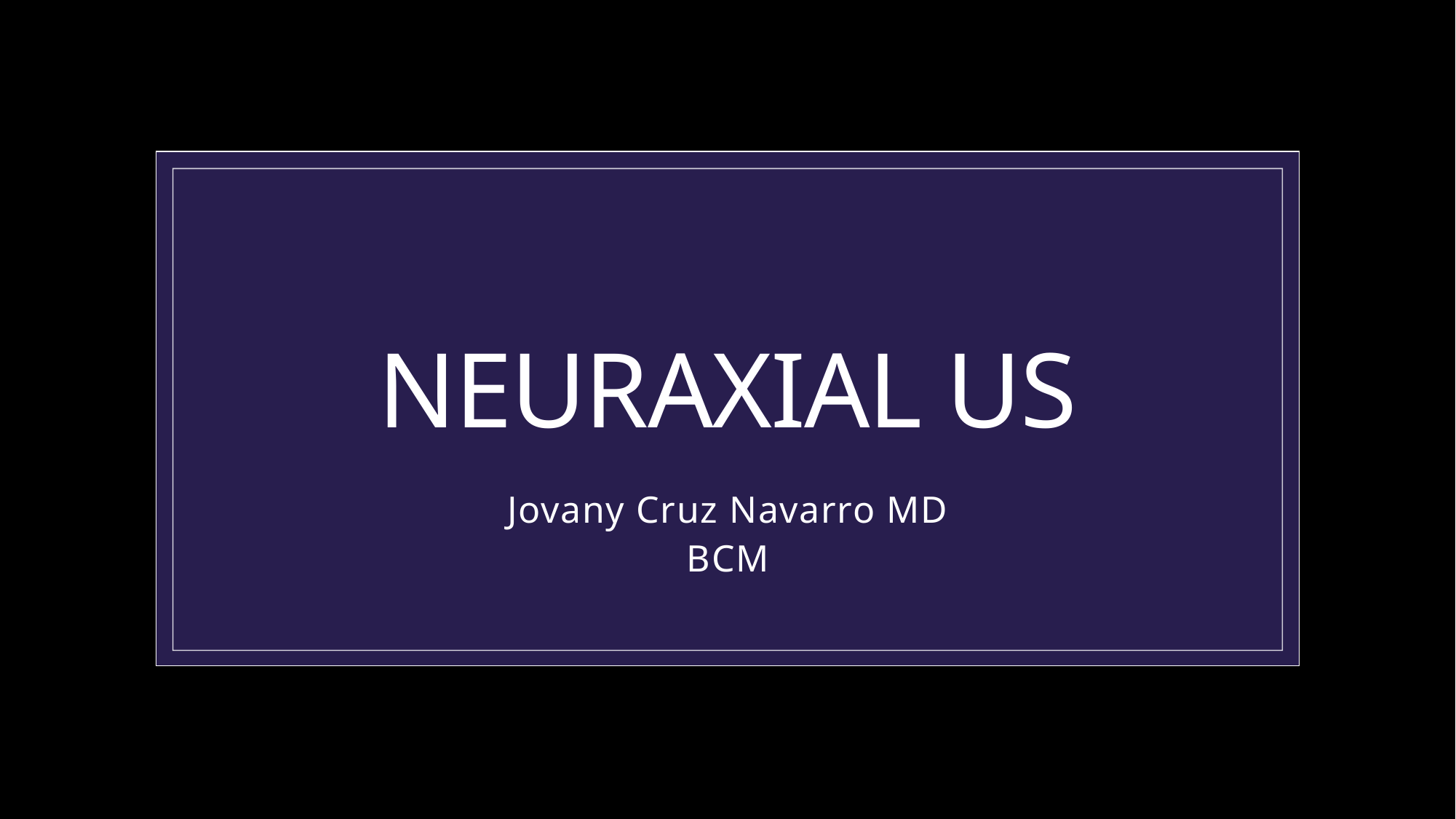

# NEURAXIAL US
Jovany Cruz Navarro MD
BCM

## Slide 2
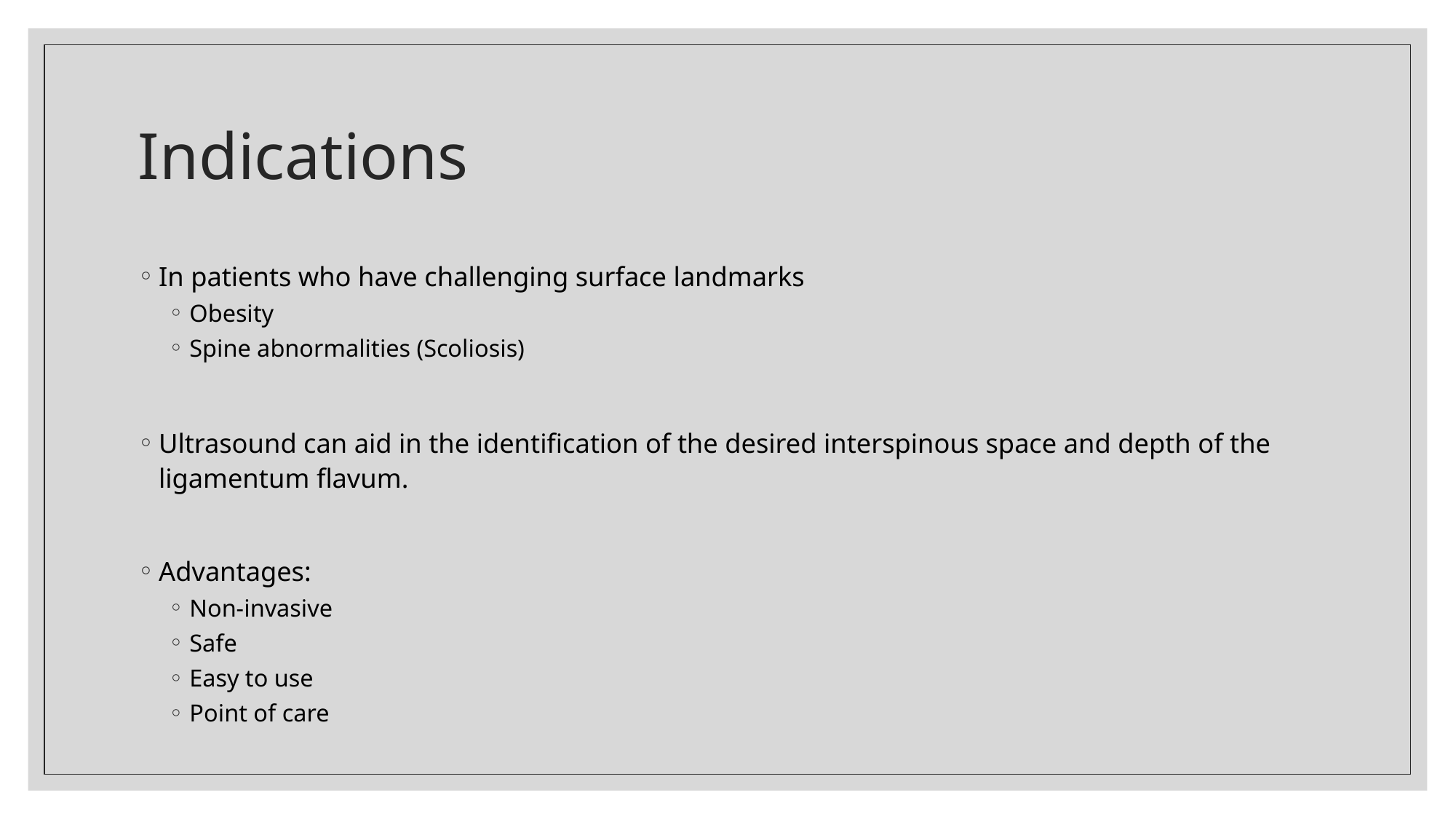

# Indications
In patients who have challenging surface landmarks
Obesity
Spine abnormalities (Scoliosis)
Ultrasound can aid in the identification of the desired interspinous space and depth of the ligamentum flavum.
Advantages:
Non-invasive
Safe
Easy to use
Point of care

## Slide 3
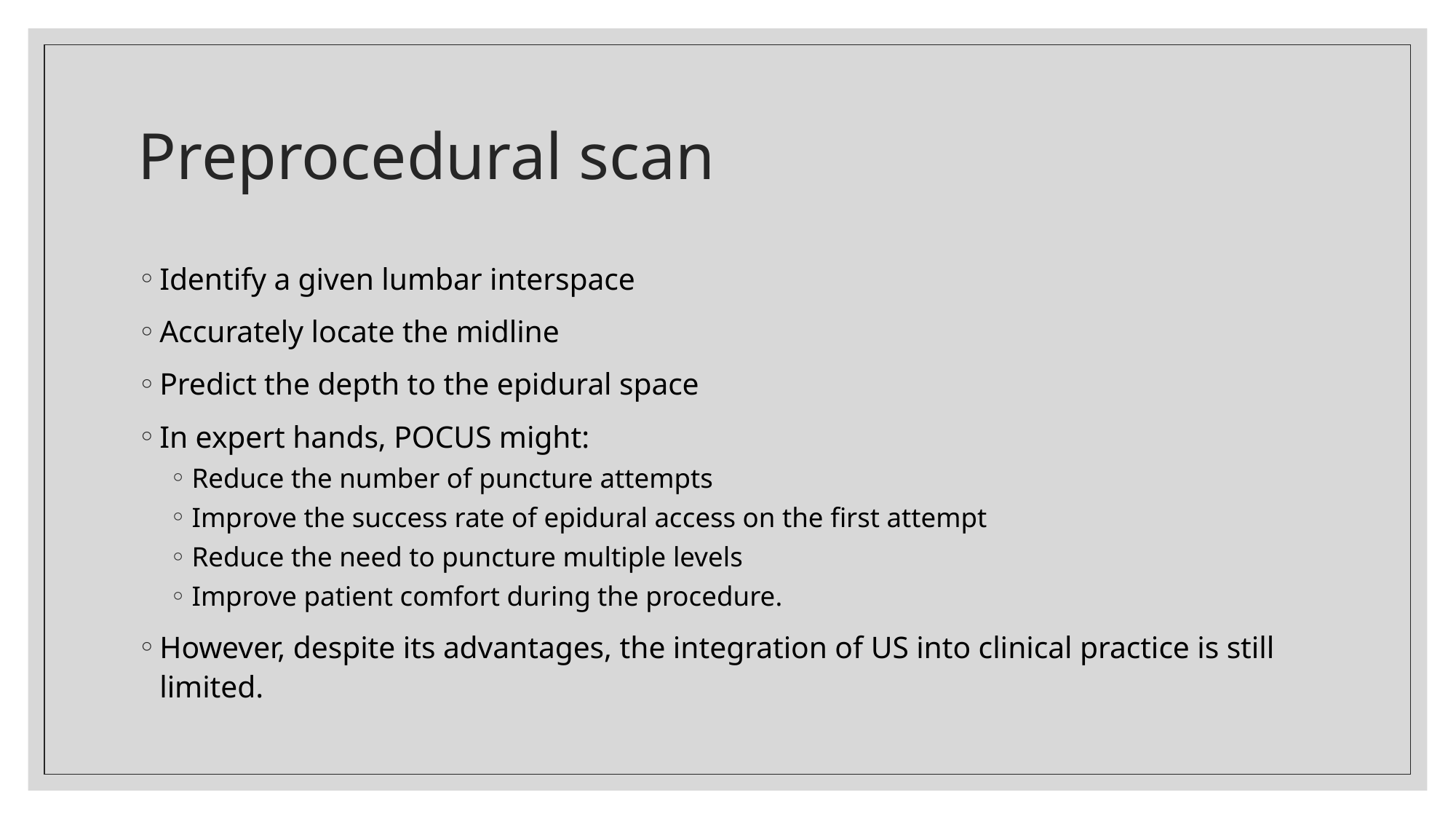

# Preprocedural scan
Identify a given lumbar interspace
Accurately locate the midline
Predict the depth to the epidural space
In expert hands, POCUS might:
Reduce the number of puncture attempts
Improve the success rate of epidural access on the first attempt
Reduce the need to puncture multiple levels
Improve patient comfort during the procedure.
However, despite its advantages, the integration of US into clinical practice is still limited.

## Slide 4
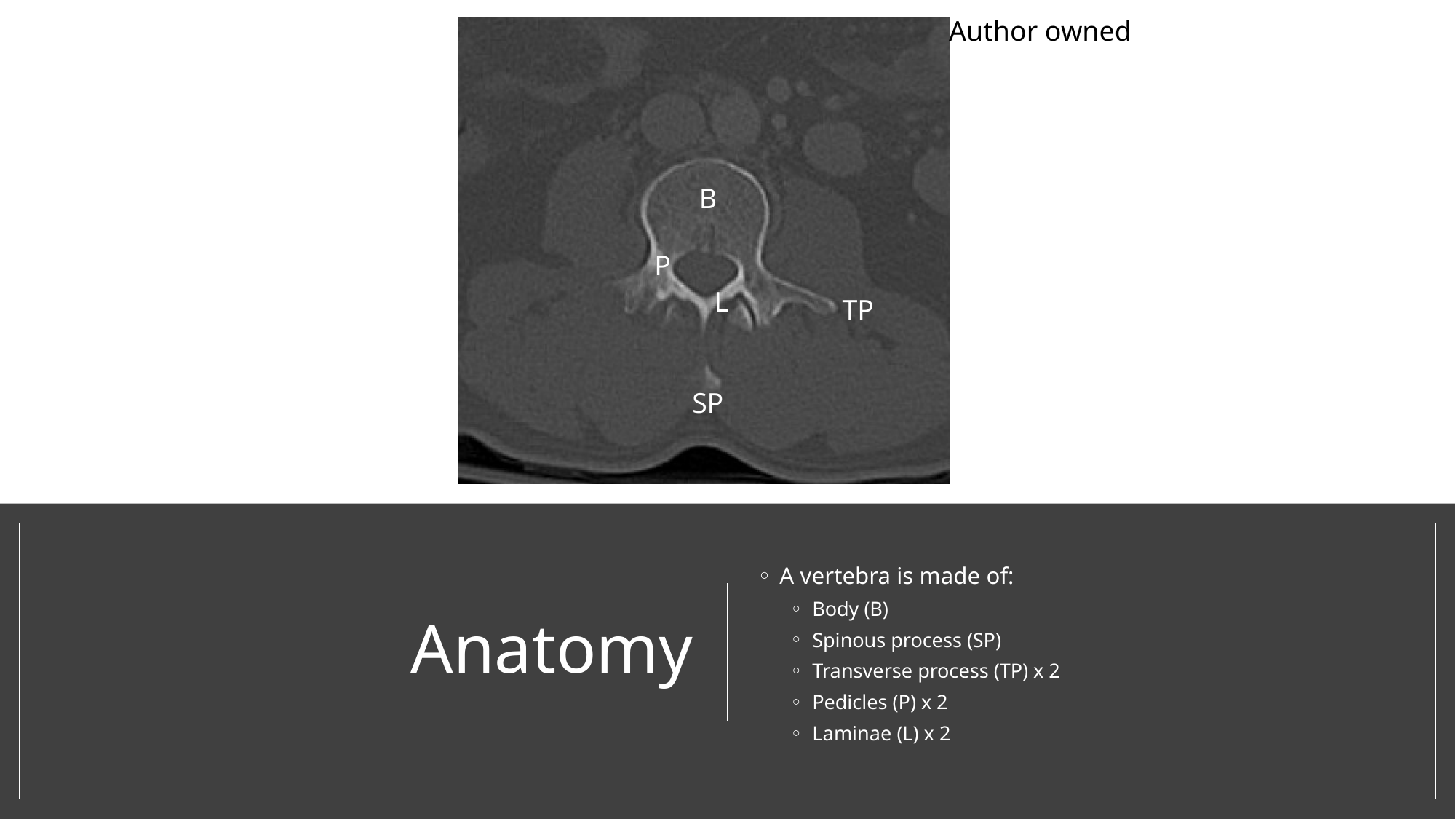

Author owned
B
P
L
TP
SP
# Anatomy
A vertebra is made of:
Body (B)
Spinous process (SP)
Transverse process (TP) x 2
Pedicles (P) x 2
Laminae (L) x 2

## Slide 5
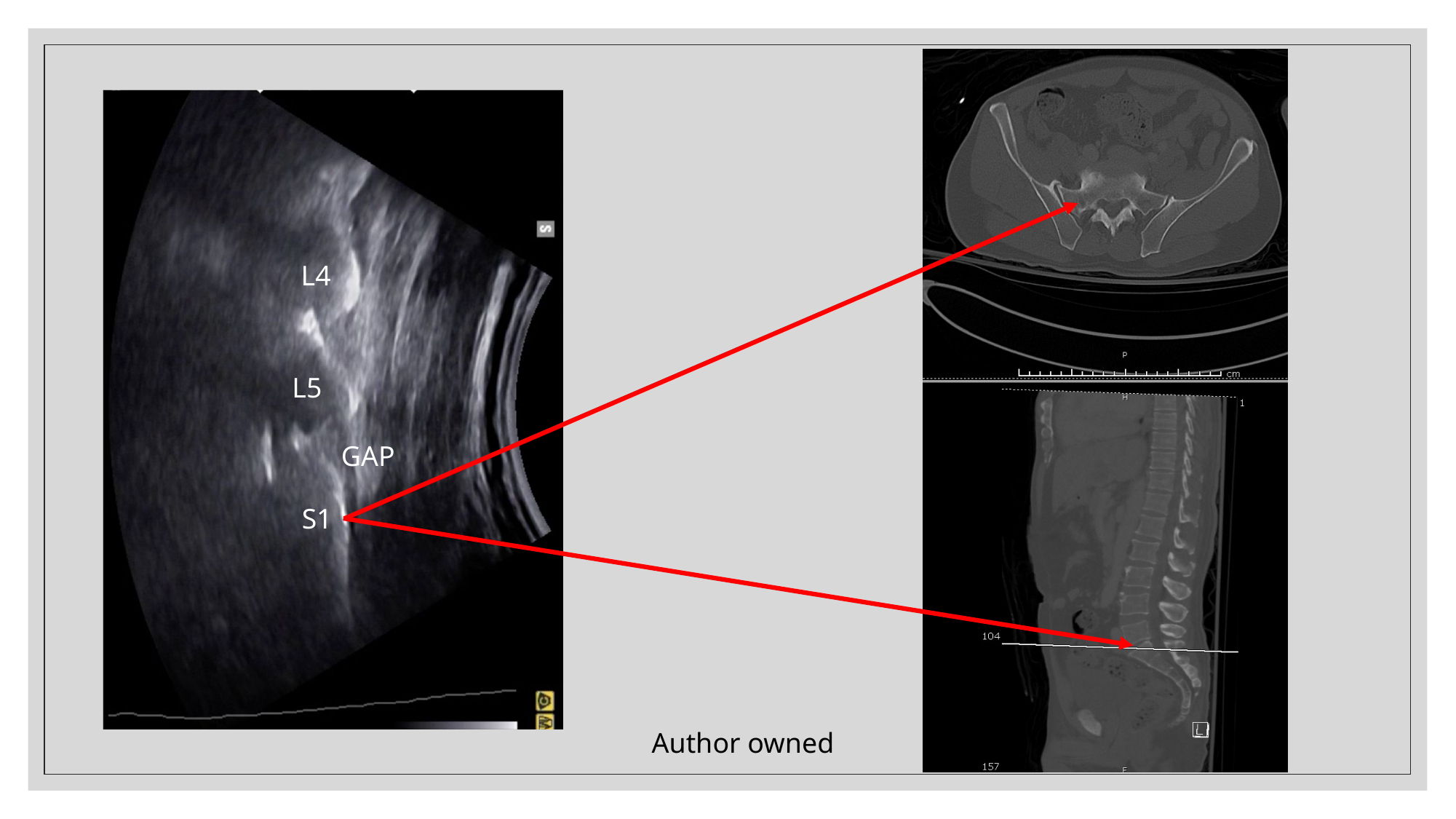

#
L4
L5
GAP
S1
Author owned

## Slide 6
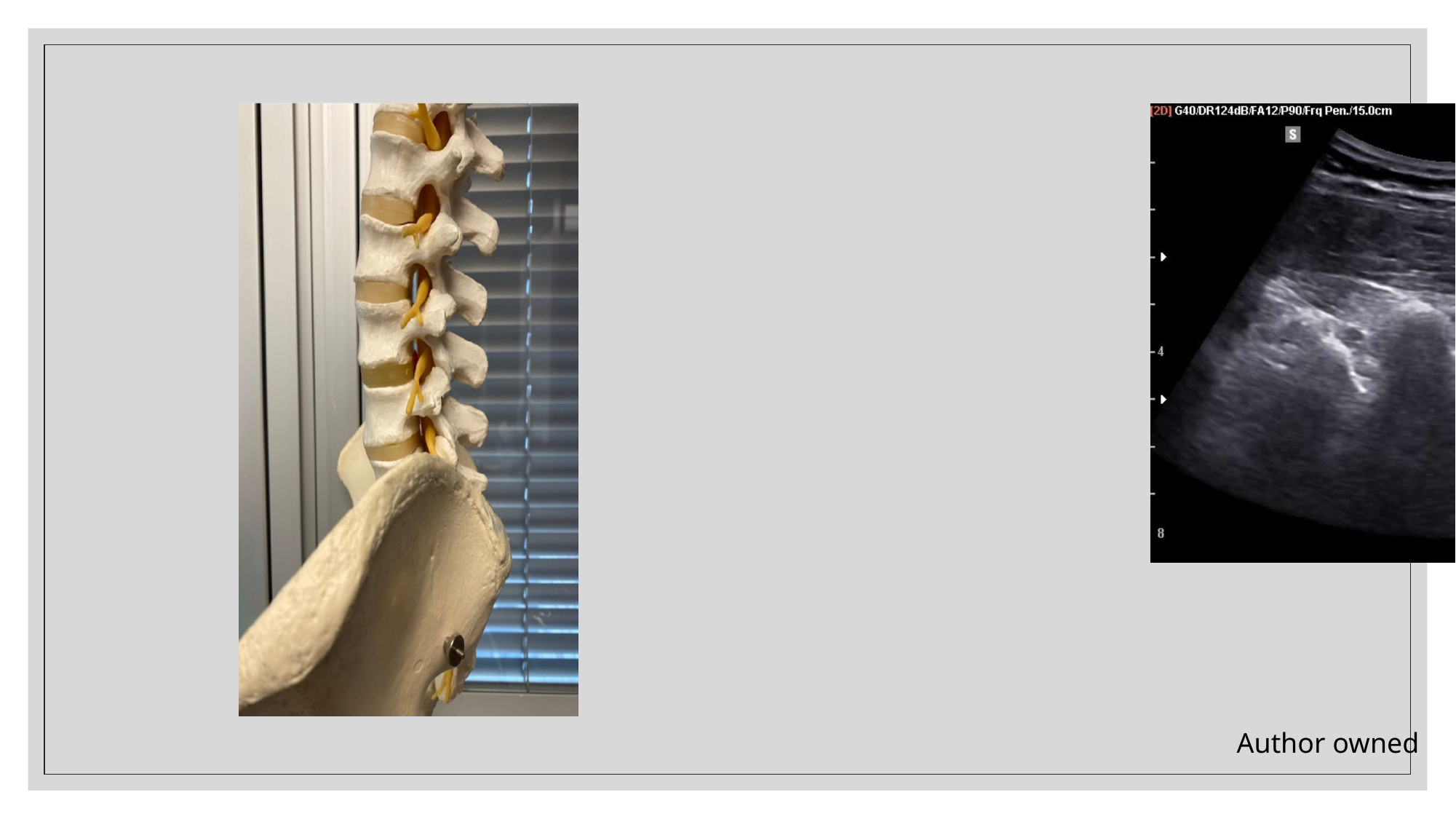

#
Author owned

## Slide 7
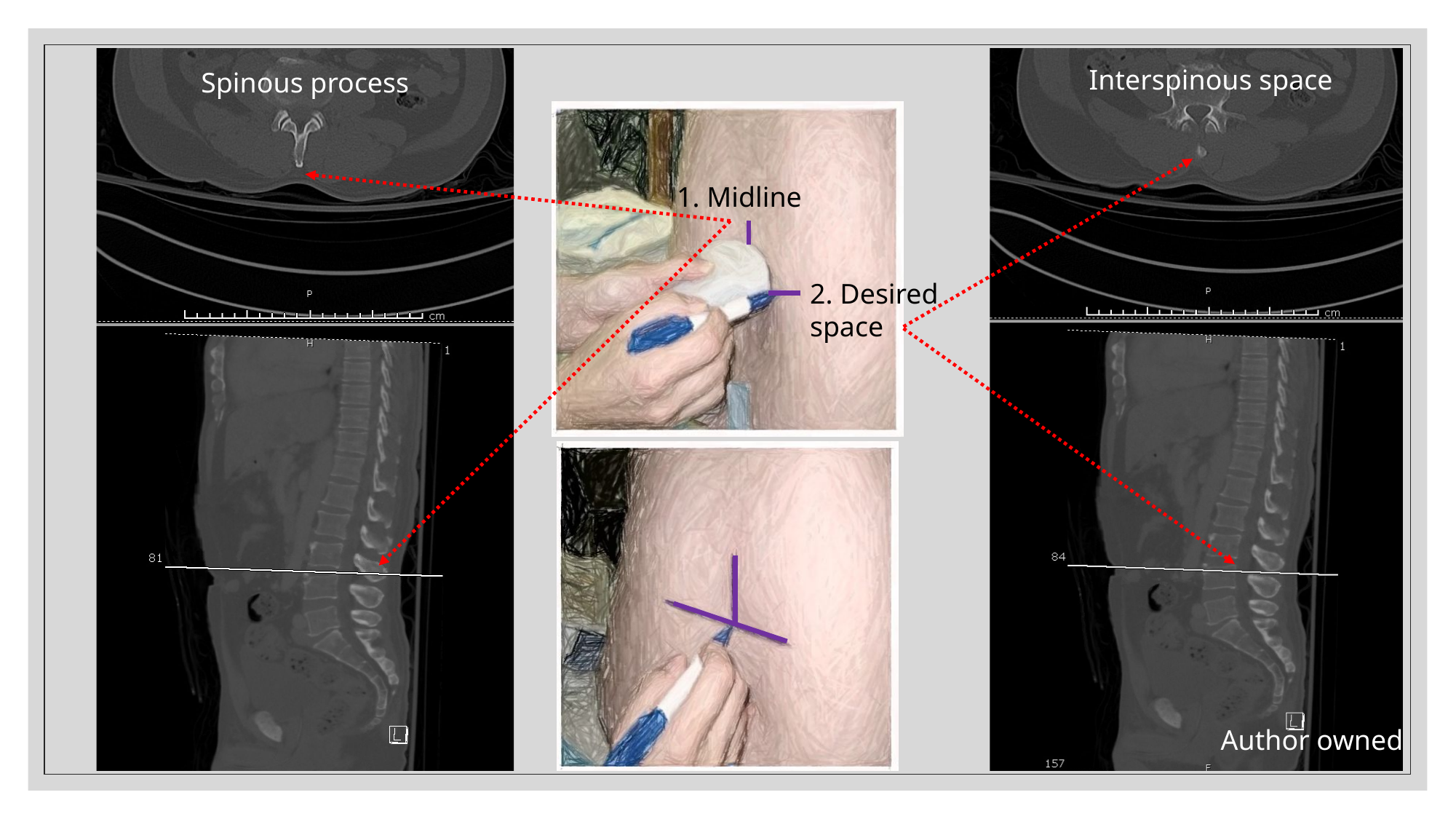

Interspinous space
Spinous process
#
1. Midline
2. Desired space
Author owned

## Slide 8
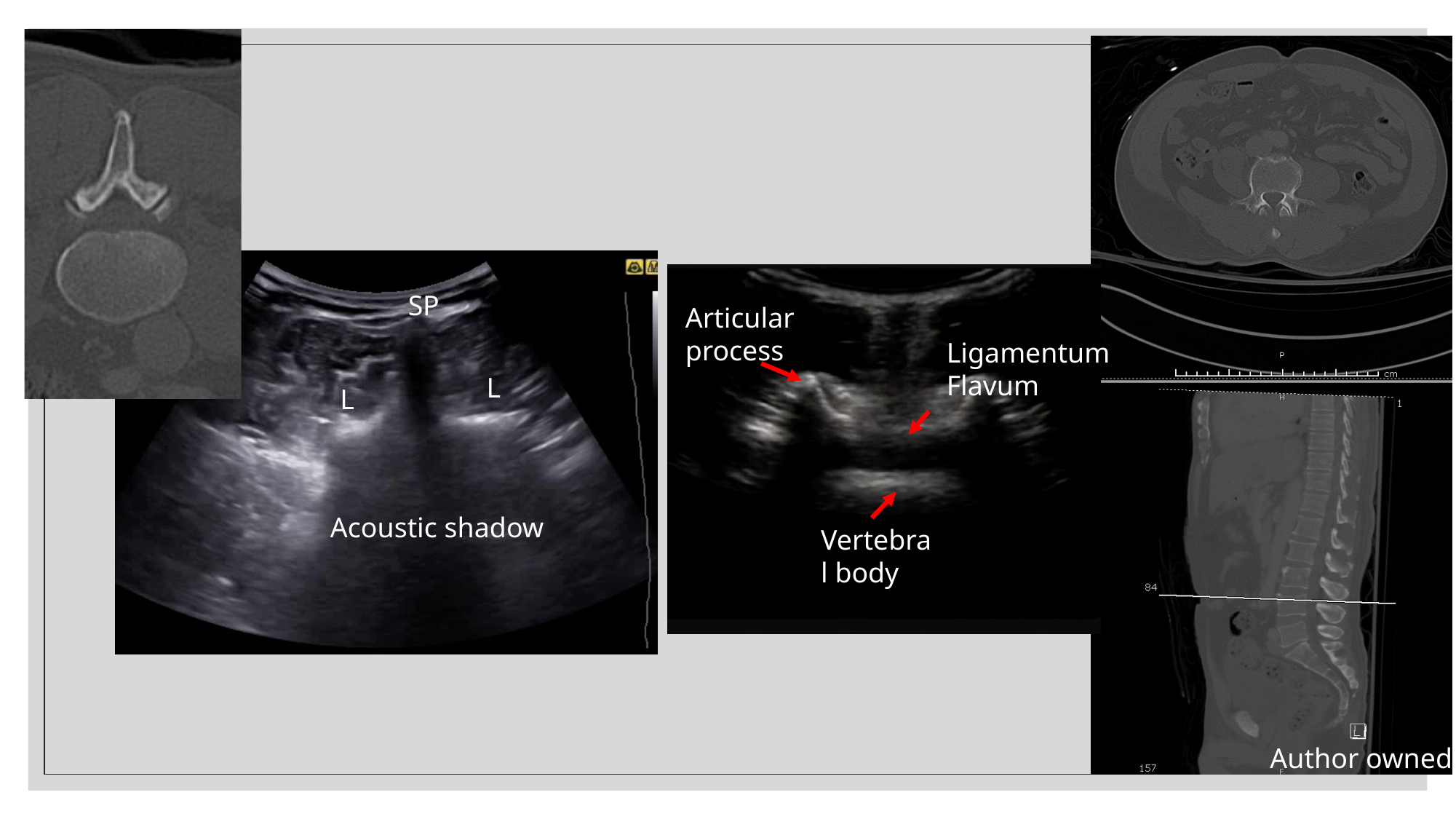

#
SP
Articular process
Ligamentum Flavum
L
L
Acoustic shadow
Vertebral body
Author owned
