# Supplementary material for: An Introductory Point-of-Care Ultrasound Curriculum for an Anesthesiology Residency Program
Source: MedEdPORTAL. 2022 Dec 23;18:11291. doi: 10.15766/mep_2374-8265.11291 (PMC9780414; doi:10.15766/mep_2374-8265.11291)
Supplement: Supplementary file 1 — Ultrasound Basics.pptxLung Ultrasound.pptxCardiac Ultrasound.pptxVascular Access Ultrasound.pptxAirway Ultrasound.pptxAbdominal Ultrasound.pptxNeuraxial Ultrasound.pptxChecklist for POCUS Scanning.docxPOCUS CA1 Curriculum Pretest.pptxPOCUS CA1 Curriculum Posttest.pptxPOCUS Survey.docx [file mep_2374-8265.11291-s001.zip › H. Checklist for POCUS Scanning.docx]

**Checklist for POCUS Scanning**

| Station | Views Required | Structure Identified |
| --- | --- | --- |
| Cardiac | Parasternal long axis  Parasternal short axis  Apical 4 chamber  Subxiphoid 4 chamber  Subxiphoid inferior vena cava | Right ventricle  Left ventricle  Right atrium  Left atrium  Mitral valve  Aortic valve  Inferior vena cava |
| Pulmonary | Upper lung field  Lower lung field | Pleura  Diaphragm  Ribs |
| Abdomen | Right upper quadrant  Left upper quadrant  Pelvic  Gastric | Liver  Spleen  Kidney  Bladder  Gastric antrum  Abdominal aorta |
| Neuraxial | Sagittal  Transverse | Spinous process  Laminae  Epidural space |
| Airway | Sagittal  Transverse | Thyroid cartilage  Cricoid cartilage  Cricothyroid membrane  Tracheal rings |
| Vascular | Lateral neck | Carotid artery  Internal jugular vein |
