# Supplementary material for: An Introductory Point-of-Care Ultrasound Curriculum for an Anesthesiology Residency Program
Source: MedEdPORTAL. 2022 Dec 23;18:11291. doi: 10.15766/mep_2374-8265.11291 (PMC9780414; doi:10.15766/mep_2374-8265.11291)
Supplement: Supplementary file 1 — Ultrasound Basics.pptxLung Ultrasound.pptxCardiac Ultrasound.pptxVascular Access Ultrasound.pptxAirway Ultrasound.pptxAbdominal Ultrasound.pptxNeuraxial Ultrasound.pptxChecklist for POCUS Scanning.docxPOCUS CA1 Curriculum Pretest.pptxPOCUS CA1 Curriculum Posttest.pptxPOCUS Survey.docx [file mep_2374-8265.11291-s001.zip › I. POCUS CA1 Curriculum Pretest.pptx]

## Slide 1
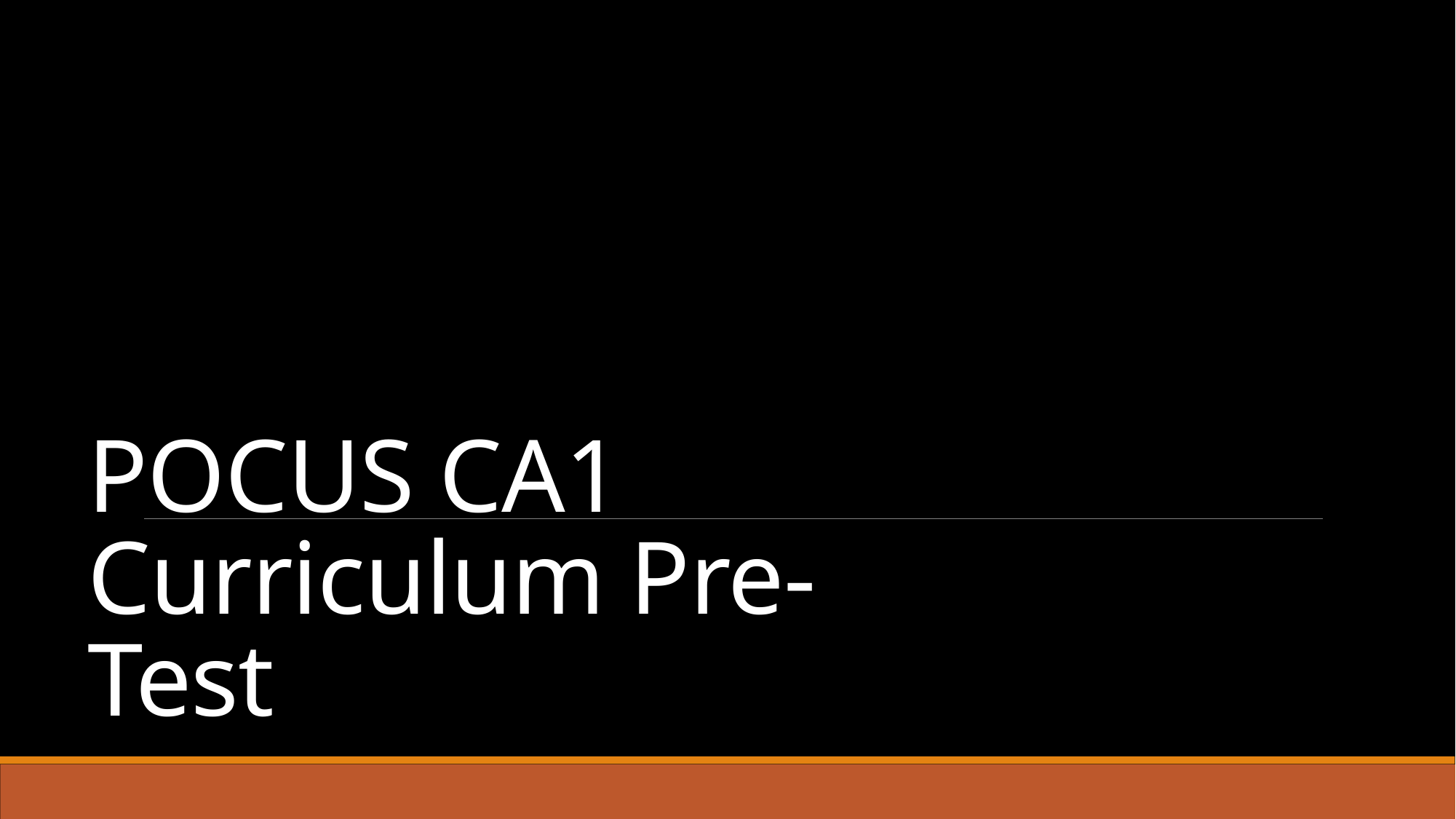

# POCUS CA1 Curriculum Pre-Test

## Slide 2
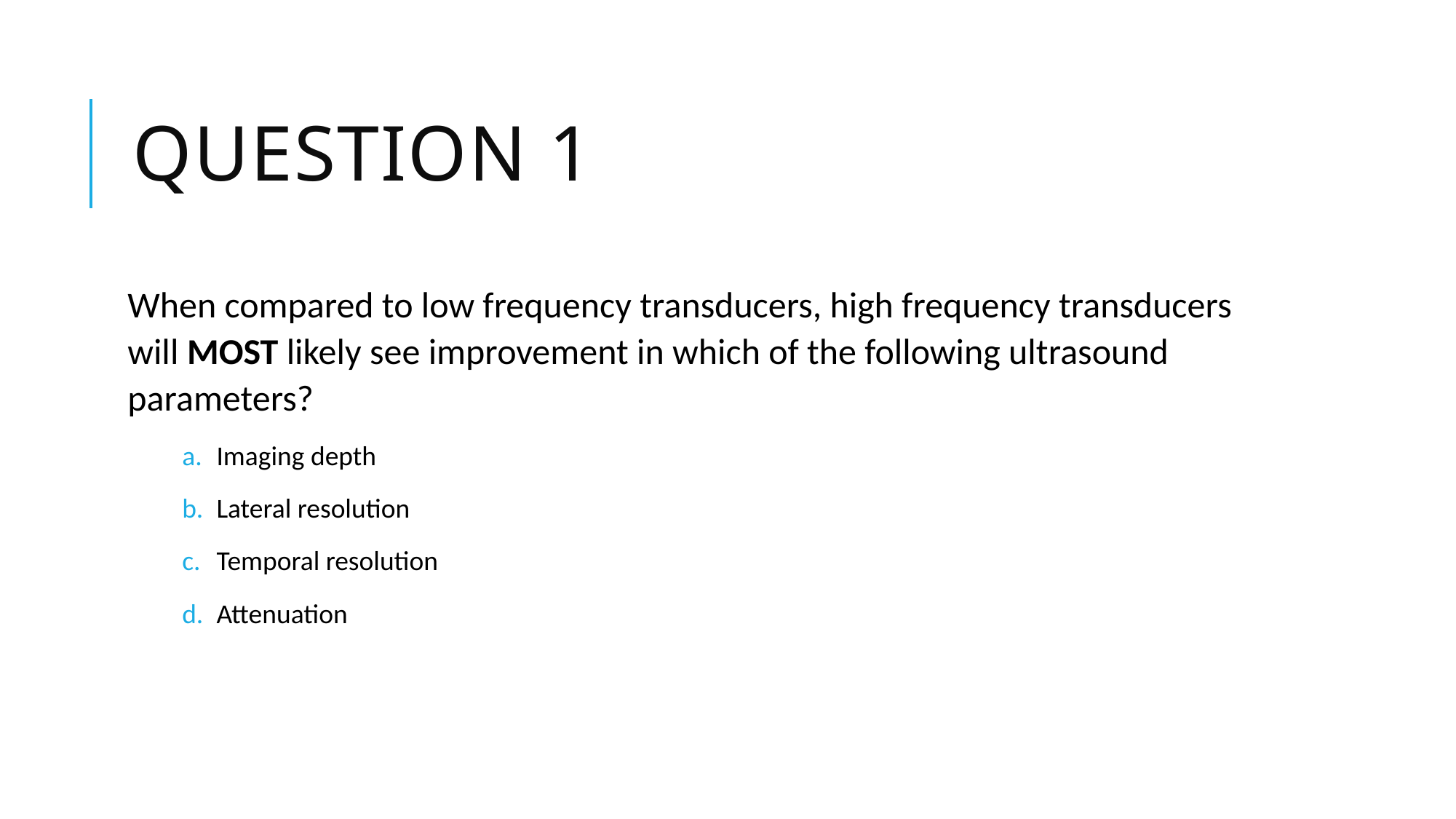

# Question 1
When compared to low frequency transducers, high frequency transducers will MOST likely see improvement in which of the following ultrasound parameters?
Imaging depth
Lateral resolution
Temporal resolution
Attenuation

## Slide 3
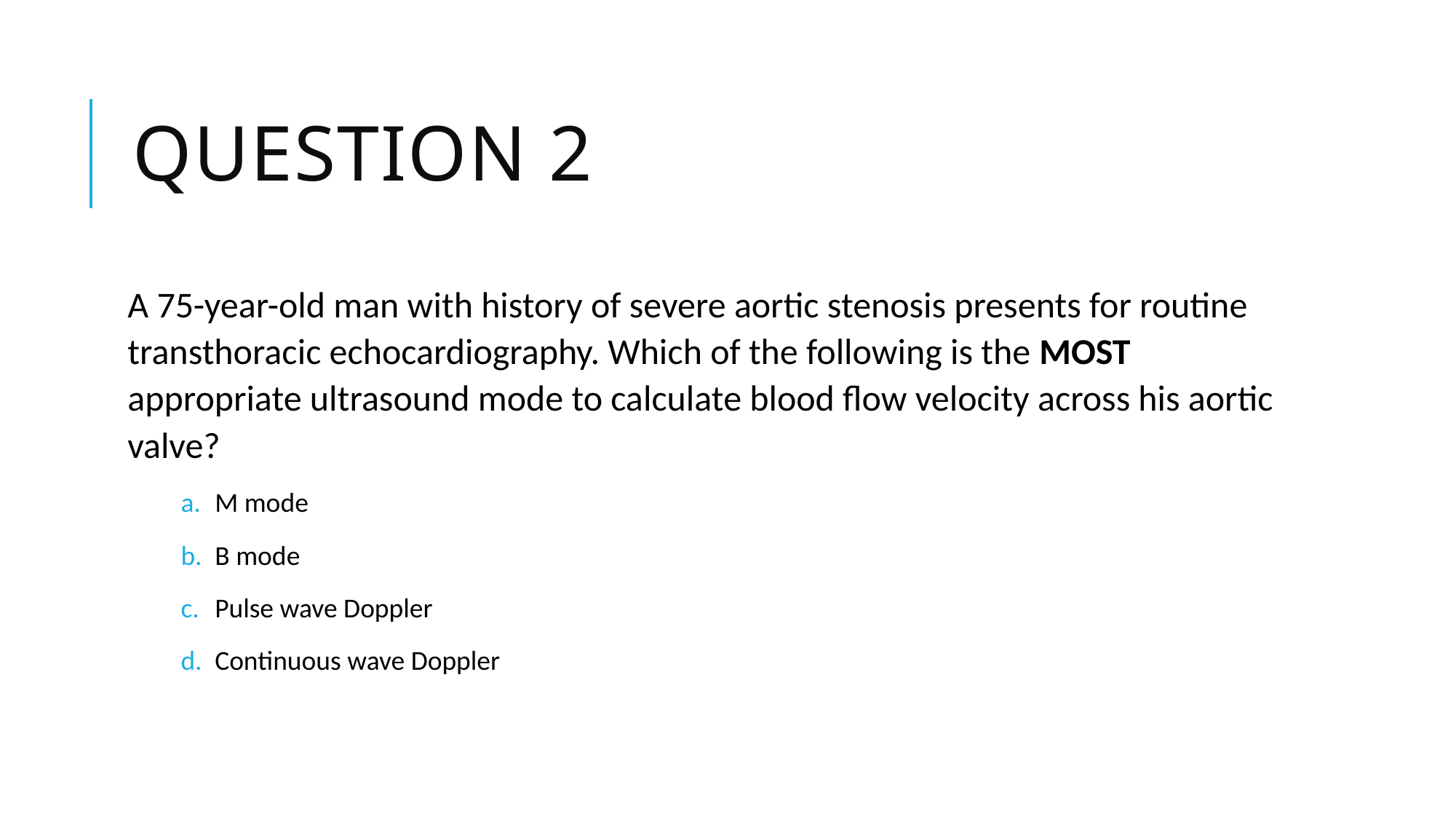

# Question 2
A 75-year-old man with history of severe aortic stenosis presents for routine transthoracic echocardiography. Which of the following is the MOST appropriate ultrasound mode to calculate blood flow velocity across his aortic valve?
M mode
B mode
Pulse wave Doppler
Continuous wave Doppler

## Slide 4
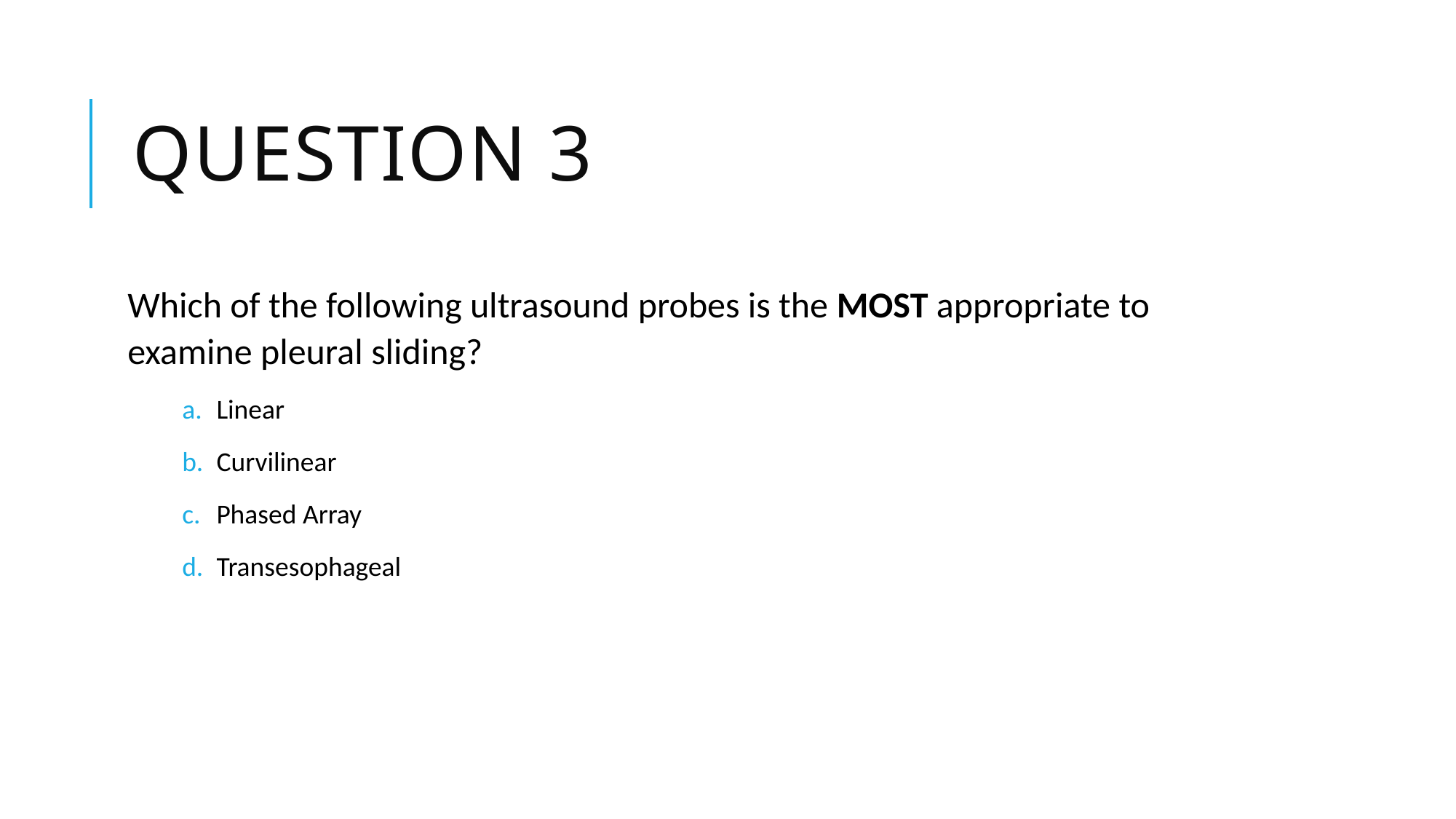

# Question 3
Which of the following ultrasound probes is the MOST appropriate to examine pleural sliding?
Linear
Curvilinear
Phased Array
Transesophageal

## Slide 5
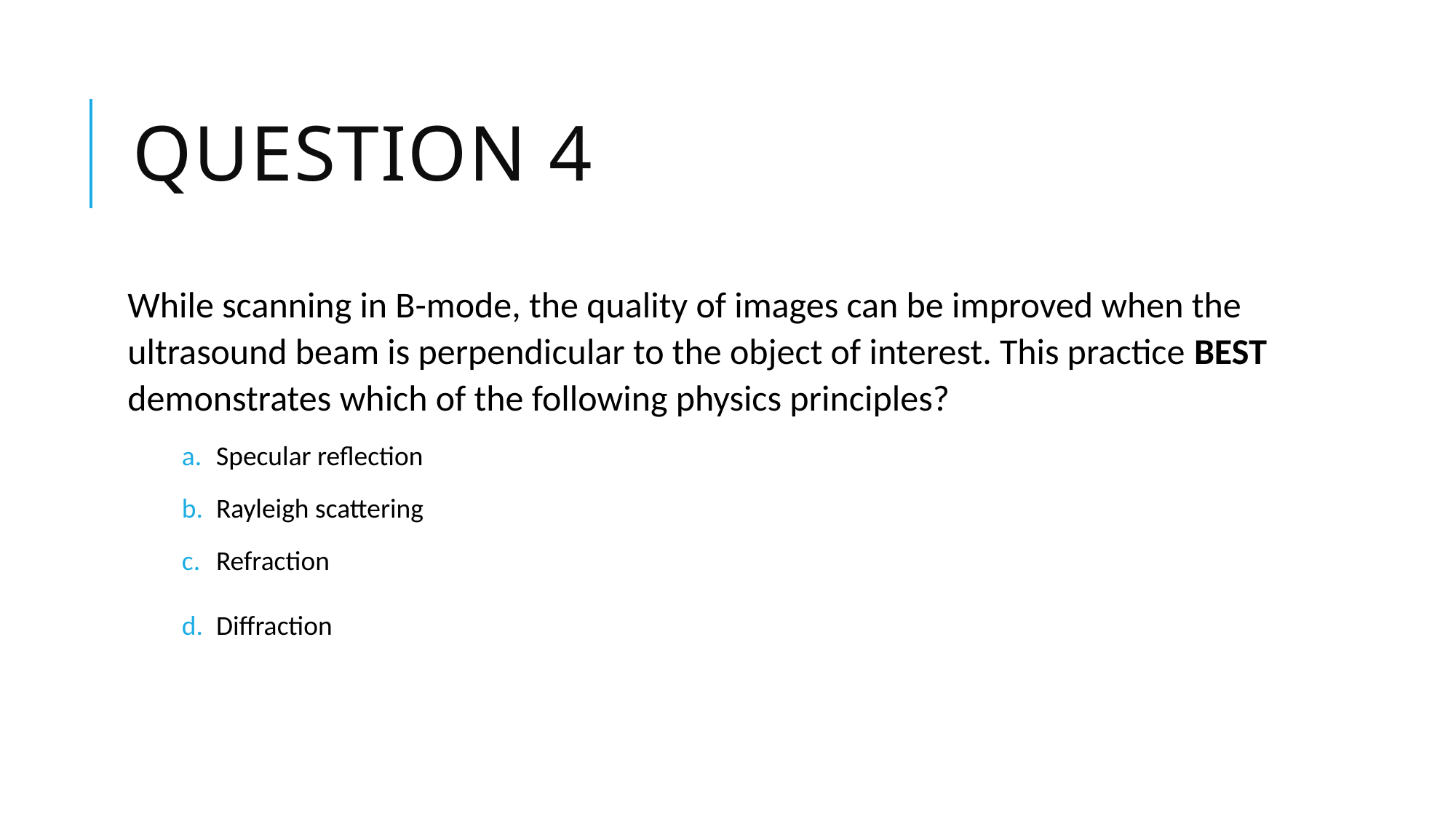

# Question 4
While scanning in B-mode, the quality of images can be improved when the ultrasound beam is perpendicular to the object of interest. This practice BEST demonstrates which of the following physics principles?
Specular reflection
Rayleigh scattering
Refraction
Diffraction

## Slide 6
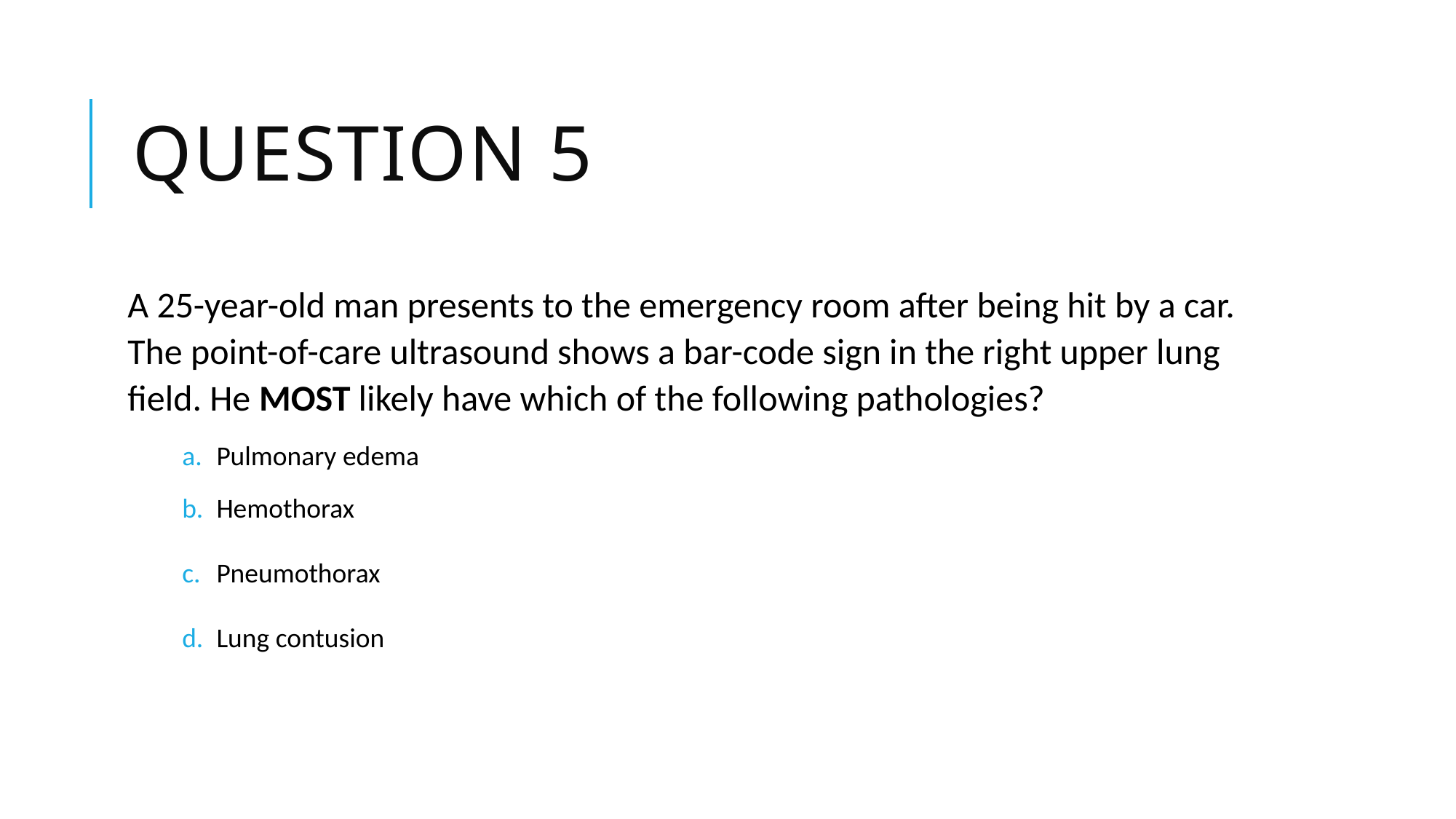

# Question 5
A 25-year-old man presents to the emergency room after being hit by a car. The point-of-care ultrasound shows a bar-code sign in the right upper lung field. He MOST likely have which of the following pathologies?
Pulmonary edema
Hemothorax
Pneumothorax
Lung contusion

## Slide 7
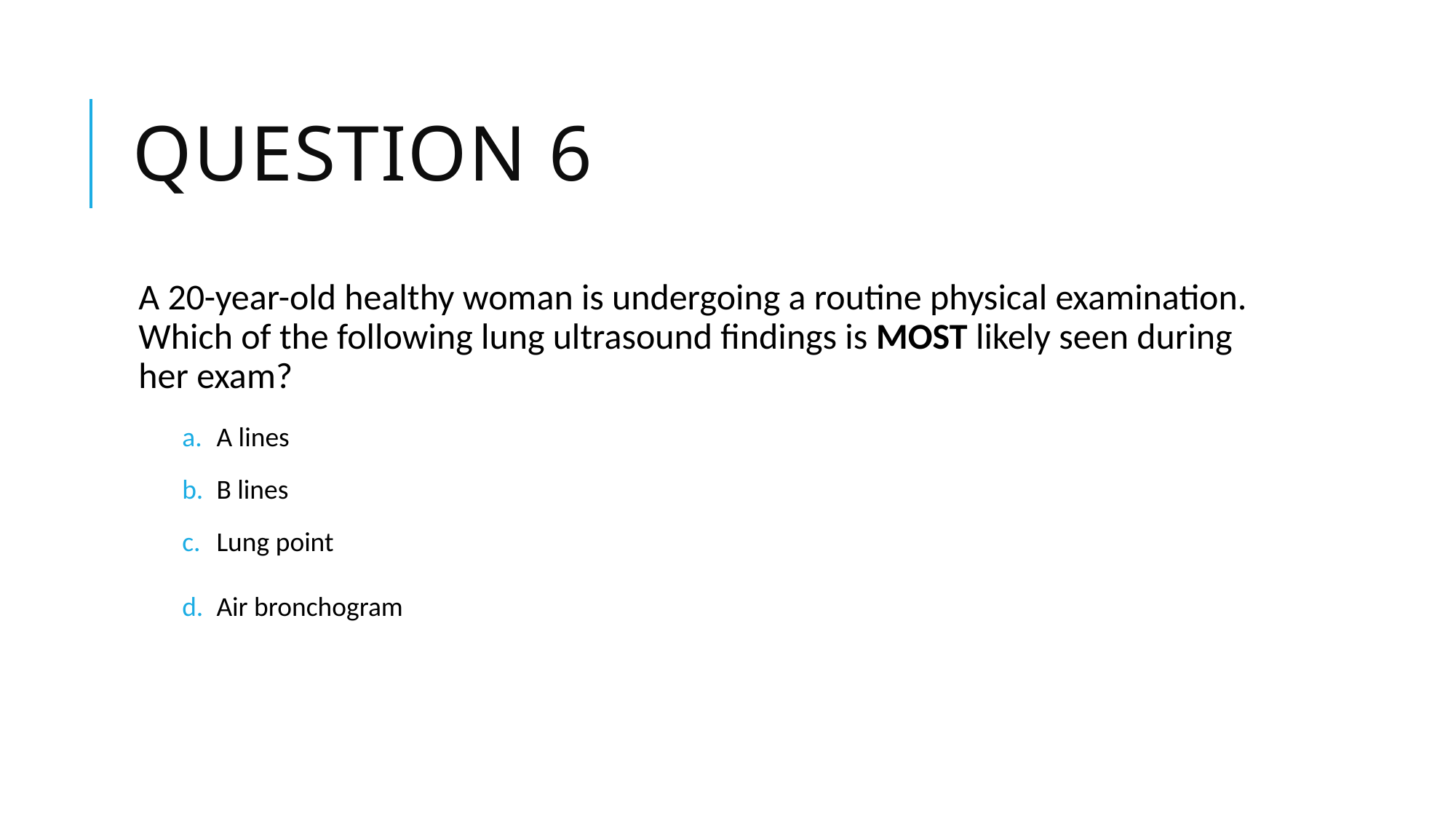

# Question 6
A 20-year-old healthy woman is undergoing a routine physical examination. Which of the following lung ultrasound findings is MOST likely seen during her exam?
A lines
B lines
Lung point
Air bronchogram

## Slide 8
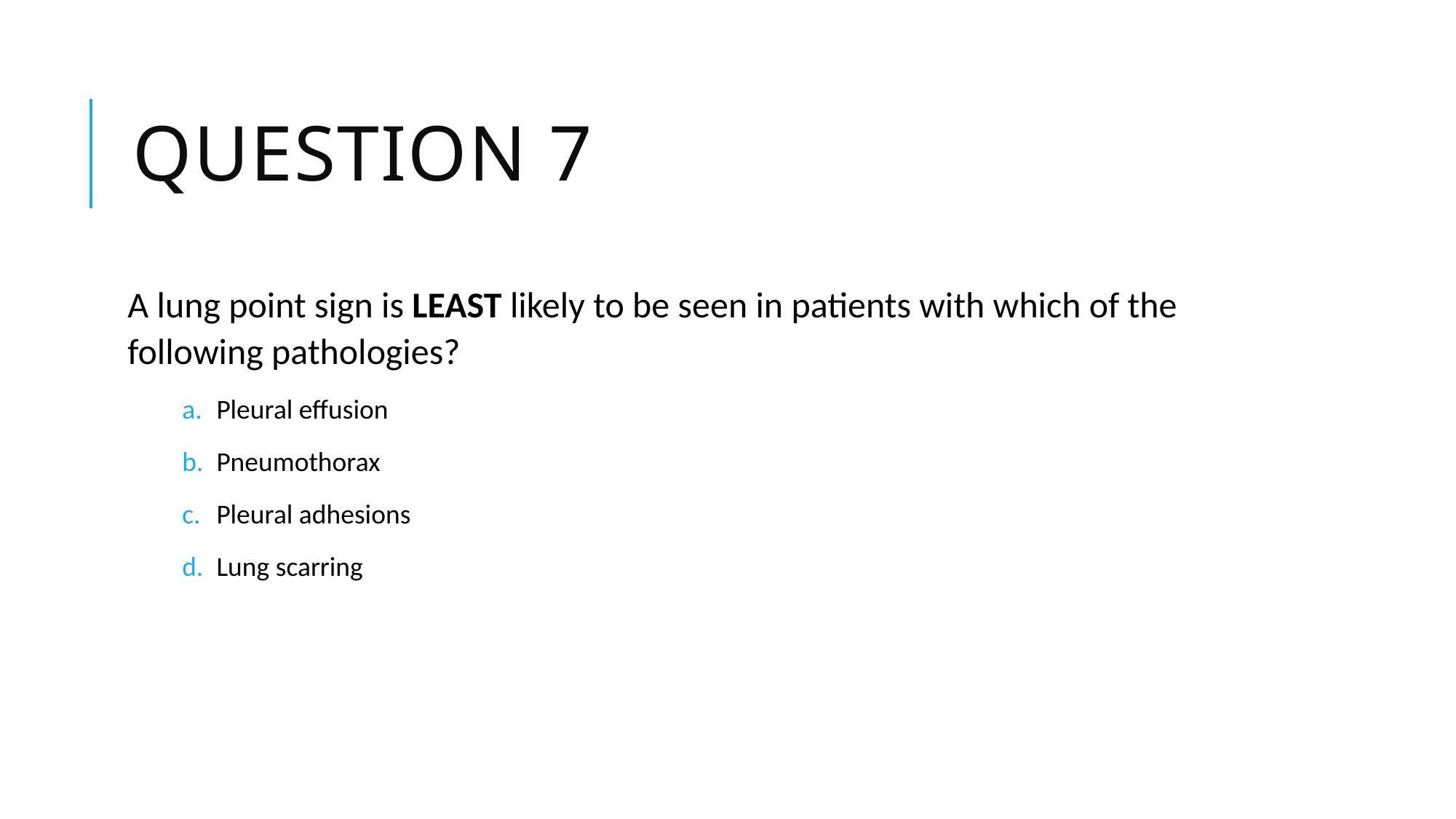

# Question 7
A lung point sign is LEAST likely to be seen in patients with which of the following pathologies?
Pleural effusion
Pneumothorax
Pleural adhesions
Lung scarring

## Slide 9
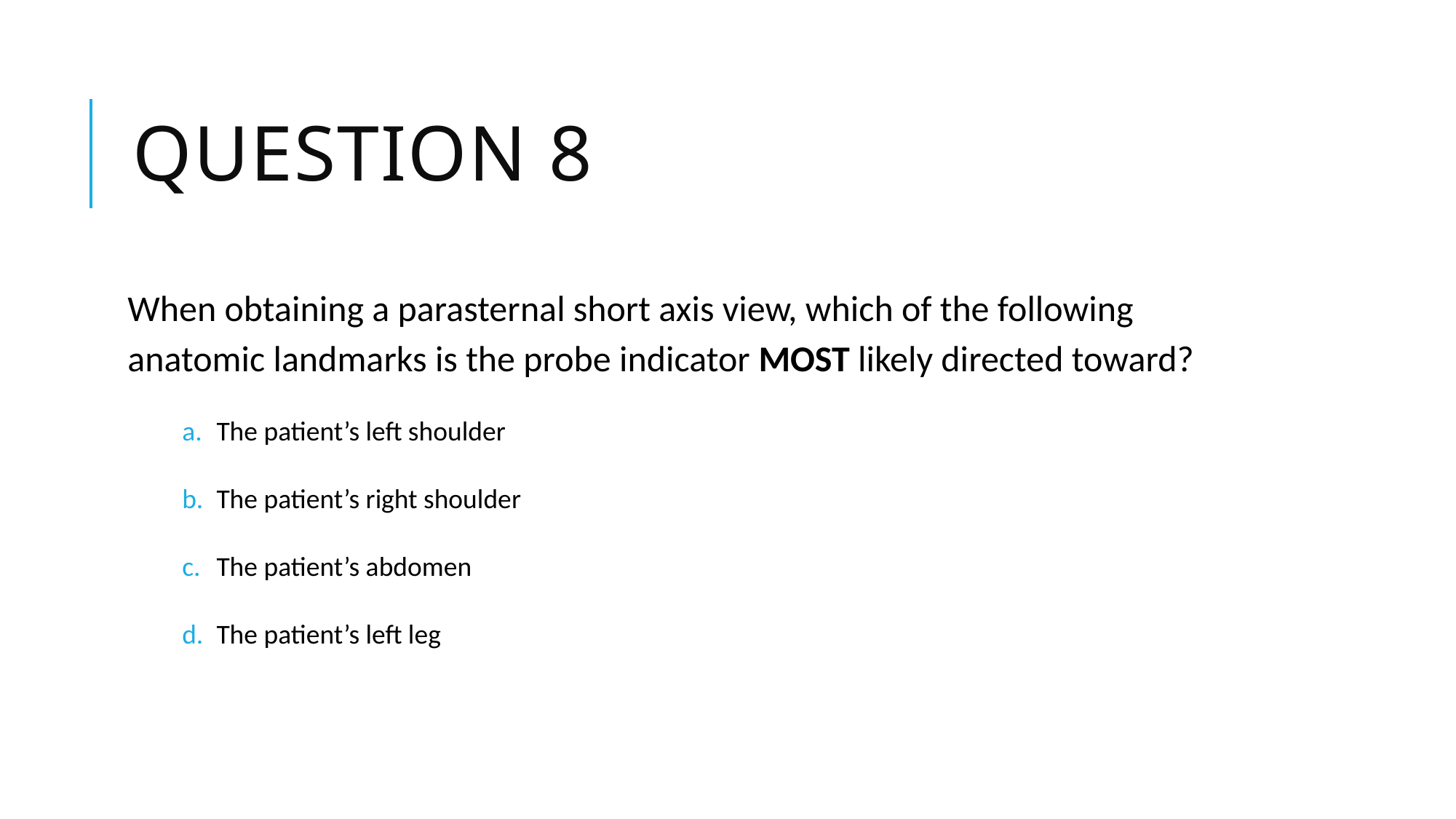

# Question 8
When obtaining a parasternal short axis view, which of the following anatomic landmarks is the probe indicator MOST likely directed toward?
The patient’s left shoulder
The patient’s right shoulder
The patient’s abdomen
The patient’s left leg

## Slide 10
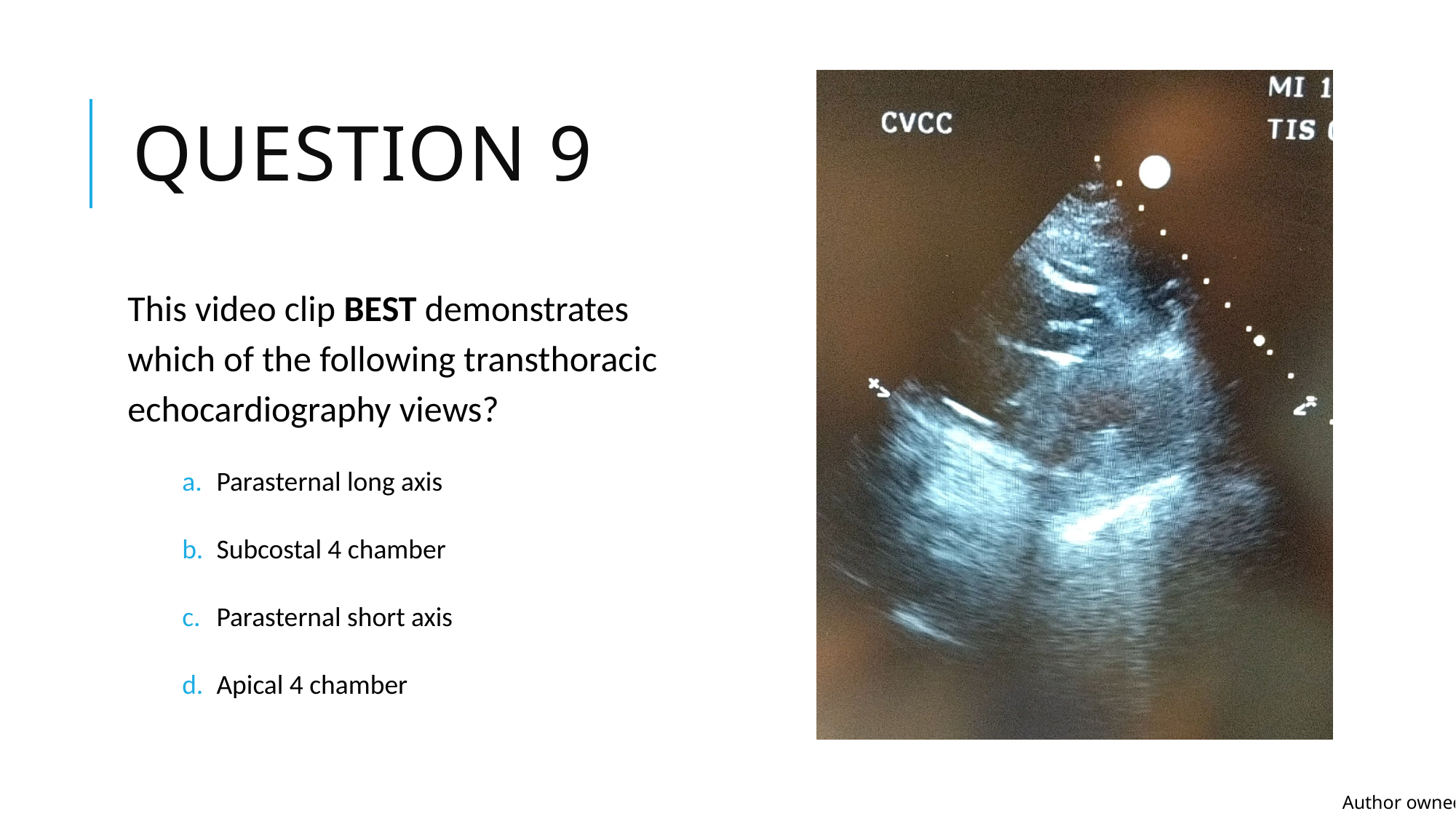

# Question 9
This video clip BEST demonstrates which of the following transthoracic echocardiography views?
Parasternal long axis
Subcostal 4 chamber
Parasternal short axis
Apical 4 chamber
Author owned

## Slide 11
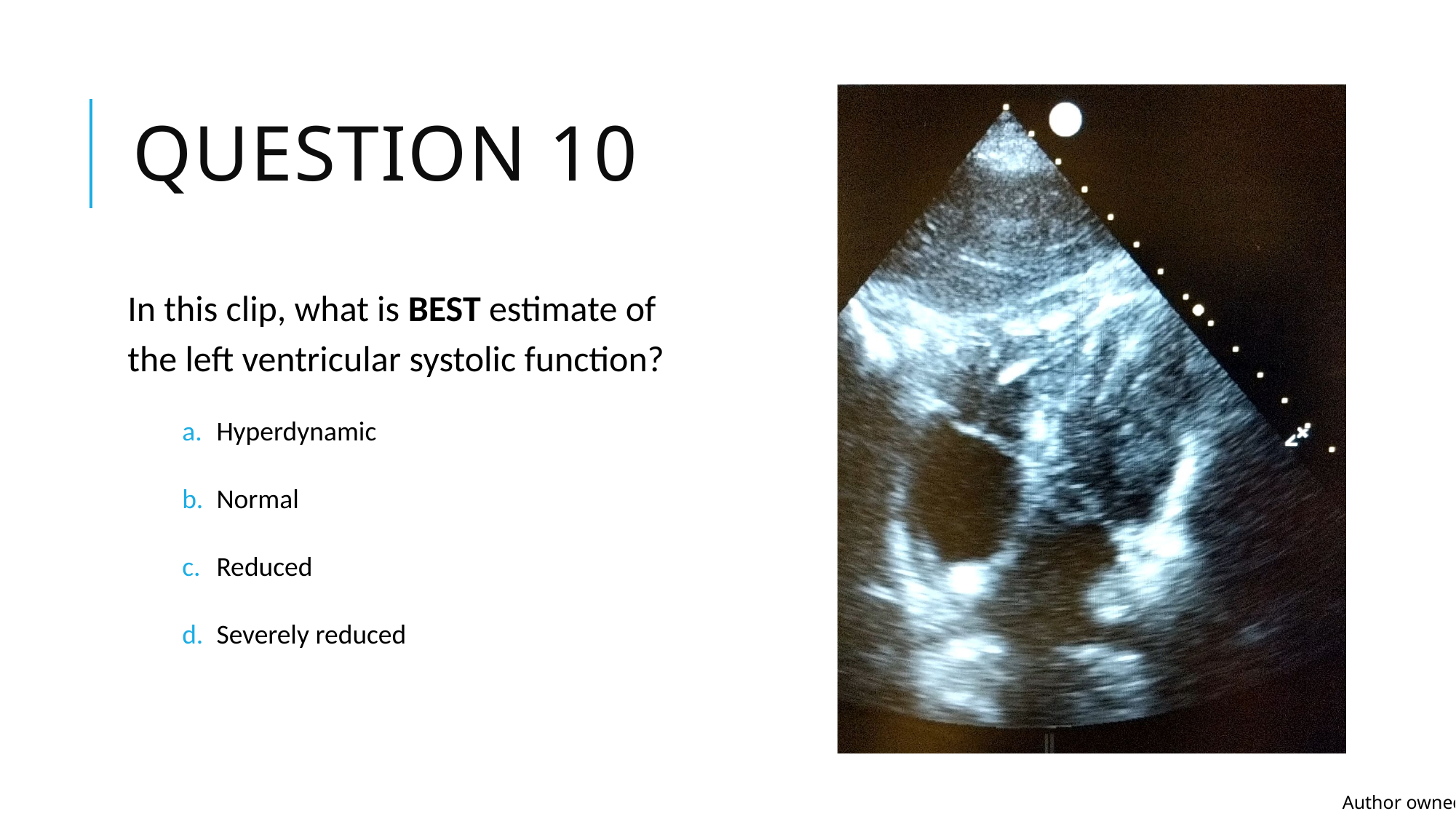

# Question 10
In this clip, what is BEST estimate of the left ventricular systolic function?
Hyperdynamic
Normal
Reduced
Severely reduced
Author owned

## Slide 12
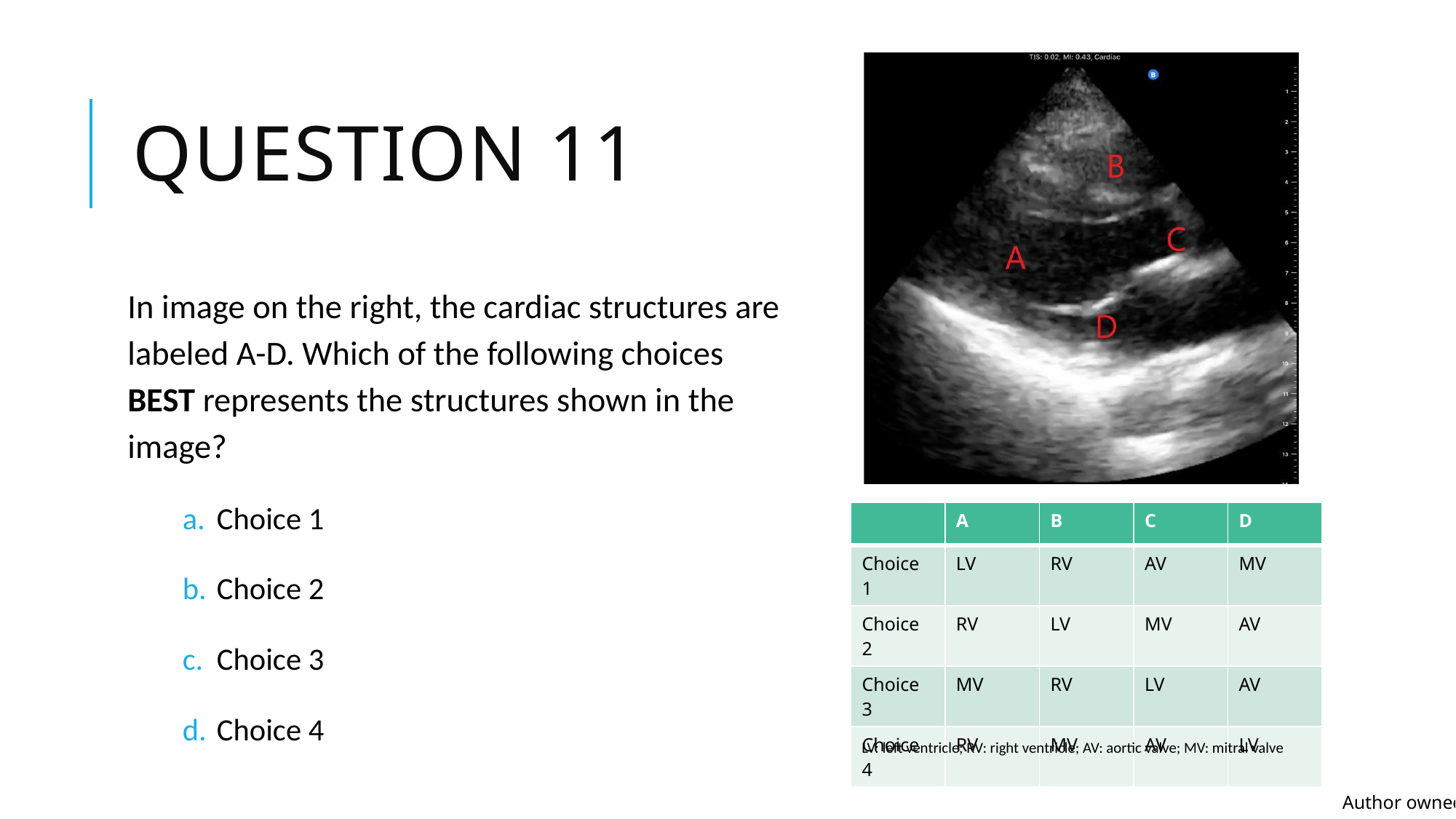

# Question 11
In image on the right, the cardiac structures are labeled A-D. Which of the following choices BEST represents the structures shown in the image?
Choice 1
Choice 2
Choice 3
Choice 4
| | A | B | C | D |
| --- | --- | --- | --- | --- |
| Choice 1 | LV | RV | AV | MV |
| Choice 2 | RV | LV | MV | AV |
| Choice 3 | MV | RV | LV | AV |
| Choice 4 | RV | MV | AV | LV |
LV: left ventricle; RV: right ventricle; AV: aortic valve; MV: mitral valve
Author owned

## Slide 13
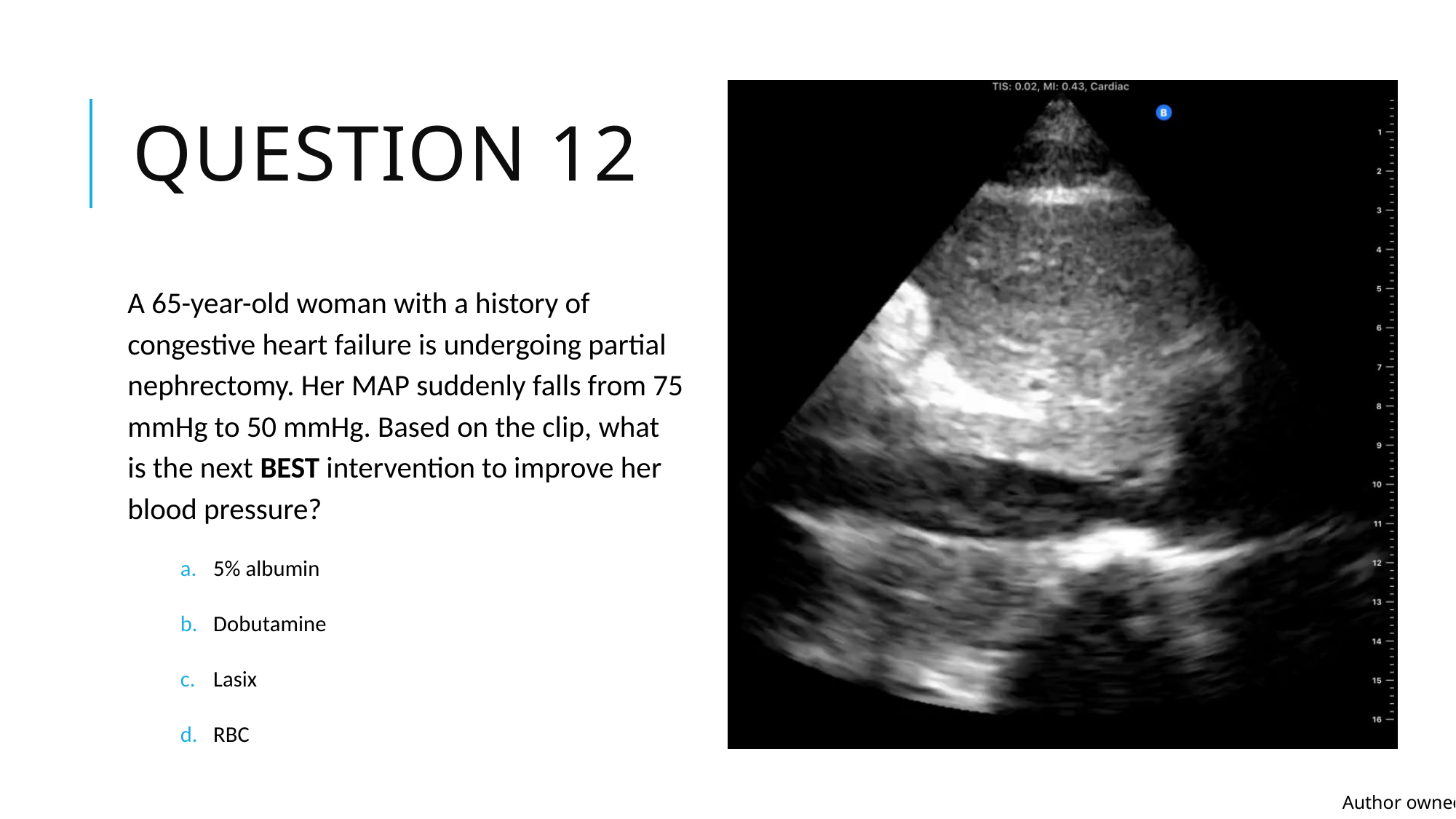

# Question 12
A 65-year-old woman with a history of congestive heart failure is undergoing partial nephrectomy. Her MAP suddenly falls from 75 mmHg to 50 mmHg. Based on the clip, what is the next BEST intervention to improve her blood pressure?
5% albumin
Dobutamine
Lasix
RBC
Author owned

## Slide 14
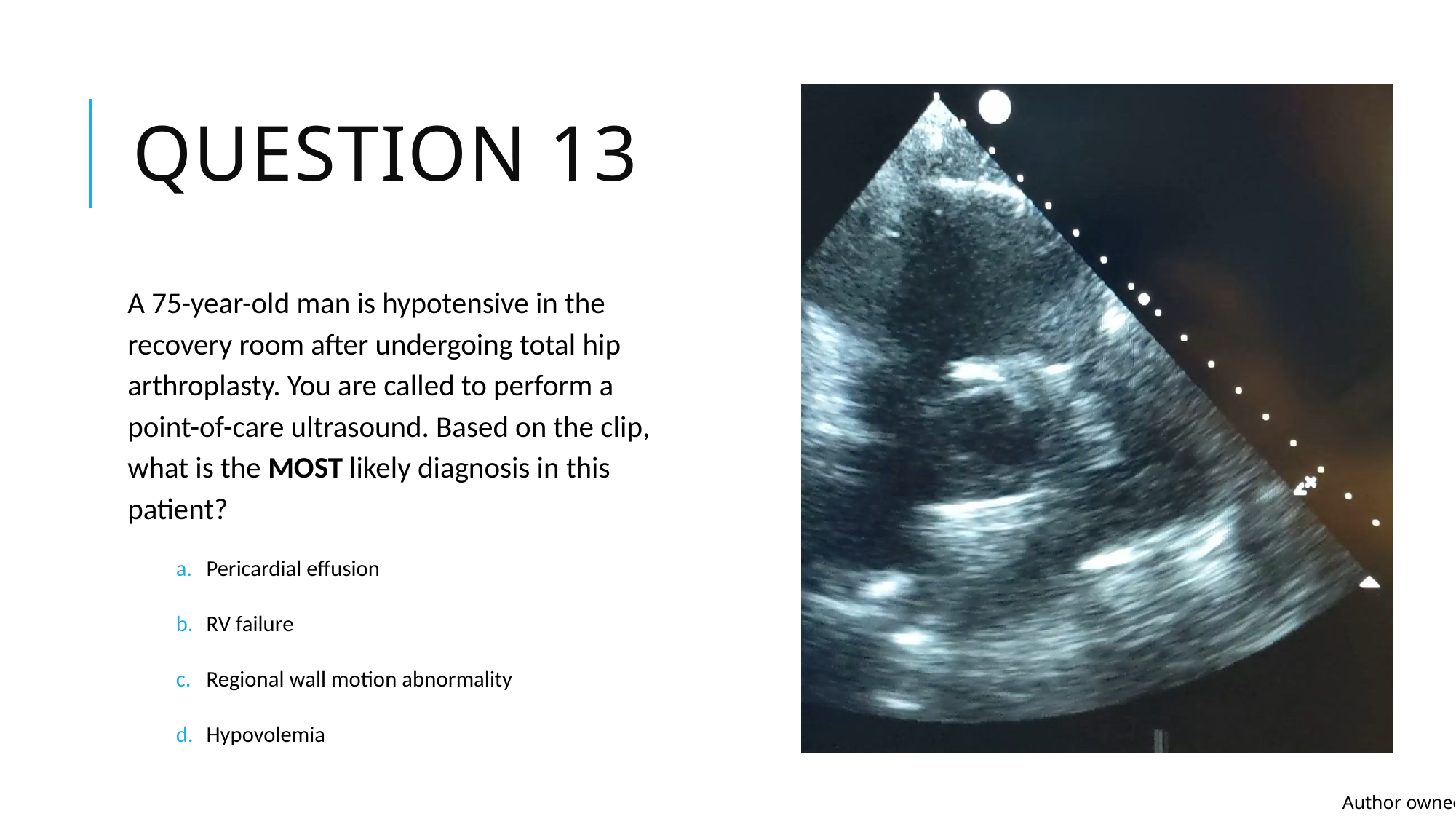

# Question 13
A 75-year-old man is hypotensive in the recovery room after undergoing total hip arthroplasty. You are called to perform a point-of-care ultrasound. Based on the clip, what is the MOST likely diagnosis in this patient?
Pericardial effusion
RV failure
Regional wall motion abnormality
Hypovolemia
Author owned

## Slide 15
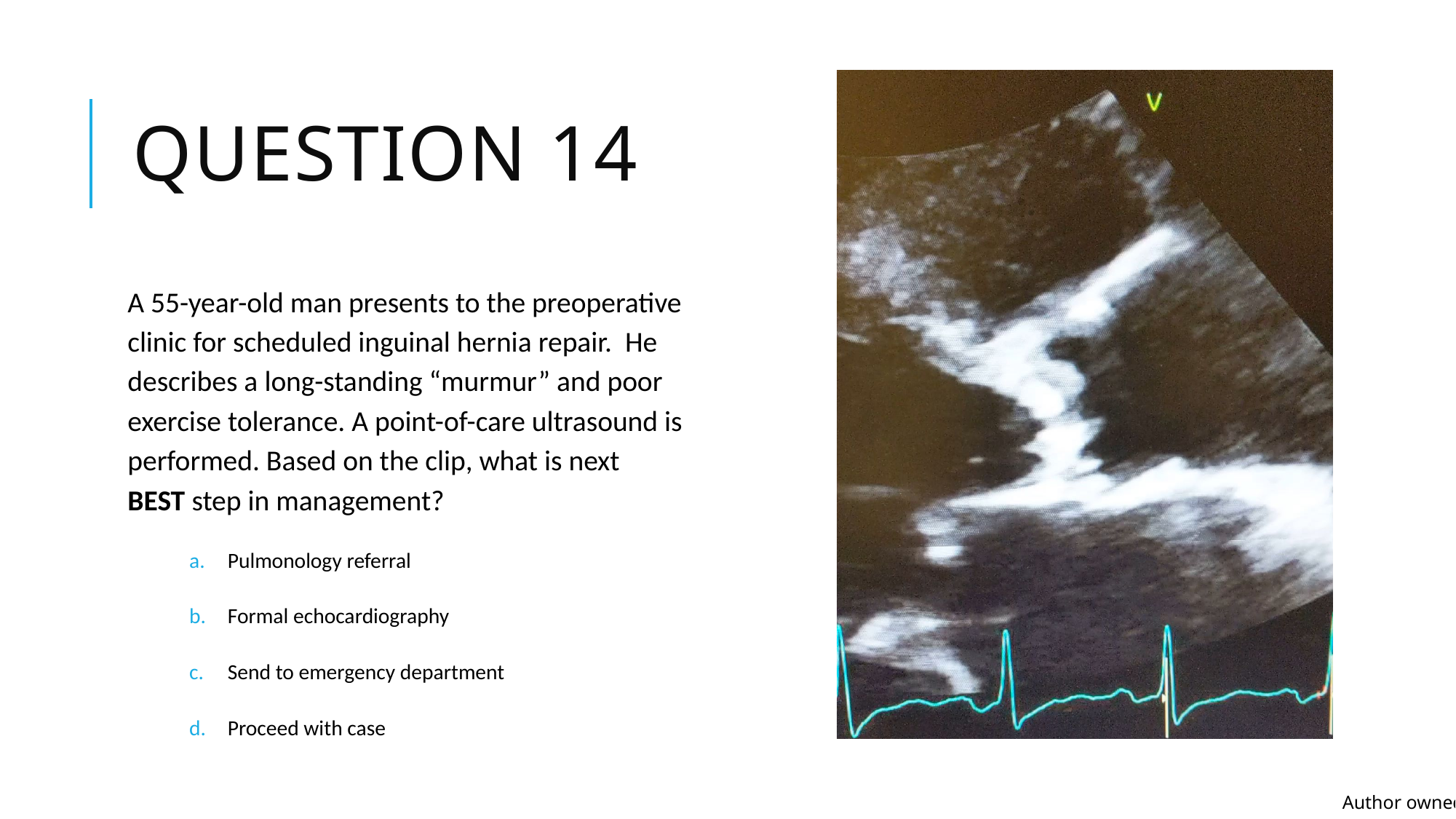

# Question 14
A 55-year-old man presents to the preoperative clinic for scheduled inguinal hernia repair. He describes a long-standing “murmur” and poor exercise tolerance. A point-of-care ultrasound is performed. Based on the clip, what is next BEST step in management?
Pulmonology referral
Formal echocardiography
Send to emergency department
Proceed with case
Author owned

## Slide 16
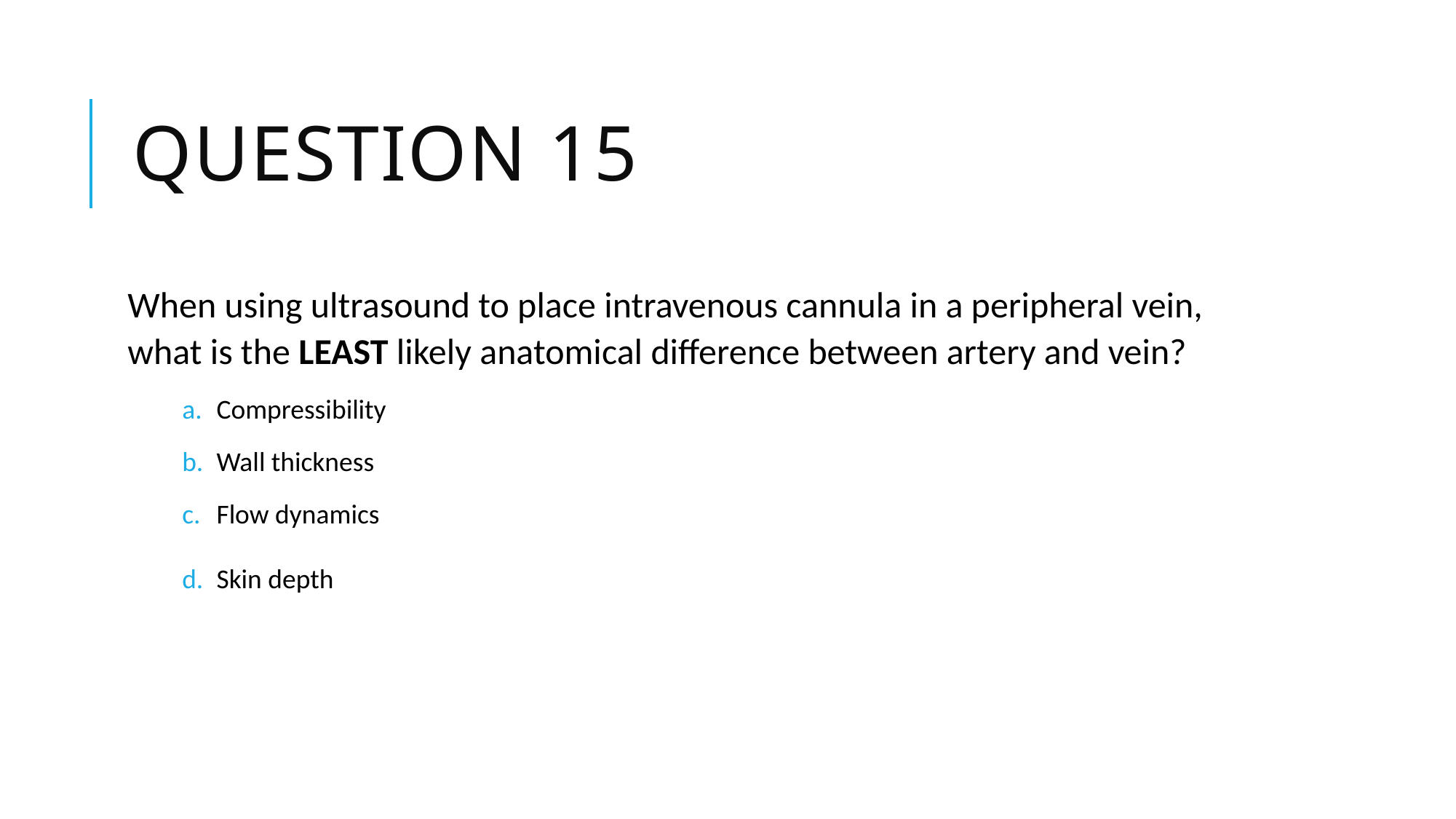

# Question 15
When using ultrasound to place intravenous cannula in a peripheral vein, what is the LEAST likely anatomical difference between artery and vein?
Compressibility
Wall thickness
Flow dynamics
Skin depth

## Slide 17
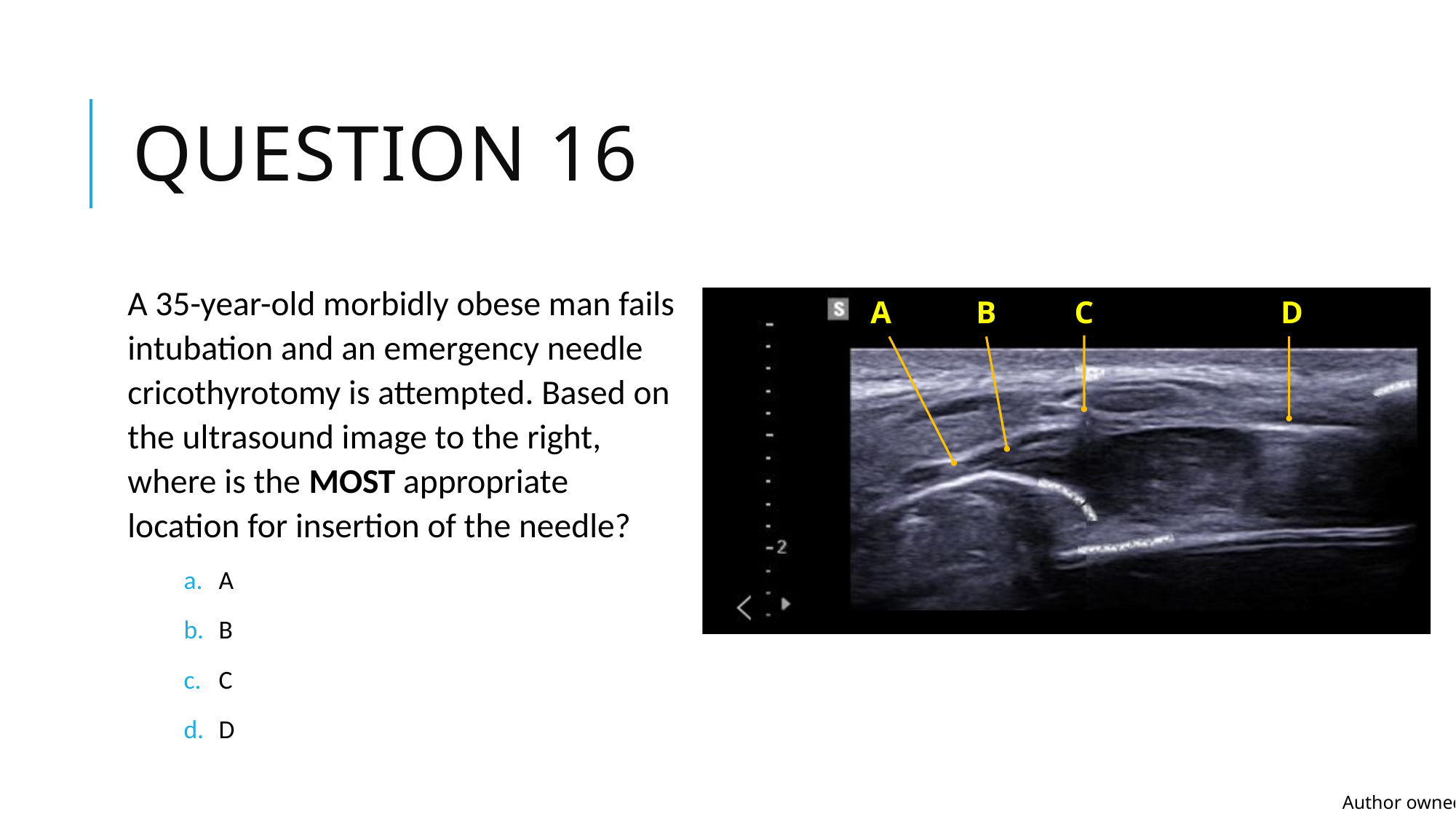

# Question 16
A 35-year-old morbidly obese man fails intubation and an emergency needle cricothyrotomy is attempted. Based on the ultrasound image to the right, where is the MOST appropriate location for insertion of the needle?
A
B
C
D
A
B
C
D
Author owned

## Slide 18
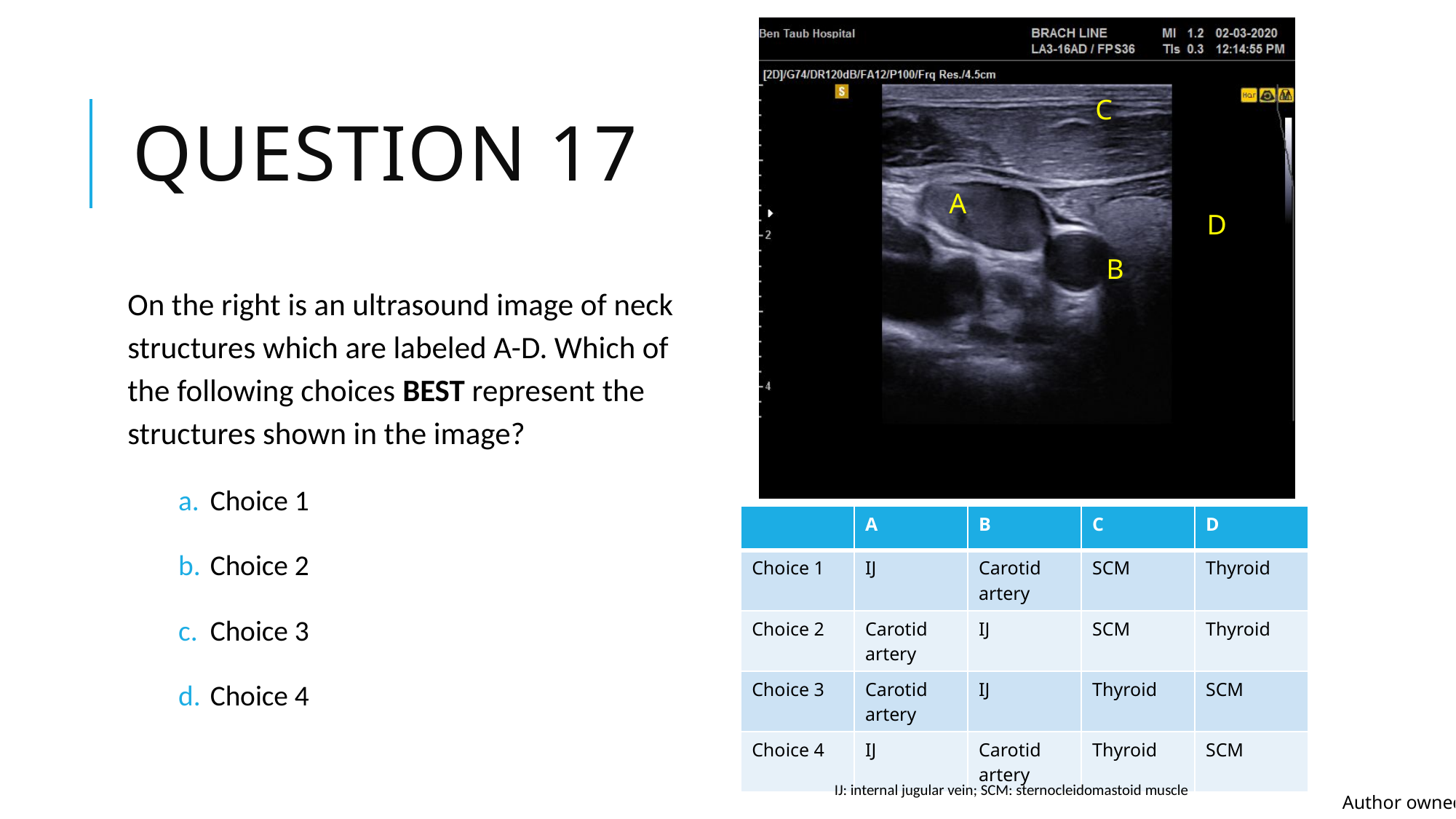

C
A
D
B
# Question 17
On the right is an ultrasound image of neck structures which are labeled A-D. Which of the following choices BEST represent the structures shown in the image?
Choice 1
Choice 2
Choice 3
Choice 4
| | A | B | C | D |
| --- | --- | --- | --- | --- |
| Choice 1 | IJ | Carotid artery | SCM | Thyroid |
| Choice 2 | Carotid artery | IJ | SCM | Thyroid |
| Choice 3 | Carotid artery | IJ | Thyroid | SCM |
| Choice 4 | IJ | Carotid artery | Thyroid | SCM |
IJ: internal jugular vein; SCM: sternocleidomastoid muscle
Author owned

## Slide 19
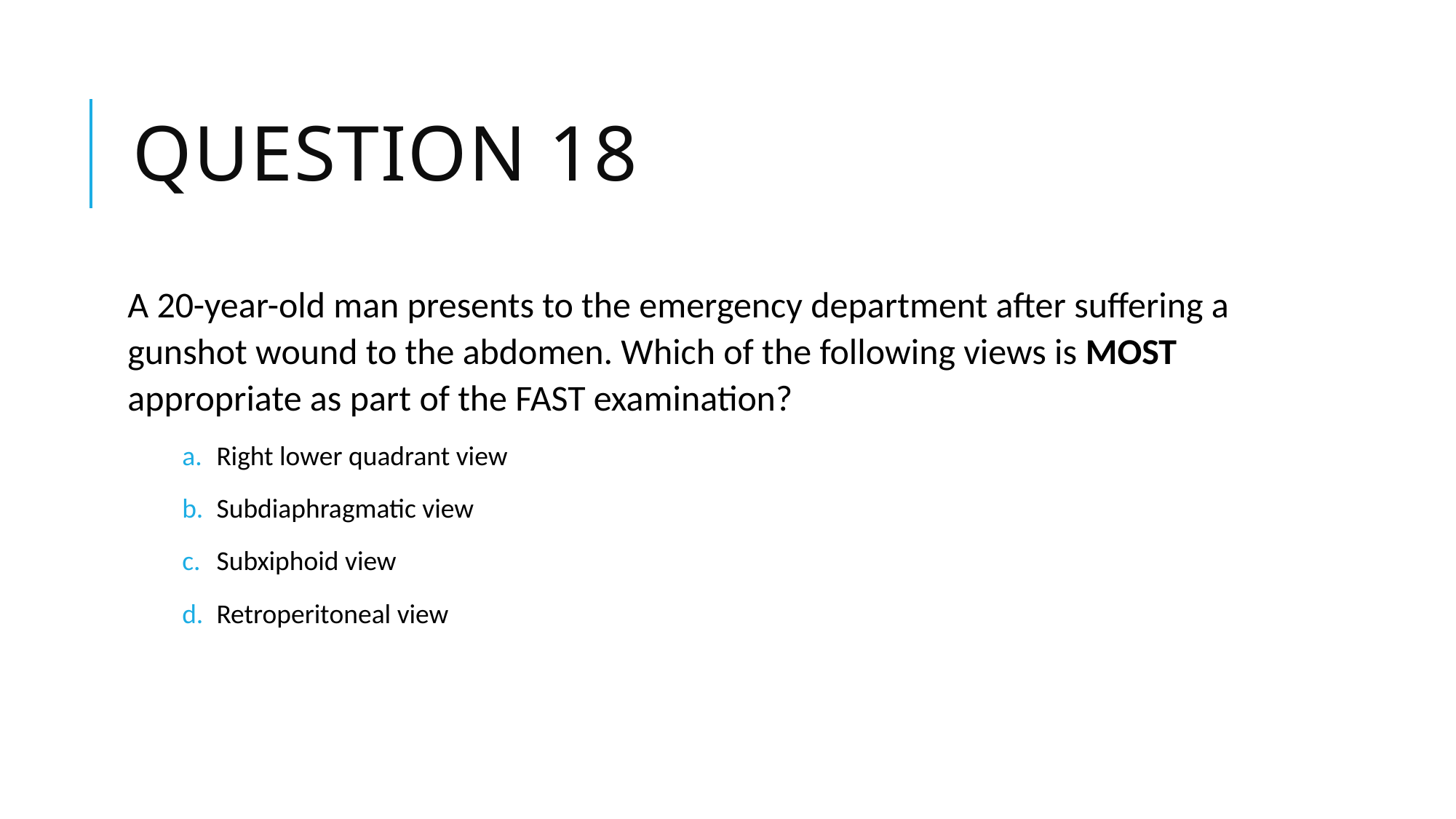

# Question 18
A 20-year-old man presents to the emergency department after suffering a gunshot wound to the abdomen. Which of the following views is MOST appropriate as part of the FAST examination?
Right lower quadrant view
Subdiaphragmatic view
Subxiphoid view
Retroperitoneal view

## Slide 20
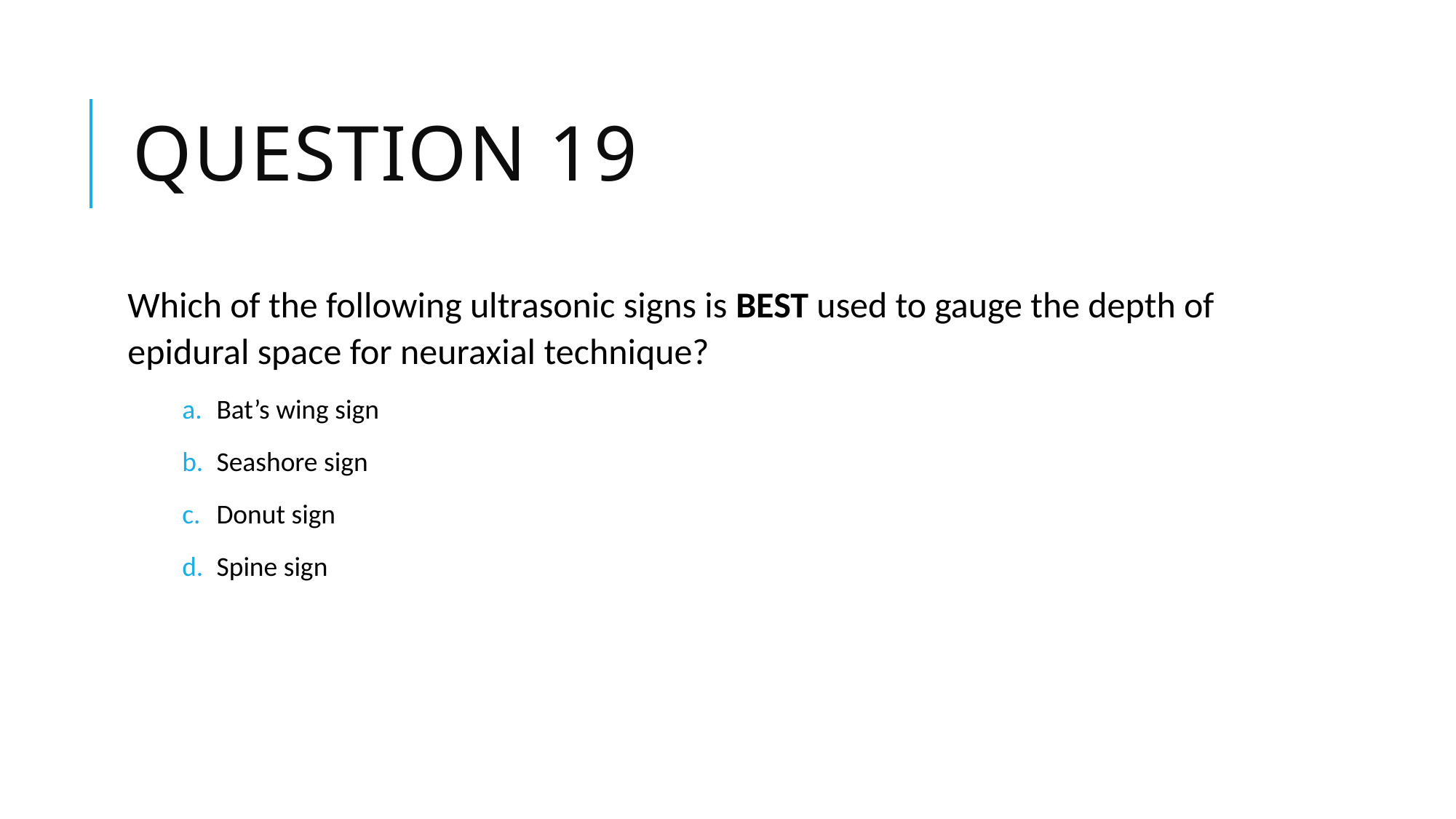

# Question 19
Which of the following ultrasonic signs is BEST used to gauge the depth of epidural space for neuraxial technique?
Bat’s wing sign
Seashore sign
Donut sign
Spine sign

## Slide 21
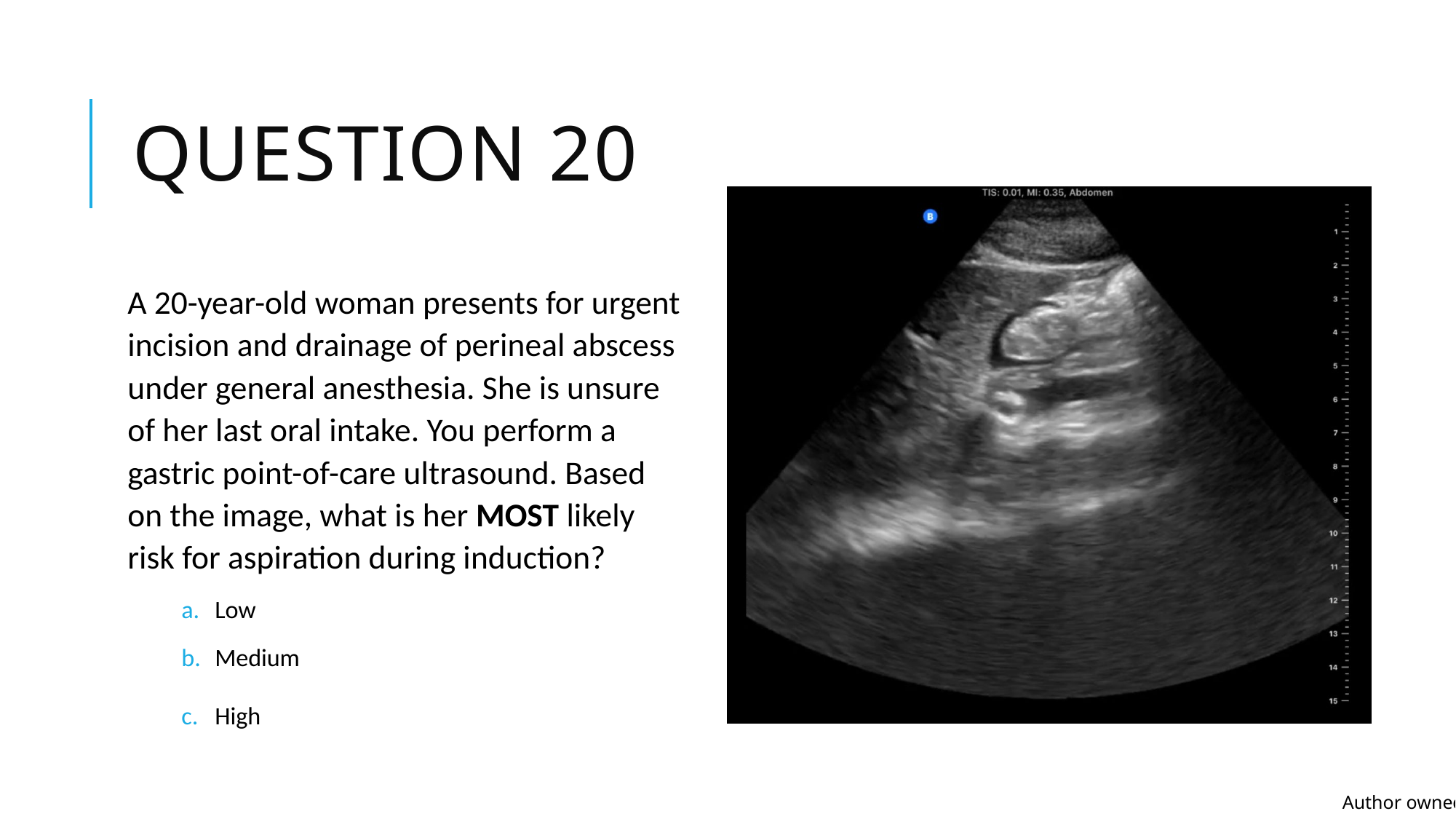

# Question 20
A 20-year-old woman presents for urgent incision and drainage of perineal abscess under general anesthesia. She is unsure of her last oral intake. You perform a gastric point-of-care ultrasound. Based on the image, what is her MOST likely risk for aspiration during induction?
Low
Medium
High
Author owned
