# Supplementary material for: An Introductory Point-of-Care Ultrasound Curriculum for an Anesthesiology Residency Program
Source: MedEdPORTAL. 2022 Dec 23;18:11291. doi: 10.15766/mep_2374-8265.11291 (PMC9780414; doi:10.15766/mep_2374-8265.11291)
Supplement: Supplementary file 1 — Ultrasound Basics.pptxLung Ultrasound.pptxCardiac Ultrasound.pptxVascular Access Ultrasound.pptxAirway Ultrasound.pptxAbdominal Ultrasound.pptxNeuraxial Ultrasound.pptxChecklist for POCUS Scanning.docxPOCUS CA1 Curriculum Pretest.pptxPOCUS CA1 Curriculum Posttest.pptxPOCUS Survey.docx [file mep_2374-8265.11291-s001.zip › J. POCUS CA1 Curriculum Posttest.pptx]

## Slide 1
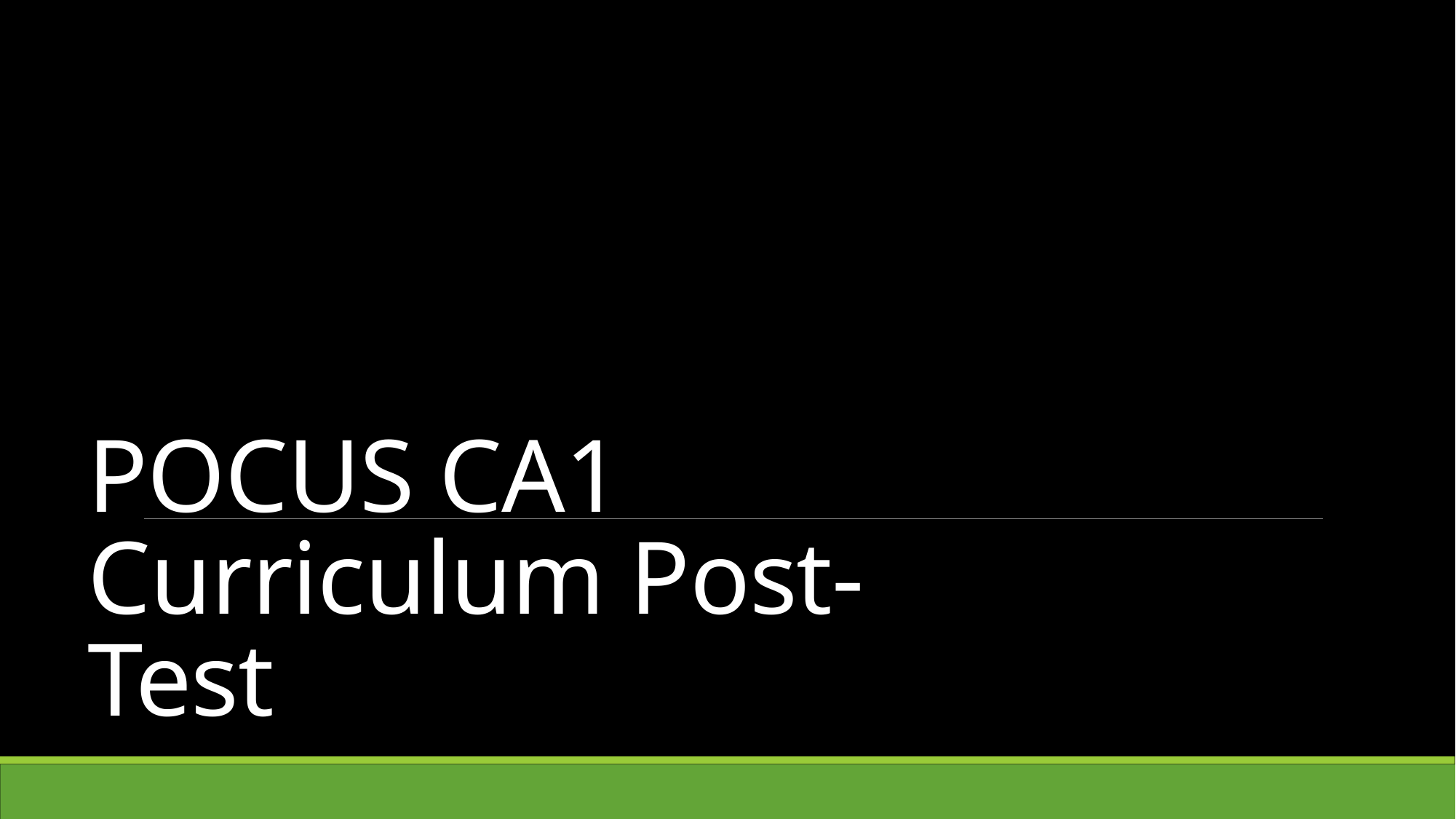

# POCUS CA1 Curriculum Post-Test

## Slide 2
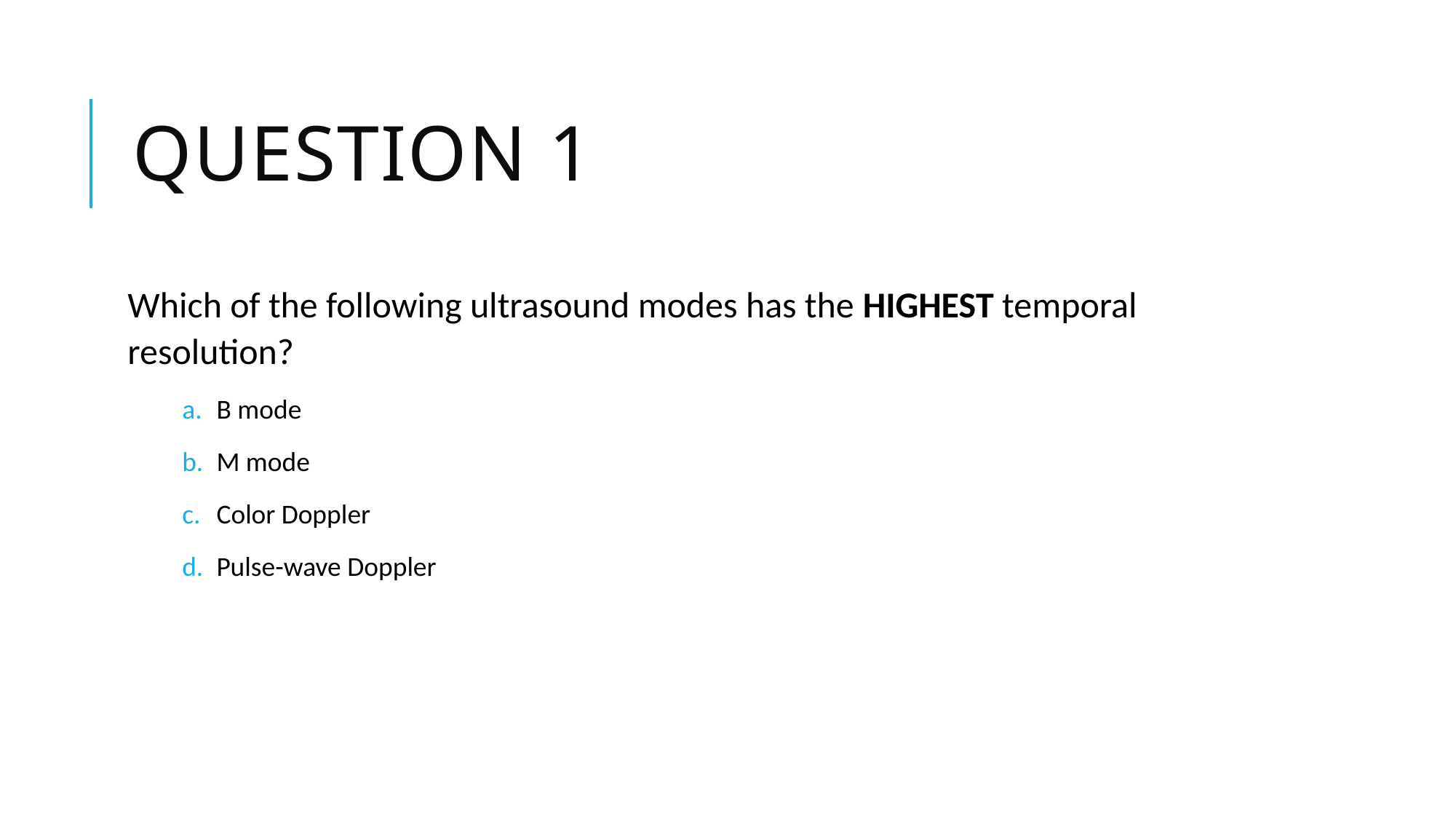

# Question 1
Which of the following ultrasound modes has the HIGHEST temporal resolution?
B mode
M mode
Color Doppler
Pulse-wave Doppler

## Slide 3
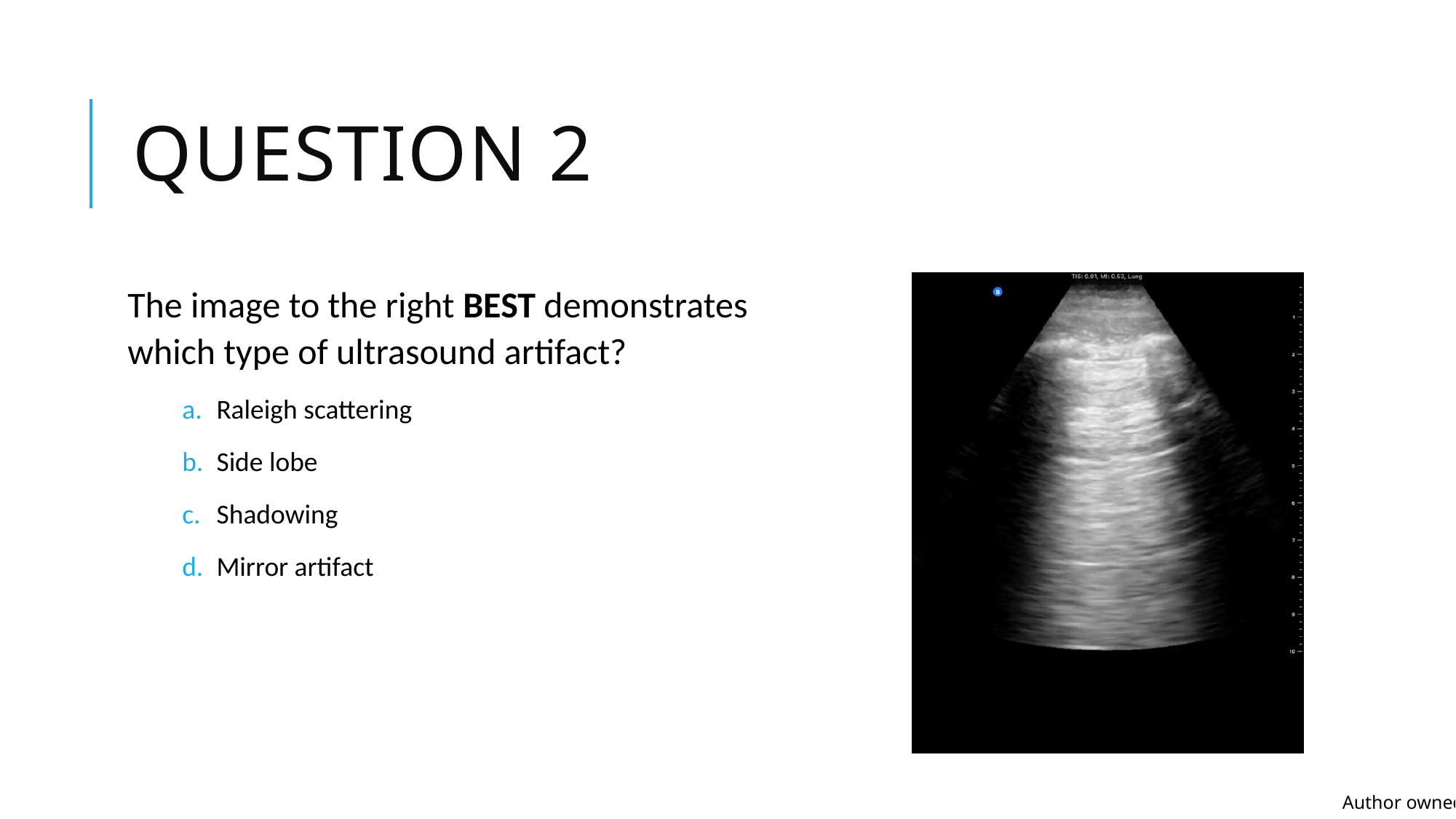

# Question 2
The image to the right BEST demonstrates which type of ultrasound artifact?
Raleigh scattering
Side lobe
Shadowing
Mirror artifact
Author owned

## Slide 4
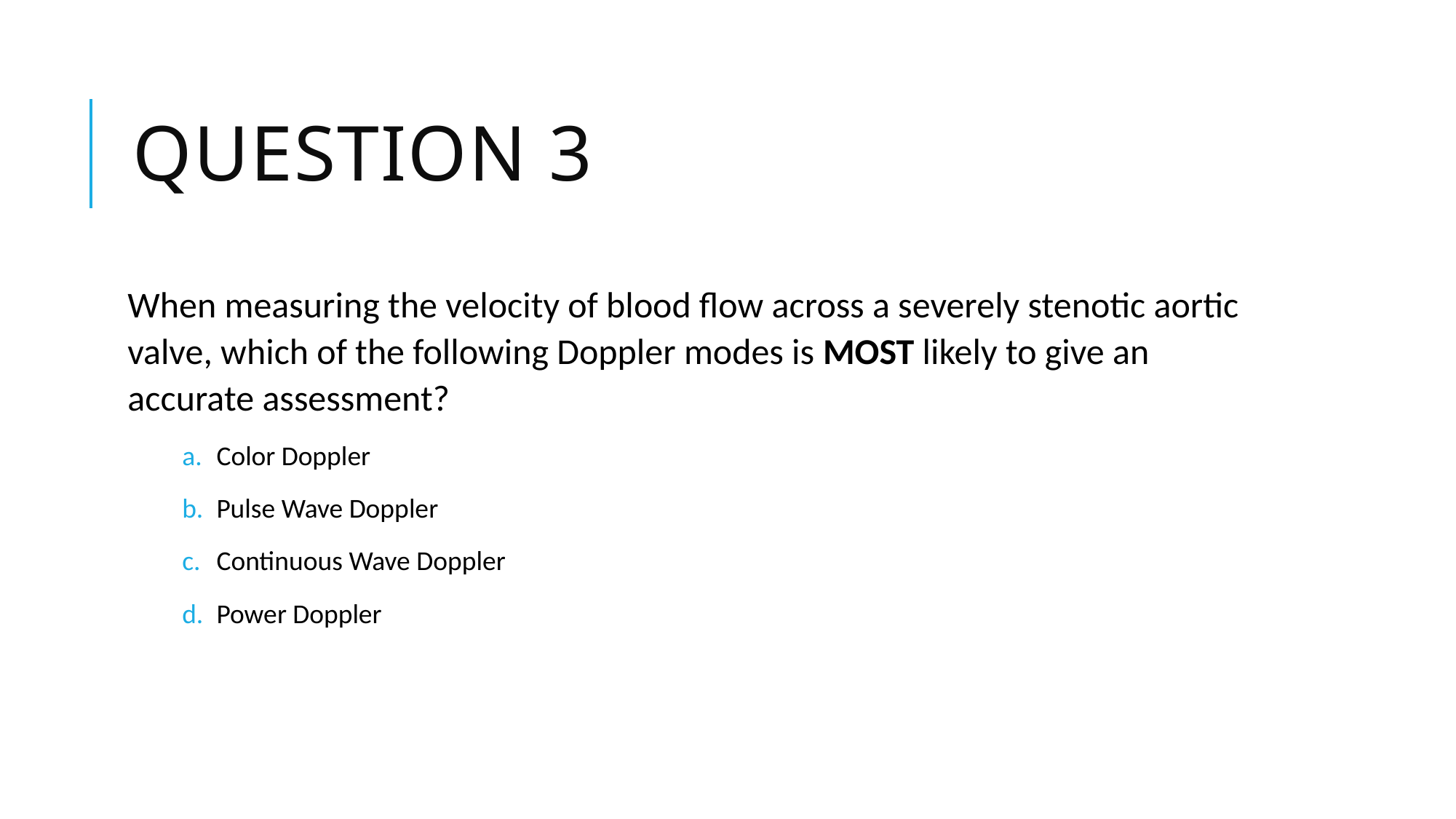

# Question 3
When measuring the velocity of blood flow across a severely stenotic aortic valve, which of the following Doppler modes is MOST likely to give an accurate assessment?
Color Doppler
Pulse Wave Doppler
Continuous Wave Doppler
Power Doppler

## Slide 5
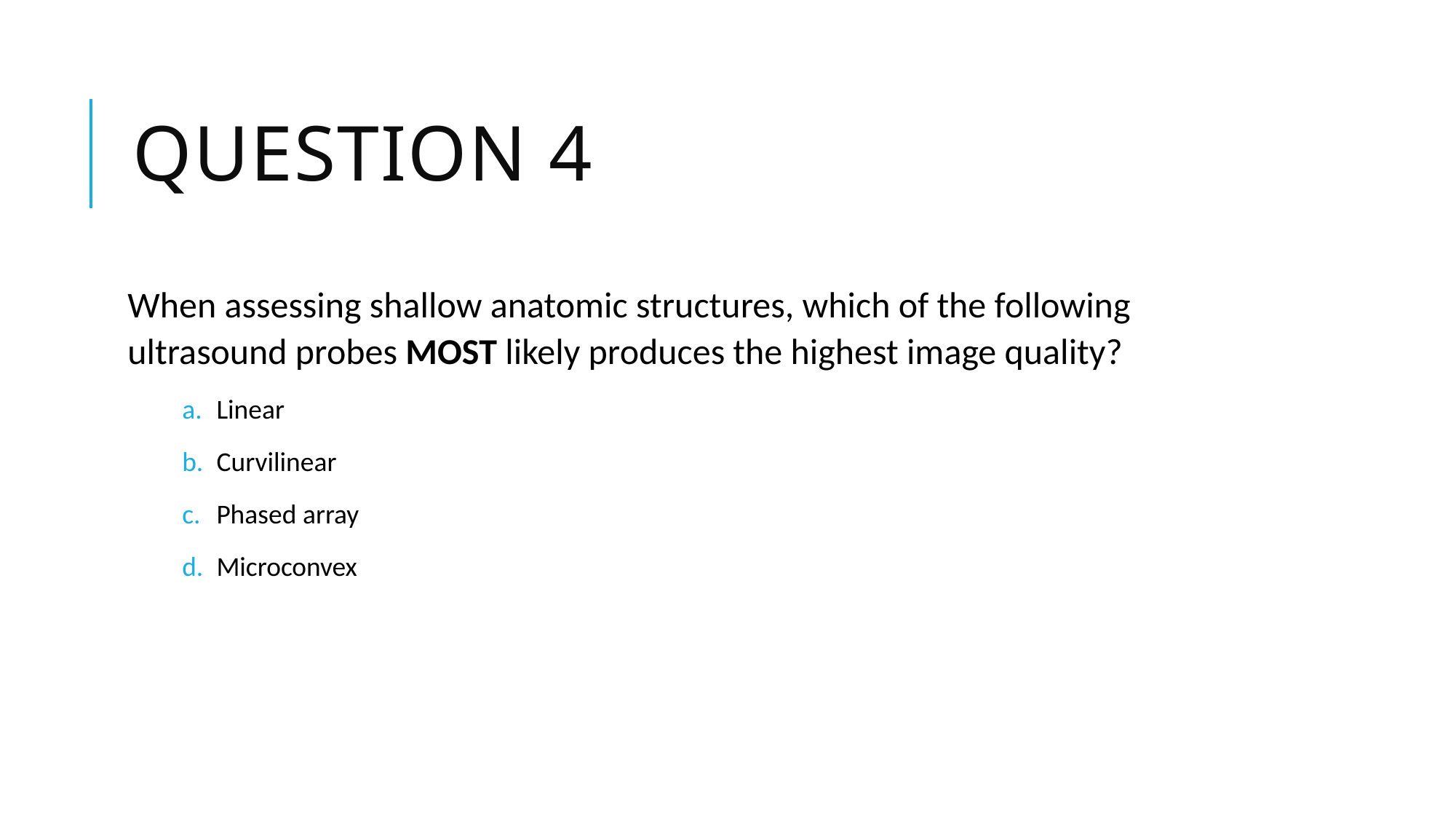

# Question 4
When assessing shallow anatomic structures, which of the following ultrasound probes MOST likely produces the highest image quality?
Linear
Curvilinear
Phased array
Microconvex

## Slide 6
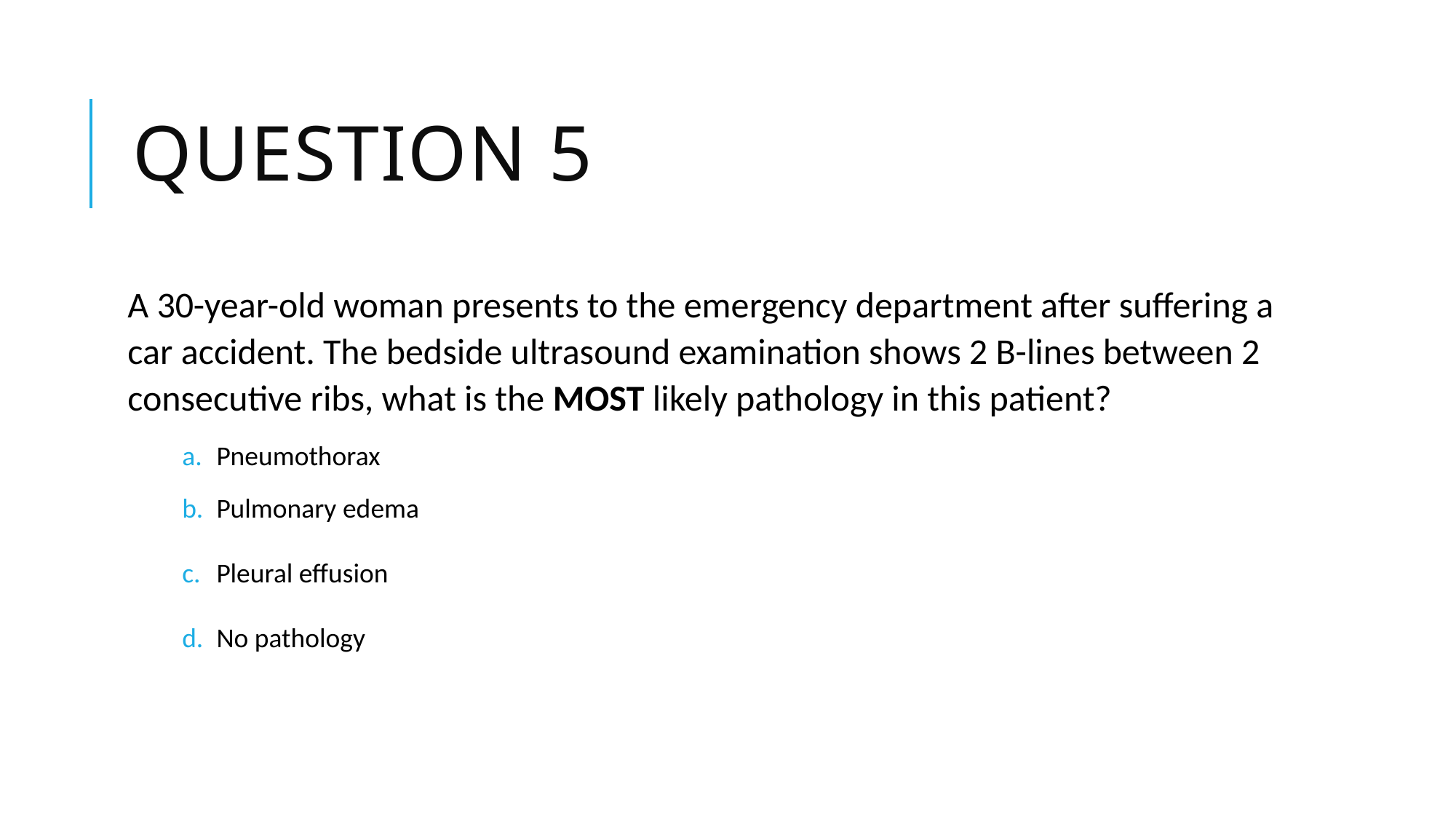

# Question 5
A 30-year-old woman presents to the emergency department after suffering a car accident. The bedside ultrasound examination shows 2 B-lines between 2 consecutive ribs, what is the MOST likely pathology in this patient?
Pneumothorax
Pulmonary edema
Pleural effusion
No pathology

## Slide 7
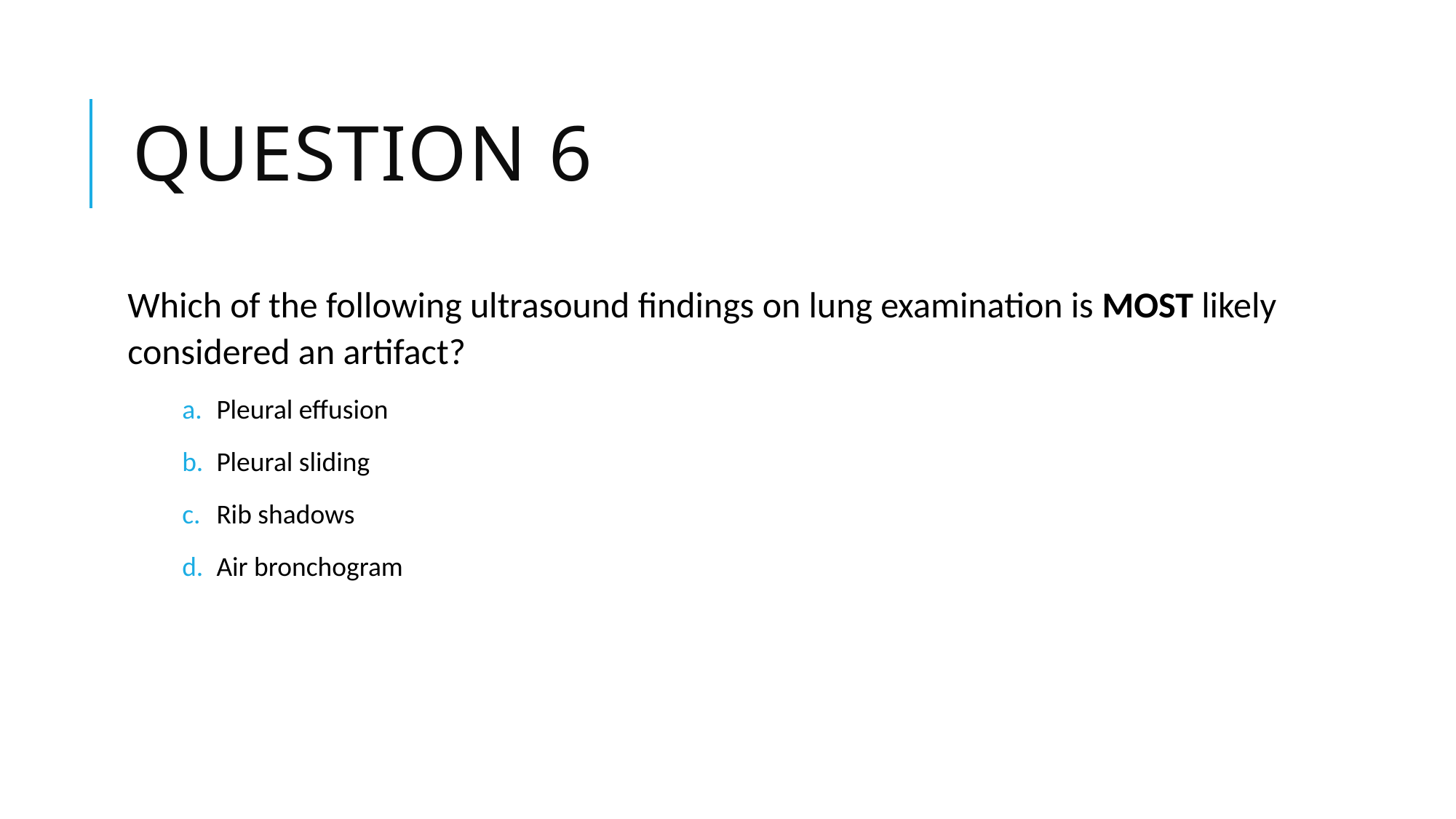

# Question 6
Which of the following ultrasound findings on lung examination is MOST likely considered an artifact?
Pleural effusion
Pleural sliding
Rib shadows
Air bronchogram

## Slide 8
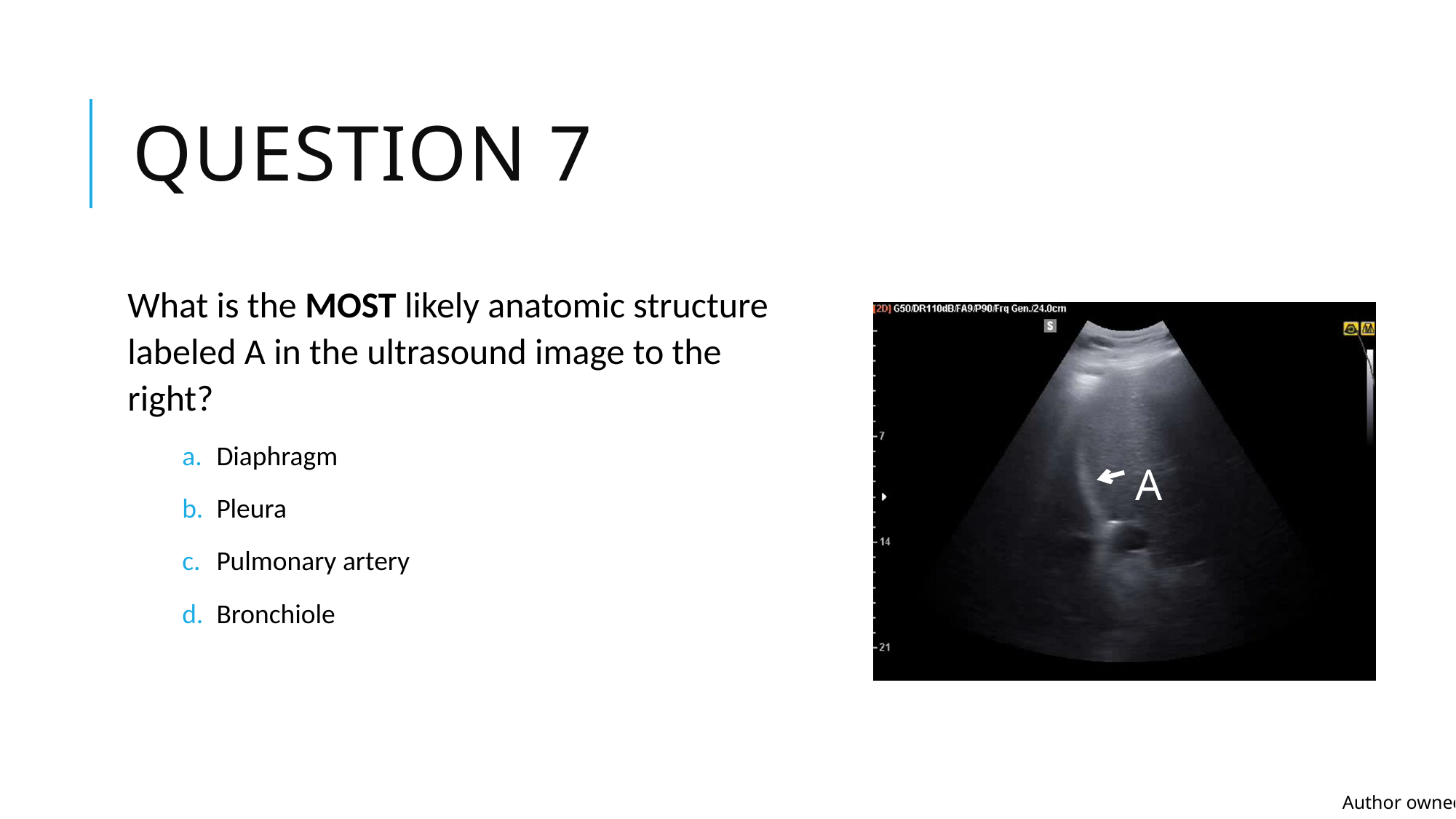

# Question 7
What is the MOST likely anatomic structure labeled A in the ultrasound image to the right?
Diaphragm
Pleura
Pulmonary artery
Bronchiole
A
Author owned

## Slide 9
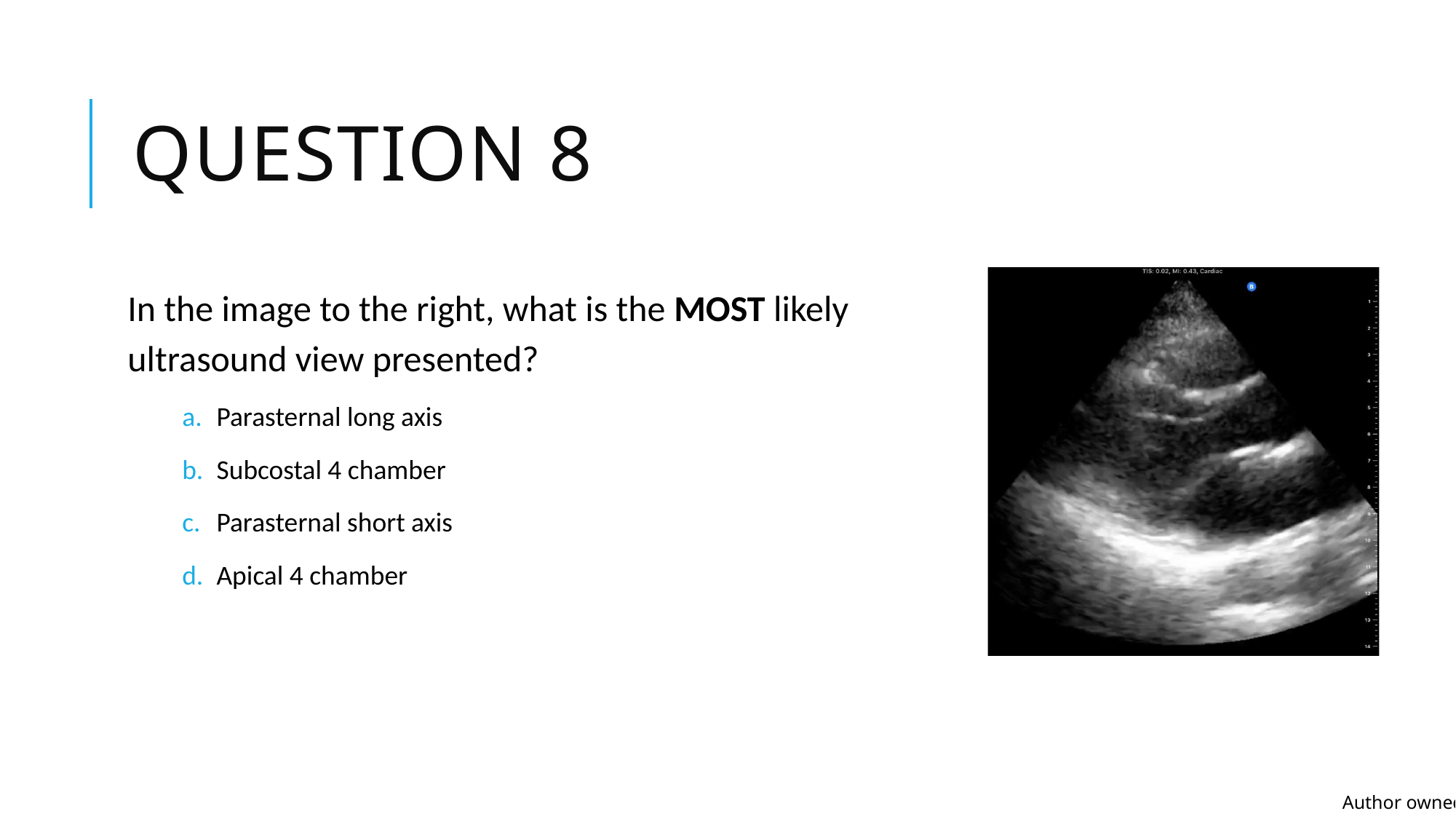

# Question 8
In the image to the right, what is the MOST likely ultrasound view presented?
Parasternal long axis
Subcostal 4 chamber
Parasternal short axis
Apical 4 chamber
Author owned

## Slide 10
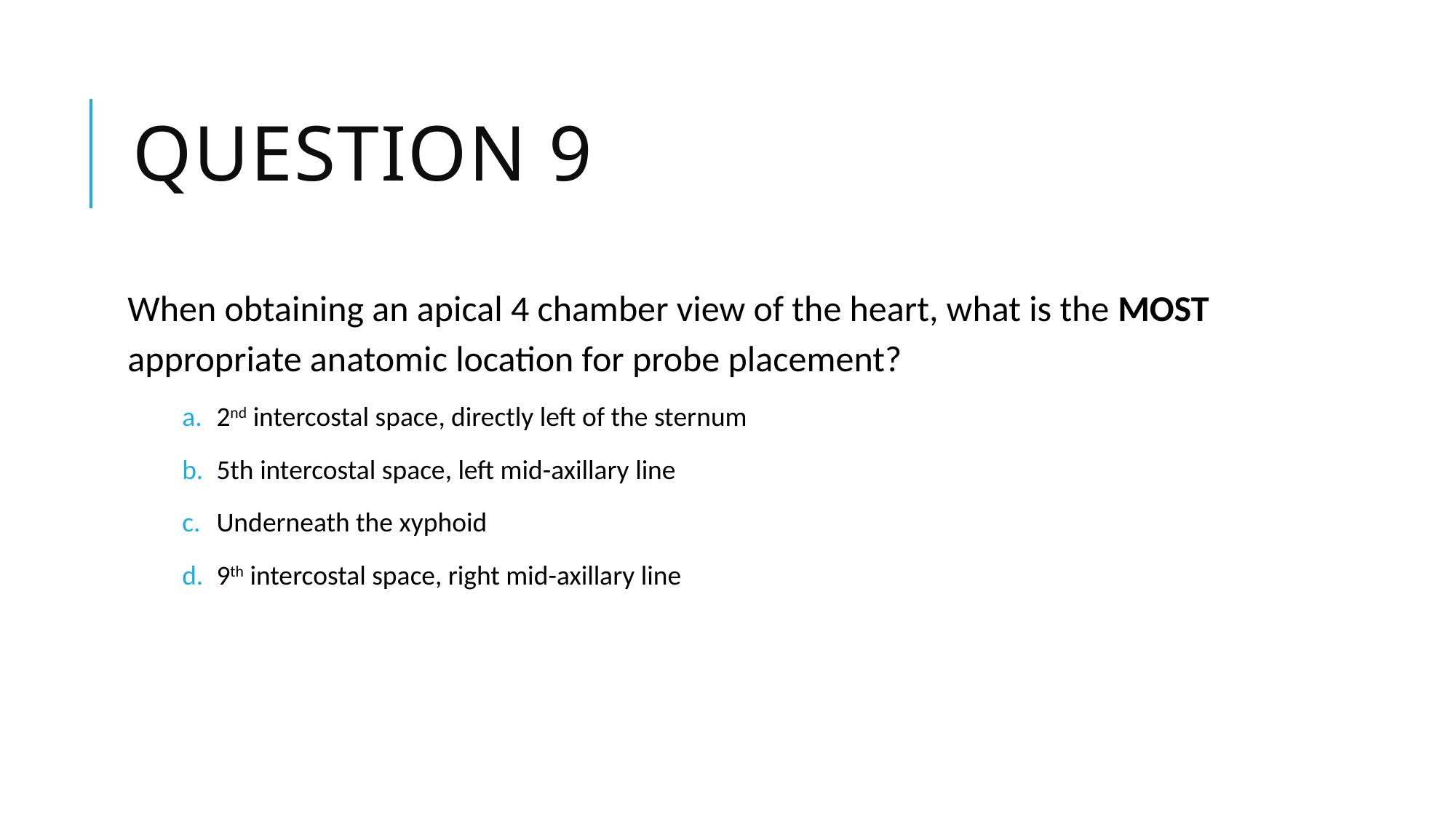

# Question 9
When obtaining an apical 4 chamber view of the heart, what is the MOST appropriate anatomic location for probe placement?
2nd intercostal space, directly left of the sternum
5th intercostal space, left mid-axillary line
Underneath the xyphoid
9th intercostal space, right mid-axillary line

## Slide 11
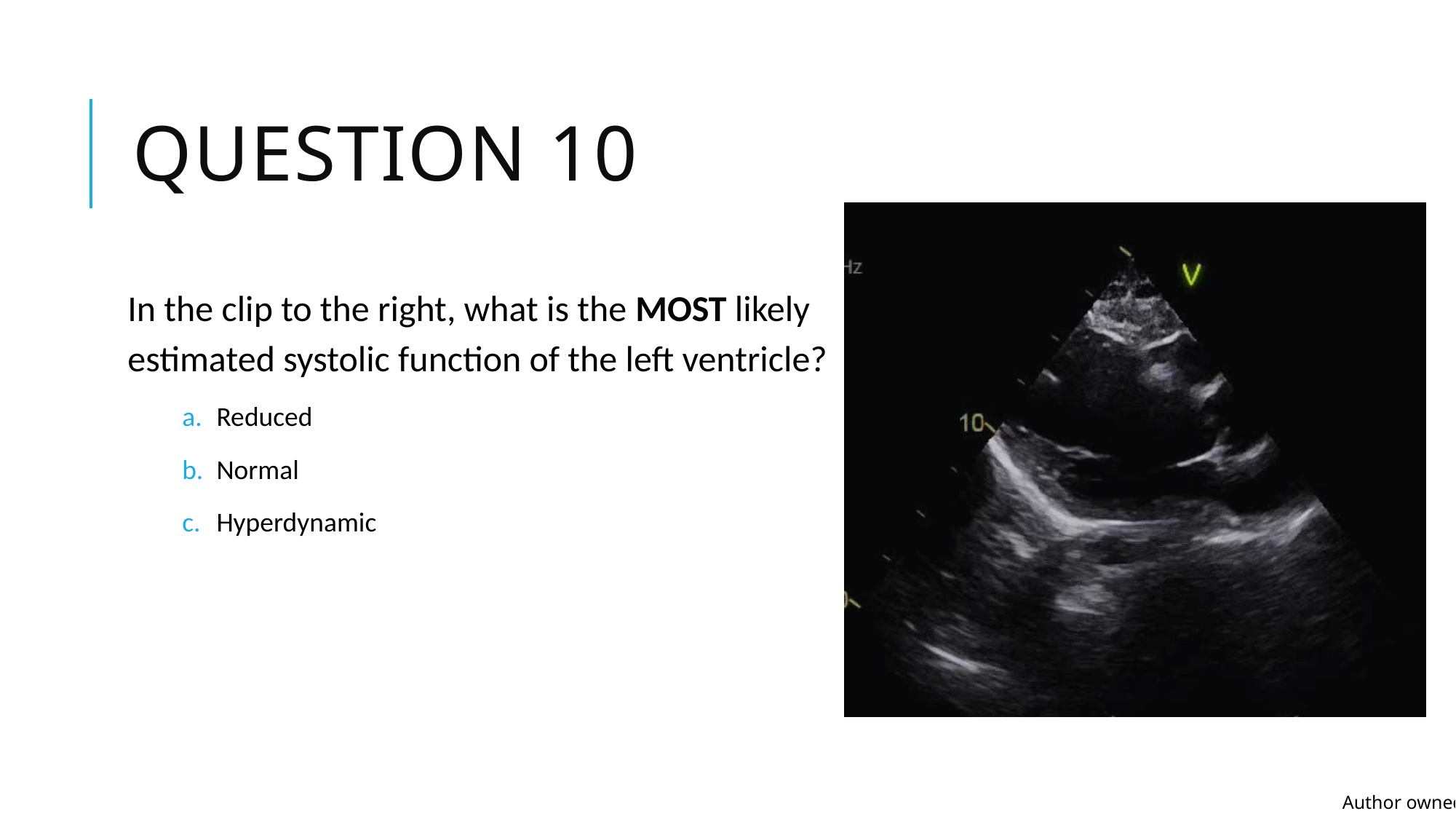

# Question 10
In the clip to the right, what is the MOST likely estimated systolic function of the left ventricle?
Reduced
Normal
Hyperdynamic
Author owned

## Slide 12
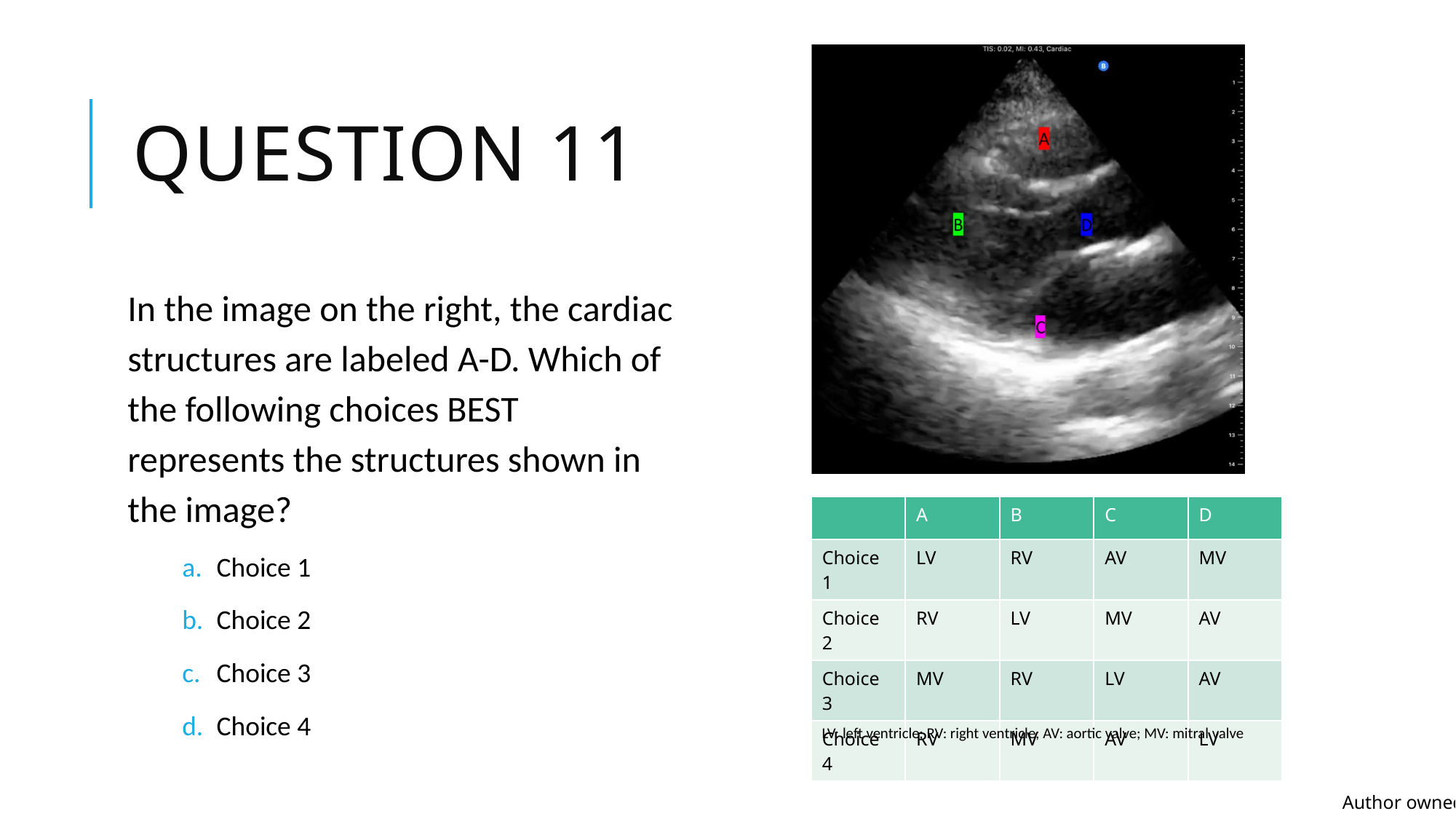

# Question 11
In the image on the right, the cardiac structures are labeled A-D. Which of the following choices BEST represents the structures shown in the image?
Choice 1
Choice 2
Choice 3
Choice 4
| | A | B | C | D |
| --- | --- | --- | --- | --- |
| Choice 1 | LV | RV | AV | MV |
| Choice 2 | RV | LV | MV | AV |
| Choice 3 | MV | RV | LV | AV |
| Choice 4 | RV | MV | AV | LV |
LV: left ventricle; RV: right ventricle; AV: aortic valve; MV: mitral valve
Author owned

## Slide 13
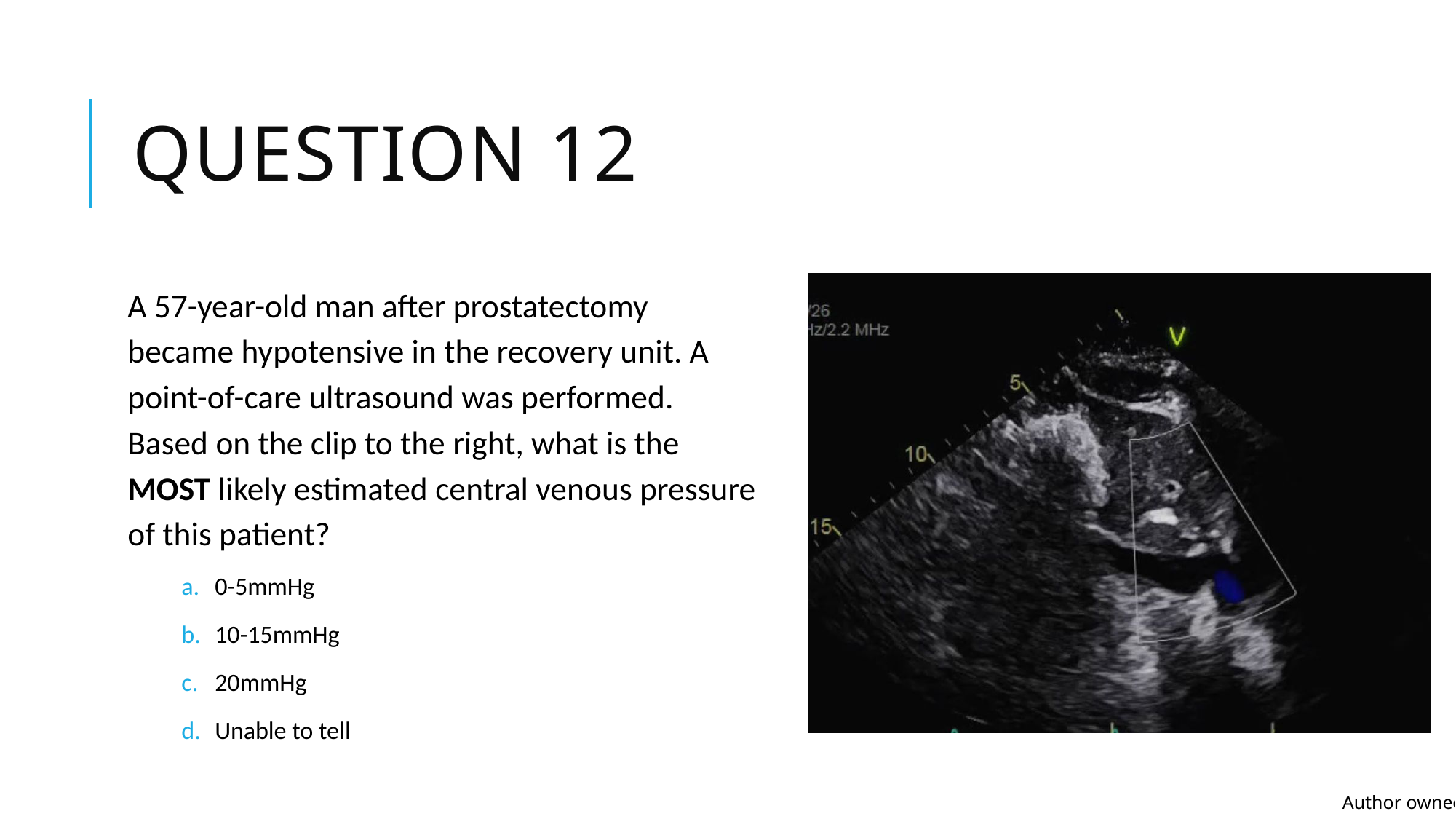

# Question 12
A 57-year-old man after prostatectomy became hypotensive in the recovery unit. A point-of-care ultrasound was performed. Based on the clip to the right, what is the MOST likely estimated central venous pressure of this patient?
0-5mmHg
10-15mmHg
20mmHg
Unable to tell
Author owned

## Slide 14
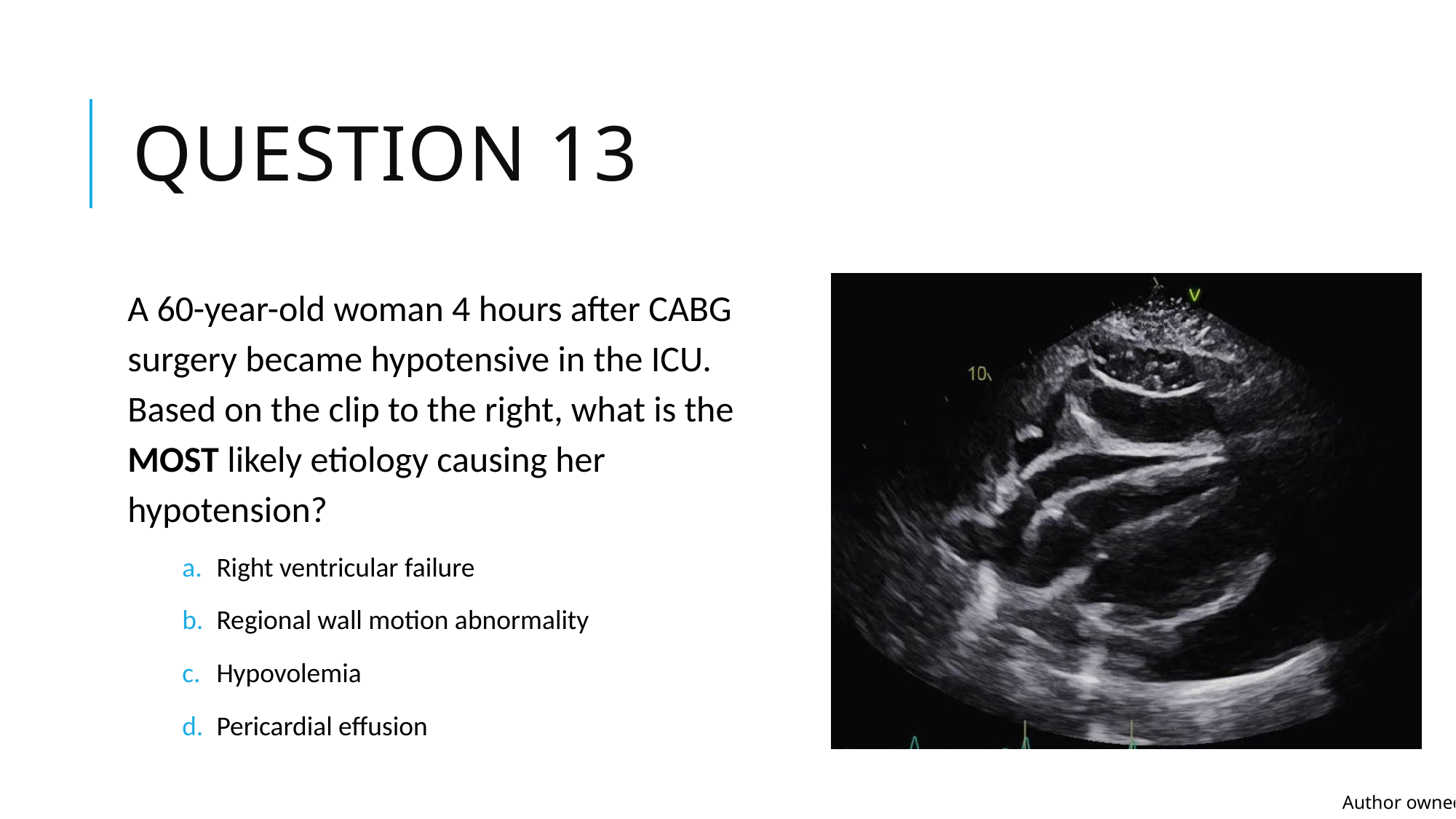

# Question 13
A 60-year-old woman 4 hours after CABG surgery became hypotensive in the ICU. Based on the clip to the right, what is the MOST likely etiology causing her hypotension?
Right ventricular failure
Regional wall motion abnormality
Hypovolemia
Pericardial effusion
Author owned

## Slide 15
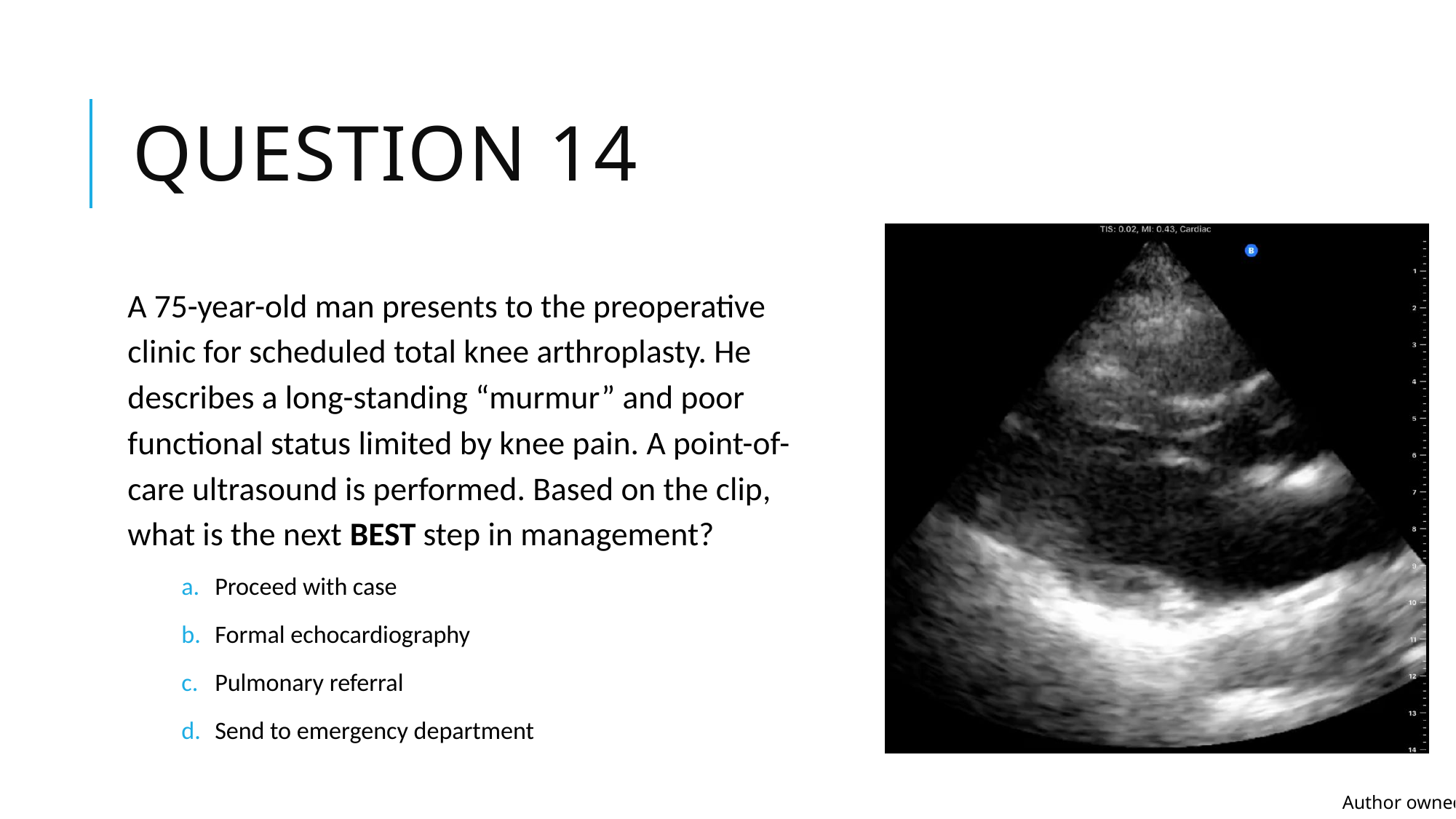

# Question 14
A 75-year-old man presents to the preoperative clinic for scheduled total knee arthroplasty. He describes a long-standing “murmur” and poor functional status limited by knee pain. A point-of-care ultrasound is performed. Based on the clip, what is the next BEST step in management?
Proceed with case
Formal echocardiography
Pulmonary referral
Send to emergency department
Author owned

## Slide 16
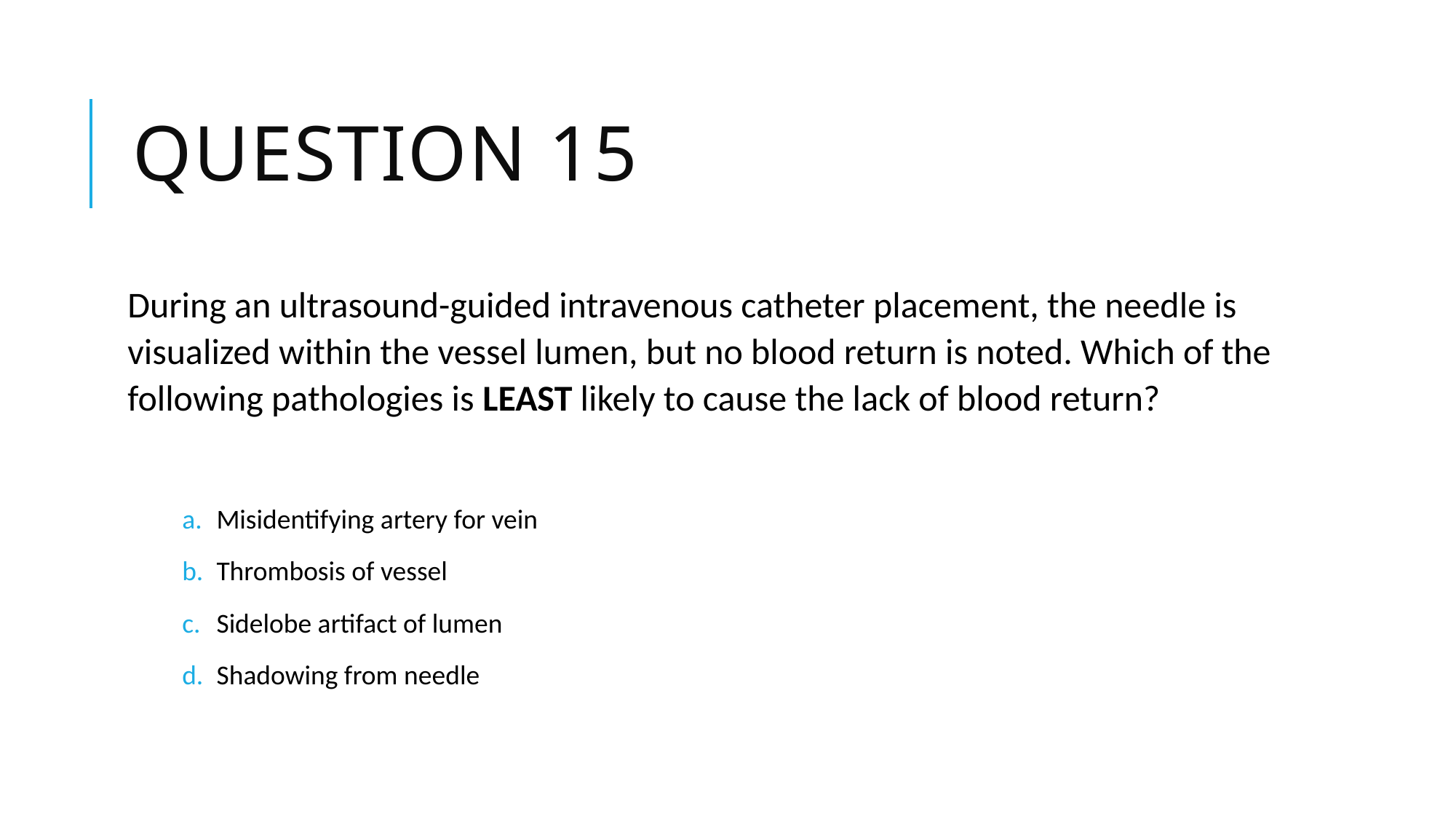

# Question 15
During an ultrasound-guided intravenous catheter placement, the needle is visualized within the vessel lumen, but no blood return is noted. Which of the following pathologies is LEAST likely to cause the lack of blood return?
Misidentifying artery for vein
Thrombosis of vessel
Sidelobe artifact of lumen
Shadowing from needle

## Slide 17
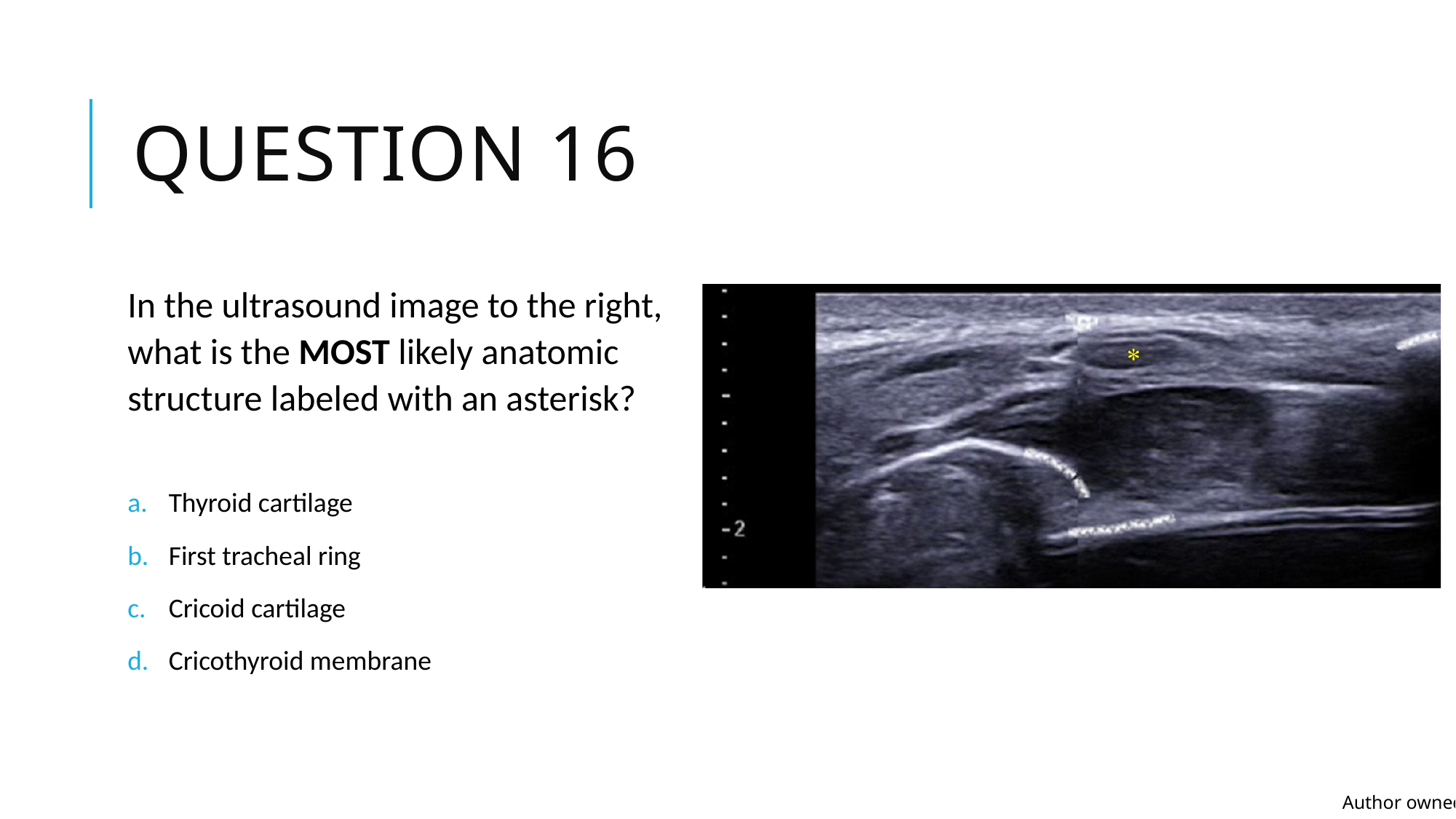

# Question 16
In the ultrasound image to the right, what is the MOST likely anatomic structure labeled with an asterisk?
Thyroid cartilage
First tracheal ring
Cricoid cartilage
Cricothyroid membrane
Author owned

## Slide 18
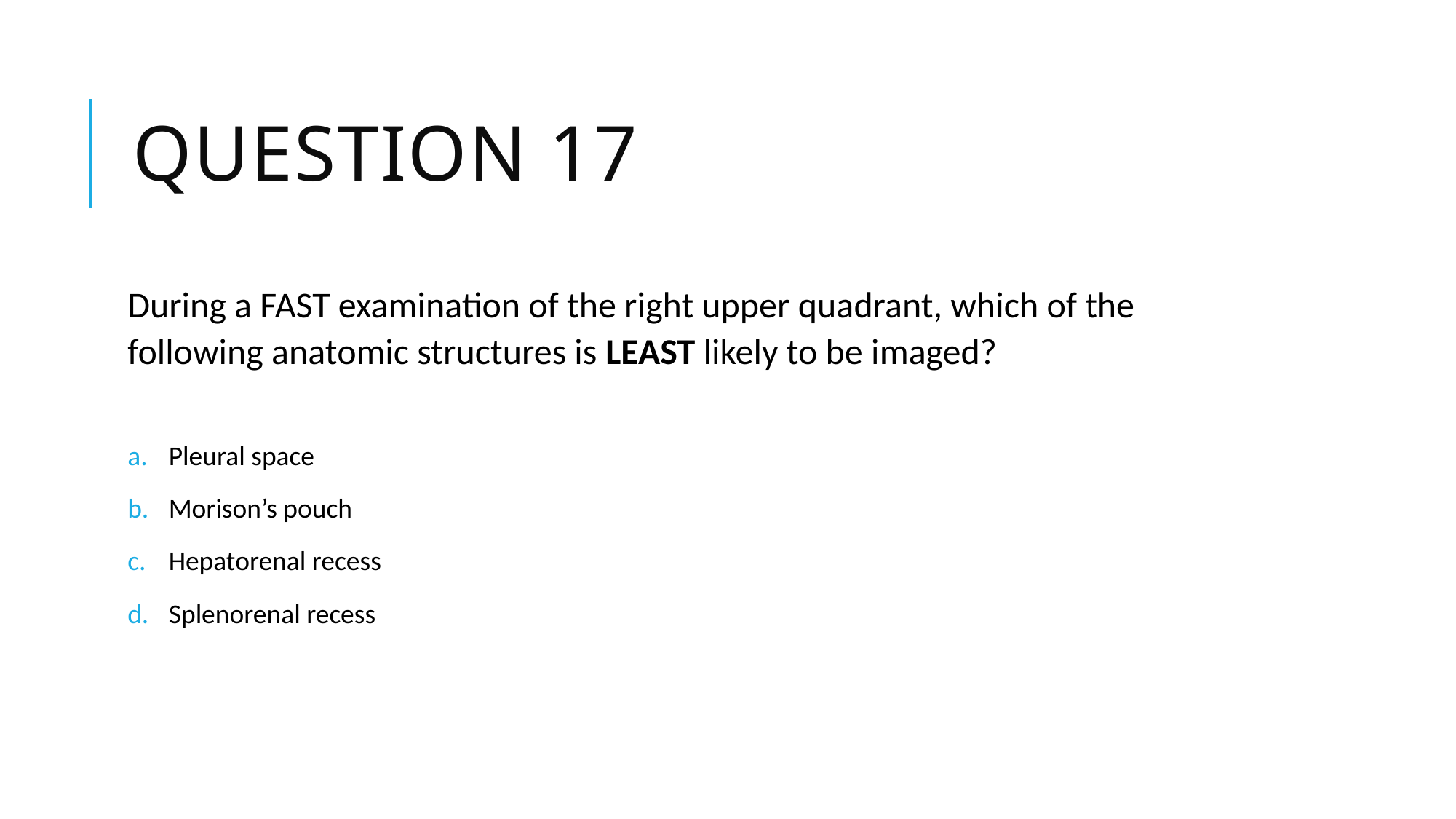

# Question 17
During a FAST examination of the right upper quadrant, which of the following anatomic structures is LEAST likely to be imaged?
Pleural space
Morison’s pouch
Hepatorenal recess
Splenorenal recess

## Slide 19
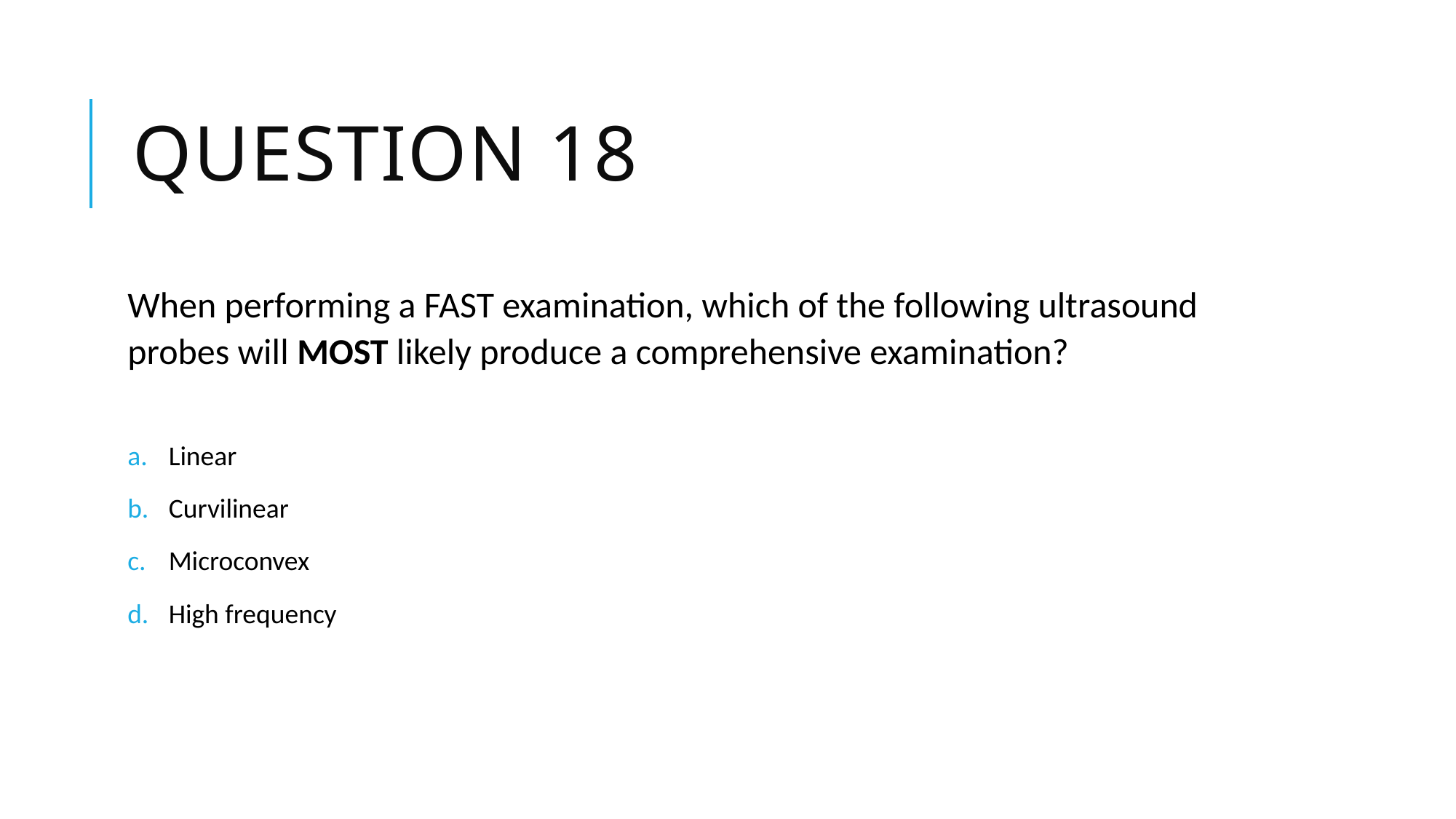

# Question 18
When performing a FAST examination, which of the following ultrasound probes will MOST likely produce a comprehensive examination?
Linear
Curvilinear
Microconvex
High frequency

## Slide 20
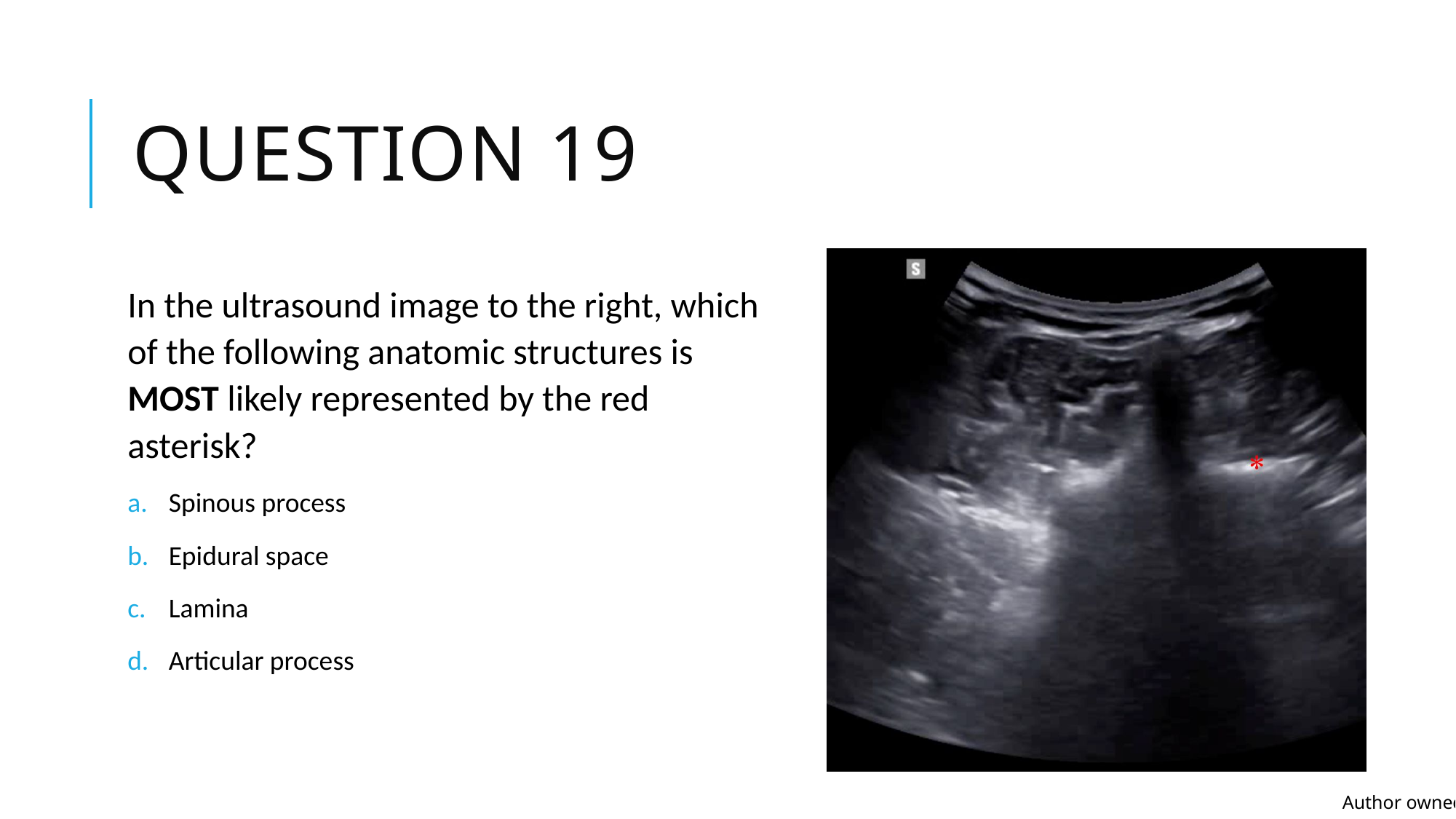

# Question 19
In the ultrasound image to the right, which of the following anatomic structures is MOST likely represented by the red asterisk?
Spinous process
Epidural space
Lamina
Articular process
Author owned

## Slide 21
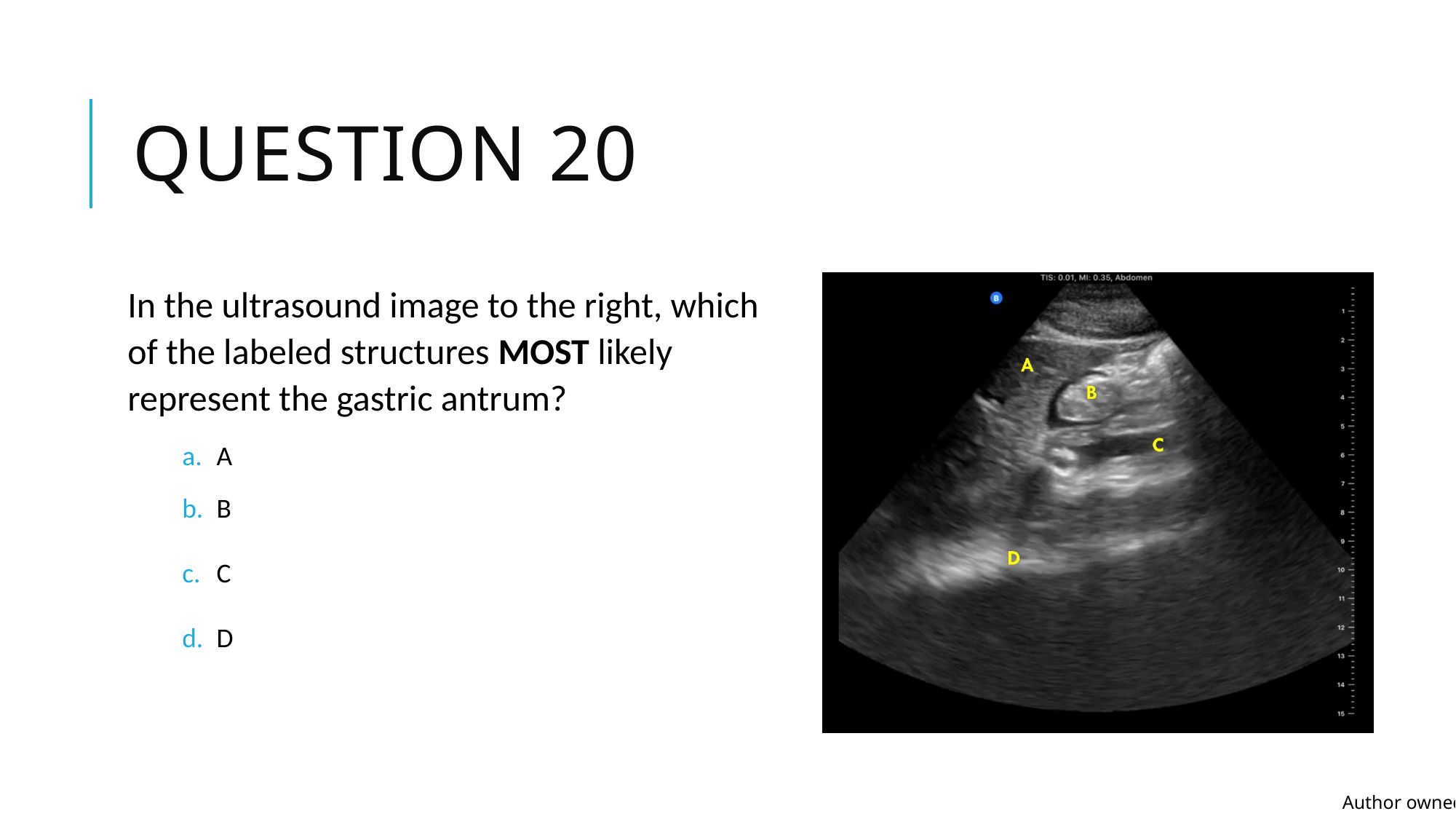

# Question 20
In the ultrasound image to the right, which of the labeled structures MOST likely represent the gastric antrum?
A
B
C
D
Author owned
