# Supplementary material for: An Introductory Point-of-Care Ultrasound Curriculum for an Anesthesiology Residency Program
Source: MedEdPORTAL. 2022 Dec 23;18:11291. doi: 10.15766/mep_2374-8265.11291 (PMC9780414; doi:10.15766/mep_2374-8265.11291)
Supplement: Supplementary file 1 — Ultrasound Basics.pptxLung Ultrasound.pptxCardiac Ultrasound.pptxVascular Access Ultrasound.pptxAirway Ultrasound.pptxAbdominal Ultrasound.pptxNeuraxial Ultrasound.pptxChecklist for POCUS Scanning.docxPOCUS CA1 Curriculum Pretest.pptxPOCUS CA1 Curriculum Posttest.pptxPOCUS Survey.docx [file mep_2374-8265.11291-s001.zip › K. POCUS Survey.docx]

POCUS Survey Questions

1. I will incorporate POCUS into my practice.
   1. Strongly disagree
   2. Disagree
   3. Neutral
   4. Agree
   5. Strongly Agree
2. The POCUS teaching and education materials were appropriate to my level of training.
   1. Strongly disagree
   2. Disagree
   3. Neutral
   4. Agree
   5. Strongly Agree
3. Please rate your level of knowledge of POCUS before this curriculum.
   1. No knowledge
   2. Little knowledge
   3. Some knowledge
   4. Sufficient knowledge
   5. Expert knowledge
4. Please rate your level of knowledge of POCUS after this curriculum.
   1. No knowledge
   2. Little knowledge
   3. Some knowledge
   4. Sufficient knowledge
   5. Expert knowledge
5. Please rate your level of POCUS technical skills before this curriculum.
   1. No technical skills
   2. Little technical skills
   3. Some technical skills
   4. Sufficient technical skills
   5. Expert technical skills
6. Please rate your level of POCUS technical skills after this curriculum.
   1. No technical skills
   2. Little technical skills
   3. Some technical skills
   4. Sufficient technical skills
   5. Expert technical skills
7. Did you learn anything new from this POCUS curriculum?
   1. Strongly disagree
   2. Disagree
   3. Neutral
   4. Agree
   5. Strongly Agree
